# Design, Synthesis, and Antileishmanial Activity of Novel C6-Substituted Eugenol Derivatives with a Triazole Linker

**DOI:** 10.1021/acsomega.5c13551

**Published:** 2026-08-12

**Authors:** Henrique P. Orenha, Fabiana M. S. U. Moncorvo, Thiago dos Santos, Guilherme Espírito Santo da Silva, Victor H. C. Fernandes, Eduardo Caio Torres-Santos, Giuliano C. Clososki

**Affiliations:** † Department of BioMolecular Sciences, 28133 School of Pharmaceutical Sciences of Ribeirão Preto − University of São Paulo, Avenida do Café s/n, Vila Monte Alegre, Ribeirão Preto, SP 14040-903, Brazil; ‡ Laboratory of Trypanosomatid Biochemistry, Oswaldo Cruz Institute − Fiocruz, Avenida Brasil 4365, Manguinhos, Rio de Janeiro, RJ 21040-360, Brazil

## Abstract

Among tropical neglected diseases, leishmaniasis stands out. This parasitic infection occurs in numerous countries, affecting thousands of people worldwide. The currently available therapies present important limitations, including high toxicity, long treatment periods requiring parenteral administration, and the emergence of parasite resistance. Based on the known antileishmanial properties of eugenol and its derivatives, a series of novel disubstituted 1,2,3-triazoles was prepared via the classical 1,3-dipolar cycloaddition reaction, employing a eugenol-azide derivative. These compounds were subsequently evaluated against *Leishmania amazonensis* (promastigotes and amastigotes). Compound **4d** emerged as the most active molecule in the series, exhibiting an IC_50_ = 3.98 ± 1.05 μM against amastigotes and a selectivity index greater than 50.25, thereby retaining and even enhancing the antileishmanial activity of its natural-product precursor.

## Graphical abstract

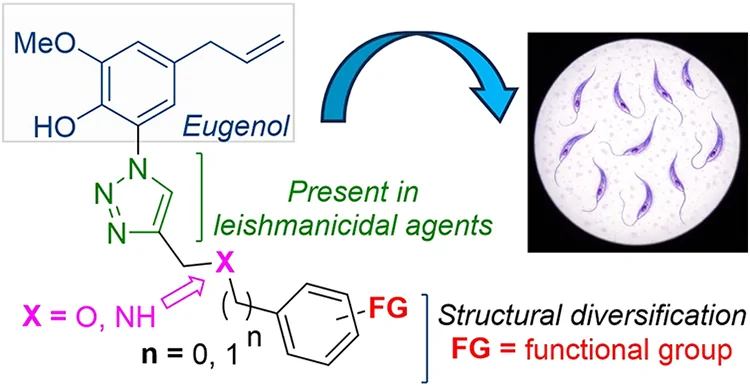

## Introduction

Currently, leishmaniasis is recognized as one of the most important neglected tropical diseases, caused by the protozoan parasites from the genus *Leishmania*.^1,2^ The life cycle involves transmission of the parasite when an infected hematophagous female sand fly takes a blood meal, inoculating promastigote forms into the host’s skin. After being phagocytosed by mononuclear phagocytic cells, such as macrophages, the parasites differentiate into amastigotes and proliferate by binary fission. The host cells eventually rupture, releasing parasites that infect additional cells of the mononuclear phagocytic system. As the infection progresses and more host cells are destroyed, the infection becomes symptomatic with varying clinical manifestations,^3^ such as visceral,^4^ cutaneous,^5^ and mucocutaneous.^2,5,6^ Epidemiological data from 2023 reported approximately 220,000 new cases of leishmaniasis worldwide, predominantly in countries of the Eastern Mediterranean region, parts of Africa, and the Americas. In the same year, Brazil recorded around 13,000 cases of cutaneous leishmaniasis and approximately 1500 cases of the visceral form.^7^

To date, pentavalent antimonials remain the first-line therapy for various forms of leishmaniasis.^8^ This class includes sodium stibogluconate^9–11^ and meglumine antimoniate^2,12–14^ (Figure 1A). Other commonly indicated antileishmanial drugs are miltefosine,^1,15^ amphotericin B,^16^ and pentamidine (Figure 1B).^17–19^ However, despite their widespread use, these therapies display significant limitations, including reduced efficacy, toxicity, prolonged treatment periods, reliance on parenteral administration,^20^ and emergence of a resistant parasitic strain.^2^

**Figure 1:**
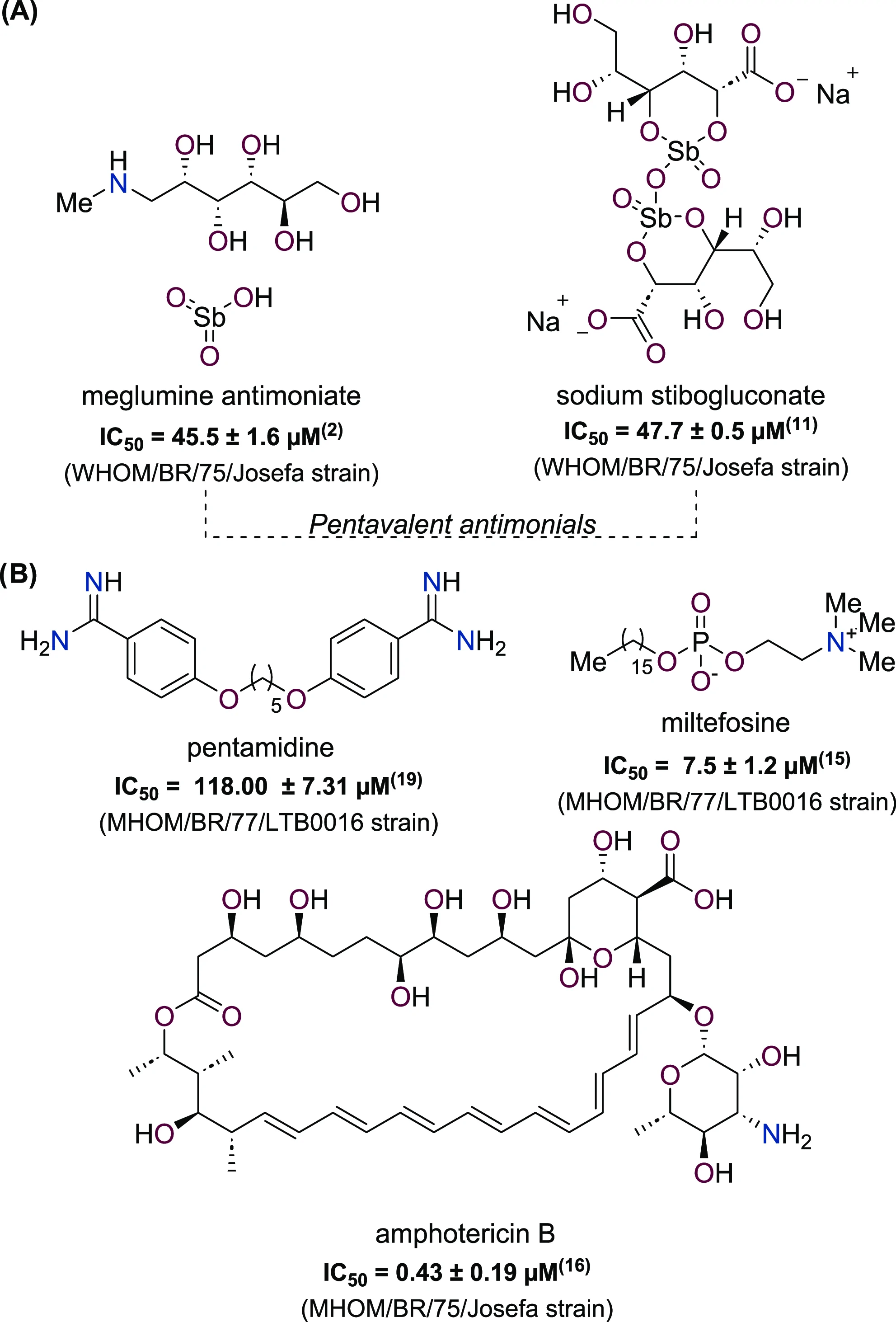
In vitro activity against the amastigote form of *Leishmania amazonensis* of (A) antimonial drugs and (B) nonantimonial drugs used for the treatment of leishmaniasis.

Thus, the search for new antileishmanial drug candidates remains an essential task.^18^

Secondary plant metabolites can be of great use in the drug discovery process, particularly regarding neglected diseases.^21^ The eugenol-rich essential oil presents several biological effects of interest, including bactericidal, antifungal, anti-inflammatory,^2,22^ and antileishmanial activities.^2,8,22,23^ It has been reported that eugenol can increase the nitric oxide levels in the host by stimulating the immune system, thereby contributing to parasite elimination.^18,20,24,25^ In this context, the observed increase in nitric oxide production appears to be linked to the upregulation of Th1-type immune responses, together with a downregulation of Th2-associated cytokines, which may contribute to the elimination of intracellular amastigotes.^26^ Molecular docking studies have highlighted favorable binding interactions of eugenol and the enzyme dihydroorotate dehydrogenase of *L. braziliensis*, which may result in mitochondrial dysfunction.^27^

A wide variety of structural derivatization strategies for eugenol have been explored,^28^ including the incorporation of various heterocyclic moieties, such as pyrazole, furan, morpholine, and triazole.^2,29^ The latter system is commonly found in anticancer, antimicrobial, tuberculostatic, antiviral, antidiabetic, antimalarial, and antileishmanial agents.^30–33^ Building on the reported pharmacological properties of eugenol and its derivatives against *Leishmania* spp. parasites and the importance of the *N* -heterocycle triazole in leishmanicidal agents, we report the design, synthesis, and biological evaluation of derivatives with 1,2,3-triazole as a linker directly connected to the eugenol backbone, while preserving the hydroxy and allylic groups without additional functionalization (Figure 2). To help rationalize the structural modifications on the phenyl ring and assess the influence of hydrophobic, electronic, and steric effects on activity, the Topliss batchwise schemes (TBS) were initially employed (APPENDIX C of the Supporting Information).^34,35^

**Figure 2:**
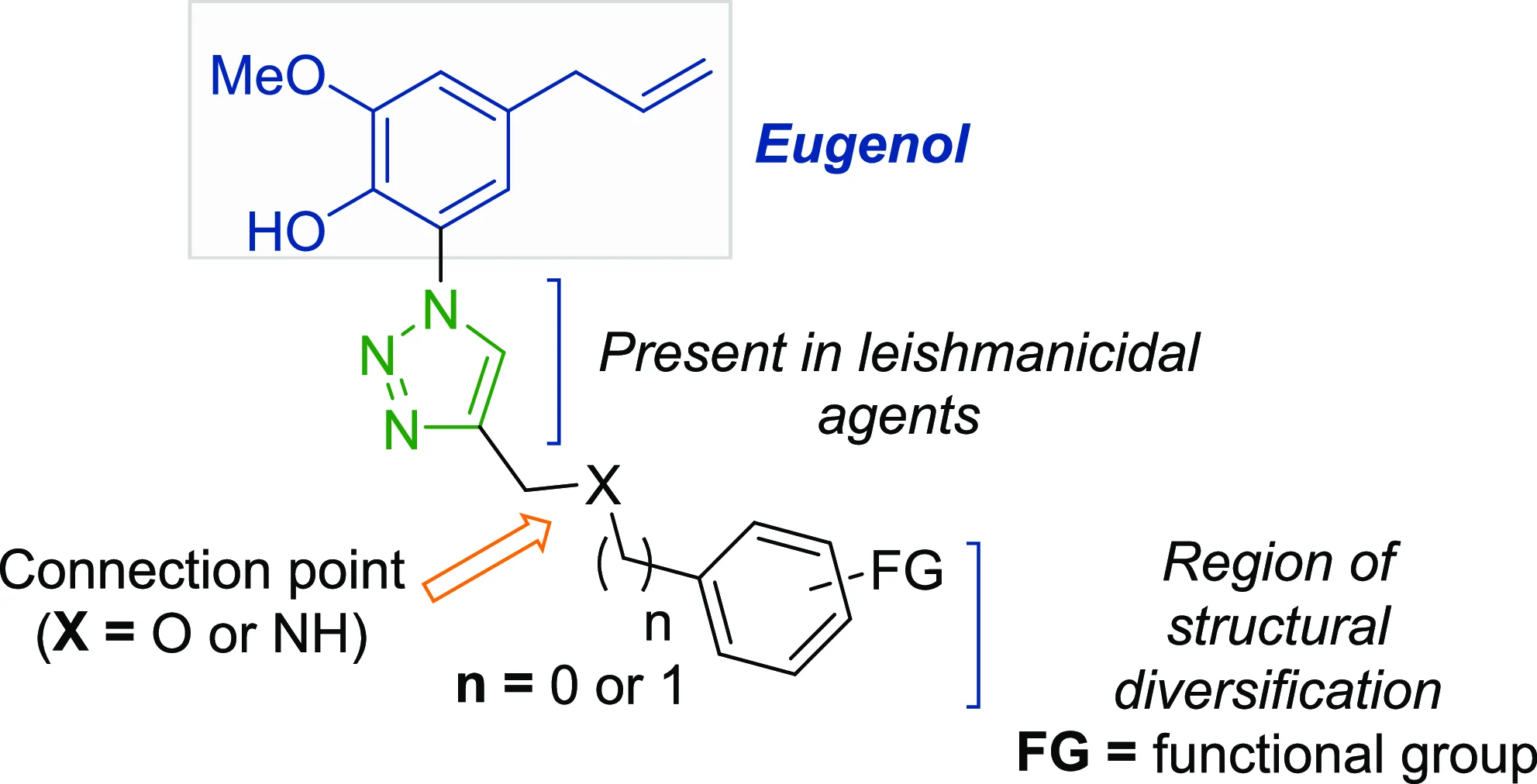
Design of novel 1,2,3-triazole-bearing eugenol derivatives.

## Results and Discussion

The eugenol-derived nitroarene **2** was successfully prepared by employing KHSO_4_ and NaNO_3_ (Scheme 1),^36^ which proved to be a safer and better alternative to procedures requiring strong acids.^37,38^ Reduction of the nitro group with zinc metal, followed by diazotization and subsequent azidation in a one-pot manner, afforded the desired azide **3**.^39^ For the preparation of the 1,2,3-triazole derivatives, a copper-catalyzed azide–alkyne cycloaddition under microwave irradiation was adopted, enabling the synthesis of compounds **4a**–**i**, **4k**–**t**, and **5** in significantly shorter reaction times (e.g., 10 min), compared with conventional batch conditions (e.g., 2.5 h), and in good to high yields (40–99%).^40,41^ The required propargyl derivatives were prepared^42–45^ from diverse anilines, phenols, and benzyl alcohols, either commercially available or obtained via synthesis.^46^

**Scheme 1:**
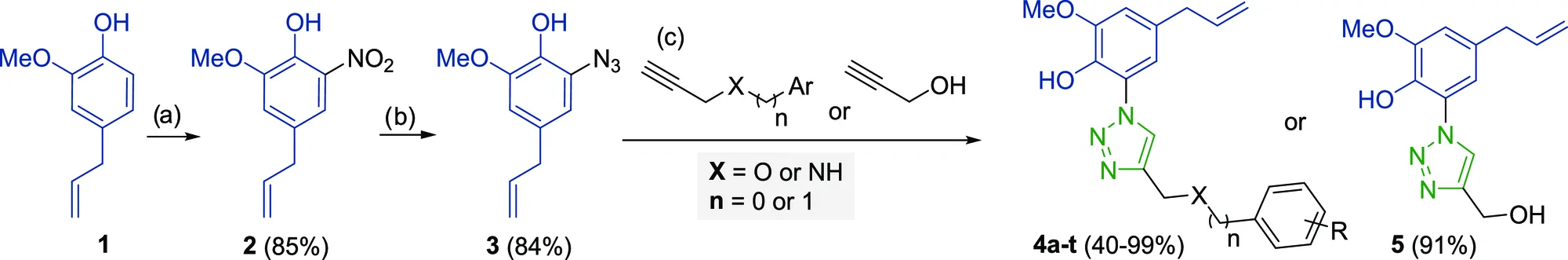
Conditions: (a) KHSO_4_ (3.6 Equiv), NaNO_3_ (4.0 Equiv), H_2_O/SiO_2_ (50% w/w), CH_2_Cl_2_, 1.5 h, 25 °C; (b) i. AcOH:H_2_O (2:3 v/v), Zn° (4.0 Equiv), 1 h, 27 °C, ii. NaNO_2_ (1.1 Equiv), 0 °C, NaN_3_ (1.5 Equiv), 50 min, from 0 to 25 °C; (c) CuSO_4_·5H_2_O (0.01 Equiv), *t*-BuOH:H_2_O (1:1 v/v), Sodium Ascorbate (0.1 Equiv), Correspondent Propargyl (1.1 Equiv), 10 min, 125 °C, 300 W (M.W.)

The initial group of benzyloxy derivatives **4a**–**e**, bearing 3,4-Cl_2_, 4-Cl, 4-Me, 4-OMe, and 4-H substituents, respectively, together with compound **5**, was subjected to in vitro assays against *L. amazonensis* promastigotes (Table 1). The 50% growth inhibitory activity values (IC_50_) were determined after 72 h of treatment with each compound. Miltefosine was employed as the positive control. The introduction of a benzyloxy group in **4a**–**e**, in place of the primary alcohol present in **5**, resulted in a significant increase in potency, yielding bioactive profiles superior to that of eugenol (IC_50_ > 128 μM). Compound **4b** proved to be the most potent derivative in this series, with an IC_50_ of 9.70 ± 1.03 μM. After calculating the potency ranking, no correspondence was found with the ranking schemes provided by Topliss (π, α, −α, Es, π + α, 2π – α, π – α, π – 2α, π – 3α, and 2π + π^2^).^34,35^

**Table 1:**
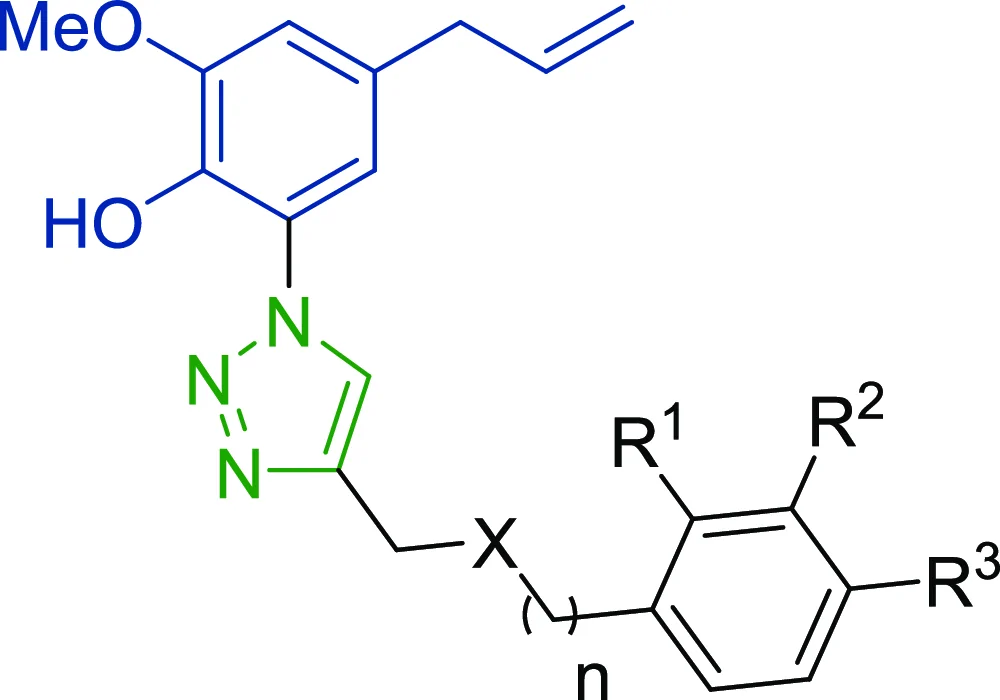
In Vitro Evaluation of the Novel C6-Substituted Eugenol Derivatives against Promastigote and Amastigote Forms of *L. amazonensis* after 72 h of Treatment, and Their Cytotoxicity against Murine Macrophages

compound	X	R^1^	R^2^	R^3^	*n*	promastigote IC_50_ ± SD (μM)[Table-fn t1fn1]	amastigote IC_50_ ± SD (μM)[Table-fn t1fn1]	murine macrophages CC_50_ (μM)[Table-fn t1fn2]	SI CC_50_/IC_50_ ^c^
**4a**	O	H	Cl	Cl	1	16.90 ± 1.04	n.d.	n.d.	n.d.
**4b**	O	H	H	Cl	1	9.70 ± 1.03	5.12 ± 1.50	79.10 ± 1.07	15.45
**4c**	O	H	H	Me	1	10.30 ± 1.04	5.73 ± 0.54	>200	>34.90
**4d**	O	H	H	OMe	1	11.60 ± 1.08	3.98 ± 1.05	>200	>50.25
**4e**	O	H	H	H	1	14.50 ± 1.05	n.d.	n.d.	n.d.
**5**	OH					>128	n.d.	n.d.	n.d.
**4f**	O	H	Cl	Cl	0	31.91 ± 1.12	n.d.	n.d.	n.d.
**4g**	O	H	H	Cl	0	18.95 ± 1.04	n.d.	n.d.	n.d.
**4h**	O	H	H	Me	0	27.25 ± 1.03	n.d.	n.d.	n.d.
**4i**	O	H	H	OMe	0	22.60 ± 1.04	n.d.	n.d.	n.d.
**4j**	O	H	H	H	0	16.40 ± 1.02	n.d.	65.08 ± 1.10	n.d.
**4k**	O	H	Me	H	0	17.30 ± 1.03	n.d.	65.72 ± 1.06	n.d.
**4l**	NH	H	Cl	Cl	0	18.50 ± 1.03	n.d.	99.10 ± 1.16	n.d.
**4m**	NH	H	H	Me	0	>128	n.d.	n.d.	n.d.
**4n**	NH	H	H	H	0	63.00 ± 1.04	n.d.	n.d.	n.d.
**4o**	NH	H	H	NO_2_	0	97.70 ± 1.16	n.d.	n.d.	n.d.
**4p**	NH	H	H	Br	0	>128	n.d.	n.d.	n.d.
**4q**	NH	F	H	F	0	27.20 ± 1.04	n.d.	n.d.	n.d.
**4r**	NH	H	Cl	H	0	>128	n.d.	n.d.	n.d.
**4s**	NH	Me	H	H	0	35.80 ± 1.02	n.d.	n.d.	n.d.
**4t**	NH	OMe	H	H	0	37.20 ± 1.03	n.d.	n.d.	n.d.
**eugenol**						>128	n.d.	n.d.	n.d.
**miltefosine** ^d^						5.04 ± 0.45	7.5 ± 1.2	109.7 ± 2.0	14.6

a,b IC_50_, 50% inhibitory concentration.

c CC_50_, 50% cytotoxic concentration.

d SI: Selectivity index in relation to the amastigote in vitro assay. Data are expressed as mean ± standard error of the mean (SEM) of three independent experiments performed in triplicate. IC_50_ values were calculated by nonlinear regression using GraphPad Prism software. Statistical significance was determined by one-way ANOVA followed by Bonferroni’s multiple comparison test, with *p* < 0.05 considered significant. n.d. = Not determined.

e Miltefosine data were previously reported by our group under identical experimental conditions and are included here for comparison.^15^

To assess the influence of the second methylene group on activity, the second series (**4f**–**j**) was prepared and evaluated. A reduction in potency was observed across almost all derivatives. Notably, compound **4g** (IC_50_ of 18.95 ± 1.04 μM) was approximately 2-fold less potent than its counterpart derivative **4b**. The 3-Me-substituted derivative **4k** showed better antileishmanial activity than **4h** but still not greater than **4j**.

Furthermore, the bioisosteric replacement of oxygen by the amino group in **4l**–**n** was investigated. Except for **4l** with an increment in leishmanicidal activity (IC_50_ of 18.50 ± 1.03 μM) compared with **4f**, no improvement was observed for the pairs **4h**–**4m** and **4j**–**4n**, nor for the other evaluated derivatives (**4o**–**t**).

After identifying compounds **4b**–**d** as the most active against extracellular promastigotes of *L. amazonensis* (Table 1), they were further evaluated against intracellular amastigotes of the same species. In addition, their cytotoxicity profiles toward uninfected murine intraperitoneal macrophages were determined. Compound **4d** proved to be the least toxic with a SI (Selective Index) > 50.25 and the most active against amastigotes (IC_50_ = 3.98 ± 1.05).

An in silico analysis of physicochemical parameters, including Lipinski’s rule of five,^47^ Veber’s,^48^ and Muegge’s drug-likeness criteria,^49^ as well as ADMET properties (absorption, distribution, metabolism, excretion, and toxicity), was performed for compounds **4b**–**d** using the online tools pkCSM and SwissADME (Tables 2–4). The physicochemical evaluation (Table 2) indicates that compounds **4b**–**d** fully comply with Lipinski’s rule of five, as well as Veber’s and Muegge’s criteria for oral drug-likeness. In addition, these derivatives are predicted to present moderate-to-low aqueous solubility, which is compatible with their moderately lipophilic profiles.

**Table 2:** Physicochemical Parameters and Drug-Likeness Evaluation Based on Lipinski’s Rule of Five, Veber’s, and Muegge’s Criteria for Compounds **4b**–**d** and **7k**, Predicted Using the pkCSM and SwissADME Tools^2^

descriptors/compounds	ideal values: Lipinski’s rule	ideal values: Veber’s rule	ideal values: Muegge’s rule	**4b**	**4c**	**4d**	**7k** ^a^
molecular weight (Da)	≤500		200 ≤ MW ≤ 600	385.851	365.433	381.432	377.488
log *P*	≤5		–2 ≤ clog *P* ≤ 5	4.0802	3.73522	3.4354	4.38352
#rotatable bonds		≤10	≤15	8	8	8	10
#acceptors	≤10		≤10	5	5	5	4
#donors	≤5		≤5	1	1	1	0
water solubility (log, mg/L)				–4.311	–4.187	–4.21	–5.843
TPSA (Å^2^)		≤140	≤150	69.40	69.40	69.40	49.17

a With compliance with the Lipinski rule, there is a lower predicted aqueous solubility.

**Table 3:** In Silico ADMET Properties of **4b**–**d** and **7k** Using the pkCSM Tool^2^

	**4b**	**4c**	**4d**	**7k** ^2^
Absorption				
permeability Caco-2 (cm/s)	1.179	1.189	1.359	1.012
intestinal absorption (human, %)	93.833	95.292	95.59	97.771
skin permeability (logKp)	–2.83	–2.835	–2.736	–2.56
Distribution				
VDss (human, logL/kg)	0.099	0.138	–0.075	0.246
unbound fraction (human)	0.074	0.085	0.052	0.037
BHE permeability (logBBB)	–0.64	–0.464	–0.36	–0.136
SNC permeability (logPS)	–2.459	–2.5	–3.229	–2.392
Metabolism				
CYP2D6 (substrate)	no	no	no	no
CYP3A4 (substrate)	yes	yes	yes	yes
CYP1A2 (inhibitor)	yes	yes	no	yes
CYP2C19 (inhibitor)	yes	yes	yes	yes
CYP2C9 (inhibitor)	yes	yes	yes	yes
CYP2D6 (inhibitor)	no	no	no	no
CYP3A4 (inhibitor)	yes	yes	yes	yes
Excretion				
total clearance (mL/min/kg)	0.296	0.305	0.339	0.335

**Table 4:** In Silico Toxicity of **4b**–**d** and **7k** Using the pkCSM Tool^2^

	**4b**	**4c**	**4d**	**7k** ^2^
AMES toxicity test	no	no	no	no
maximum tolerated dose (human, mg/kg/day)	0.495	0.477	0.763	0.497
hERG I (inhibitor)	no	no	no	no
hERG II (inhibitor)	yes	yes	yes	yes
acute oral toxicity mouse (LD_50_) (mol/kg)	2.747	2.697	2.108	2.128
chronic oral toxicity in mouse (LOAEL) (mg/kg_bw/day)	1.387	1.614	1.3	1.88
hepatotoxicity	yes	yes	yes	yes
skin sensitization	no	no	no	no
toxicity in *Tetrahymena pyriformis*	0.461	0.479	0.302	0.736
IC_50_ (μg/L)				
toxicity minnow	0.056	0.274	–1.379	–1.147
LC_50_ (mM)				

To contextualize the present findings, we compared series **4** with the structurally related series **7** previously reported by Teixeira et al.^2^ Both series feature 1,2,3-triazole-eugenol conjugates but differ in key structural aspects: series **4** contains the triazole nucleus directly attached to the aromatic ring via a C–N bond while preserving the free phenolic hydroxyl group, whereas series **7** incorporates a three-carbon alkyl spacer between the triazole and the etherified eugenol moiety. Direct comparison of compounds bearing identical *para*-substituents revealed distinct structure–activity relationship (SAR) profiles. For the chloro derivatives, compound **4b** (IC_50_ = 9.7 μM) displayed ∼84-fold higher potency than **7c** (IC_50_ = 817 μM) in promastigotes, suggesting that hydrogen-bonding interactions mediated by the free phenolic hydroxyl group play a critical role in molecular recognition. Conversely, for the methyl derivatives, compound **7k** (IC_50_ = 7.4 μM against promastigotes; 1.6 μM against amastigotes) was more potent than **4c** (IC_50_ = 10.3 and 5.73 μM, respectively), indicating that enhanced conformational flexibility and optimized hydrophobic interactions can compensate for the absence of specific polar contacts.

The in silico ADMET comparison (Tables 2–4) revealed important differences between the two series. Series **4** derivatives exhibited higher polarity (HBD = 1, HBA = 5) and generally lower predicted CNS penetration compared to **7k** (HBD = 0, HBA = 4, logBB = −0.136). While all compounds showed predicted hepatotoxicity and CYP inhibition liability, compounds **4d** and **7k** were also predicted to inhibit the hERG II channel, which may indicate a potential cardiotoxicity concern requiring further experimental investigation (Table 4). In addition, **7k** displayed lower predicted aqueous solubility, indicating a potentially narrower safety margin.

Overall, series **7** achieves a higher maximum potency through hydrophobic optimization but displays greater structural sensitivity and less favorable ADMET properties. In contrast, series **4** demonstrates more consistent activity, exploits complementary polar interactions, and presents a more balanced pharmacological profile, making it a robust scaffold for further rational optimization.

Regarding the in silico ADMET analysis (Table 3), the compounds exhibited good predicted permeability in Caco-2 cells, with values above the adopted threshold of 0.9 for all derivatives. They also showed a high probability of human intestinal absorption, with predicted values exceeding 90%, within the reference range of 66.9–99.2%.

Compound **4d** presented a low volume of distribution (logVDss < −0.15), suggesting preferential distribution in the plasma compartment with significant protein binding. Metabolism predictions indicate that derivative **4b** is likely to inhibit CYP3A4, CYP1A2, CYP2C19, and CYP2C9.

In summary, twenty-one novel triazole derivatives based on the eugenol scaffold were synthesized in good to high yields and fully characterized by NMR (^1^H, ^13^C, ^13^C DEPT-135, HSQC) and HRMS analyses. The established one-pot reduction, diazotization, and azidation sequence, along with the copper-catalyzed 1,3-dipolar cycloaddition reactions under microwave irradiation, were decisive for obtaining the target compounds. Regarding the SAR studies, the benzyloxy-substituted derivatives displayed the most promising antileishmanial profiles against both parasite forms, highlighting the importance of the additional methylene group. In contrast, replacing the benzyloxy unit with a phenyloxy group or performing a bioisosteric substitution of the ether moiety with an amino group proved detrimental, resulting in decreased potency. Among the most active compounds, **4d** stood out with a selectivity index >50.25 and IC_50_ values of 11.60 ± 1.08 and 3.98 ± 1.05 μM against promastigotes and amastigotes of *L. amazonensis*, respectively. Moreover, as a eugenol-derived structure, compound **4d** retained the intrinsic antileishmanial activity of the natural product while exhibiting enhanced potency.

## Supplementary Material



## Notes

S1. Supporting figures, tables and compound’s characterization data; biological assays and computational experiments procedures (PDF)
