# Supplementary material for: Design, Synthesis, and Antileishmanial Activity of Novel C6-Substituted Eugenol Derivatives with a Triazole Linker
Source: ACS Omega. 2026 Aug 12;11(33):49026–32. doi: 10.1021/acsomega.5c13551 (PMC13520032; doi:10.1021/acsomega.5c13551)
Supplement: Supplementary file 1 [file ao5c13551_si_001.pdf]

Supporting Information

Design, Synthesis, and Antileishmanial Activity of Novel C6-Substituted Eugenol Derivatives with a Triazole Linker

Henrique Pereira Orenha,[1] Fabiana Maia Santos Urbancg Moncorvo,[2] Thiago dos Santos,[1] Guilherme Espírito Santo da Silva,[2] Victor H. C. Fernandes,[1] Eduardo Caio Torres-Santos,[2*] Giuliano Cesar Clososki[1*]

[1] Department of BioMolecular Sciences, School of Pharmaceutical Sciences of Ribeirão Preto – University of São Paulo. Avenida do Café s/n, Vila Monte Alegre. Ribeirão Preto, SP, 14040-903, Brazil.

[2] Laboratory of Trypanosomatid Biochemistry, Oswaldo Cruz Institute – Fiocruz. Avenida Brasil 4365, Manguinhos. Rio de Janeiro, RJ, 21040-360, Brazil.

Corresponding Authors

1. Giuliano Cesar Clososki

   Department of BioMolecular Sciences, School of Pharmaceutical Sciences of Ribeirão Preto – University of São Paulo. Avenida do Café s/n, Vila Monte Alegre. Ribeirão Preto, SP, 14040-903, Brazil.

   E-mail id: gclososki@usp.br

   ORCID: 0000-0001-7605-4150

2. Eduardo Caio Torres-Santos

   Trypanosomatid Biochemistry Laboratory, Oswaldo Cruz Institute – Fiocruz. Avenida Brasil 4365, Manguinhos. Rio de Janeiro, RJ, 21040-360, Brazil.

   E-mail id: ects@ioc.fiocruz.br

   ORCID: 0000-0003-2240-4519

Table of Contents

APPENDIX A: Chemistry………………………………………………………….... S8

APPENDIX B: Biology…………………………………………………………........S22

APPENDIX C: Medicinal Chemistry………………………………………….............S23

Figure S1. Diagram of *Topliss Batchwise* used for eugenol derivatives…………....…S23

APPENDIX D: Nuclear Magnetic Resonance Spectra of ^{1}H, ^{13}C, ^{13}C DEPT--135 and ^{1}H/^{13}C HSQC…………………………………………………….......................S24

Figure S2. ^{1}H NMR of **1** (500 MHz, $CDCl_3$)…………………………………………S24

Figure S3. ^{13}C NMR of **1** (126 MHz, $CDCl_3$)……………………………….……S24

Figure S4. ^{13}C NMR DEPT--135 of **1** (126 MHz, $CDCl_3$)……………………………S25

Figure S5. ^{1}H NMR of **2** (400 MHz, $CDCl_3$)…………………………………………S25

Figure S6. ^{13}C NMR of **2** (101 MHz, $CDCl_3$)……………………………………...…S26

Figure S7. ^{13}C NMR DEPT--135 of **2** (101 MHz, $CDCl_3$)……………………………S26

Figure S8. ^{1}H NMR of **3** (400 MHz, $CDCl_3$)…………………………………………S27

Figure S9. ^{13}C NMR of **3** (101 MHz, $CDCl_3$)………………………...………S27

Figure S10. ^{13}C NMR DEPT--135 of **3** (101 MHz, $CDCl_3$)……………….…………S28

Figure S11. ^{1}H NMR of **4a** (400 MHz, $CDCl_3$)…………………………….………S28

Figure S12. ^{13}C NMR of **4a** (101 MHz, $CDCl_3$)……………………………...……S29

Figure S13. ^{13}C NMR DEPT--135 of **4a** (101 MHz, $CDCl_3$)…………………………S29

Figure S14. ^{1}H/^{13}C NMR HSQC of **4a** (400 and 101 MHz, $CDCl_3$)………………….…S30

Figure S15. ^{1}H NMR of **4b** (400 MHz, $CDCl_3$)………………………………………S30

Figure S16. ^{13}C NMR of **4b** (101 MHz, $CDCl_3$)……………………………...……S31

Figure S17. ^{13}C NMR DEPT--135 of **4b** (101 MHz, $CDCl_3$)…………………………S31

Figure S18. ^{1}H/^{13}C NMR HSQC of **4b** (400 and 101 MHz, $CDCl_3$)…………….……S32

Figure S19. ^{1}H NMR of **4c** (400 MHz, $CDCl_3$)……………………………………S32

Figure S20. ^{13}C NMR of **4c** (101 MHz, $CDCl_3$)……………………………………S33

Figure S21. ^{13}C NMR DEPT--135 of **4c** (101 MHz, $CDCl_3$)……………………………S33

Figure S22. ^{1}H/^{13}C NMR HSQC of **4c** (400 and 101 MHz, $CDCl_3$)…………………S34

Figure S23. ^{1}H NMR of **4d** (400 MHz, $CDCl_3$)……………………………………S34

Figure S24. ^{13}C NMR of **4d** (101 MHz, $CDCl_3$)……………………………………S35

Figure S25. ^{13}C NMR DEPT--135 of **4d** (101 MHz, $CDCl_3$)……………………………S35

Figure S26. ^{1}H/^{13}C NMR HSQC of **4d** (400 and 101 MHz, $CDCl_3$)…………………S36

Figure S27. ^{1}H NMR of **4e** (400 MHz, $CDCl_3$)……………………………………S36

Figure S28. ^{13}C NMR of **4e** (101 MHz, $CDCl_3$)……………………………………S37

Figure S29. ^{13}C NMR DEPT--135 of **4e** (101 MHz, $CDCl_3$)……………………………S37

Figure S30. ^{1}H/^{13}C NMR HSQC of **4e** (400 and 101 MHz, $CDCl_3$)…………………S38

Figure S31. ^{1}H NMR of **4f** (400 MHz, $CDCl_3$)……………………………………S38

Figure S32. ^{13}C NMR of **4f** (101 MHz, $CDCl_3$)……………………………………S39

Figure S33. ^{13}C NMR DEPT--135 of **4f** (101 MHz, $CDCl_3$)……………………………S39

Figure S34. ^{1}H/^{13}C NMR HSQC of **4f** (400 and 101 MHz, $CDCl_3$)…………………S40

Figure S35. ^{1}H NMR of **4g** (400 MHz, $CDCl_3$)……………………………………S40

Figure S36. ^{13}C NMR of **4g** (101 MHz, $CDCl_3$)……………………………………S41

Figure S37. ^{13}C NMR DEPT--135 of **4g** (101 MHz, $CDCl_3$)……………………………S41

Figure S38. ^{1}H/^{13}C NMR HSQC of **4g** (400 and 101 MHz, $CDCl_3$)…………………S42

Figure S39. ^{1}H NMR of **4h** (400 MHz, $CDCl_3$)……………………………………S42

Figure S40. ^{13}C NMR of **4h** (101 MHz, $CDCl_3$)……………………………………S43

Figure S41. ^{13}C NMR DEPT--135 of **4h** (101 MHz, $CDCl_3$)……………………………S43

Figure S42. ^{1}H/^{13}C NMR HSQC of **4h** (400 and 101 MHz, $CDCl_3$)…………………S44

Figure S43. ^{1}H NMR of **4i** (400 MHz, $CDCl_3$)……S44

Figure S44. ^{13}C NMR of **4i** (101 MHz, $CDCl_3$)……S45

Figure S45. ^{13}C NMR DEPT--135 of **4i** (101 MHz, $CDCl_3$)……S45

Figure S46. ^{1}H/^{13}C NMR HSQC of **4i** (400 and 101 MHz, $CDCl_3$)……S46

Figure S47. ^{1}H NMR of **4j** (400 MHz, $CDCl_3$)……S46

Figure S48. ^{13}C NMR of **4j** (101 MHz, $CDCl_3$)……S47

Figure S49. ^{13}C NMR DEPT--135 of **4j** (101 MHz, $CDCl_3$)……S47

Figure S50. ^{1}H/^{13}C NMR HSQC of **4j** (400 and 101 MHz, $CDCl_3$)……S48

Figure S51. ^{1}H NMR of **4k** (400 MHz, $CDCl_3$)……S48

Figure S52. ^{13}C NMR of **4k** (101 MHz, $CDCl_3$)……S49

Figure S53. ^{13}C NMR DEPT--135 of **4k** (101 MHz, $CDCl_3$)……S49

Figure S54. ^{1}H/^{13}C NMR HSQC of **4k** (400 and 101 MHz, $CDCl_3$)……S50

Figure S55. ^{1}H NMR of **4l** (400 MHz, $CDCl_3$)……S50

Figure S56. ^{13}C NMR of **4l** (101 MHz, $CDCl_3$)……S51

Figure S57. ^{13}C NMR DEPT--135 of **4l** (101 MHz, $CDCl_3$)……S51

Figure S58. ^{1}H/^{13}C NMR HSQC of **4l** (400 and 101 MHz, $CDCl_3$)……S52

Figure S59. ^{1}H NMR of **4m** (500 MHz, $CDCl_3$)……S52

Figure S60. ^{13}C NMR of **4m** (126 MHz, $CDCl_3$)……S53

Figure S61. ^{13}C NMR DEPT--135 of **4m** (126 MHz, $CDCl_3$)……S53

Figure S62. ^{1}H/^{13}C NMR HSQC of **4m** (500 and 126 MHz, $CDCl_3$)……S54

Figure S63. ^{1}H NMR of **4n** (400 MHz, $CDCl_3$)……S54

Figure S64. ^{13}C NMR of **4n** (101 MHz, $CDCl_3$)……S55

Figure S65. ^{13}C NMR DEPT--135 of **4n** (101 MHz, $CDCl_3$)……S55

Figure S66. ^{1}H/^{13}C NMR HSQC of **4n** (400 and 101 MHz, $CDCl_3$)……S56

Figure S67. ^{1}H NMR of **4o** (500 MHz, DMSO)…………………...………………S56

Figure S68. ^{13}C NMR of **4o** (126 MHz, DMSO)……………………..……………S57

Figure S69. ^{13}C NMR DEPT--135 of **4o** (126 MHz, DMSO)……………...…………S57

Figure S70. $^{1}H/^{13}C$ NMR HSQC of **4o** (500 and 126 MHz, DMSO)…………………S58

Figure S71. ^{1}H NMR of **4p** (400 MHz, DMSO)……………………………...……S58

Figure S72. ^{13}C NMR of **4p** (101 MHz, DMSO)………………………………..…S59

Figure S73. ^{13}C NMR DEPT--135 of **4p** (101 MHz, DMSO)………...………………S59

Figure S74. $^{1}H/^{13}C$ NMR HSQC of **4p** (400 and 101 MHz, DMSO)…….……………S60

Figure S75. ^{1}H NMR of **4q** (500 MHz, $CDCl_3$)………………………………………S60

Figure S76. ^{13}C NMR of **4q** (126 MHz, $CDCl_3$)……………………………...……S61

Figure S77. ^{13}C NMR DEPT--135 of **4q** (126 MHz, $CDCl_3$)…………………………S61

Figure S78. $^{1}H/^{13}C$ NMR HSQC of **4q** (500 and 126 MHz, $CDCl_3$)………………..…S62

Figure S79. ^{1}H NMR of **4r** (400 MHz, $CDCl_3$)………………………………….……S62

Figure S80. ^{13}C NMR of **4r** (101 MHz, $CDCl_3$)……………………………...……S63

Figure S81. ^{13}C NMR DEPT--135 of **4r** (101 MHz, $CDCl_3$)…………………………S63

Figure S82. $^{1}H/^{13}C$ NMR HSQC of **4r** (400 and 101 MHz, $CDCl_3$)…………….……S64

Figure S83. ^{1}H NMR of **4s** (400 MHz, $CDCl_3$)………………………………….……S64

Figure S84. ^{13}C NMR of **4s** (101 MHz, $CDCl_3$)………………………………………S65

Figure S85. ^{13}C NMR DEPT--135 of **4s** (101 MHz, $CDCl_3$)……………………….…S65

Figure S86. $^{1}H/^{13}C$ NMR HSQC of **4s** (400 and 101 MHz, $CDCl_3$)………………..…S66

Figure S87. ^{1}H NMR of **4t** (400 MHz, $CDCl_3$)…………………………………..…S66

Figure 88. ^{13}C NMR of **4t** (101 MHz, $CDCl_3$)…………………………………...…S67

Figure S89. ^{13}C NMR DEPT--135 of **4t** (101 MHz, $CDCl_3$)……………………….…S67

Figure S90. $^{1}H/^{13}C$ NMR HSQC of **4t** (400 and 101 MHz, $CDCl_3$)………………..…S68

Figure S91. ^{1}H NMR of **5** (400 MHz, DMSO)……………………….………………S68

Figure S92. ^{1}H NMR of **5** (400 MHz, CD_3OD)………………………………………S69

Figure S93. ^{13}C NMR of **5** (101 MHz, DMSO)………………………………………S69

Figure S94. ^{13}C NMR DEPT--135 of **5** (101 MHz, DMSO)…………….…………S70

APPENDIX E: High Resolution Mass Spectrometry (ISE) Spectra……………………………………………………………..……...S71

Figure S95. Ionization Spectrum of **4a** (HRMS ESI-TOF)..…………………………..…S71

Figure S96. Ionization Spectrum of **4b** (HRMS ESI-TOF)..……………… …..……S72

Figure S97. Ionization Spectrum of **4c** (HRMS ESI-TOF)..………… …….…………S72

Figure S98. Ionization Spectrum of **4d** (HRMS ESI-TOF)..……………… ……..……S73

Figure S99. Ionization Spectrum of **4e** (HRMS ESI-TOF)..…………… …….………S73

Figure S100. Ionization Spectrum of **4f** (HRMS ESI-TOF)..…………… …...………S74

Figure S101. Ionization Spectrum of **4g** (HRMS ESI-TOF)..…………… …...………S74

Figure S102. Ionization Spectrum of **4h** (HRMS ESI-TOF)..……………...…………S75

Figure S103. Ionization Spectrum of **4i** (HRMS ESI-TOF)..…………………………S75

Figure S104. Ionization Spectrum of **4j** (HRMS ESI-TOF)..…………………………S76

Figure S105. Ionization Spectrum of **4k** (HRMS ESI-TOF)..……………...…………S76

Figure S106. Ionization Spectrum of **4l** (HRMS ESI-TOF)..…………………………S77

Figure S107. Ionization Spectrum of **4m** (HRMS ESI-TOF)……………...…………S77

Figure S108. Ionization Spectrum of **4n** (HRMS ESI-TOF)..………………...………S78

Figure S109. Ionization Spectrum of **4o** (HRMS ESI-TOF)..…………………………S78

Figure S110. Ionization Spectrum of **4p** (HRMS ESI-TOF)..…………………………S79

Figure S111. Ionization Spectrum of **4q** (HRMS ESI-TOF)..…………………………S79

Figure S112. Ionization Spectrum of **4r** (HRMS ESI-TOF)..…………………………S80

Figure S113. Ionization Spectrum of **4s** (HRMS ESI-TOF)..…………………………S80

Figure S114. Ionization Spectrum of **4t** (HRMS ESI-TOF)..…………………………S81

Figure S115. Ionization Spectrum of **5** (HRMS ESI-TOF)..……………..……………S81

APPENDIX F: High-pressure Liquid Chromatography ……………………………………...…………………………………………....S82

Figure S116. HPLC Chromatogram of: 0.1% formic acid in H_2O, and MeOH………..S82

Figure S117. HPLC Chromatogram of **4a**. ……………………………………..……S83

Figure S118. HPLC Chromatogram of **4b**. ……………….……………….…………S83

Figure S119. HPLC Chromatogram of **4c**. ………………………...……………S84

Figure S120. HPLC Chromatogram of **4d**. ……………………….……………S84

Figure S121. HPLC Chromatogram of **4e**. ………………………….…………S85

Figure S122. HPLC Chromatogram of **4f**. ………………………...………S85

Figure S123. HPLC Chromatogram of **4g**. ………………………….…………S86

Figure S124. HPLC Chromatogram of **4h**. ……………………….……………S86

Figure S125. HPLC Chromatogram of **4i**. ………………………...……………S87

Figure S126. HPLC Chromatogram of **4j**. ………………………...……………S87

Figure S127. HPLC Chromatogram of **4k**. ……………………….……………S88

Figure S128. HPLC Chromatogram of **4l**. ………………………...…………S88

Figure S129. HPLC Chromatogram of **4m**. ……………………….……………S89

Figure S130. HPLC Chromatogram of **4n**. ………………………….………S89

Figure S131. HPLC Chromatogram of **4o**. ……………………………..……S90

Figure S132. HPLC Chromatogram of **4p**. ………………………….………S90

Figure S133. HPLC Chromatogram of **4q**. ………………………….…………S91

Figure S134. HPLC Chromatogram of **4r**. ………………………….…………S91

Figure S135. HPLC Chromatogram of **4s**. ………………………...…………S92

Figure S136. HPLC Chromatogram of **4t**. …………………………...………S92

Figure S137. HPLC Chromatogram of **5**. ……………………………………S93

APPENDIX G: Parasite growth inhibition profile of *L. amazonensis* promastigotes……………………………...……………………………....S94

Figure S138. Inhibition curves of test compounds against *L. amazonensis*……………………………………………………………..…...S94-95

References……………………………………….…………………………...S95

Experimental Procedures

APPENDIX A: Chemistry

All solvents were purified according to standard procedures.[1] The starting material, and all reactants were purchased from Sigma-Aldrich Corp. Reactions were monitored by thin layer chromatography on Fluka Analytical silica gel (silica gel matrix, with fluorescent indicator 254 nm) by using UV light and gas chromatography on Shimadzu GC-2014 with capillary column (Restek, RTX-1,30 m × 0,25 mm), nitrogen gas as mobile phase and flame ionization detector. Silica gel (particle size 0.040-0.063 mm) from Sigma-Aldrich was used as stationary phase for flash column chromatography. NMR analysis was recorded with a Bruker DRX 400 and 500 (at 400 and 500 MHz for protons and 100 and 126 MHz for carbon-13, respectively) using chloroform or dimethylsulfoxide deuterated solvents. The chemical shifts are reported as δ units in parts per million (ppm) relative to the solvent residual peak as internal reference. High resolution mass spectra were obtained with a Bruker Daltonics micrOTOF QII/ESI-TOF. Microwave irradiation reactions were carried out using a dedicated single-mode microwave reactor (Monowave 300, Anton Paar, Graz, Austria) able to provide 850 W maximum continuous microwave power in combination with an efficient magnetic stirring system. A capillary tube was filled with a sample sufficient to form a column at the bottom of the tube that is between 2.5 and 3.5 mm high. The equipment (Melting Point M-560) was adjusted to reach a base temperature of 30 ºC below the expected melting point. A rate of rise of 2.0 ºC per minute was determined. After the equipment reached the base temperature, the capillary tube was slowly introduced into the heating block so that there was enough time to heat the capillary tube and the "yes" button was on the keyboard to start heating with the rate of rise. After the sample liquefied, the melting point interval values of the sample were recorded.

Synthesis of 4-allyl-2-methoxy-6-nitrophenol (2)

In a 10 mL round bottom flask, CH_2Cl_2 (3.5 mL) was added in 4-allyl-2-methoxyphenol (0.15 mL; 1.0 mmol; 1.0 equiv.) at 25°C, and then H_2O (0.2 mL) and SiO_2 (0.2 g) were added. After this, $KHSO_4$ (0.4903 g, 3.6 mmol, 3.6 equiv.) and $NaNO_3$ (0.4300 g, 4.0 mmol; 4.0 equiv.) were added. The reaction mixture remained under magnetic stirring at 25°C for 1 hour and 30 minutes, and was monitored using thin layer chromatography. After the end of the reaction, the mixture was filtrated and then it was washed with distilled

water (10 mL), extracted with ethyl acetate (3 times with 10 mL each) and washed with concentrated NaCl solution (10 mL). The organic phase was dried over $MgSO_4$, filtered and evaporated under vacuum rotation. The crude material was purified by column chromatography (Hexanes/Ethyl Acetate 90:10) to give the product as orange solid (1779 mg, 85% yield). [2]

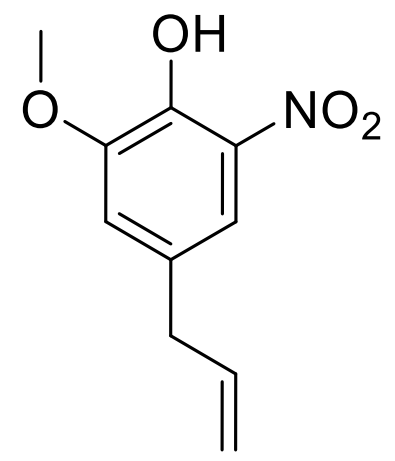

^{1}H NMR (400 MHz, $CDCl_3$) δ 10.67 (s, 1H), 7.50 (d, *J* = 1.8 Hz, 1H), 6.96 (d, *J* = 1.7 Hz, 1H), 6.07 – 5.77 (m, 1H), 5.21 – 5.03 (m, 2H), 3.93 (s, 3H), 3.35 (d, *J* = 6.6 Hz, 2H); ^{13}C NMR (101 MHz, $CDCl_3$) δ 150.3, 145.4, 136.4, 134.1, 131.7, 119.1, 117.6, 115.6, 57.2, 39.9.

Synthesis of 4-allyl-2-azido-6-methoxyphenol (3)

In a 50 mL round-bottom flask, 4-allyl-2-methoxy-6-nitrophenol (1.2078 g; 5.77 mmol; 1.0 equiv.) was added, and then a mixture of AcOH and H_2O (5.77 mL and 8.66 mL respectively) keeping in magnetic stirring at 25°C. Then Zn° (1.592 g; 23.08 mmol; 4.0 equiv.) was added, and the reaction mixture was kept under magnetic stirring until 1 hour. After this, $NaNO_2$ (0.4379 g; 6.35 mmol; 1.1 equiv. in 5.77 mL of distilled water) was added, kept under magnetic stirring at 0° C for 15 minutes. And then NaN_3 (0.5627 g; 8.66 mmol in 6.92 mL of distilled water) was added and kept under stirring at 0° C for 5 minutes and at 25°C for 30 minutes. This reaction was monitored by thin layer chromatography. The mixture was washed with distilled water (10 mL), extracted with ethyl acetate (3 times of 10 mL each), and washed with concentrated NaCl solution (twice with 10 mL each). The organic phase was dried over $MgSO_4$, filtered and evaporated under vacuum rotation. [3]

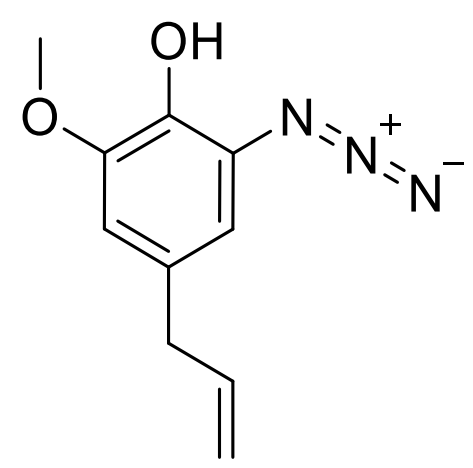

The crude material was purified by column chromatography (Hexanes/Ethyl Acetate 80:20) to give the product as brown oil (686 mg, 92% yield). ^{1}H NMR (400 MHz, $CDCl_3$) δ 6.50 (s, 2H), 5.98 - 5.87 (m, 1H), 5.51 (s, 1H), 5.10 (m, 1H), 5.07 (m, 1H), 3.88 (s, 3H), 3.30 (d, J = 6.7 Hz, 2H), ^{13}C NMR (101 MHz, $CDCl_3$) δ 147.1, 136.9, 135.8, 132.0, 125.6, 116.0, 112.0, 107.8, 56.1, 39.7.

Synthesis of 4-allyl-2-methoxy-6-(4-(phenoxymethyl)-*1H*-1,2,3-triazol-1-yl)phenol (4j)

In a 10 mL round bottom flask, (prop-2-yn-1-yloxy)benzene (0.83 equiv.; 0.37 mmol; 0.0489g) was solubilized in *t*-BuOH (1.0 mL). Then, in this order, 4-allyl-2-azido-6-methoxyphenol (1.2 equiv.; 0.45 mmol; 0.0902g), distilled water (1.0 mL), $CuSO_4{\cdot}5H_2O$ (0.1 equiv.; 0.04 mmol; 0.0114 g) and sodium *L*-ascorbate (0.2 equiv.; 0.08 mmol, 0.0160 g) were added in the reaction mixture. The reaction was maintained under magnetic stirring at 25°C and monitored by thin layer chromatography. After 2 hours and 30 minutes, the crude product was washed with distilled water (10 mL) and extracted with ethyl acetate (twice with 10 mL each). [4]

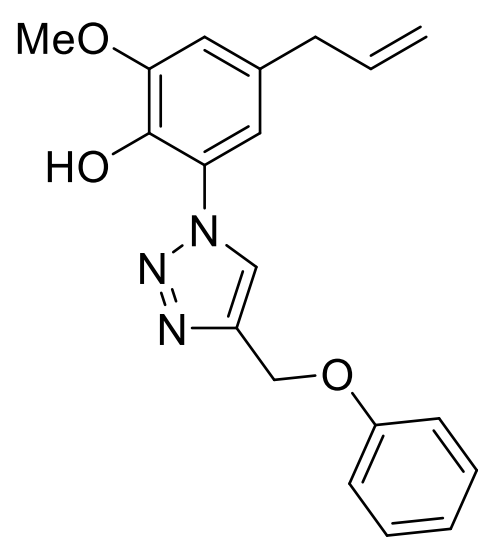

4-allyl-2-methoxy-6-(4-(phenoxymethyl)-1H-1,2,3-triazol-1-yl)phenol ($C_{19}H_{19}N_3O_3$) (4j). The crude material was purified by column chromatography (Hexanes/Ethyl Acetate 80:20) to give the product as brown gel (61 mg, 40% yield). ^{1}H NMR (400 MHz, $CDCl_3$) δ: 8.39 (s, 1H), 7.55 (s, 1H), 7.42 - 7.36 (m, 2H), 7.26 (s, 1H), 7.13 - 7.09 (m, 2H), 7.09 - 7.04 (m, 1H), 6.84 (d, J = 1.5 Hz, 1H), 6.09 - 5.98 (m, 1H), 5.38 (s, 2H), 5.23 - 5.20 (m, 1H), 5.18 (t, J = 1.3 Hz, 1H), 4.02 (s, 3H), 3.46 (d, J = 6.6 Hz, 2H). ^{13}C NMR (101 MHz, $CDCl_3$) δ 158.4, 148.4, 144.1, 136.8, 136.6, 132.3, 129.7, 123.4, 123.3, 121.4, 116.6, 115.0, 114.4, 111.4, 62.1, 56.6, 39.8. HRMS (ESI/Q-TOF) *m/z* Calcd for $[C_{19}H_{20}N_3O_3]^+$: 338.1499; Found: 338.1518 $[M+H]^+$ (error: 5.6188 ppm). Purity (HPLC): 95.5 % (R_t = 13.4 min).

General Procedure for the Synthesis of Compounds 4a-i, 4k-t, 5

In a microwaving sealed tube, 4-allyl-2-azido-6-methoxyphenol (1.0 equiv.), $CuSO_4 \cdot 5H_2O$ (0.01 equiv.), Sodium *L*-ascorbate (0.1 equiv.), propargyl derivative (1.1 equiv.), tert-butanol (1 mL) and distilled water (1 mL) were added. The reaction was kept under stirring in a microwave reactor at 125 °C and 300 W. After 10 minutes, the crude product was washed with distilled water (5 mL) and with saturated NaCl solution (10 mL) and extracted with ethyl acetate (3 times with 10 mL each). Column chromatography was performed with the crude product, obtaining a pure compound.[5]

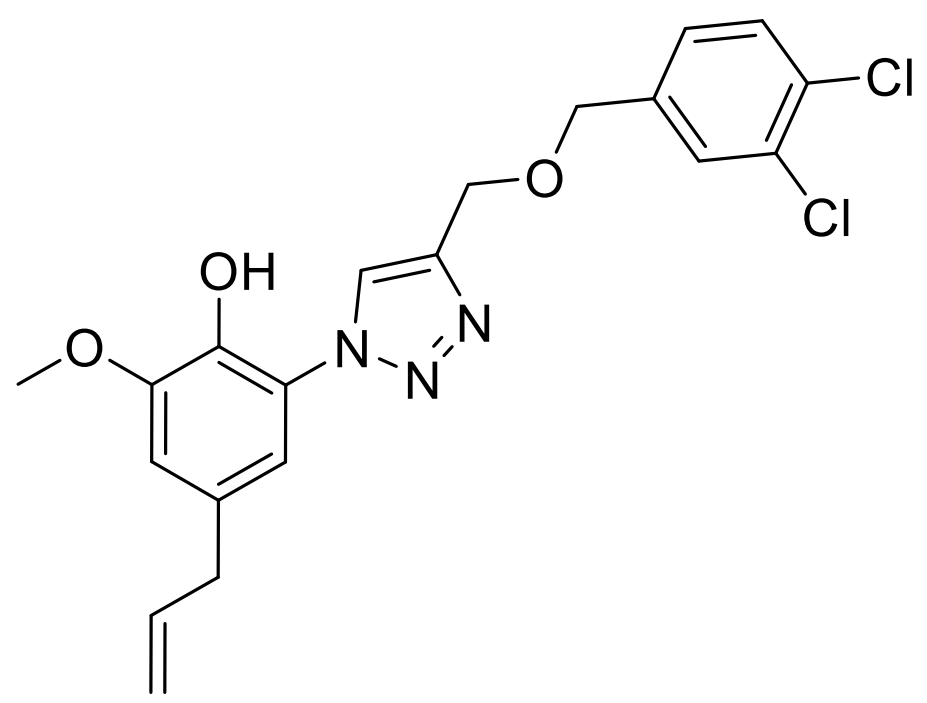

4-allyl-2-(4-(((3,4-dichlorobenzyl)oxy)methyl)-1H-1,2,3-triazol-1-yl)-6-methoxyphenol ($C_{20}H_{19}Cl_2N_3O_3$) (4a). The crude material was purified by column chromatography (Hexanes/Ethyl Acetate 60:40) to give the product as orange solid (111 mg, 84% yield). M.p. 82.4 – 84.0 ºC. ^{1}H NMR (400 MHz, $CDCl_3$) δ 8.25 (s, 1H), 7.46 (d, *J* = 1.9 Hz, 1H), 7.39 (d, *J* = 8.2 Hz, 1H), 7.22 – 7.15 (m, 2H), 6.75 (d, *J* = 1.7 Hz, 1H), 5.94 (m, 1H), 5.12 (ddd, *J* = 4.1, 3.1, 1.6 Hz, 1H), 5.09 (t, *J* = 1.3 Hz, 1H), 4.77 (s, 2H), 4.57 (s, 2H), 3.93 (s, 3H), 3.37 (d, *J* = 6.7 Hz, 2H). ^{13}C NMR (101 MHz, $CDCl_3$) δ 148.4, 144.4, 138.2, 136.8, 136.5, 132.6, 132.3, 131.7, 130.5, 129.7, 127.1, 123.4, 123.3, 116.7, 114.4, 111.4, 71.0, 63.8, 56.5, 39.8. HRMS (ESI/Q-TOF) *m/z* Calcd for $[C_{20}H_{20}Cl_2N_3O_3]^+$: 420.0876; Found: 420.0834 $[M + H]^+$ (error: -9.9979 ppm). Purity (HPLC): 98.0 % (R_t = 15.9 min).

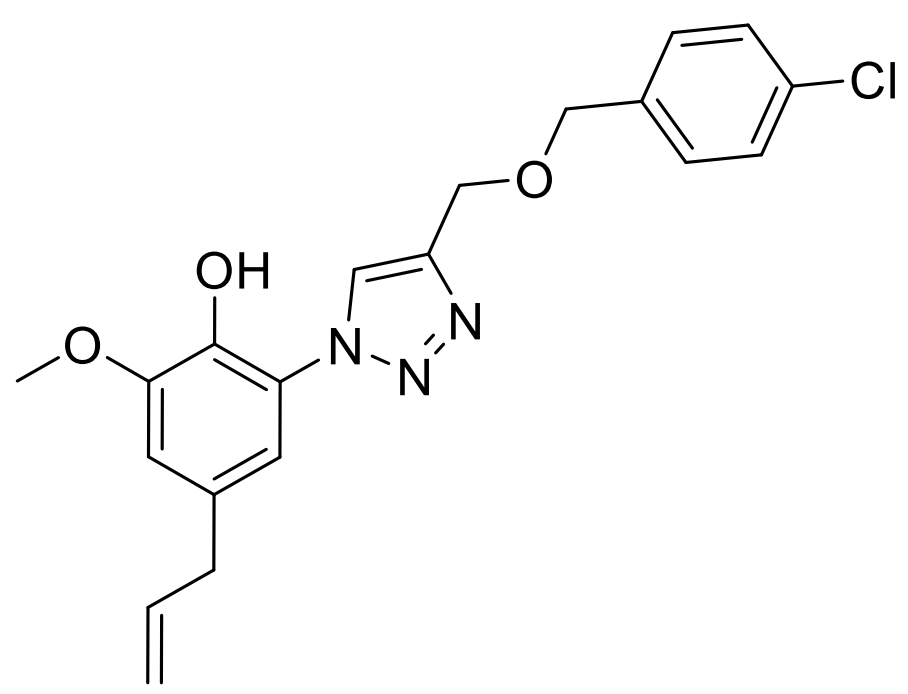

4-allyl-2-(4-(((4-chlorobenzyl)oxy)methyl)-1H-1,2,3-triazol-1-yl)-6-methoxyphenol ($C_{20}H_{20}ClN_3O_3$) (4b). The crude material was purified by column chromatography (Hexanes/Ethyl Acetate 60:40) to give the product as yellow solid (98 mg, 88% yield). M.p. 83.2 – 84.6 ºC. ^{1}H NMR (400 MHz, $CDCl_3$) δ 8.24 (s, 1H), 7.30 (s, 4H), 7.15 (d, J = 1.8 Hz, 1H), 6.75 (d, J = 1.8 Hz, 1H), 5.99 – 5.89 (m, 1H), 5.14 – 5.11 (m, 1H), 5.09 (t, J = 1.4 Hz, 1H), 4.76 (s, 2H), 4.60 (s, 2H), 3.93 (s, 3H), 3.37 (d, J = 6.7 Hz, 2H). ^{13}C NMR (101 MHz, $CDCl_3$) δ 148.4, 144.6, 136.8, 136.5, 136.3, 133.7, 132.3, 129.4, 128.7, 123.4, 123.2, 116.7, 114.3, 111.4, 71.8, 63.6, 56.5, 39.8. HRMS (ESI/Q-TOF) m/z Calcd for $[C_{20}H_{21}ClN_3O_3]^+$: 386.1266; Found: 386.1243 $[M+H]^+$ (error: -5.9566 ppm). Purity (HPLC): 99.1 % (R_t = 14.5 min).

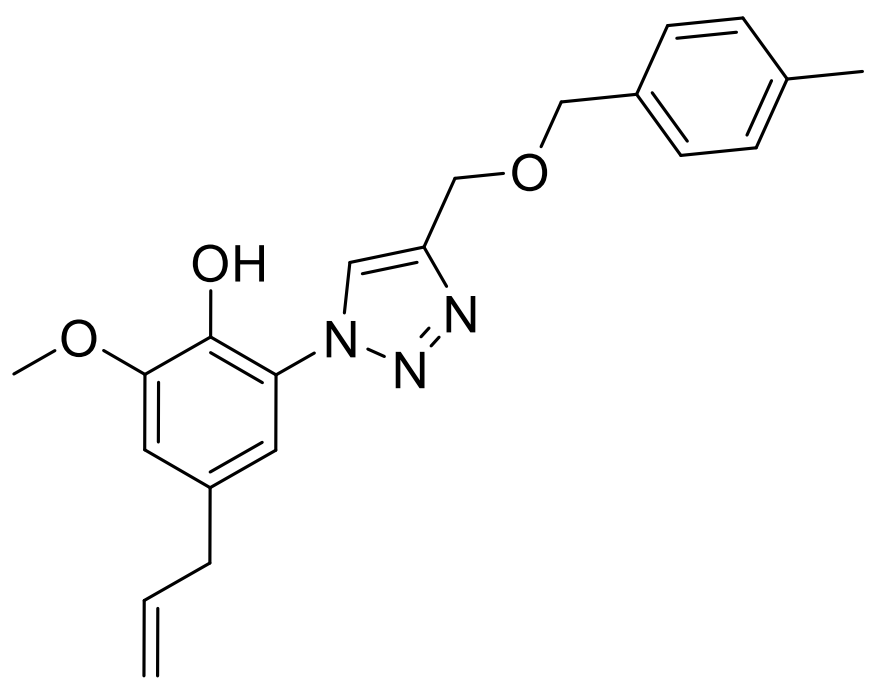

4-allyl-2-methoxy-6-(4-(((4-methylbenzyl)oxy)methyl)-1H-1,2,3-triazol-1-yl)phenol ($C_{21}H_{23}N_3O_3$) (4c). The crude material was purified by column chromatography (Hexanes/Ethyl Acetate 60:40) to give the product as beige solid (89 mg, 80% yield). M.p. 77.5 – 78.3 ºC. ^{1}H NMR (400 MHz, $CDCl_3$) δ 8.20 (s, 1H), 7.25 (d, J = 7.6 Hz, 2H), 7.14 (d, J = 7.8 Hz, 2H), 7.11 (d, J = 1.8 Hz, 1H), 6.73 (d, J = 1.7 Hz, 1H), 5.98 – 5.87 (m, 1H), 5.11 (dq, J = 4.2, 1.7 Hz, 1H), 5.08 (t, J = 1.4 Hz, 1H), 4.74 (s, 2H), 4.59 (s, 2H), 3.92 (s, 3H), 3.92 (s, 3H), 3.35 (d, J = 6.7 Hz, 2H), 2.32 (s, 3H). ^{13}C NMR (101 MHz, $CDCl_3$) δ 148.5, 145.0, 137.7, 136.8, 136.7, 134.7, 132.2, 129.3, 128.2, 123.2, 123.1, 116.7, 114.0, 111.4, 72.6, 63.4, 56.5, 39.8, 21.3. HRMS (ESI/Q-TOF) m/z Calcd for $[C_{21}H_{24}N_3O_3]^+$: 366.1812; Found: 366.1773 $[M+H]^+$ (error: -10.6505 ppm). Purity (HPLC): 100 % (R_t = 13.8 min).

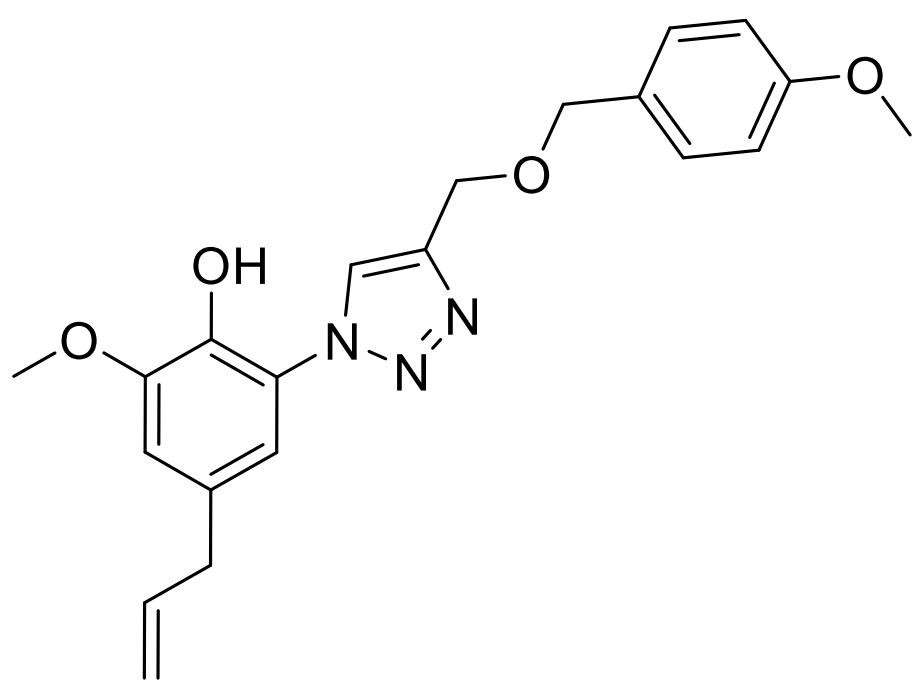

4-allyl-2-methoxy-6-(4-(((4-methoxybenzyl)oxy)methyl)-1H-1,2,3-triazol-1-yl)phenol ($C_{21}H_{23}N_3O_4$) (4d). The crude material was purified by column chromatography (Hexanes/Ethyl Acetate 60:40) to give the product as yellow solid (98 mg, 84% yield). M.p. 71.4 – 72.8 ºC. ^{1}H NMR (400 MHz, $CDCl_3$) δ 8.21 (s, 1H), 7.30 (m, 2H), 7.13 (d, *J* = 1,3Hz, 1H), 6.91 – 6.85 (m, 2H), 6.74 (d, *J* = 1.7 Hz, 1H), 6.00 – 5.88 (m, 1H), 5.12 (dd, *J* = 4.4, 2.8 Hz, 1H), 5.09 (d, *J* = 1.2 Hz, 1H), 4.74 (s, 2H), 4.57 (s, 2H), 3.92 (s, 1H), 3.79 (s, 3H), 3.36 (d, *J* = 6.6 Hz, 2H). ^{13}C NMR (101 MHz, $CDCl_3$) δ 159.4, 148.5, 145.0, 136.8, 136.6, 132.2, 129.8, 129.7, 123.2, 123.1, 116.6, 114.1, 113.9, 111.4, 72.3, 63.2, 56.5, 55.4, 39.8. HRMS (ESI/Q-TOF) *m/z* Calcd for $[C_{21}H_{24}N_3O_4]^+$: 382.1761; Found: 382.1737 $[M+H]^+$ (error: -6.2798 ppm). Purity (HPLC): 99.7 % (R_t = 13.2 min).

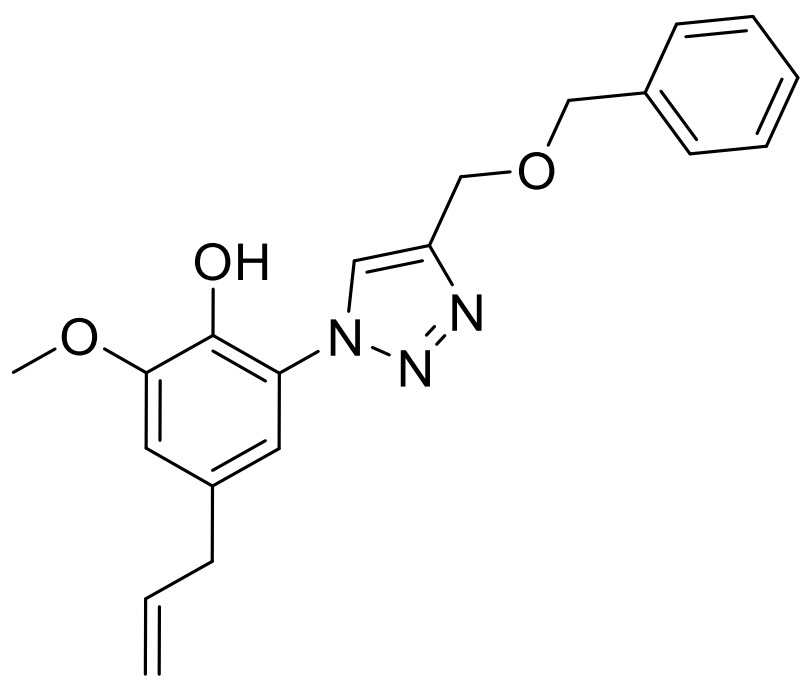

4-allyl-2-(4-((benzyloxy)methyl)-1H-1,2,3-triazol-1-yl)-6-methoxyphenol ($C_{20}H_{21}N_3O_3$) (4e). The crude material was purified by column chromatography (Hexanes/Ethyl Acetate 60:40) to give the product as beige solid (87 mg, 79% yield). M.p. 78.4 – 79.8 ºC. ^{1}H NMR (400 MHz, $CDCl_3$) δ 8.24 (s, 1H), 7.40 – 7.26 (m, 5H), 7.14 (d, *J* = 1.8 Hz, 1H), 6.75 (d, *J* = 1.8 Hz, 1H), 6.01 – 5.88 (m, 1H), 5.13 (dq, *J* = 4.2, 1.6 Hz, 1H), 5.10 (t, *J* = 1.4 Hz, 1H), 4.78 (s, 2H), 4.65 (s, 2H), 3.93 (s, 3H), 3.37 (d, *J* = 6.7 Hz, 2H). ^{13}C NMR (101 MHz, $CDCl_3$) δ 148.5, 144.8, 137.7, 136.8, 136.6, 132.2, 128.6, 128.1, 128.0, 123.3, 123.2, 116.7, 114.2, 111.5, 72.8, 63.5, 56.5, 39.8. HRMS (ESI/Q-TOF) *m/z* Calcd for $[C_{20}H_{22}N_3O_3]^+$: 352.1656; Found: 352.1631 $[M+H]^+$ (error: -7.0989 ppm). Purity (HPLC): 97.7 % (R_t = 13.1 min).

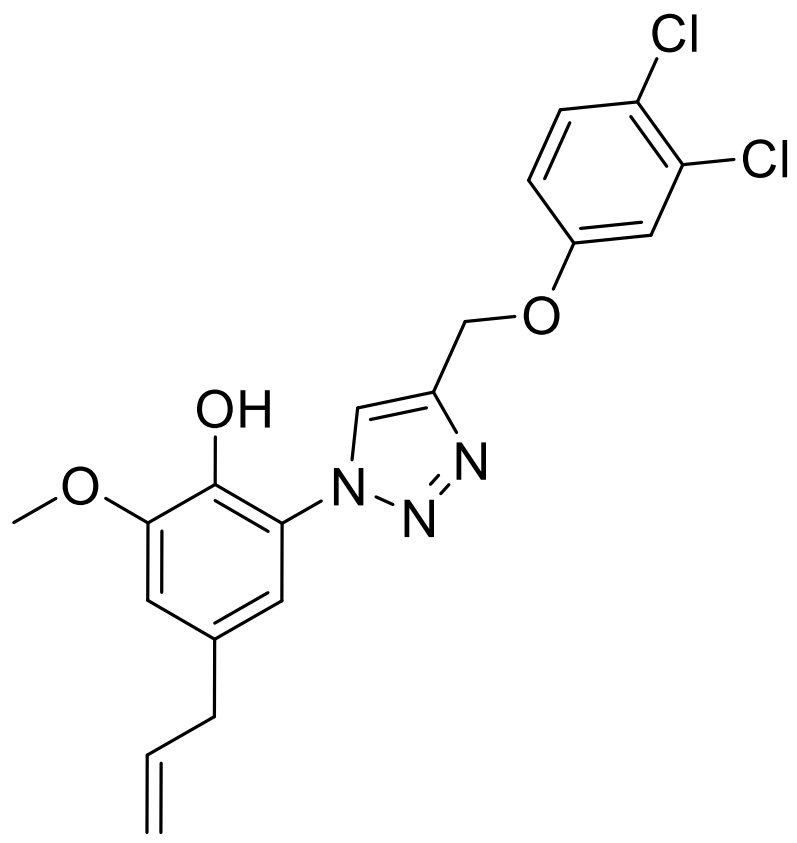

4-allyl-2-(4-((3,4-dichlorophenoxy)methyl)-1H-1,2,3-triazol-1-yl)-6-methoxyphenol ($C_{19}H_{17}Cl_2N_3O_3$) (4f). The crude material was purified by column chromatography (Hexanes/Ethyl Acetate 60:40) to give the product as white solid (114 mg, 93% yield). M.p. 129.8 – 130.8 ºC. ^{1}H NMR (400 MHz, $CDCl_3$) δ 8.32 (s, 1H), 7.33 (d, *J* = 8.8 Hz, 1H), 7.21 (s, 1H), 7.13 (d, *J* = 2.3 Hz, 1H), 6.89 (dd, *J* = 8.8, 2.3 Hz, 1H), 6.76 (s, 1H), 5.95 (ddt, J = 17.2, 10.4, 6.6 Hz, 1H), 5.26 (s, 2H), 5.16 – 5.11 (m, 1H), 5.10 (s, 1H), 3.95 (s, 3H), 3.38 (d, *J* = 6.6 Hz, 2H). ^{13}C NMR (101 MHz, $CDCl_3$) δ 157.3, 148.2, 143.0, 136.8, 136.3, 133.1, 132.4, 130.9, 124.7, 123.8, 123.2, 117.1, 116.7, 114.8, 114.6, 111.4, 62.5, 56.6, 39.9. HRMS (ESI/Q-TOF) *m/z* Calcd for $[C_{19}H_{18}Cl_2N_3O_3]^+$: 406.0720; Found: 406.0690 $[M+H]^+$ (error: -7.3878 ppm). Purity (HPLC): 100 % (R_t = 15.8 min).

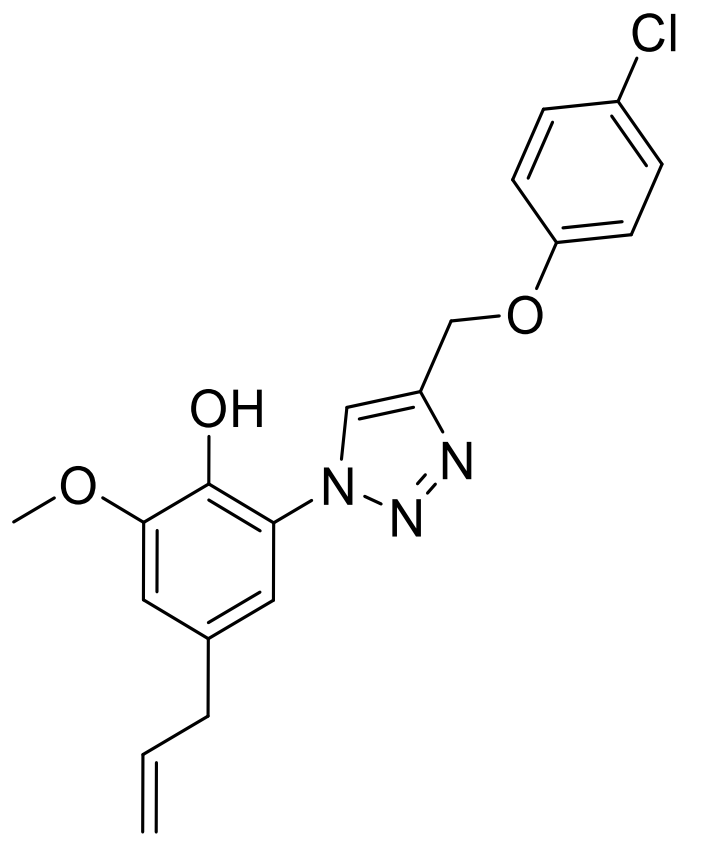

4-allyl-2-(4-((4-chlorophenoxy)methyl)-1H-1,2,3-triazol-1-yl)-6-methoxyphenol ($C_{19}H_{18}ClN_3O_3$) (4g). The crude material was purified by column chromatography (Hexanes/Ethyl Acetate 60:40) to give the product as orange solid (101,6 mg, 87% yield). M.p. 101.5 – 102.7 ºC. ^{1}H NMR (400 MHz, $CDCl_3$) δ 8.32 (s, 1H), 7.26 – 7.22 (m, 2H), 7.19 (d, *J* = 1.9 Hz, 1H), 6.97 – 6.92 (m, 2H), 6.76 (d, *J* = 1.8 Hz, 1H), 6.03 – 5.87 (m, 1H), 5.27

(s, 1H), 5.13 (dq, J = 4.2, 1.6 Hz, 1H), 5.09 (t, J = 1.4 Hz, 1H), 3.94 (s, 3H), 3.37 (d, J = 6.7 Hz, 2H). ^{13}C NMR (101 MHz, $CDCl_3$) δ 156.9, 148.3, 143.4, 136.8, 136.4, 132.4, 129.6, 126.4, 123.8, 123.2, 116.7, 116.3, 114.5, 111.5, 62.2, 56.6, 39.8. HRMS (ESI/Q-TOF) *m/z* Calcd for $[C_{19}H_{19}ClN_3O_3]^+$: 372.1109; Found: 372.1111 $[M+H]^+$ (error: 0.5375 ppm). Purity (HPLC): 100 % (R_t = 14.6 min).

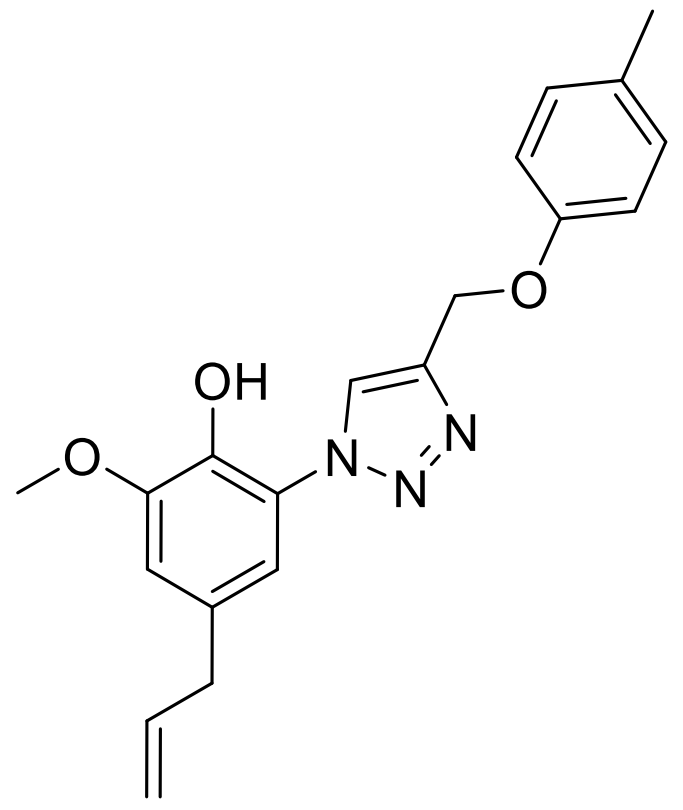

4-allyl-2-methoxy-6-(4-((p-tolyloxy)methyl)-1H-1,2,3-triazol-1-yl)phenol ($C_{20}H_{21}N_3O_3$) (4h). The crude material was purified by column chromatography (Hexanes/Ethyl Acetate 60:40) to give the product as beige solid (88 mg, 83% yield). M.p. 94.9 – 95.6 ºC. ^1H NMR (400 MHz, $CDCl_3$) δ 8.30 (s, 1H), 7.16 (d, J = 1.8 Hz, 1H), 7.12 – 7.07 (m, 2H), 6.94 – 6.89 (m, 2H), 6.75 (d, J = 1.8 Hz, 1H), 6.00 – 5.89 (m, 1H), 5.27 (s, 1H), 5.13 (dq, J = 4.2, 1.6 Hz, 1H), 5.10 (t, J = 1.4 Hz, 1H), 3.93 (s, 3H), 3.37 (d, J = 6.7 Hz, 2H), 2.28 (s, 3H). ^{13}C NMR (101 MHz, $CDCl_3$) δ 156.1, 148.4, 144.0, 136.8, 136.6, 132.3, 130.7, 130.1, 123.5, 123.2, 116.7, 114.7, 114.3, 111.5, 62.0, 56.5, 39.8, 20.6. HRMS (ESI/Q-TOF) *m/z* Calcd for $[C_{20}H_{22}N_3O_3]^+$: 352.1656; Found: 352.1639 $[M+H]^+$ (error: -4.8273 ppm). Purity (HPLC): 100 % (R_t = 14.1 min).

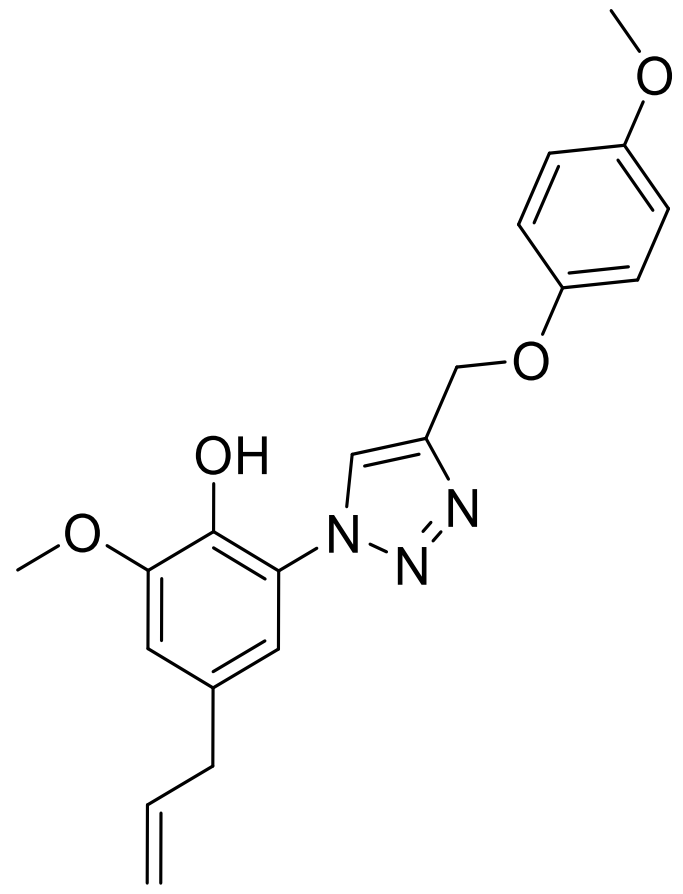

4-allyl-2-methoxy-6-(4-((4-methoxyphenoxy)methyl)-1H-1,2,3-triazol-1-yl)phenol ($C_{20}H_{21}N_3O_4$) (4i). The crude material was purified by column chromatography (Hexanes/Ethyl Acetate 60:40) to give the product as beige solid (95 mg, 86% yield). M.p. 72.2 – 73.5 ºC. ^{1}H NMR (400 MHz, $CDCl_3$) δ 8.29 (s, 1H), 7.16 (d, *J* = 1.7 Hz, 1H), 6.98 – 6.93 (m, 2H), 6.87 – 6.81 (m, 2H), 6.75 (d, *J* = 1.7 Hz, 1H), 6.00 – 5.88 (m, 1H), 5.24 (s, 2H), 5.14 – 5.11 (m, 1H), 5.09 (t, *J* = 1.3 Hz, 1H), 3.93 (s, 3H), 3.76 (s, 3H), 3.37 (d, *J* = 6.7 Hz, 2H). ^{13}C NMR (101 MHz, $CDCl_3$) δ 154.3, 152.4, 148.4, 144.1, 136.8, 136.5, 132.3, 123.4, 123.2, 116.7, 116.0, 114.8, 114.3, 111.4, 62.7, 56.5, 55.8, 39.8. HRMS (ESI/Q-TOF) *m/z* Calcd for $[C_{20}H_{22}N_3O_4]^+$: 368.1605; Found: 368.1573 $[M+H]^+$ (error: -8.6919 ppm). Purity (HPLC): 100 % (R_t = 13.2 min).

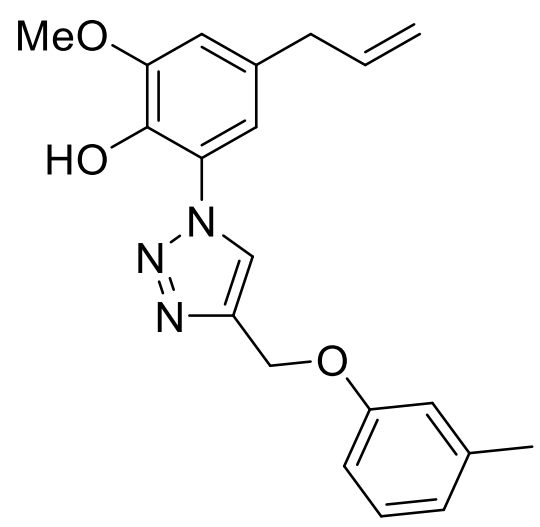

4-allyl-2-methoxy-6-(4-((m-tolyloxy)methyl)-1H-1,2,3-triazol-1-yl)phenol ($C_{20}H_{21}N_3O_3$) (4k). The crude material was purified by column chromatography (Hexanes/Ethyl Acetate 60:40) to give the product as brown solid (197 mg, 98% yield). M.p. 90.2--91.4 °C. ^{1}H NMR (400 MHz, $CDCl_3$) δ: 8.29 (s, 1H), 7.52 (s, 1H), 7.20 (dd, *J* = 7.7, 0.6 Hz, 1H), 7.17 – 7.16 (m, 1H), 6.86 – 6.78 (m, 3H), 6.75 (d, *J* = 1.8 Hz, 1H), 6.00 – 5.90 (m, 1H), 5.28 (s, 2H), 5.15 – 5.12 (m, 1H), 5.10 (t, *J* = 1.4 Hz, 1H), 3.94 (s, 3H), 3.38 (d, *J* = 6.7 Hz, 2H), 2.34 (s, 3H). ^{13}C NMR (101 MHz, $CDCl_3$) δ: 158.4, 148.5, 144.2, 139.8, 136.8, 136.6, 132.3, 129.4, 123.3, 123.3, 122.3, 116.7, 115.8, 114.3, 111.8, 111.4, 62.1, 56.6, 39.8, 21.6. HRMS (ESI/Q-TOF) *m/z* Calcd for $[C_{20}H_{22}N_3O_3]^+$: 352.1656; Found: 352.1648 $[M+H]^+$ (error: -2.2717 ppm). Purity (HPLC): 100 % (R_t = 14.3 min).

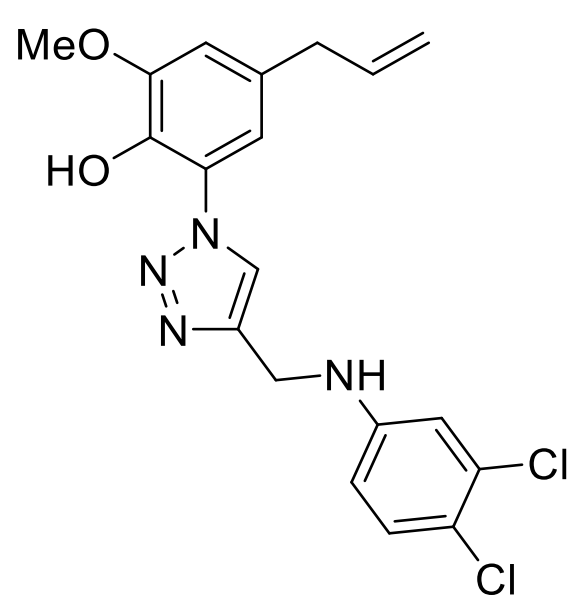

4-allyl-2-(4-((3,4-dichlorophenyl)amino)methyl)-1H-1,2,3-triazol-1-yl)-6-methoxyphenol ($C_{19}H_{18}Cl_2N_4O_2$) (4l). The crude material was purified by column chromatography (Hexanes/Ethyl Acetate 60:40) to give the product as white solid (115 mg, 93% yield).

M.p. 107.5-110.3 °C. ^{1}H NMR (400 MHz, $CDCl_3$) δ: 8.13 (s, 1H), 7.31 (s, 1H), 7.18 (d, J = 8.7 Hz, 1H), 7.15 (d, J = 1.8 Hz, 1H), 6.76 - 6.74 (m, 2H), 6.51 (dd, J = 8.7, 2.8 Hz, 1H), 5.99 - 5.88 (m, 1H), 5.12 (dq, J = 4.1, 1.6 Hz, 1H), 5.09 (t, J = 1.3 Hz, 1H), 4.47 (s, 2H) , 3.93 (s, 3H), 3.36 (d, J = 6.7 Hz, 2H) .^{13}C NMR (101 MHz, $CDCl_3$) δ 148.3, 147.2, 144.9, 136.8, 136.4, 133.0, 132.4, 130.8, 123.4, 122.4, 120.7, 116.7, 114.6, 114.4, 113.0, 111.4, 56.6, 39.8. HRMS (ESI/Q-TOF) *m/z* Calcd for $[C_{19}H_{19}Cl_2N_4O_2]^+$: 405.0880; Found: 405.0873 $[M+H]^+$ (error: -1.7280 ppm). Purity (HPLC): 99.7 % (R_t = 14.7 min).

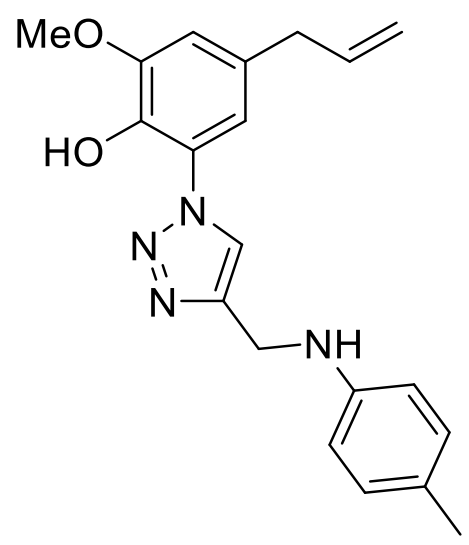

4-allyl-2-methoxy-6- (4 - ((p-tolylamino) methyl) -1H-1,2,3-triazol-1-yl) phenol ($C_{20}H_{22}N_4O_2$) (4m). The crude material was purified by column chromatography (Hexanes/Ethyl Acetate 60:40) to give the product as green solid (143 mg, 99% yield). M.p. 123.3-125.0 °C. ^{1}H NMR (500 MHz, $CDCl_3$) δ: 8.10 (s, 1H), 7.96 - 7.50 (m, 1H), 7.09 (s, 1H), 7.00 (d, J = 8.2 Hz, 2H), 6.73 (d, J = 1.2 Hz, 1H), 6.62 (d, J = 8.3 Hz, 2H), 5.94 (m, 1H), 5.14 - 5.10 (m, 1H), 5.09 (s, 1H), 4.51 (s, 2H), 4.20 (m, 1H), 3.93 (s, 3H), 3.36 (d, J = 6.7 Hz, 2H), 2.24 (s, 3H) .^{13}C NMR (126 MHz, $CDCl_3$) δ 148.6, 146.3, 145.5, 136.9, 136.7, 132.2, 129.9, 127.5, 123.5, 122.1, 116.6, 114.3, 113.6, 111.5, 56.6, 40.5, 39.8, 20.5. HRMS (ESI/Q-TOF) *m/z* Calcd for $[C_{20}H_{23}N_4O_2]^+$: 351.1816; Found: 351.1792 $[M+H]^+$ (error: -6.8341 ppm). Purity (HPLC): 100 % (R_t = 12.9 min).

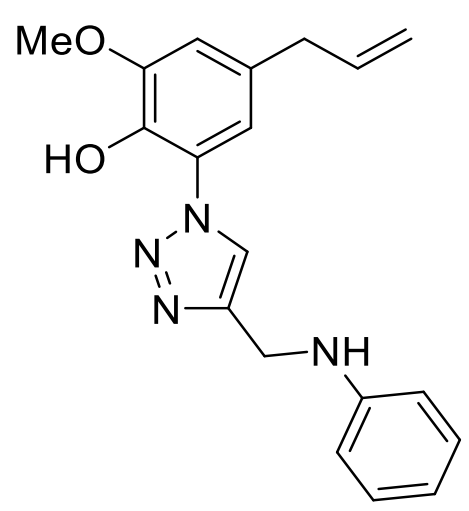

4-allyl-2-methoxy-6- (4- ((phenylamino) methyl) -1H-1,2,3-triazol-1-yl) phenol ($C_{19}H_{20}N_4O_2$) (4n). The crude material was purified by column chromatography (Hexanes/Ethyl Acetate 60:40) to give the product as beige solid (157 mg, 76% yield). M.p. 109.3-112.5 °C. ^{1}H NMR (400 MHz, $CDCl_3$) δ: 8.11 (s, 1H), 7.67 (s, 1H), 7.19 (t, J =

7.8 Hz, 2H), 7.11 (s, 1H), 6.79 - 6.67 (m, 4H), 5.94 (ddt, J = 16.2, 10.7, 6.7 Hz, 1H), 5.15 – 5.11 (m, 1H), 5.09 (s, 1H), 4.54 (s, 2H), 4.30 (s, 1H), 3.93 (s, 3H), 3.36 (d, J = 6.5 Hz, 2H) .^{13}C NMR (101 MHz, $CDCl_3$) δ 148.5, 147.7, 146.1, 136.8, 136.6, 132.2, 129.4, 123.4, 122.1, 118.2, 116.6, 114.2, 113.3, 111.4, 56.5, 40.1, 39.9. HRMS (ESI/Q-TOF) *m/z* Calcd for $[C_{19}H_{21}N_4O_2]^+$: 337.1659; Found: 337.1647 $[M+H]^+$ (error: -3.5591 ppm). Purity (HPLC): 100 % (R_t = 12.5 min).

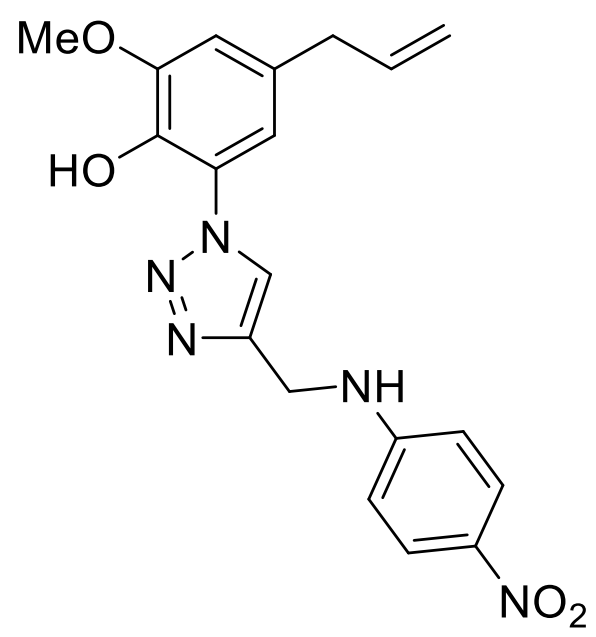

4-allyl-2-methoxy-6-(4-(((4-nitrophenyl)amino)methyl)-1H-1,2,3-triazol-1-yl)phenol ($C_{19}H_{19}N_5O_4$) (4o). The crude material was purified by column chromatography (Hexanes/Ethyl Acetate 60:40) to give the product as yellow solid (95 mg, 57% yield). M.p. 150.6—152.6 °C. ^{1}H NMR (500 MHz, DMSO) δ: 9.60 (s, 1H), 8.41 (s, 1H), 8.00 (d, *J* = 9.2 Hz, 2H), 7.79 (t, *J* = 5.7 Hz, 1H), 7.02 (s, 1H), 6.94 (d, *J* = 1.3 Hz, 1H), 6.79 (d, *J* = 9.2 Hz, 2H), 5.96 (ddt, *J* = 16.8, 10.0, 6.8 Hz, 1H), 5.11 (d, *J* = 17.0 Hz, 1H), 5.06 (d, *J* = 10.0 Hz, 1H), 4.54 (d, *J* = 5.7 Hz, 2H), 3.87 (s, 3H), 3.38 – 3.33 (m, 5H). ^{13}C NMR (126 MHz, DMSO) δ: 154.1, 148.6, 143.7, 137.4, 137.2, 136.1, 130.9, 126.0, 124.6, 124.5, 116.0, 115.9, 112.3, 111.2, 56.2, 38.8, 37.7. HRMS (ESI/Q-TOF) *m/z* Calcd for $[C_{19}H_{20}N_5O_4]^+$: 382.1510; Found: 382.1479 $[M+H]^+$ (error: -8.1120 ppm). Purity (HPLC): 100 % (R_t = 13.1 min).

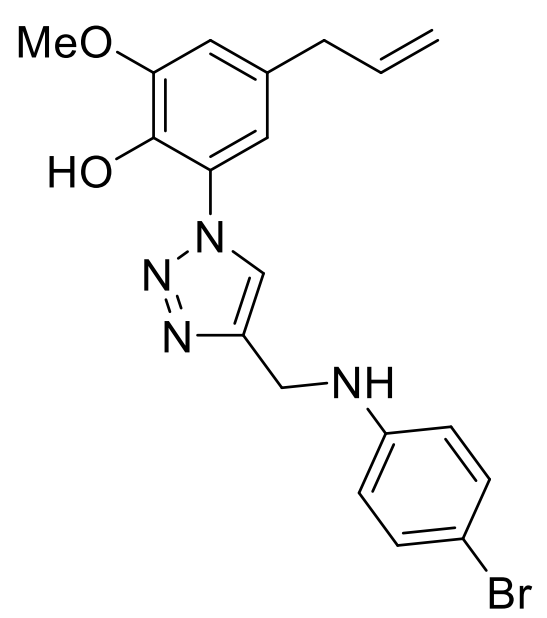

4-allyl-2-(4-(((4-bromophenyl)amino)methyl)-1H-1,2,3-triazol-1-yl)-6-methoxyphenol ($C_{19}H_{19}BrN_4O_2$) (4p). The crude material was purified by column chromatography (Hexanes/Ethyl Acetate 60:40) to give the product as brown solid (107 mg, 93% yield). M.p. 160.5--163.4 °C. ^{1}H NMR (400 MHz, DMSO) δ: 9.58 (s, 1H), 8.32 (s, 1H), 7.22 – 7.17

(m, 2H), 7.03 (d, J = 1.9 Hz, 1H), 6.94 (d, J = 1.9 Hz, 1H), 6.67 – 6.62 (m, 2H), 5.96 (m, 1H), 5.11 (m, 1H), 5.08 – 5.03 (m, 1H), 4.35 (s, 2H), 3.86 (s, 3H), 3.56 (s, 1H), 3.35 (d, J = 6.7 Hz, 2H). ^{13}C NMR (101 MHz, DMSO) δ: 148.6, 147.6, 145.0, 137.4, 137.0, 131.3, 130.9, 124.6, 124.2, 116.0, 115.9, 114.3, 112.2, 106.8, 56.2, 38.8, 38.3. HRMS (ESI/Q-TOF) m/z Calcd for $[C_{19}H_{20}BrN_4O_2]^+$: 415.0764; Found: 415.0728 $[M+H]^+$ (error: -8.6731 ppm). Purity (HPLC): 98.1 % (R_t = 14.4 min).

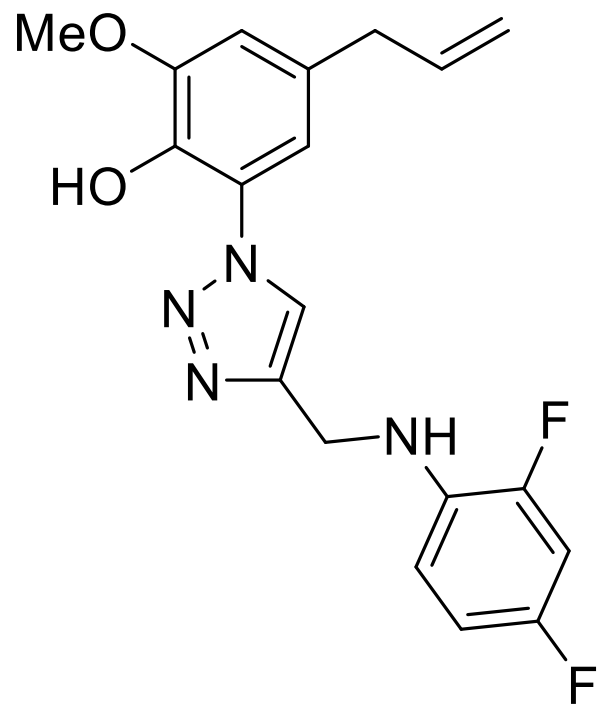

4-allyl-2-(4-(((2,4-difluorophenyl)amino)methyl)-1H-1,2,3-triazol-1-yl)-6-methoxyphenol ($C_{19}H_{18}F_2N_4O_2$) (4q). The crude material was purified by column chromatography (Hexanes/Ethyl Acetate 60:40) to give the product as brown solid (105 mg, 90% yield). M.p. 89.6—92.9 °C. ^{1}H NMR (500 MHz, $CDCl_3$) δ: 8.14 (s, 1H), 7.45 (s, 1H), 7.14 (s, 1H), 6.81 – 6.68 (m, 4H), 5.94 (m, 1H), 5.14 – 5.10 (m, 1H), 5.09 (s, 1H), 4.54 (s, 2H), 4.33 (s, 1H), 3.93 (s, 3H), 3.36 (d, J = 6.7 Hz, 2H). ^{13}C NMR (126 MHz, $CDCl_3$) δ: 153.1 (ddd, J = 253.9, 240.4, 11.2 Hz), 132.7 (dd, J = 11.5, 2.9 Hz), 112.9 (dd, J = 8.8, 4.4 Hz), 110.8 (dd, J = 21.8, 3.8 Hz), 103.7 (dd, J = 26.7, 22.8 Hz). HRMS (ESI/Q-TOF) m/z Calcd for $[C_{19}H_{19}F_2N_4O_2]^+$: 373.1471; Found: 373.1446 $[M+H]^+$ (error: -6.6998 ppm). Purity (HPLC): 95.9 % (R_t = 13.0 min).

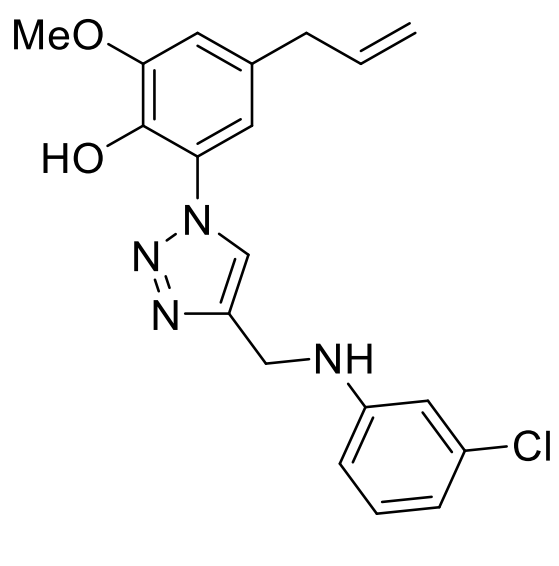

4-allyl-2-(4-(((3-chlorophenyl)amino)methyl)-1H-1,2,3-triazol-1-yl)-6-methoxyphenol ($C_{19}H_{19}ClN_4O_2$) (4r). The crude material was purified by column chromatography (Hexanes/Ethyl Acetate 60:40) to give the product as white solid (200 mg, 89% yield). M.p. 128.3--131.0 °C. ^{1}H NMR (400 MHz, $CDCl_3$) δ: 8.12 (s, 1H), 7.44 (s, 1H), 7.14 (s, 1H), 7.08 (t, J = 8.0 Hz, 1H), 6.74 (d, J = 1.3 Hz, 1H), 6.69 (d, J = 7.9 Hz, 1H), 6.66 (s, 1H), 6.55

(dd, *J* = 8.1, 2.0 Hz, 1H), 5.94 (m, 1H), 5.14 – 5.11 (m, 1H), 5.09 (s, 1H), 4.50 (s, 2H), 4.42 (s, 1H), 3.93 (s, 3H), 3.36 (d, *J* = 6.6 Hz, 2H). ^{13}C NMR (101 MHz, $CDCl_3$) δ 148.8, 148.4, 145.3, 136.8, 136.5, 135.2, 132.3, 130.4, 123.3, 122.3, 118.0, 116.7, 114.5, 113.0, 111.6, 111.4, 56.6, 39.8, 39.8. HRMS (ESI/Q-TOF) *m/z* Calcd for $[C_{19}H_{20}ClN_4O_2]^+$: 371.1269; Found: 371.1237 $[M+H]^+$ (error: -8.6224 ppm). Purity (HPLC): 100 % (R_t = 13.8 min).

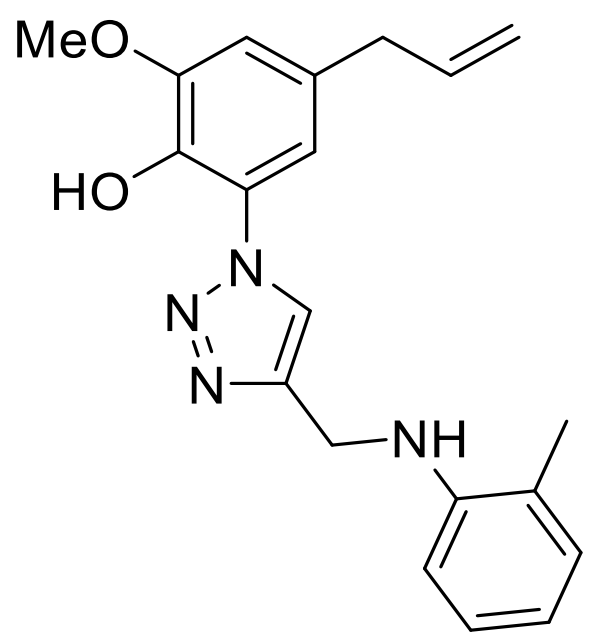

4-allyl-2-methoxy-6-(4-((o-tolylamino)methyl)-1H-1,2,3-triazol-1-yl)phenol ($C_{20}H_{22}N_4O_2$) (4s). The crude material was purified by column chromatography (Hexanes/Ethyl Acetate 60:40) to give the product as brown gel (96 mg, 87% yield). ^1H NMR (400 MHz, $CDCl_3$) δ: 8.13 (s, 1H), 7.84 – 7.50 (m, 1H), 7.16 – 7.06 (m, 3H), 6.75 – 6.68 (m, 3H), 6.00 – 5.89 (m, 1H), 5.13 (dq, *J* = 4.2, 1.6 Hz, 1H), 5.09 (t, *J* = 1.4 Hz, 1H), 4.59 (s, 2H), 4.27 – 4.07 (m, 1H), 3.93 (s, 3H), 3.37 (d, *J* = 6.7 Hz, 2H), 2.19 (s, 3H). ^{13}C NMR (101 MHz, $CDCl_3$) δ: 148.5, 146.1, 145.7, 136.9, 136.6, 132.2, 130.3, 127.3, 123.3, 122.7, 122.1, 117.8, 116.6, 114.2, 111.4, 110.3, 56.5, 40.0, 39.8, 17.6. HRMS (ESI/Q-TOF) *m/z* Calcd for $[C_{20}H_{23}N_4O_2]^+$: 351.1816; Found: 351.1765 $[M+H]^+$ (error: -14.5224 ppm). Purity (HPLC): 98.7 % (R_t = 13.7 min).

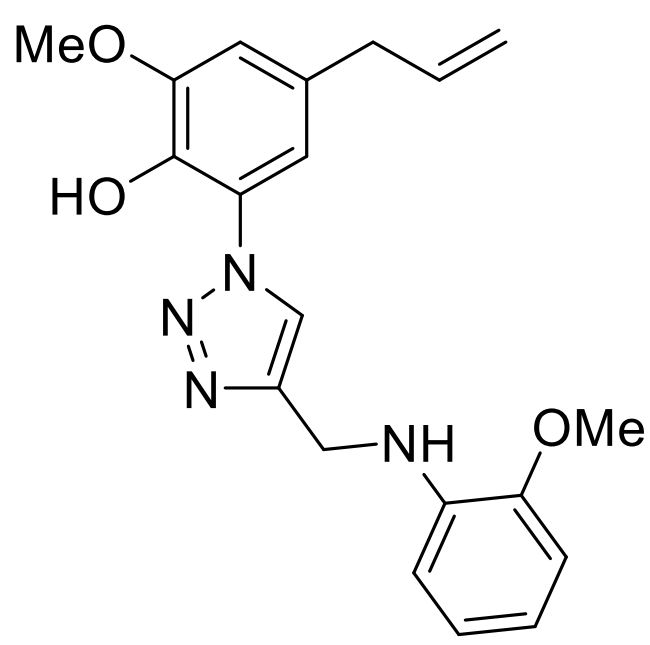

4-allyl-2-methoxy-6-(4-(((2-methoxyphenyl)amino)methyl)-1H-1,2,3-triazol-1-yl)phenol ($C_{20}H_{22}N_4O_3$) (4t). The crude material was purified by column chromatography (Hexanes/Ethyl Acetate 60:40) to give the product as red gel (150 mg, 79% yield). ^1H

NMR (400 MHz, $CDCl_3$) δ: 8.11 (s, 1H), 7.86 (s, 1H), 7.08 (d, *J* = 1.5 Hz, 1H), 6.87 (td, *J* = 7.6, 1.4 Hz, 1H), 6.81 – 6.78 (m, 1H), 6.73 (s, 1H), 6.71 (m, 1H), 6.69 (s, 1H), 5.99 – 5.88 (m, 1H), 5.14 – 5.11 (m, 1H), 5.09 (t, *J* = 1.3 Hz, 1H), 4.97 – 4.71 (m, 1H), 4.57 (s, 2H), 3.93 (s, 3H), 3.85 (s, 3H), 3.36 (d, *J* = 6.7 Hz, 2H). ^{13}C NMR (101 MHz, $CDCl_3$) δ: 148.7, 147.2, 146.5, 137.6, 136.9, 136.7, 132.1, 123.3, 122.0, 121.4, 117.4, 116.6, 113.9, 111.4, 110.5, 109.7, 56.5, 55.5, 39.9, 39.8. HRMS (ESI/Q-TOF) *m/z* Calcd for $[C_{20}H_{23}N_4O_3]^+$: 367.1765; Found: 367.1765 $[M+H]^+$ (error: 0.0000 ppm). Purity (HPLC): 99.6 % (R_t = 13.2 min).

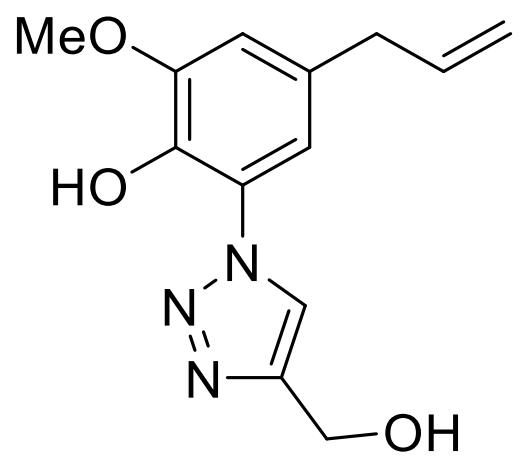

4-allyl-2-(4-(hydroxymethyl)-1H-1,2,3-triazol-1-yl)-6-methoxyphenol ($C_{13}H_{15}N_3O_3$) (5). The crude material was purified by column chromatography (Hexanes/Ethyl Acetate 60:40) to give the product as orange solid (137 mg, 91% yield). M.p. 135.0—137.8 °C. ^1H NMR (400 MHz, DMSO) δ: 9.57 (s, 1H), 8.28 (s, 1H), 7.03 (d, *J* = 1.8 Hz, 1H), 6.94 (d, *J* = 1.8 Hz, 1H), 5.97 (m, 1H), 5.28 (t, *J* = 5.7 Hz, 1H), 5.12 (dd, *J* = 17.0, 1.8 Hz, 1H), 5.06 (dd, *J* = 10.0, 1.8 Hz, 1H), 4.59 (d, *J* = 5.5 Hz, 2H), 3.87 (s, 3H), 3.36 (d, *J* = 8.0 Hz, 4H). ^1H NMR (400 MHz, CD_3OD) δ: 8.32 (s, 1H), 7.05 (d, *J* = 2.0 Hz, 1H), 6.85 (d, *J* = 2.0 Hz, 1H), 5.93 (ddt, *J* = 16.8, 10.0, 6.7 Hz, 1H), 5.09 – 5.00 (m, 2H), 4.71 (s, 2H), 3.86 (s, 3H), 3.32 (d, *J* = 6.8 Hz, 2H). ^{13}C NMR (101 MHz, DMSO) δ: 148.7, 147.7, 137.5, 137.2, 130.9, 124.8, 124.1, 116.0, 116.0, 112.1, 56.2, 55.0, 38.9. HRMS (ESI/Q-TOF) *m/z* Calcd for $[C_{13}H_{16}N_3O_3]^+$: 262.1186; Found: 262.1180 $[M+H]^+$ (error: -2.2890 ppm). Purity (HPLC): 99.7 % (R_t = 8.6 min).

APPENDIX B: Biology

In vitro leishmanicidal assay

After obtaining these eugenol derivatives, samples of each of these compounds were prepared to perform biological activity tests. First, the promastigotes of *Leishmania amazonensis* were cultivated in *Schneider* culture medium, supplemented with 10% fetal calf serum, in increasing concentrations of the test compounds (0-128µM), of the reference drug miltefosine (0,39 -100µM) and dimethyl sulfoxide (0,1 - 1%), used in the dilution of the samples. The culture was started with 1,0 x 10^6 cells per milliliter of suspension and maintained for 72 hours. After this period of time, cell viability was

estimated by the resazurin assay. The 50% inhibitory concentration (IC_{50}) value was determined by logarithmic regression analysis. [6]

To evaluate the same biological effect on intracellular amastigotes of *L. amazonensis*, resident peritoneal macrophages from *Swiss* mice were seeded in RPMI culture medium supplemented with 10% fetal bovine serum, 1% glutamine and 1% pyruvate, containing 1,0 x 10^6 cells per milliliter of suspension in a 24-well plate with coverslips and incubated at 37°C with 5% CO_2 for 1 hour. The resulting monolayers were then washed with pre-warmed complete medium to remove non-adherent cells. Then the stationary phase of *L. amazonensis* promastigotes was added at a ratio of 3:1 or 5:1 parasites/macrophage, and the cultures were incubated for an additional 3 hours. After the incubation period, the chambers were washed again to remove free parasites, then the synthesized compounds were added and the chambers were incubated for 72 hours. The coverslips were then stained using panoptic and then examined under light microscopy. The number of infected cells and intracellular amastigotes was determined by counting at least 100 macrophages per well. The concentration and effect curves were fitted by non-linear regression, using the *GraphPrism 10* program and the IC_{50} values were obtained. [6]

Cytotoxicity screening

Peritoneal macrophages from Swiss mice were plated at a concentration of 2.0 x 10^6 cells/mL in RPMI medium supplemented with 10% fetal bovine serum, 1% glutamine and 1% pyruvate in 96-well plates and incubated at 37 °C in an atmosphere containing 5% CO_2 for 1 h, after which the non-adherent cells were removed by washing with prewarmed medium. The cell monolayers were then incubated for 72 hours with increasing concentrations of the substances (1.56–200 µM). Cell viability was determined by adding 50 µM resazurin per well during the final three hours of incubation. After this time, fluorescence was quantified at 560 and 590 nm (excitation and emission, respectively). The value corresponding to half the maximum cytotoxic concentration (CC_{50}) for each curve was determined by logarithmic non-linear regression using the GraphPad Prism 5.0 programme. The selectivity index (SI) for the molecules was calculated from the ratio between the CC_{50} for macrophages and the IC_{50} for intracellular amastigotes.

APPENDIX C: Medicinal Chemistry

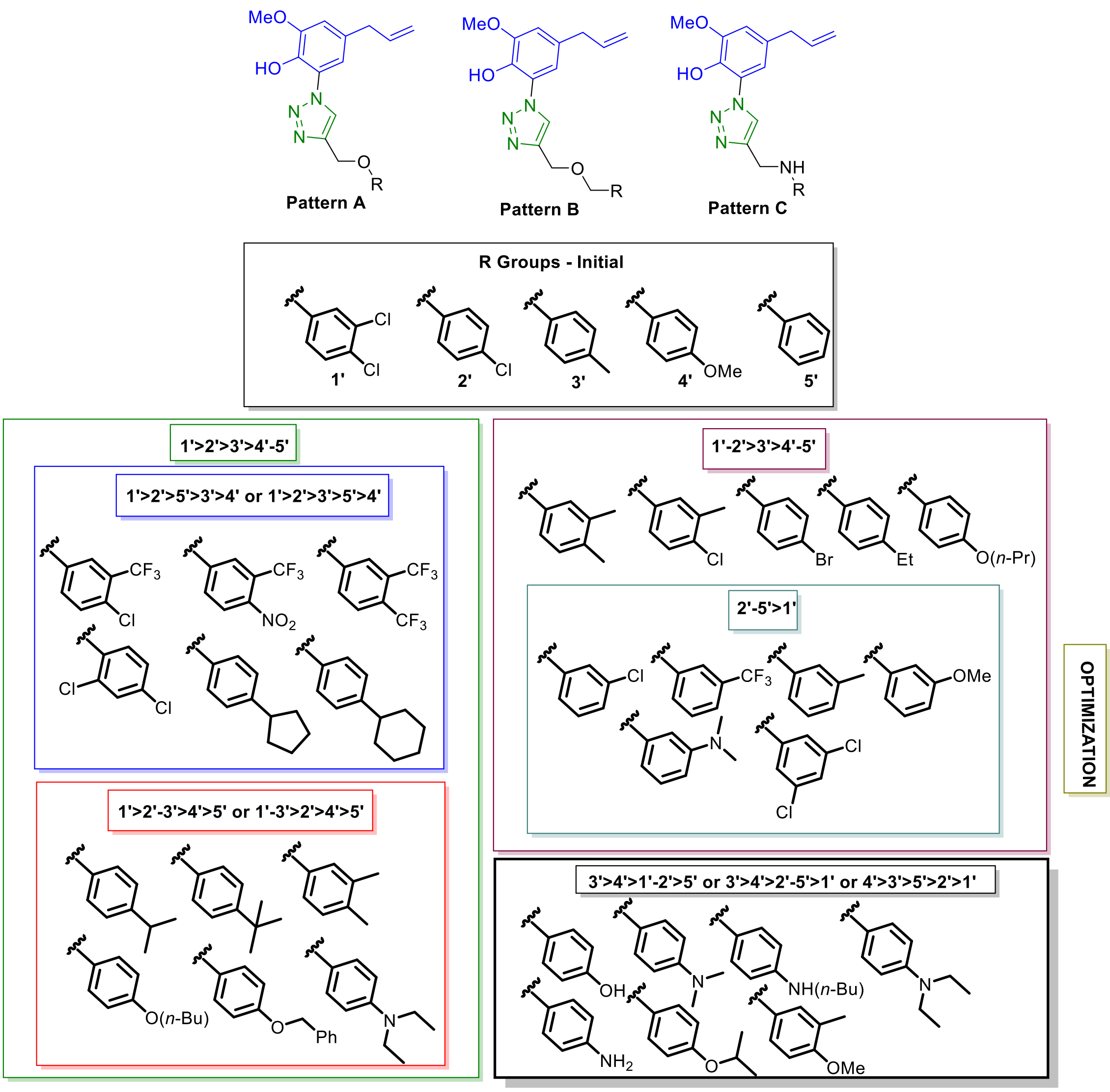

Figure S1. Diagram of *Topliss Batchwise* used for eugenol derivatives.

APPENDIX D: Nuclear Magnetic Resonance Spectra of ^{1}H, ^{13}C, ^{13}C DEPT--135 and ^{1}H/^{13}C HSQC:

^{1}H NMR of 1 (500 MHz, $CDCl_3$)

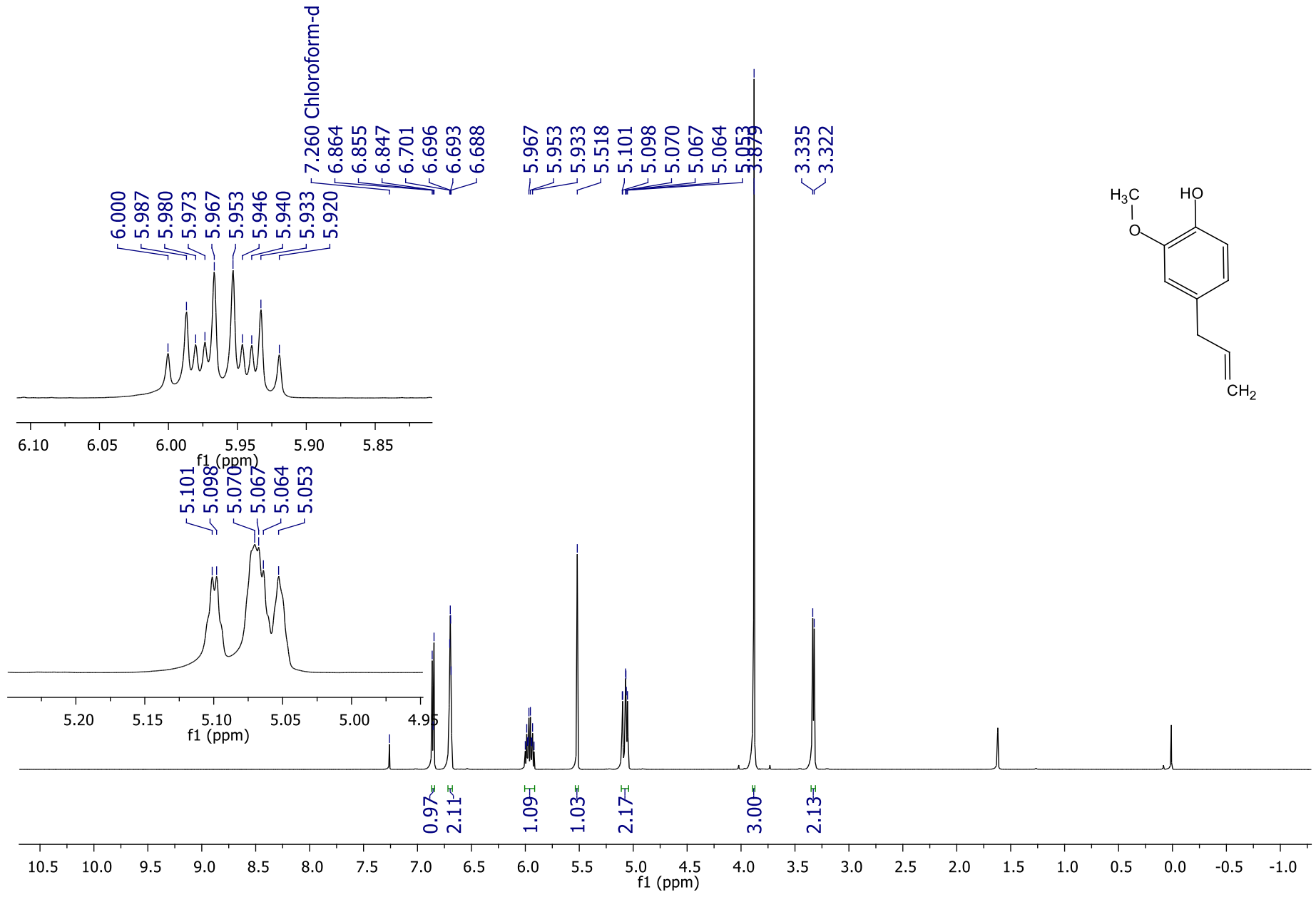

Figure S2. ^{1}H NMR of **1** (500 MHz, $CDCl_3$).

^{13}C NMR of 1 (126 MHz, $CDCl_3$)

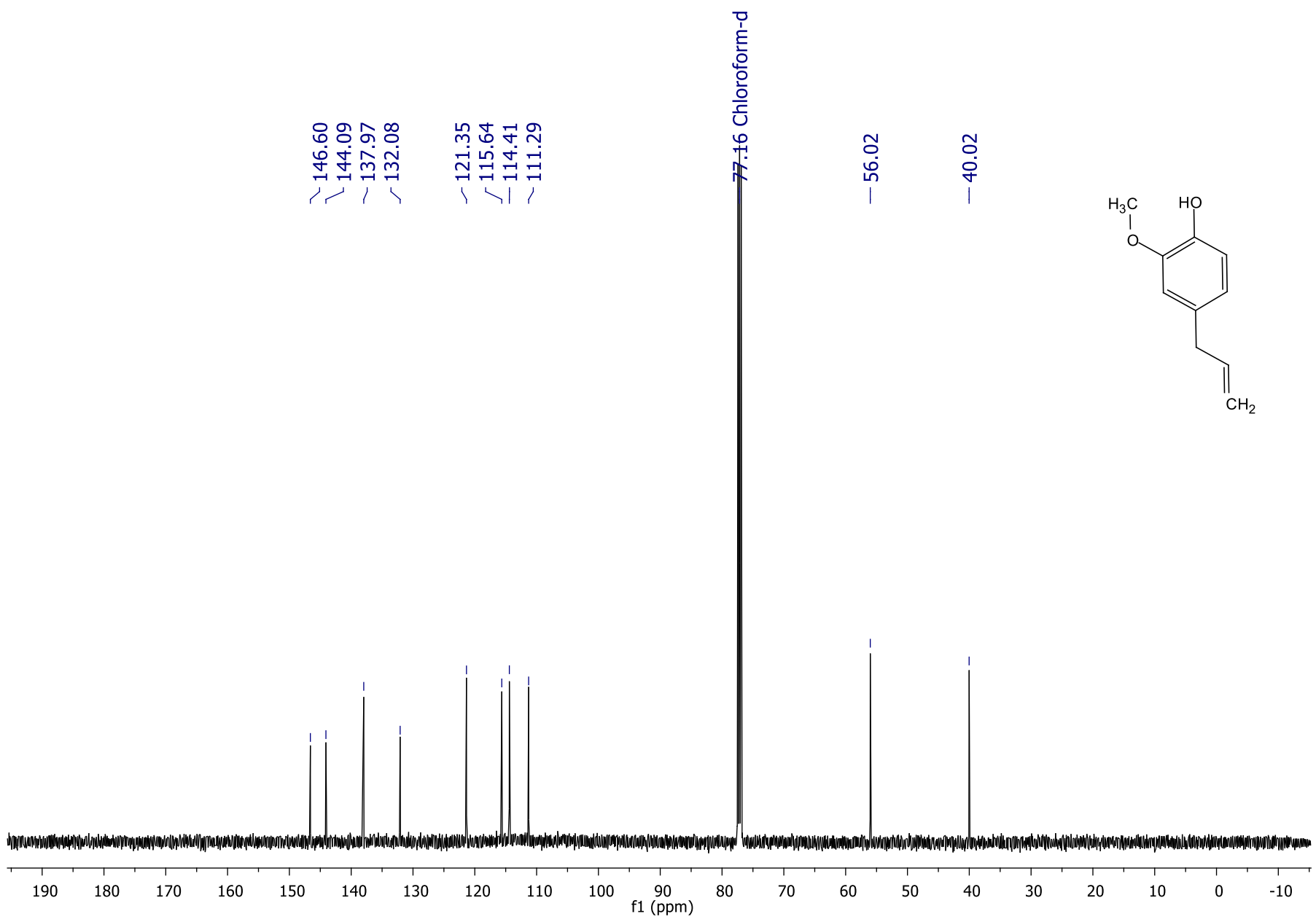

Figure S3. ^{13}C NMR of **1** (126 MHz, $CDCl_3$).

^{13}C NMR DEPT--135 of 1 (126 MHz, CDCl3)

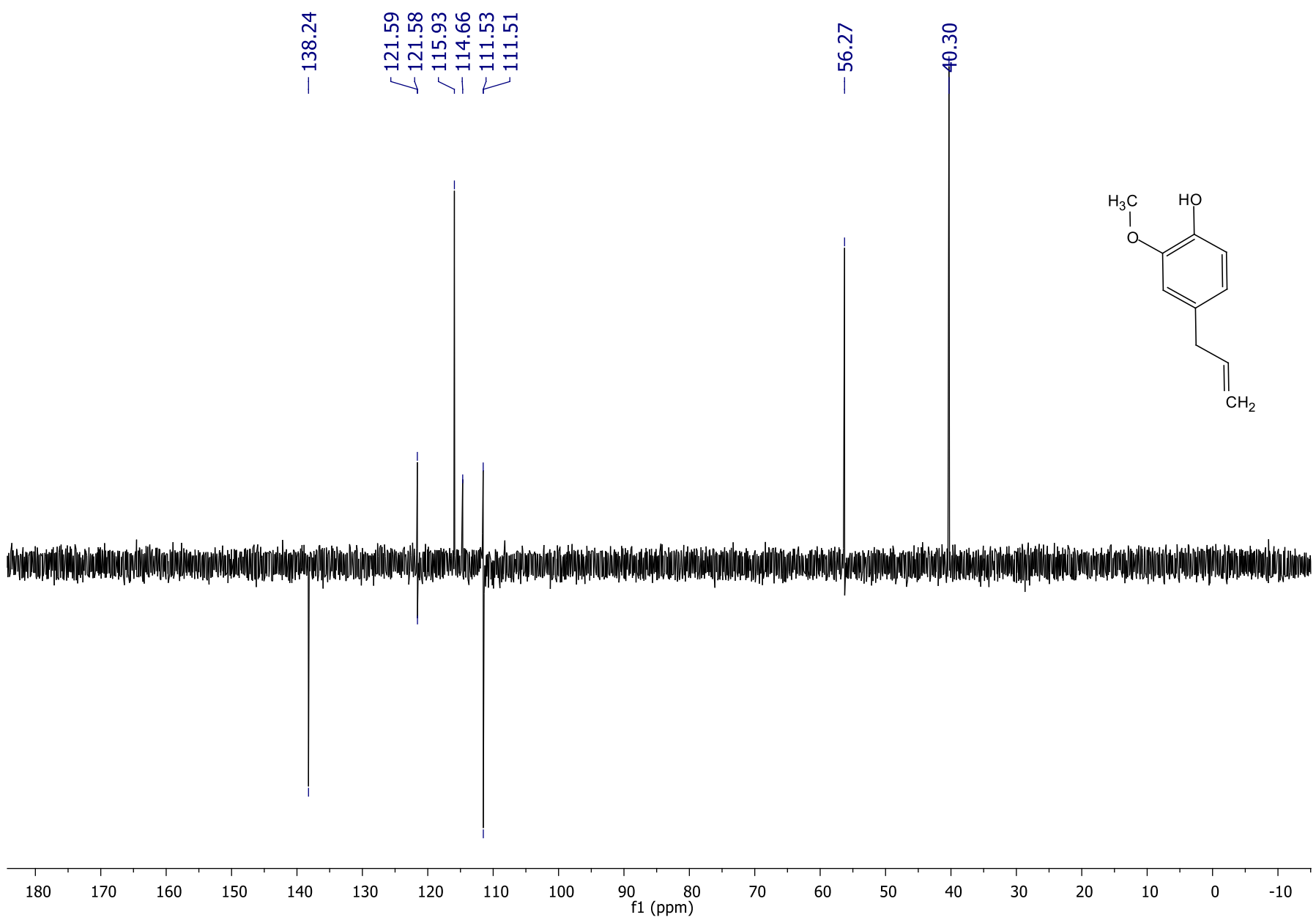

Figure S4. ^{13}C NMR DEPT--135 of **1** (126 MHz, $CDCl_3$).

^{1}H NMR of 2 (400 MHz, CDCl3)

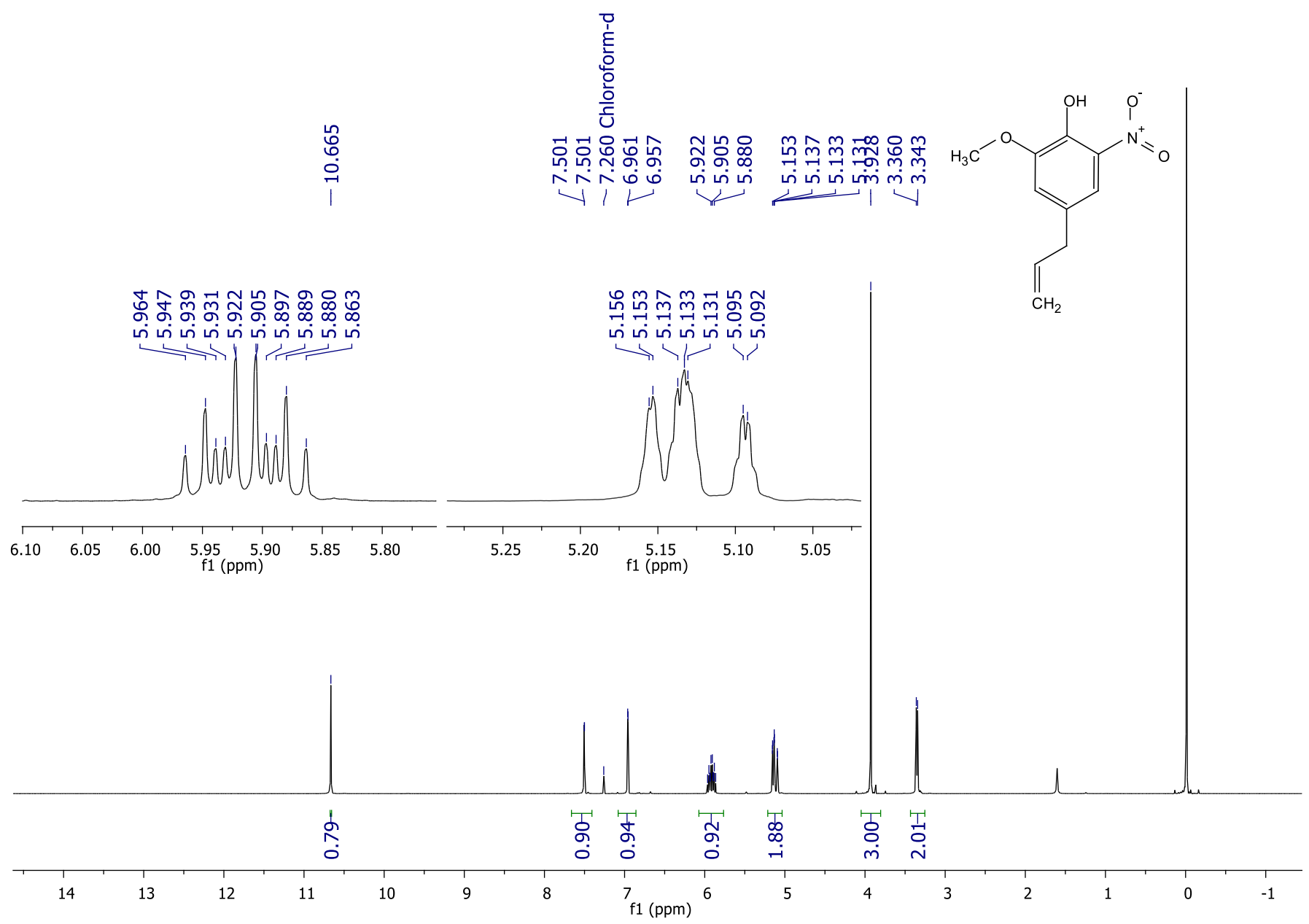

Figure S5. ^{1}H NMR of **2** (400 MHz, $CDCl_3$).

^{13}C NMR of 2 (101 MHz, $CDCl_3$)

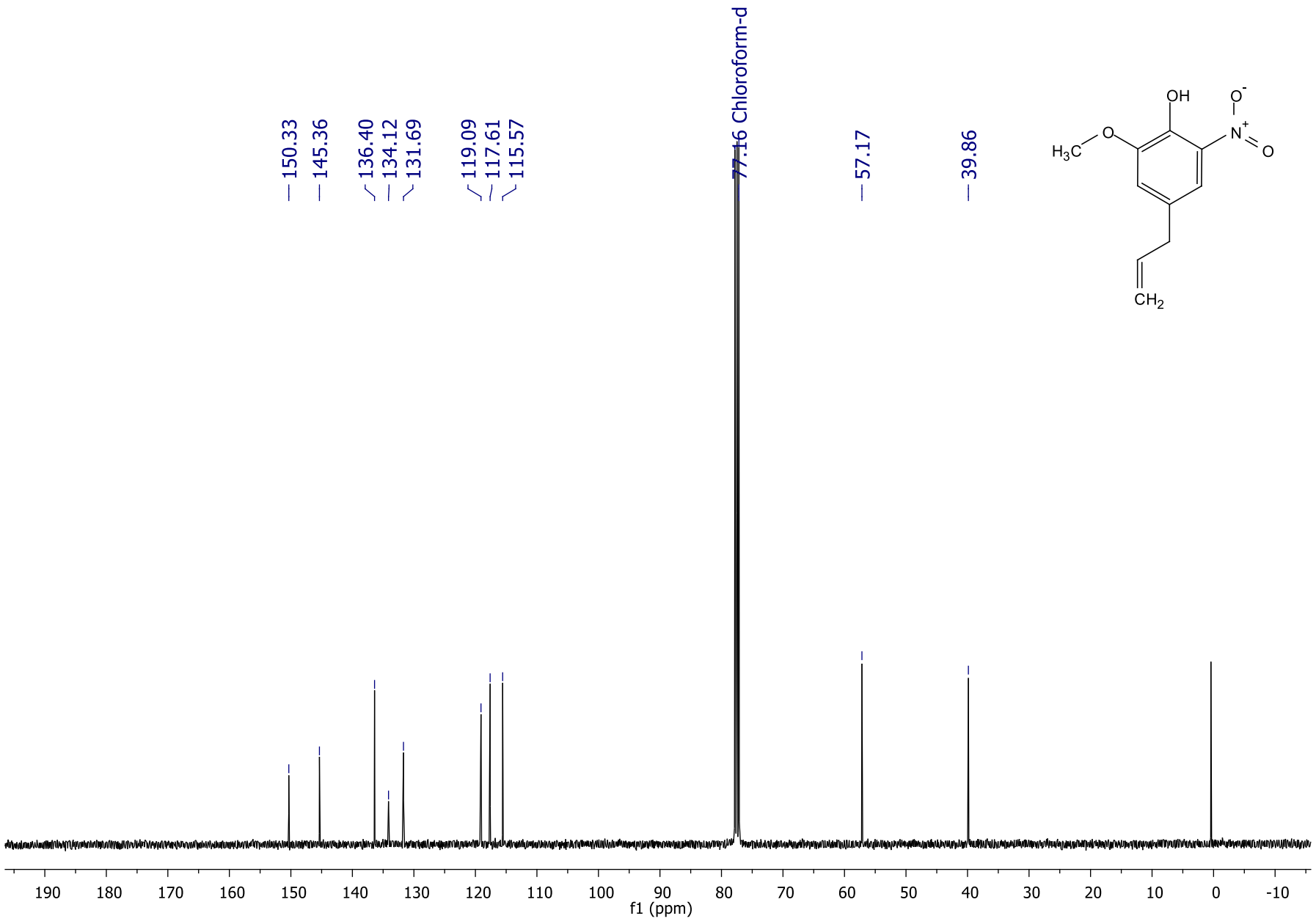

Figure S6. ^{13}C NMR of **2** (101 MHz, $CDCl_3$).

^{13}C NMR DEPT--135 of 2 (101 MHz, $CDCl_3$)

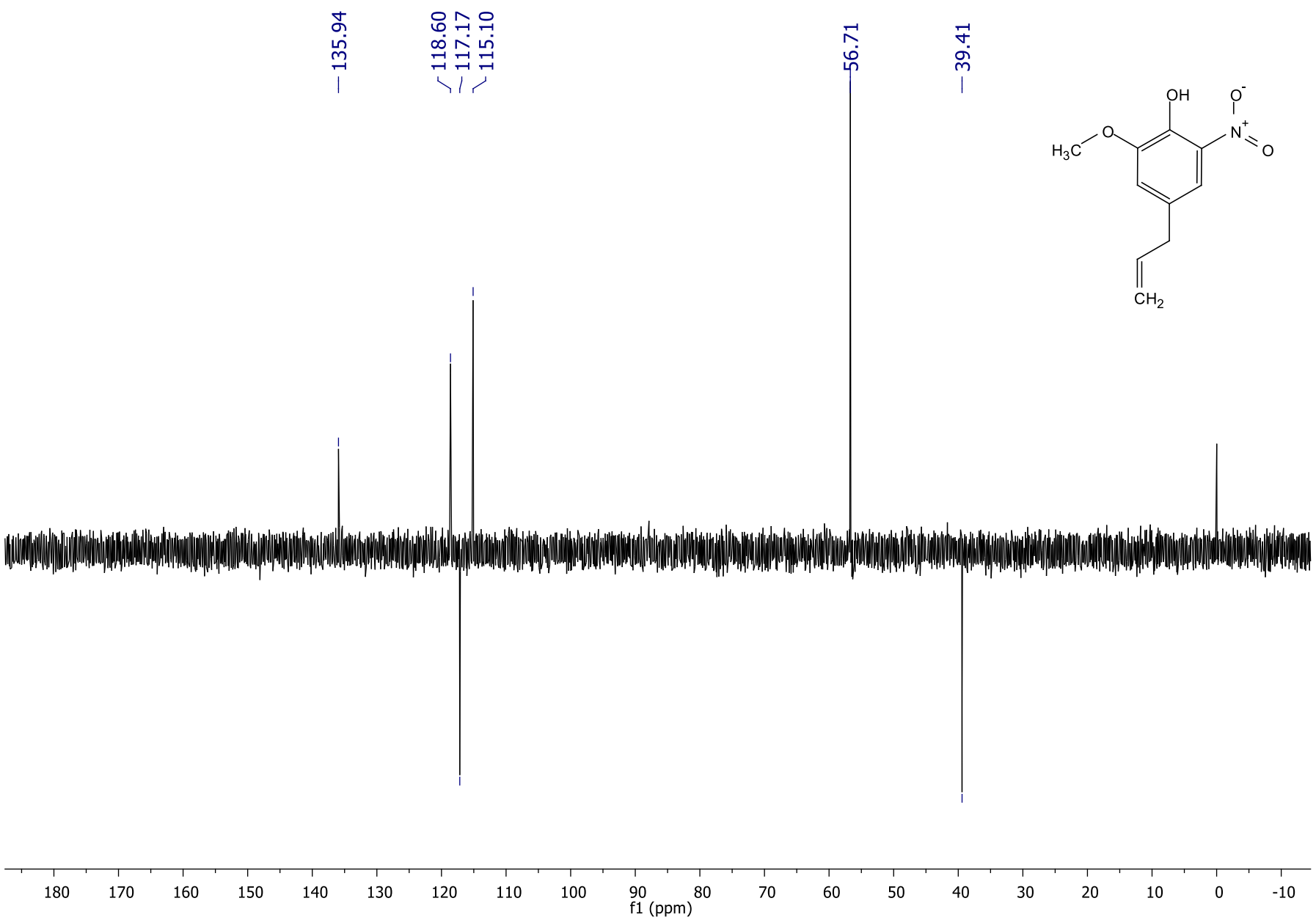

Figure S7. ^{13}C NMR DEPT--135 of **2** (101 MHz, $CDCl_3$).

^{1}H NMR of 3 (400 MHz, CDCl$_3$)

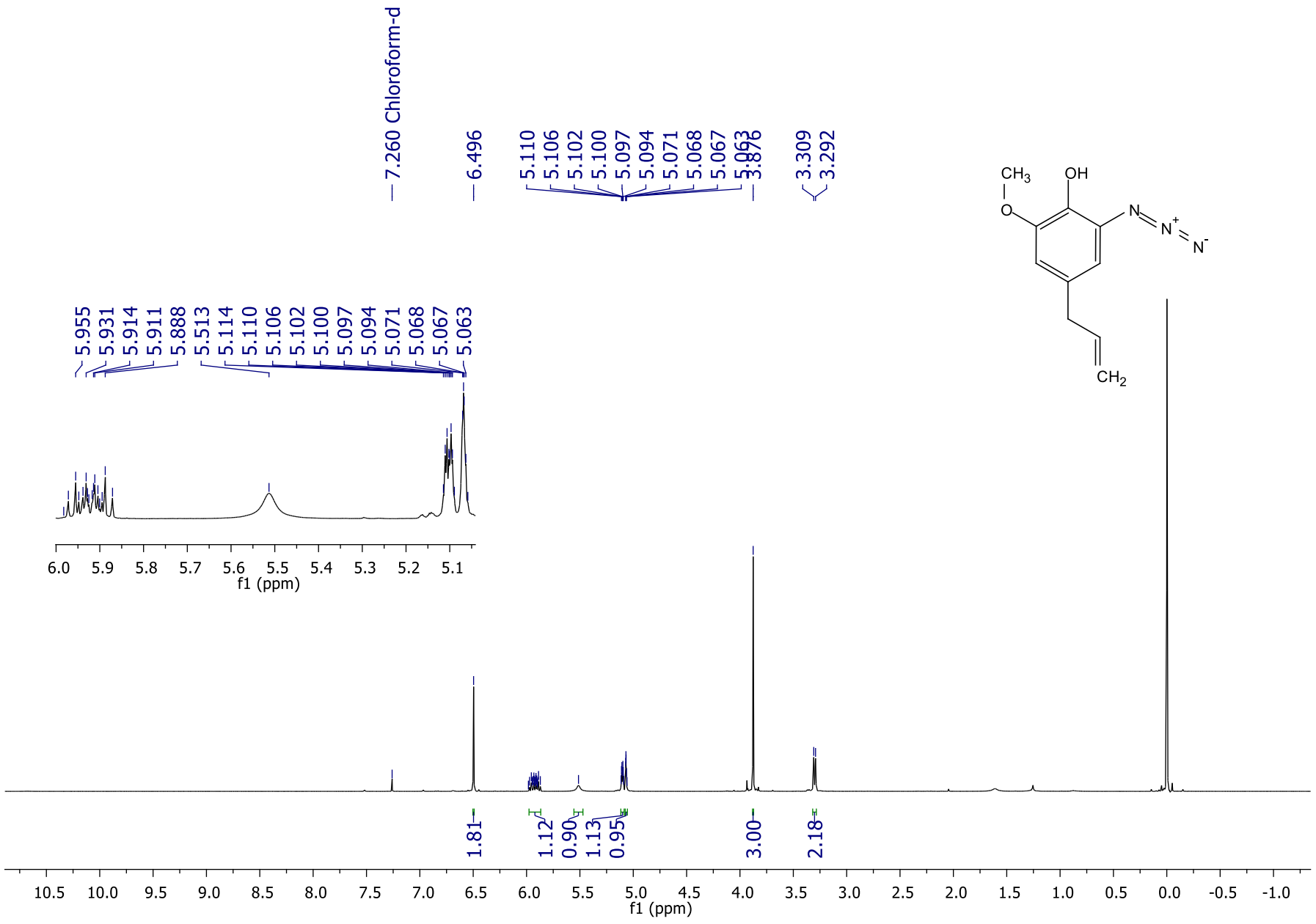

Figure S8. ^{1}H NMR of **3** (400 MHz, CDCl$_3$).

^{13}C NMR of 3 (101 MHz, CDCl$_3$)

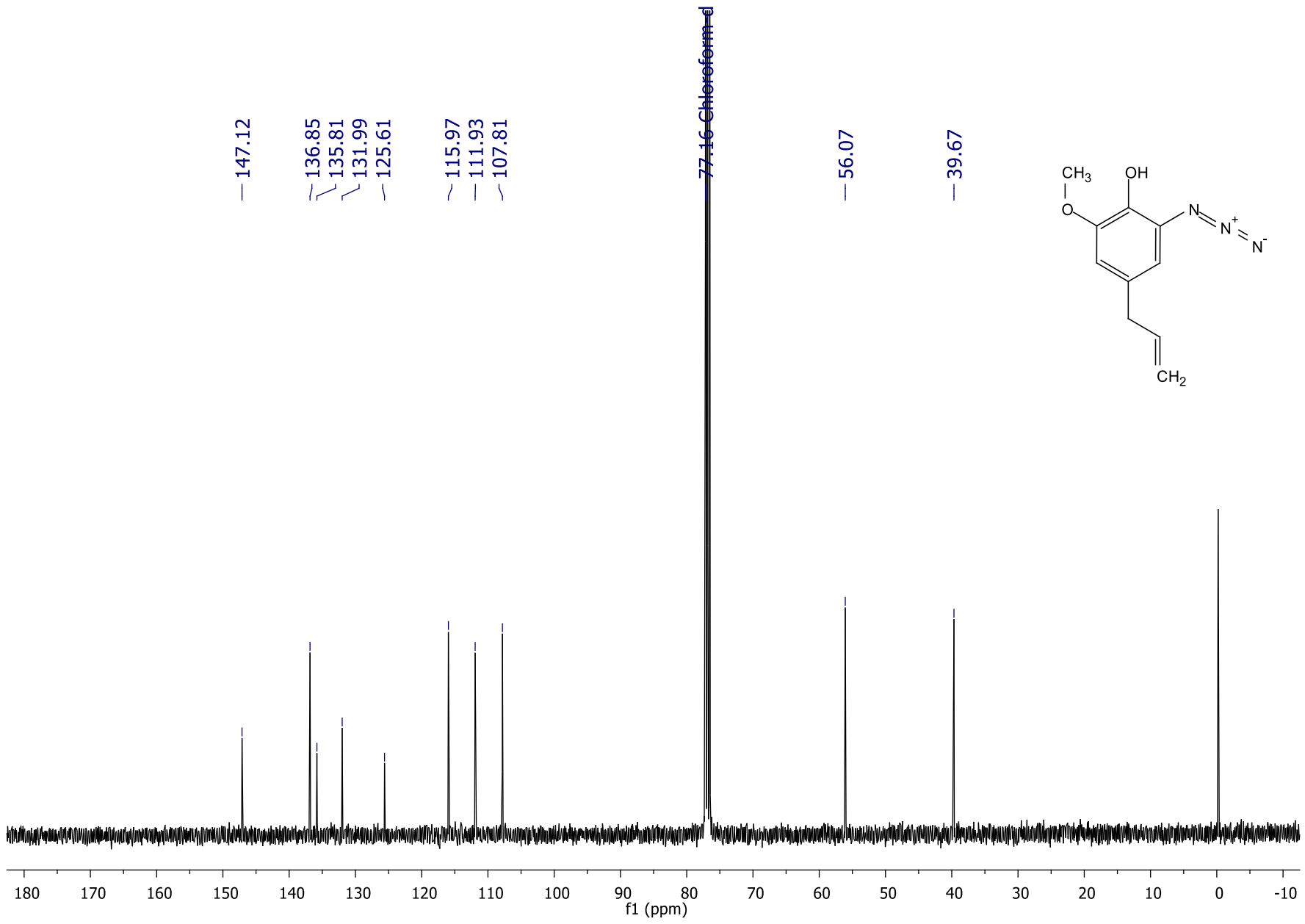

Figure S9. ^{13}C NMR of **3** (101 MHz, CDCl$_3$).

^{13}C NMR DEPT--135 of 3 (101 MHz, $CDCl_3$)

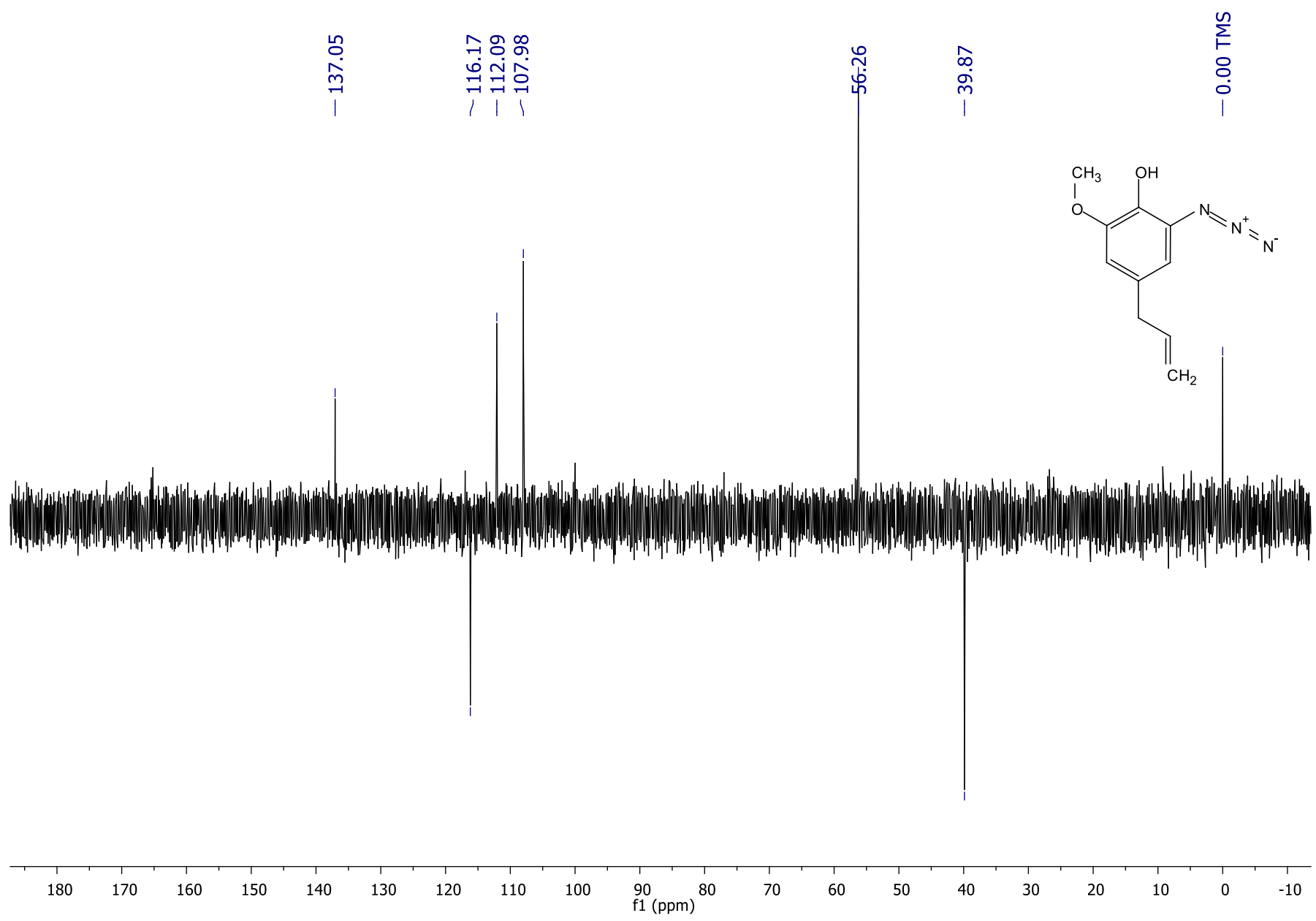

Figure S10. ^{13}C NMR DEPT--135 of **3** (101 MHz, $CDCl_3$).

^{1}H NMR of 4a (400 MHz, $CDCl_3$)

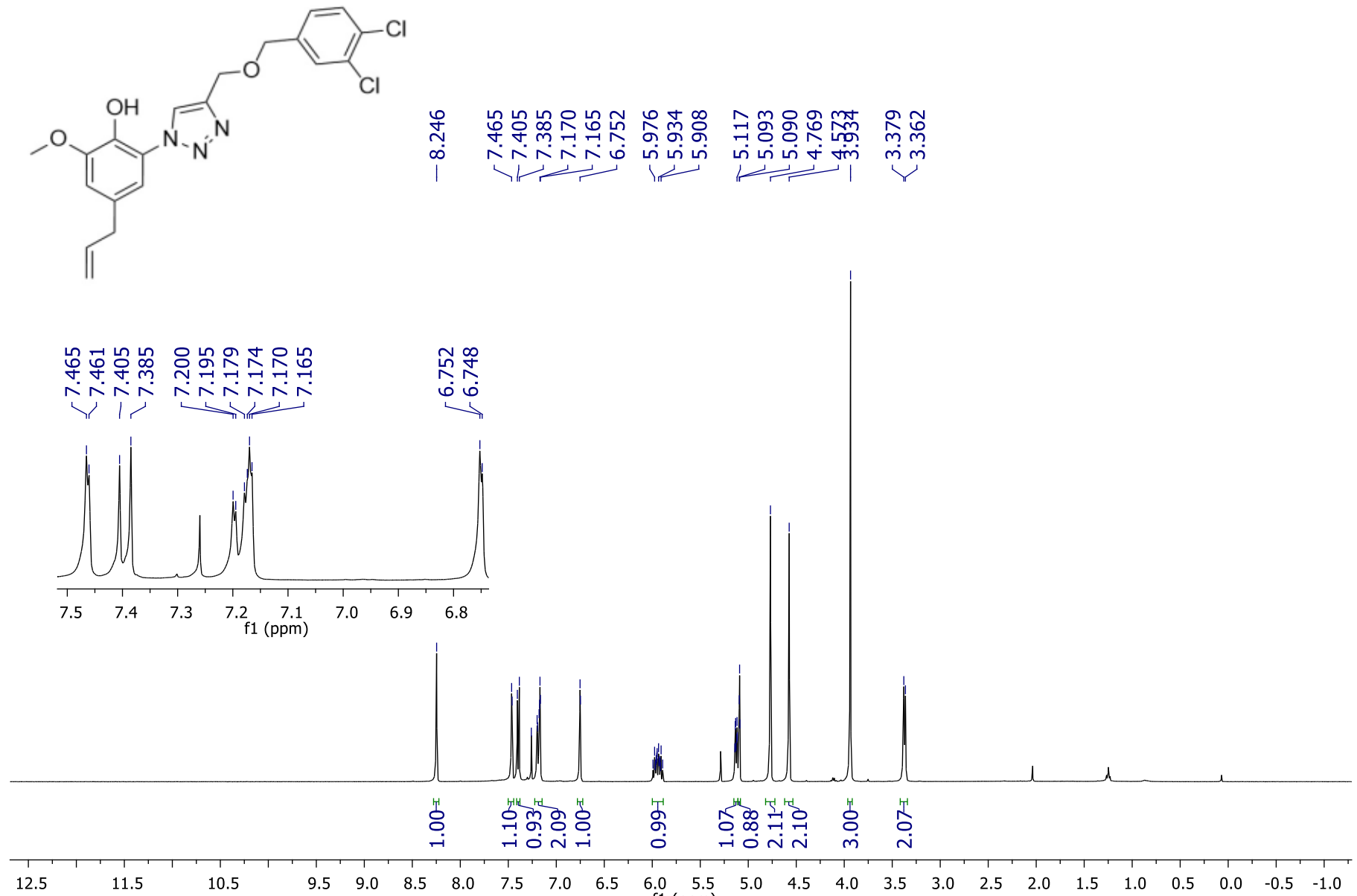

Figure S11. ^{1}H NMR of **4a** (400 MHz, $CDCl_3$).

^{13}C NMR of 4a (101 MHz, CDCl$_3$)

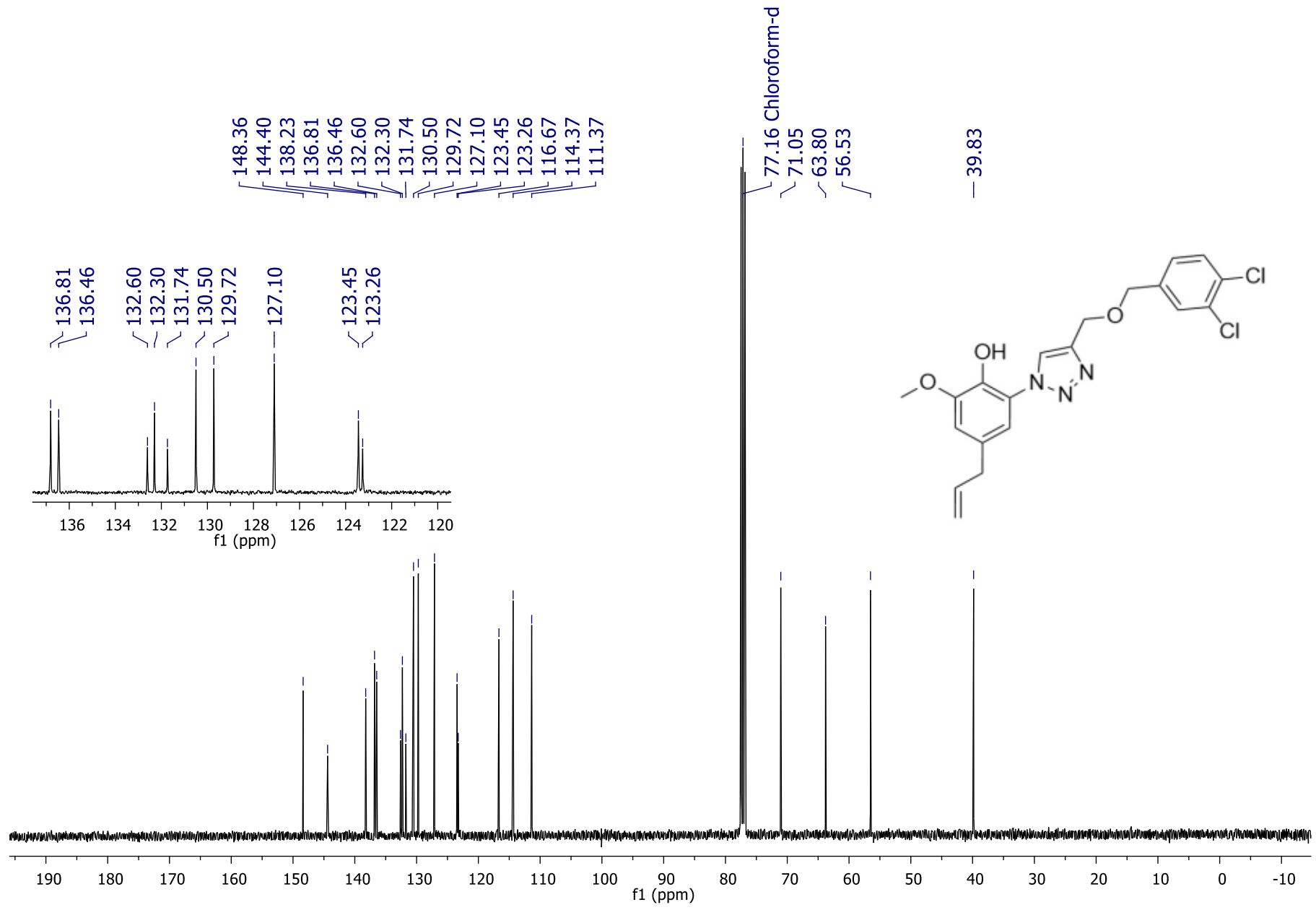

Figure S12. ^{13}C NMR of **4a** (101 MHz, CDCl$_3$).

^{13}C NMR DEPT--135 of 4a (101 MHz, CDCl$_3$)

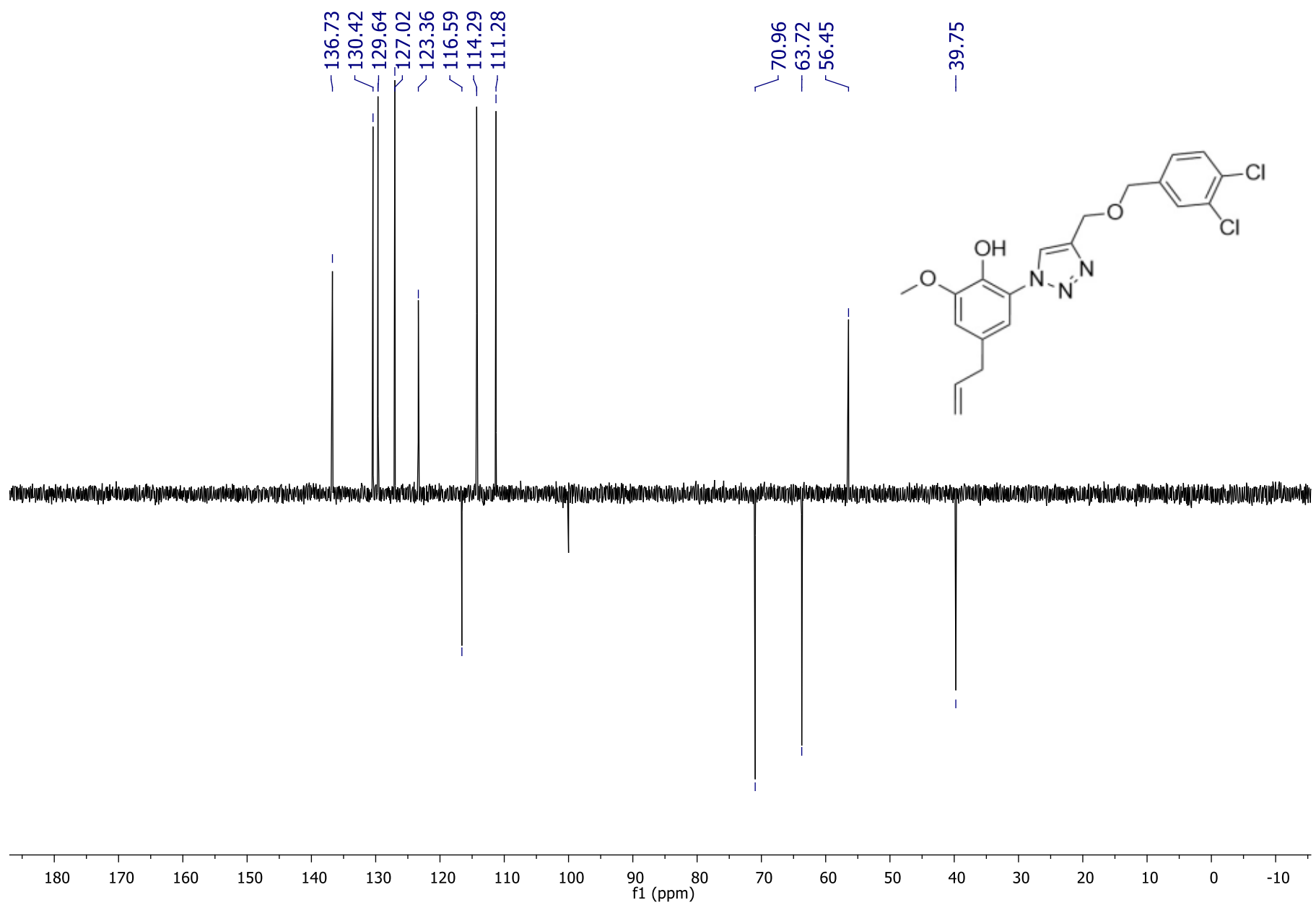

Figure S13. ^{13}C NMR DEPT--135 of **4a** (101 MHz, CDCl$_3$).

^{1}H/^{13}C NMR HSQC of 4a (400 and 101 MHz, CDCl$_3$)

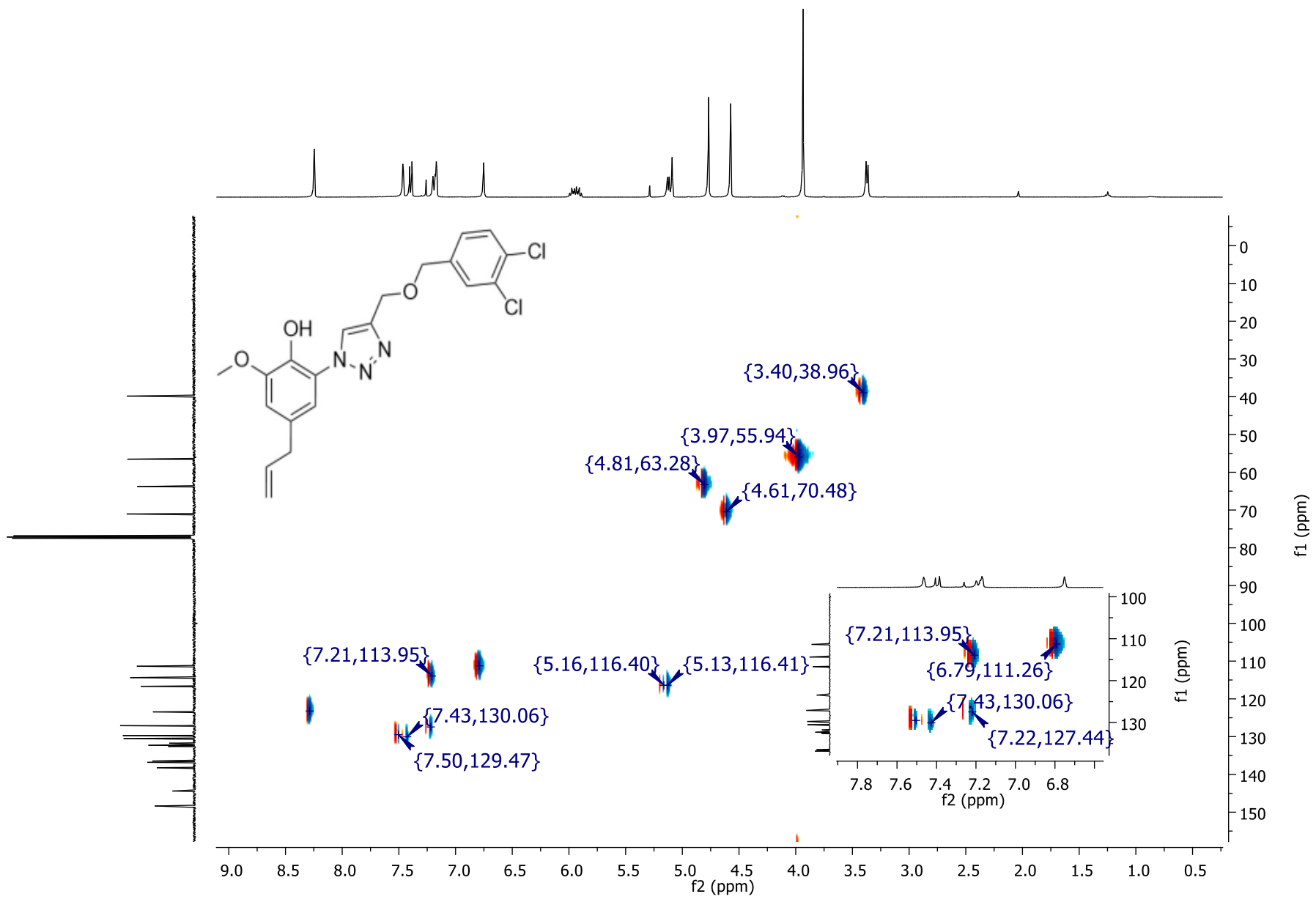

Figure S14. ^{1}H/^{13}C NMR HSQC of **4a** (400 and 101 MHz, CDCl$_3$).

^{1}H NMR of 4b (400 MHz, CDCl$_3$)

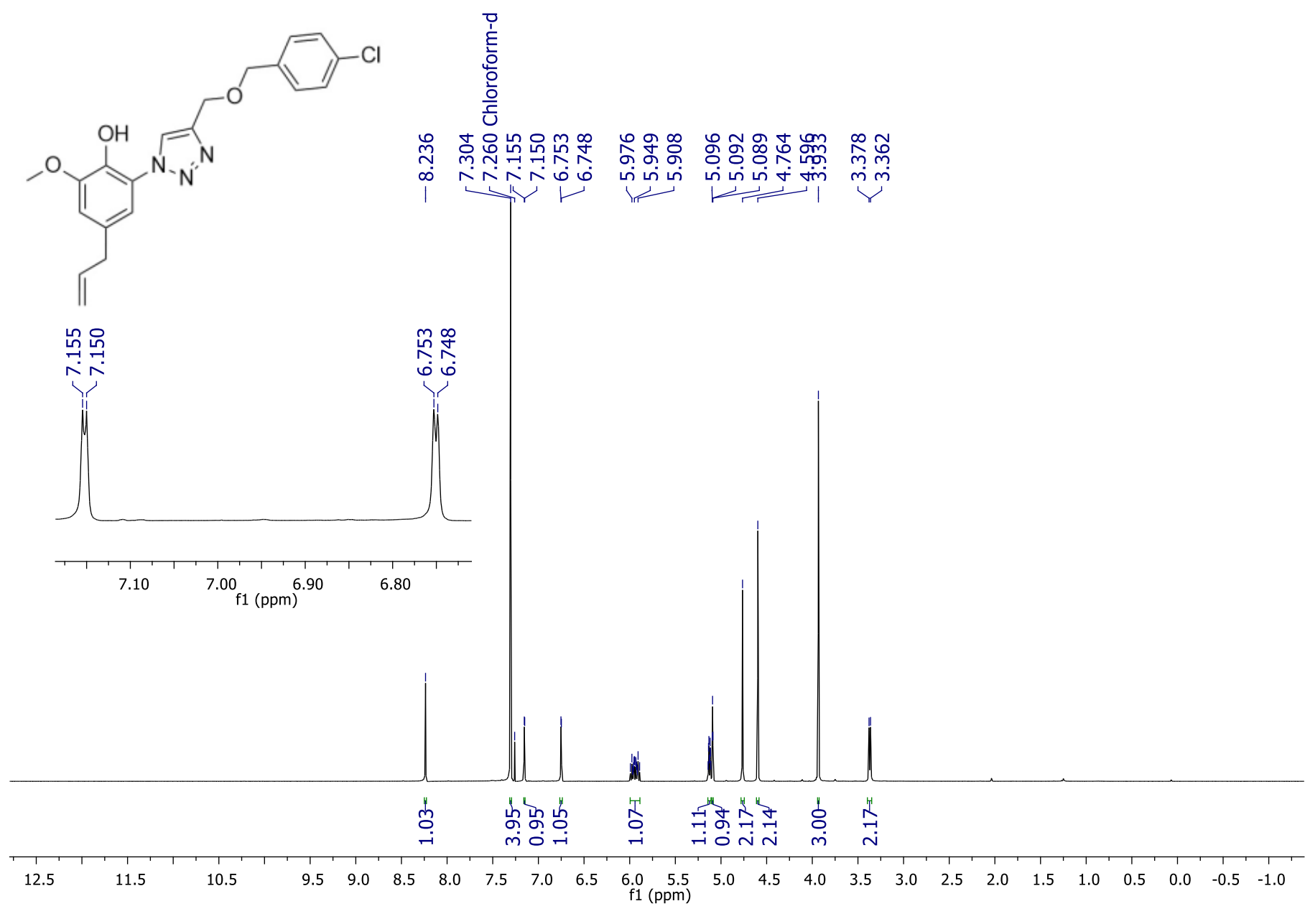

Figure S15. ^{1}H NMR of **4b** (400 MHz, CDCl$_3$).

^{13}C NMR of 4b (101 MHz, $CDCl_3$)

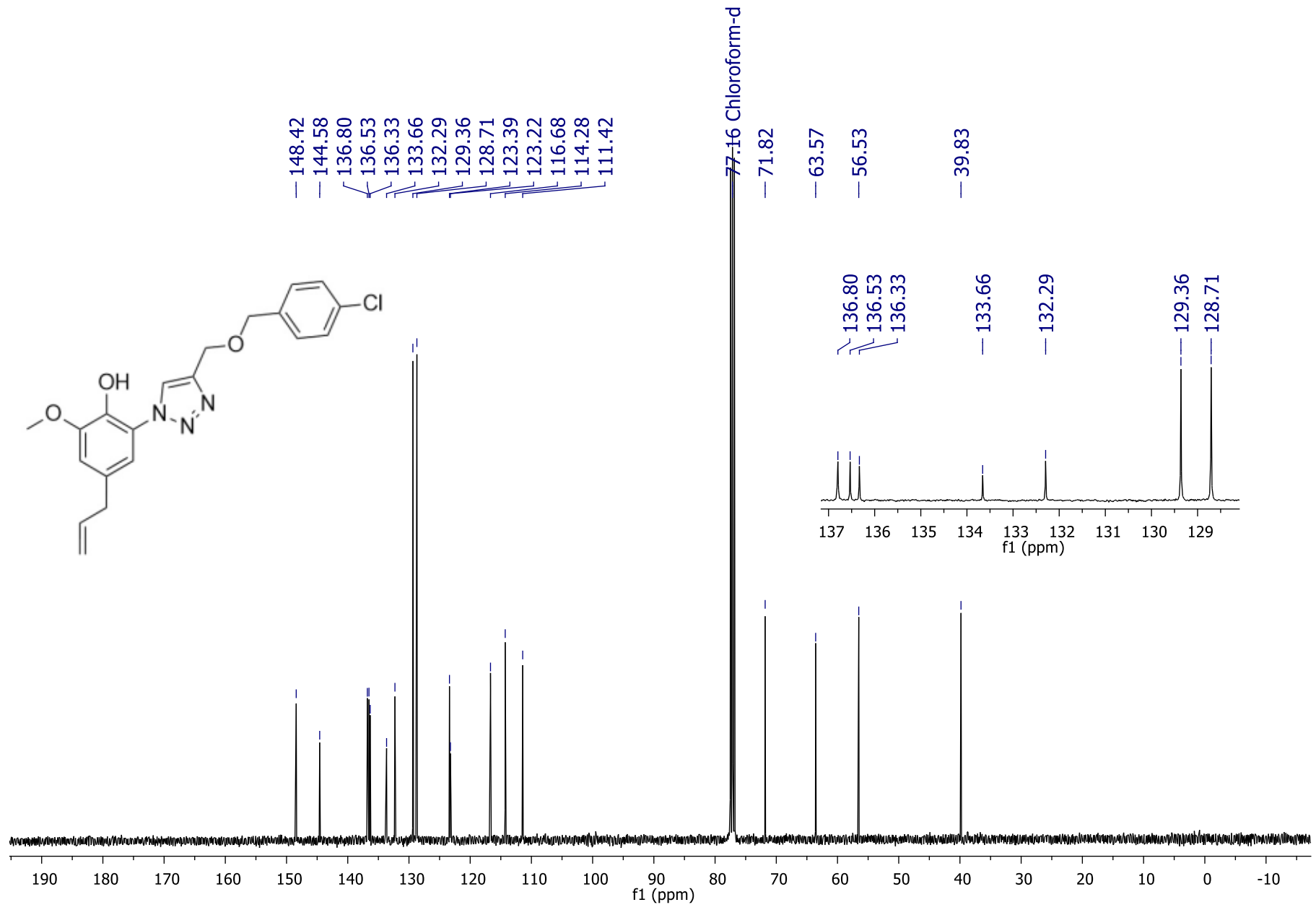

Figure S16. ^{13}C NMR of **4b** (101 MHz, $CDCl_3$).

^{13}C NMR DEPT--135 of 4b (101 MHz, $CDCl_3$)

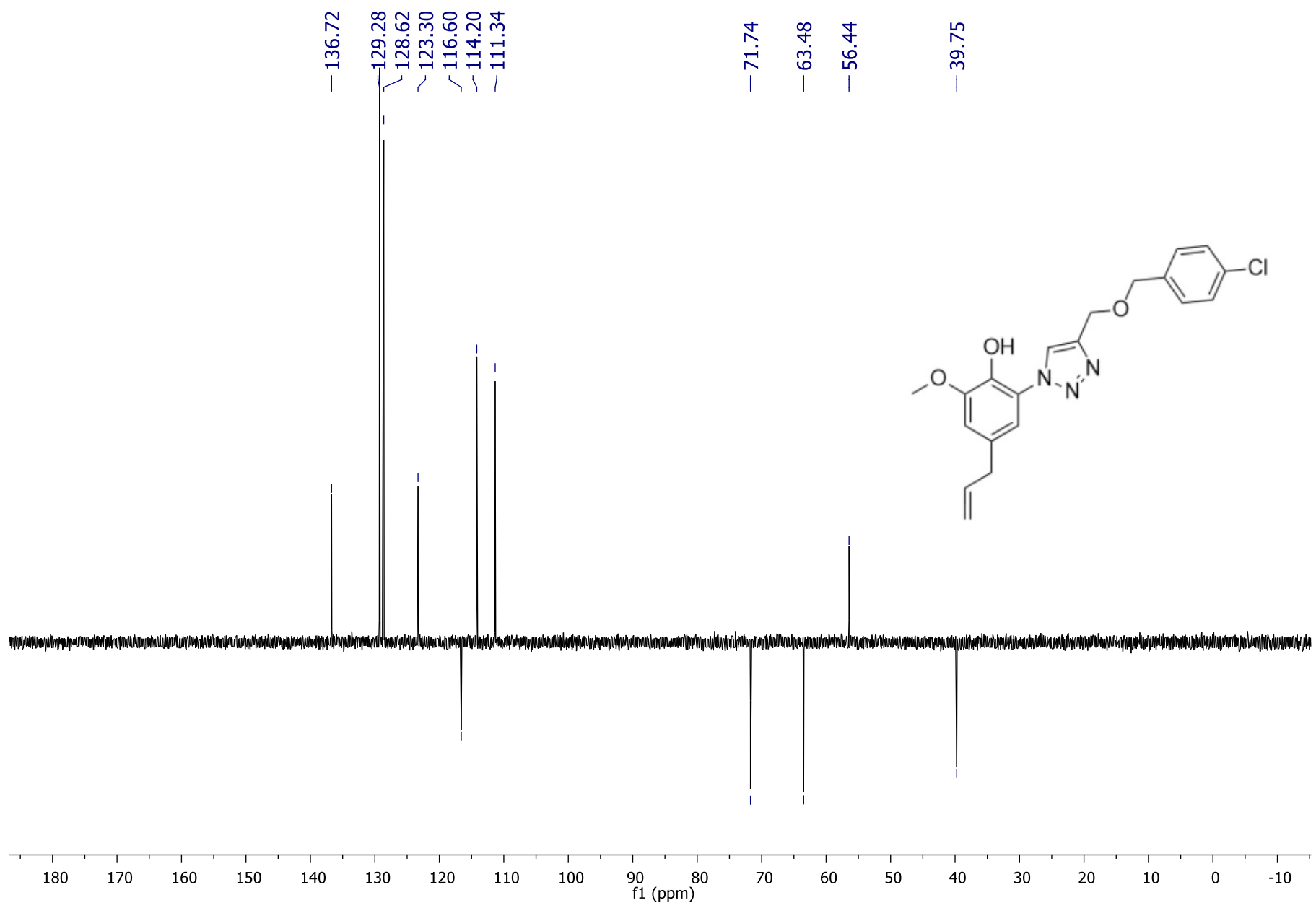

Figure S17. ^{13}C NMR DEPT--135 of **4b** (101 MHz, $CDCl_3$).

$^{1}H/^{13}C$ NMR HSQC of 4b (400 and 101 MHz, $CDCl_3$)

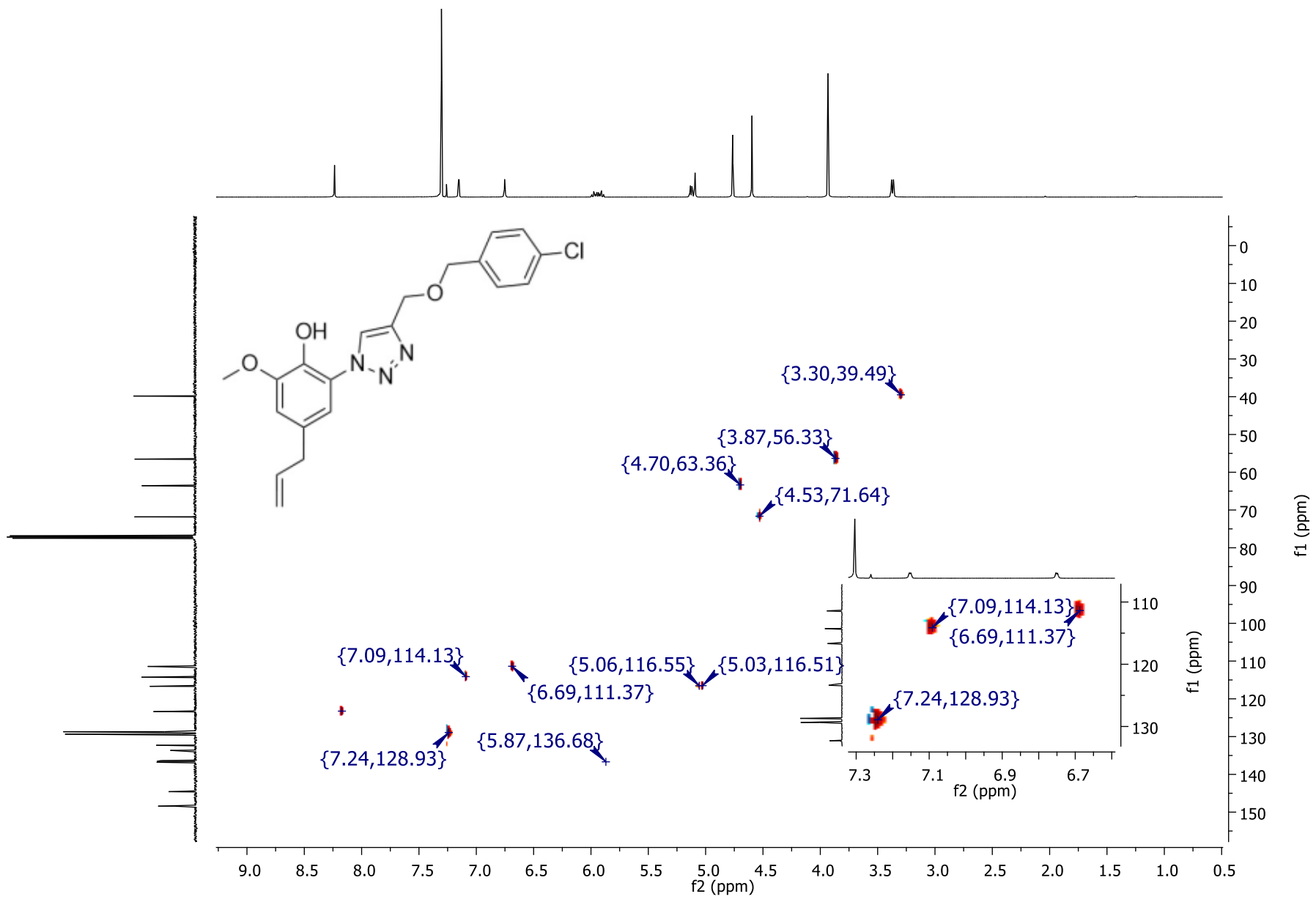

Figure S18. $^{1}H/^{13}C$ NMR HSQC of **4b** (400 and 101 MHz, $CDCl_3$).

^{1}H NMR of 4c (400 MHz, $CDCl_3$)

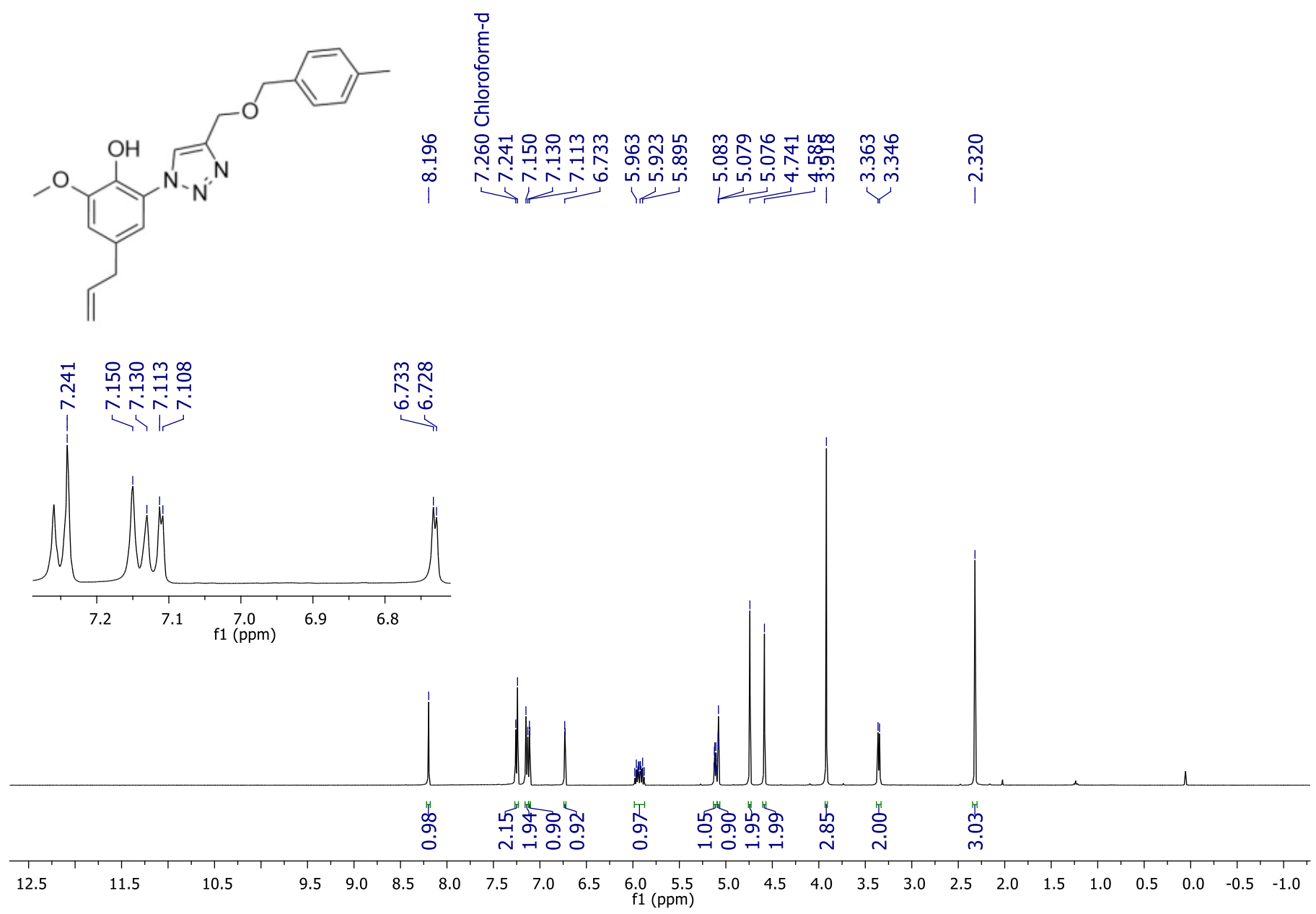

Figure S19. ^{1}H NMR of **4c** (400 MHz, $CDCl_3$).

^{13}C NMR of 4c (101 MHz, CDCl$_3$)

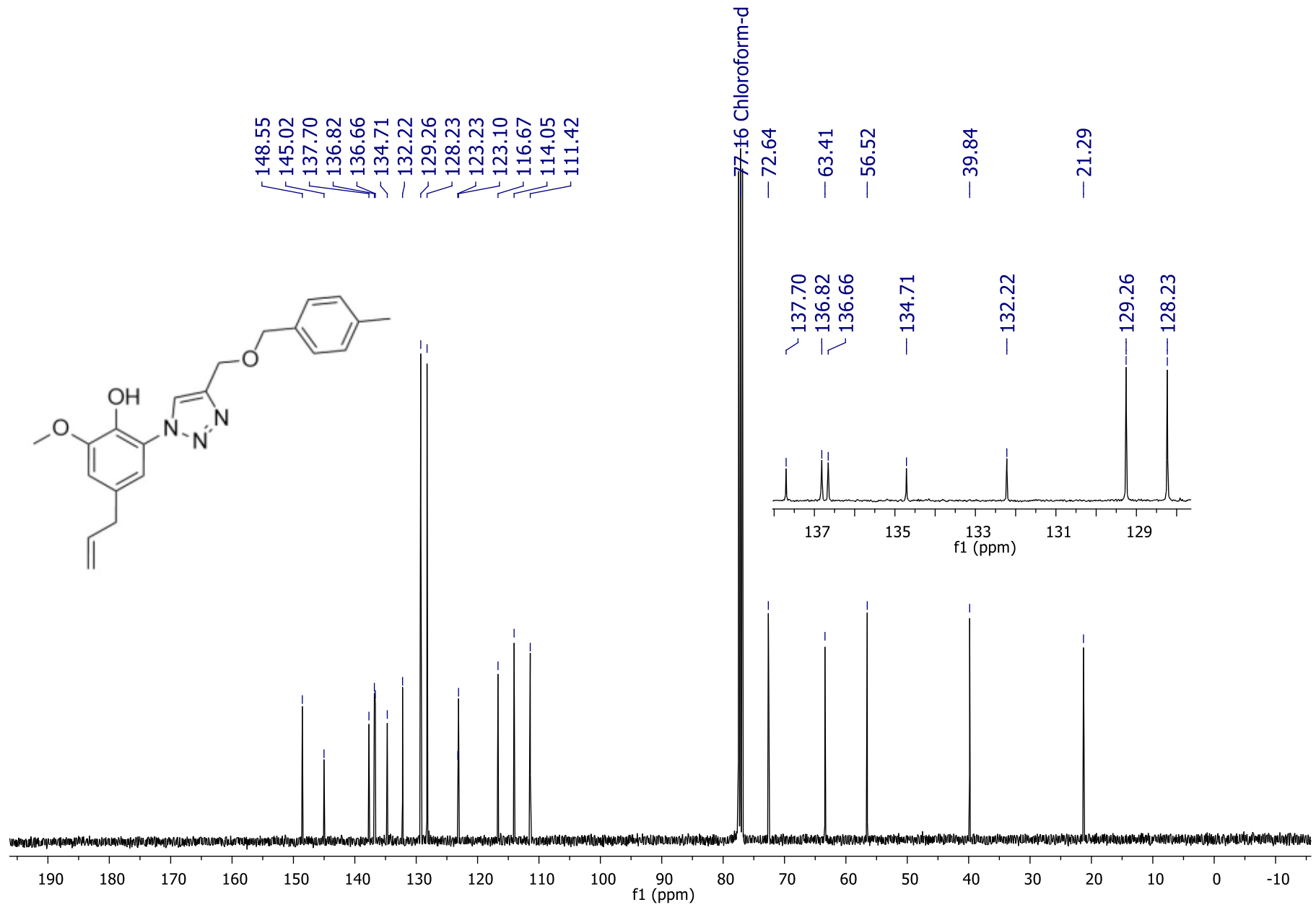

Figure S20. ^{13}C NMR of **4c** (101 MHz, CDCl$_3$).

^{13}C NMR DEPT--135 of 4c (101 MHz, CDCl$_3$)

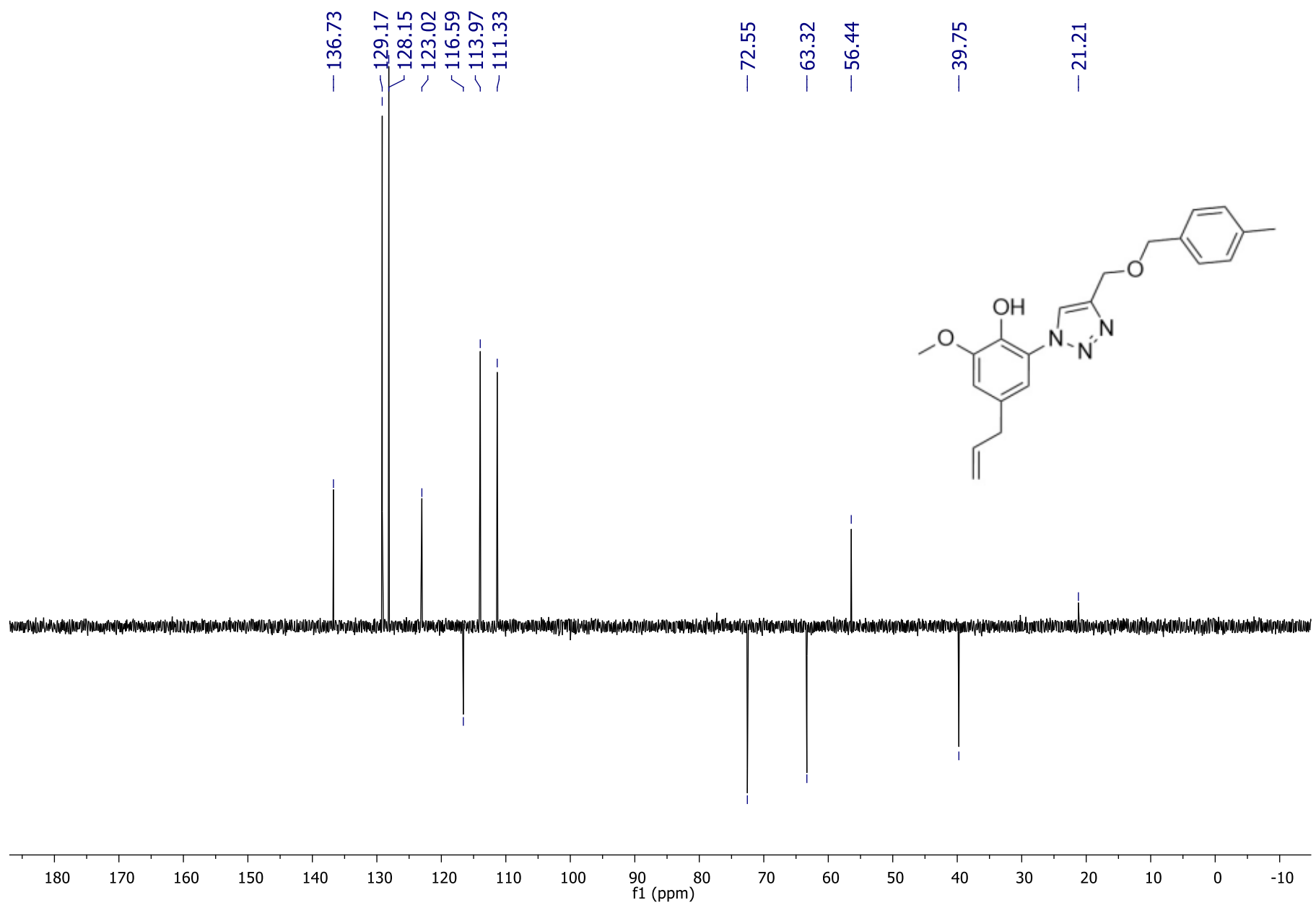

Figure S21. ^{13}C NMR DEPT--135 of **4c** (101 MHz, CDCl$_3$).

$^{1}H/^{13}C$ NMR HSQC of 4c (400 and 101 MHz, $CDCl_3$)

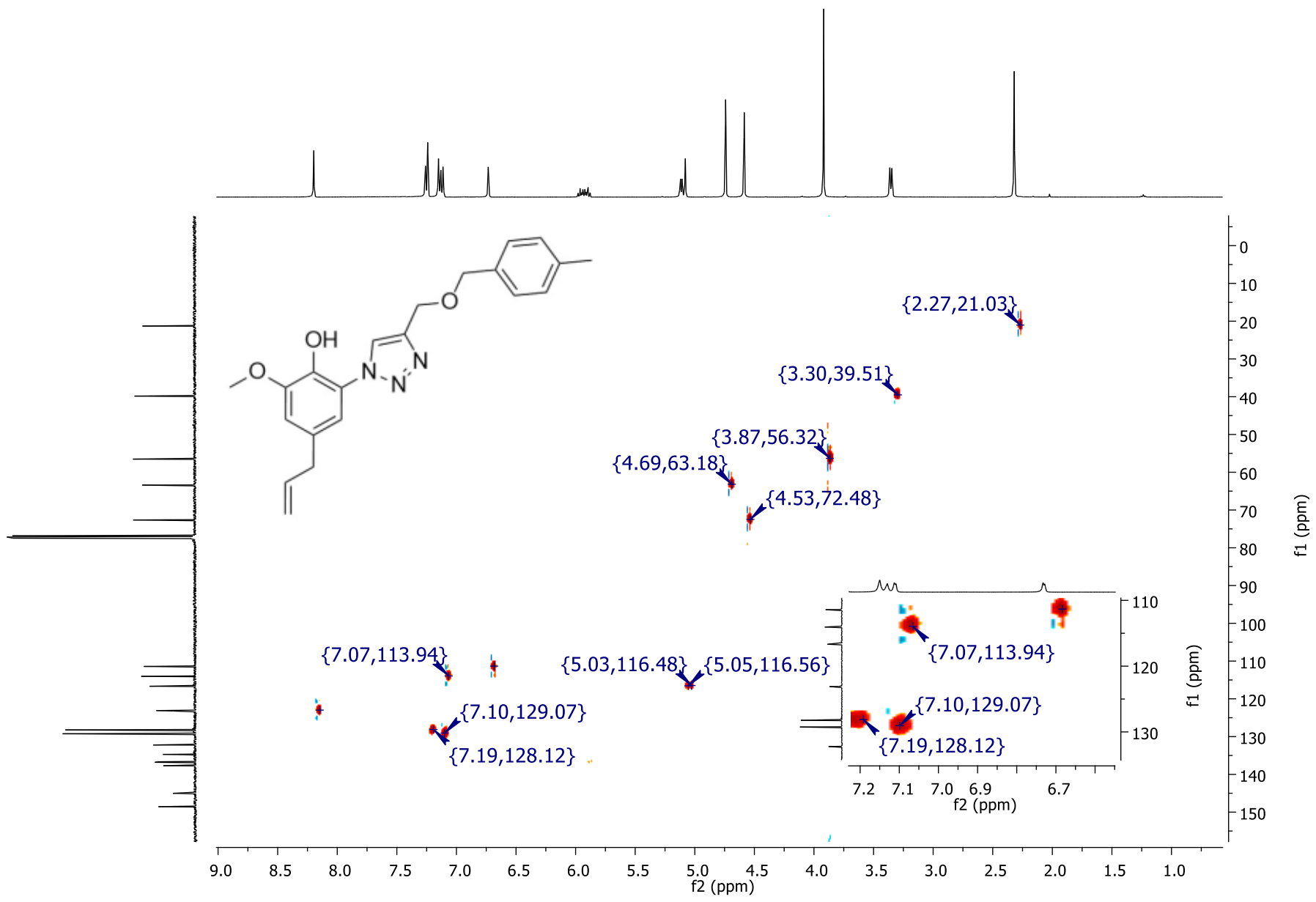

Figure S22. $^{1}H/^{13}C$ NMR HSQC of **4c** (400 and 101 MHz, $CDCl_3$).

^{1}H NMR of 4d (400 MHz, $CDCl_3$)

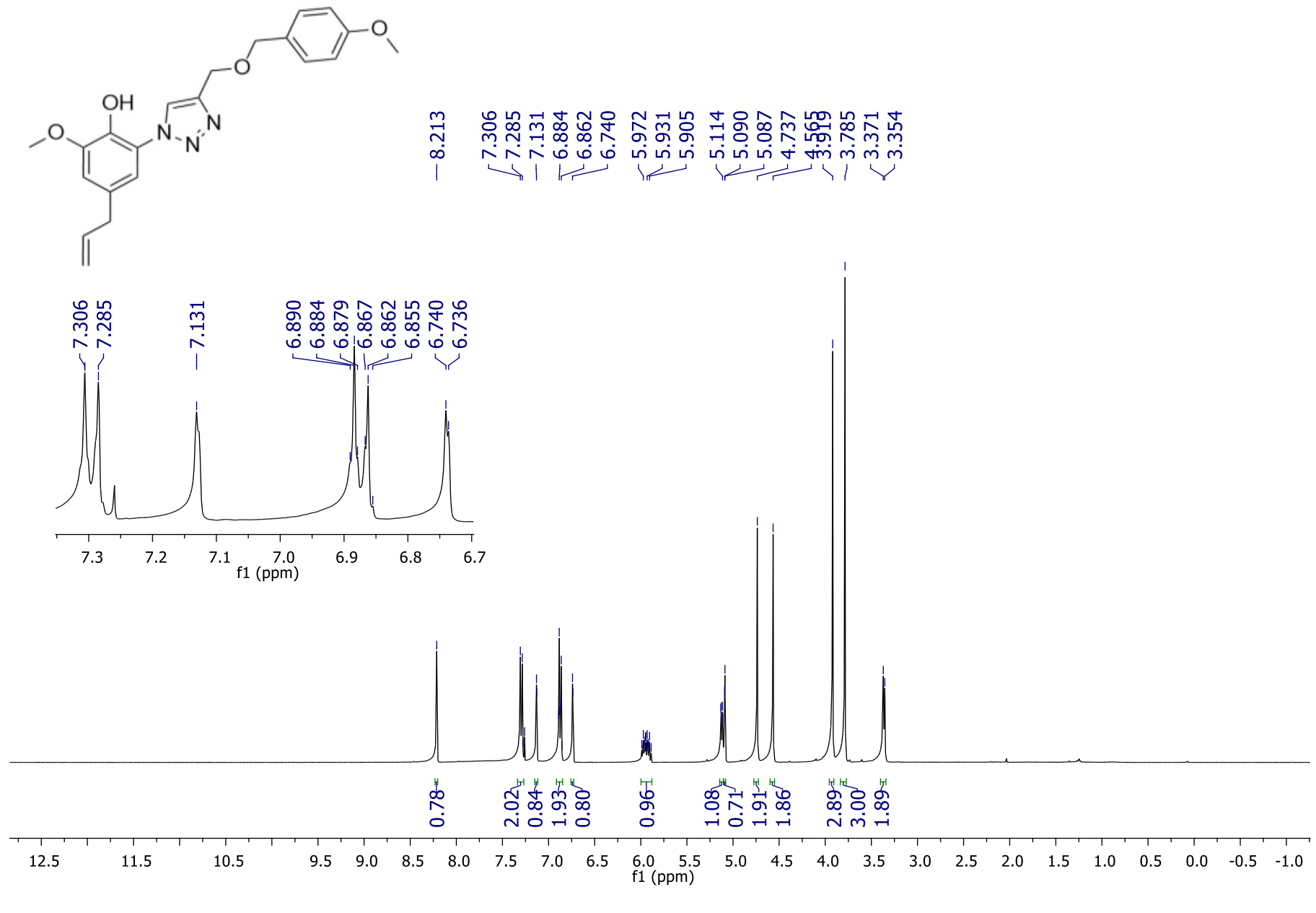

Figure S23. ^{1}H NMR of **4d** (400 MHz, $CDCl_3$).

^{13}C NMR of 4d (101 MHz, CDCl$_3$)

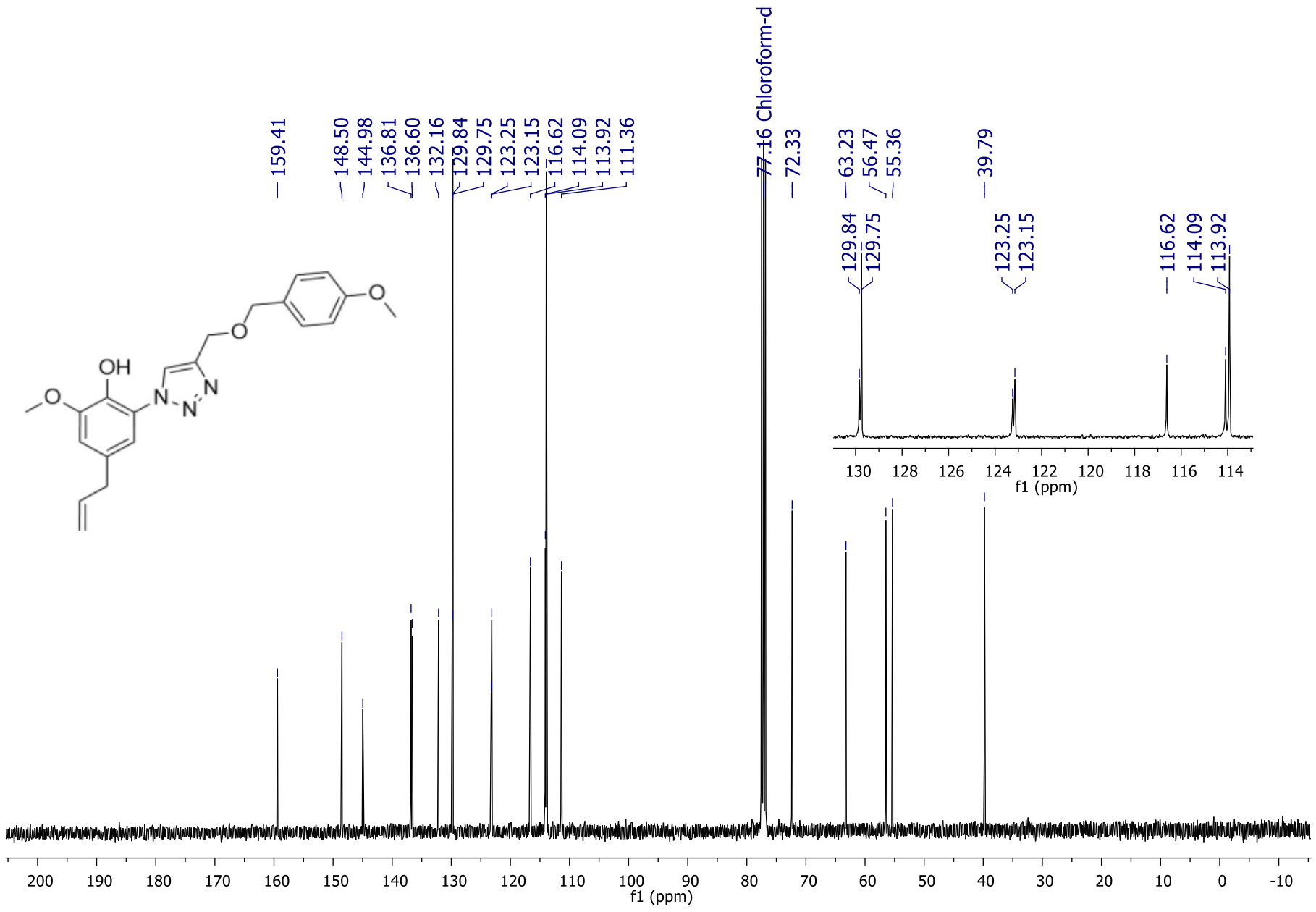

Figure S24. ^{13}C NMR of **4d** (101 MHz, CDCl$_3$).

^{13}C NMR DEPT--135 of 4d (101 MHz, CDCl$_3$)

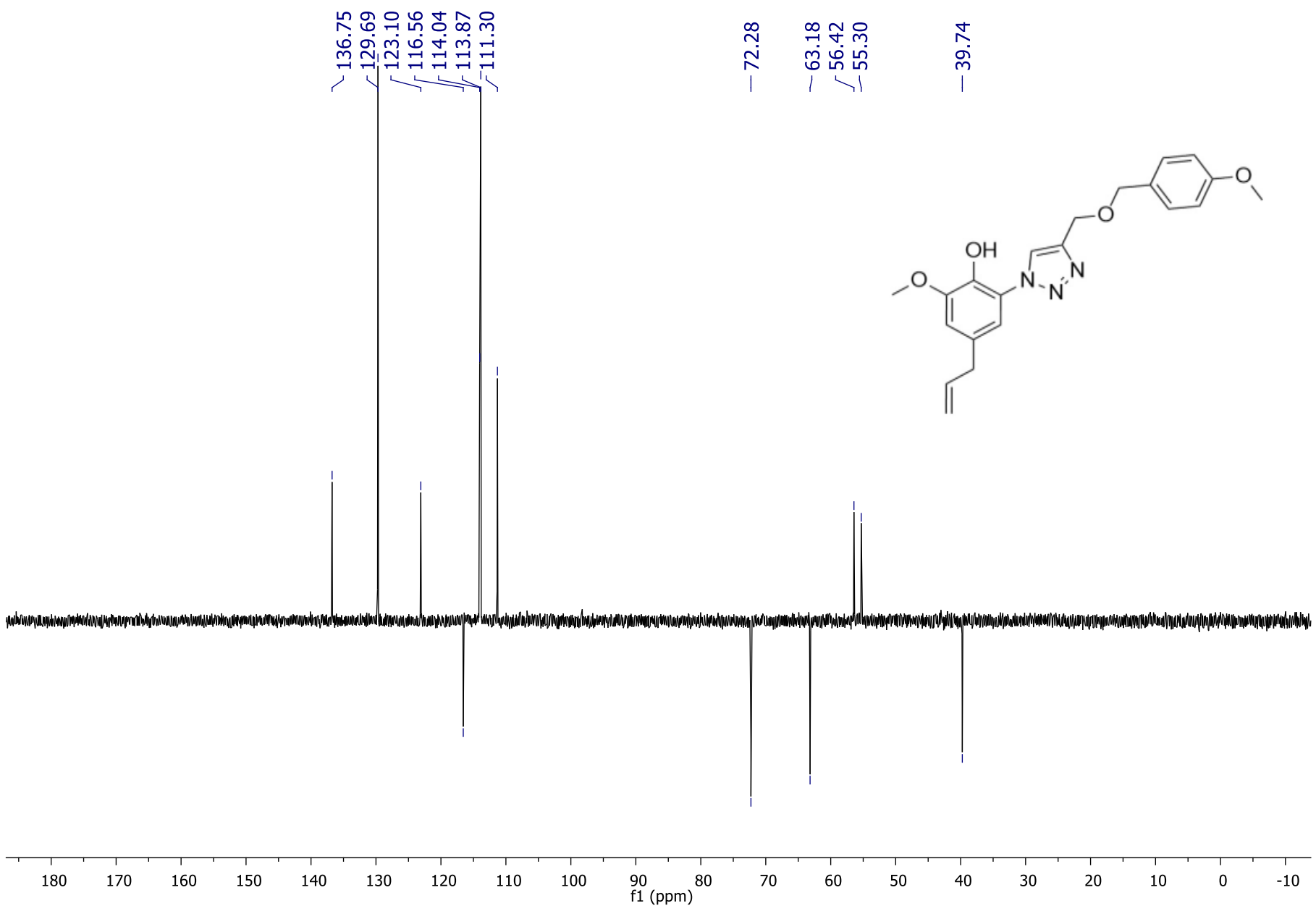

Figure S25. ^{13}C NMR DEPT--135 of **4d** (101 MHz, CDCl$_3$).

$^1H/^{13}C$ NMR HSQC of 4d (400 and 101 MHz, $CDCl_3$)

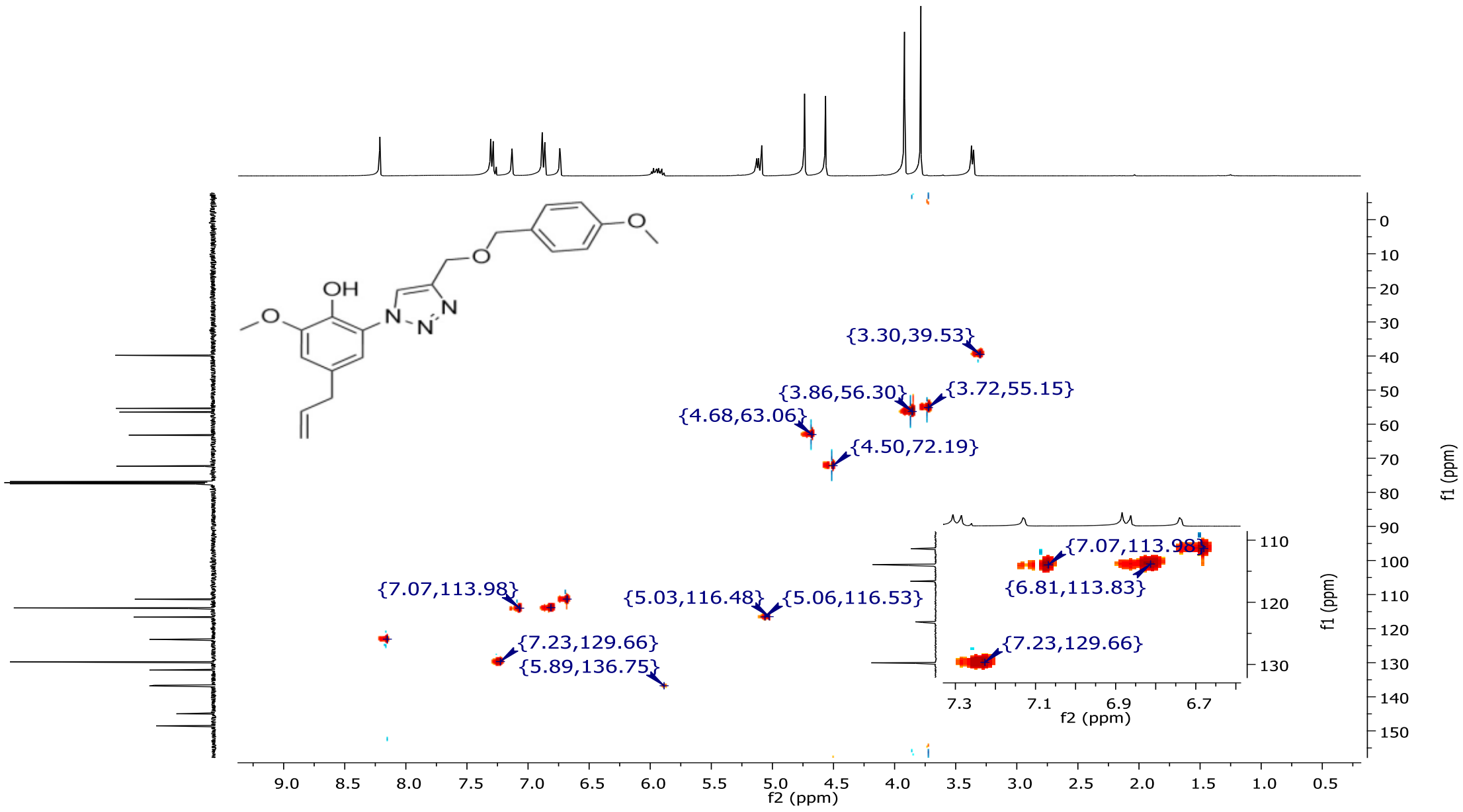

Figure S26. $^1H/^{13}C$ NMR HSQC of **4d** (400 and 101 MHz, $CDCl_3$).

^1H NMR of 4e (400 MHz, $CDCl_3$)

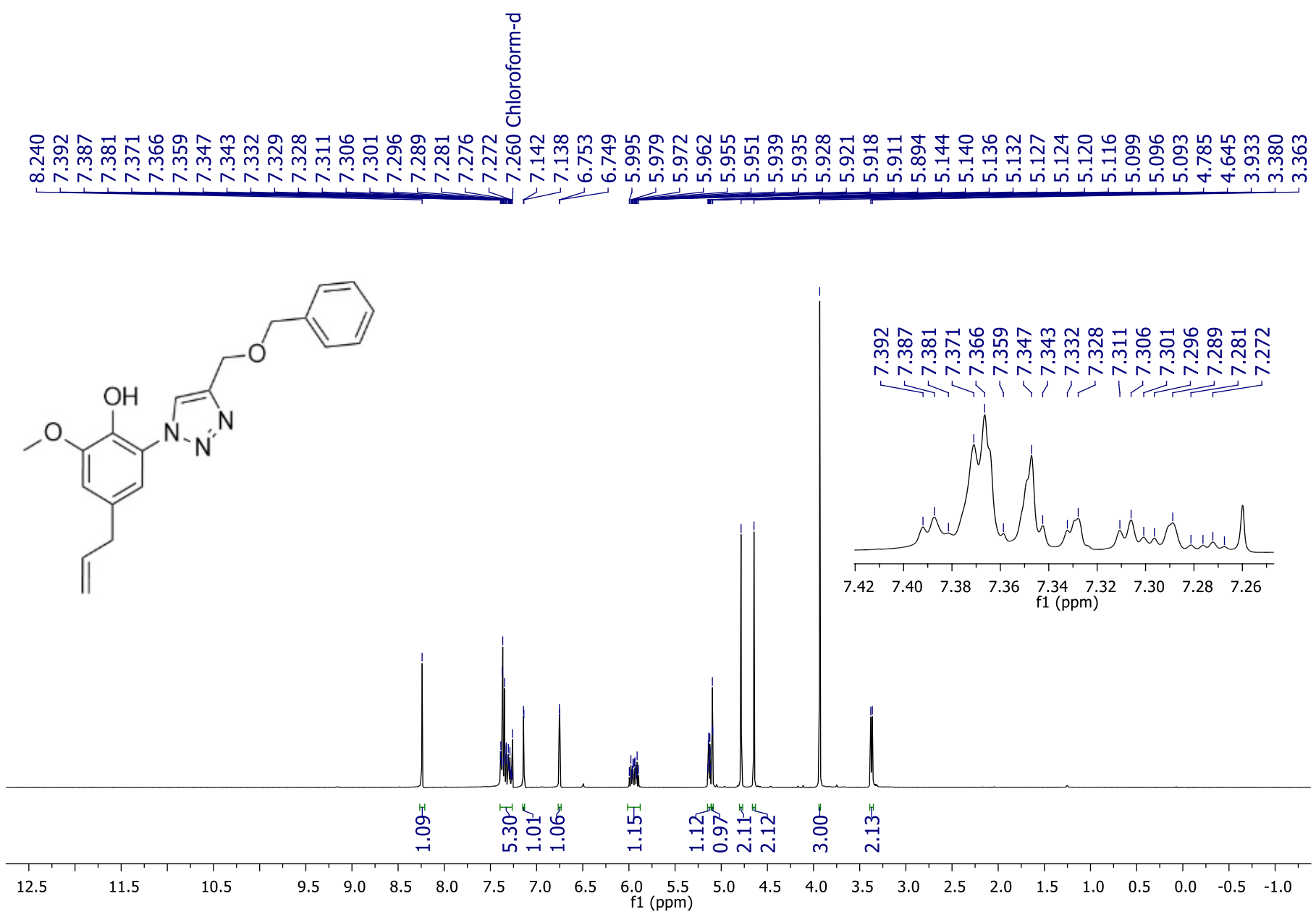

Figure S27. ^1H NMR of **4e** (400 MHz, $CDCl_3$).

^{13}C NMR of 4e (101 MHz, CDCl$_3$)

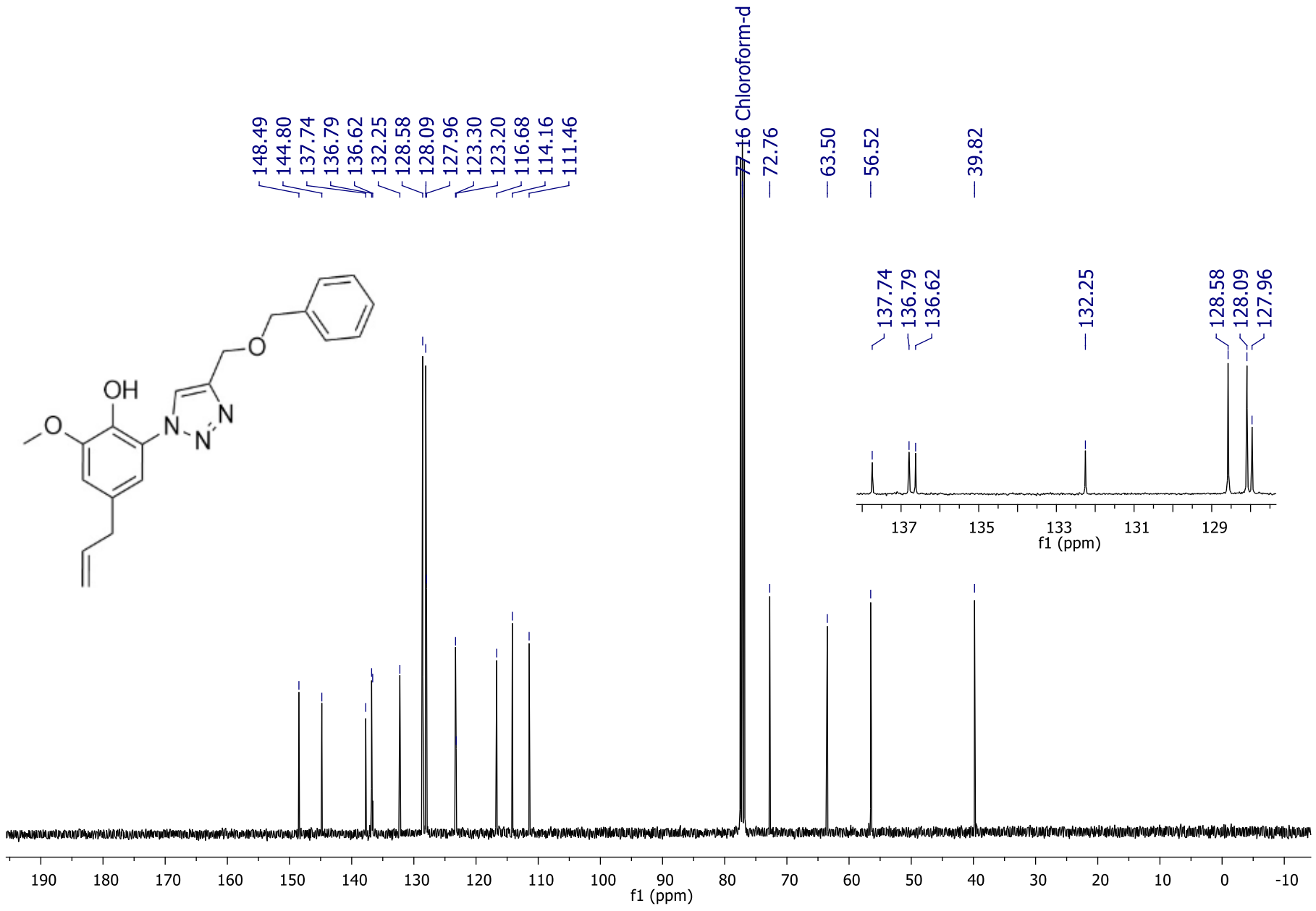

Figure S28. ^{13}C NMR of **4e** (101 MHz, CDCl$_3$).

^{13}C NMR DEPT--135 of 4e (101 MHz, CDCl$_3$)

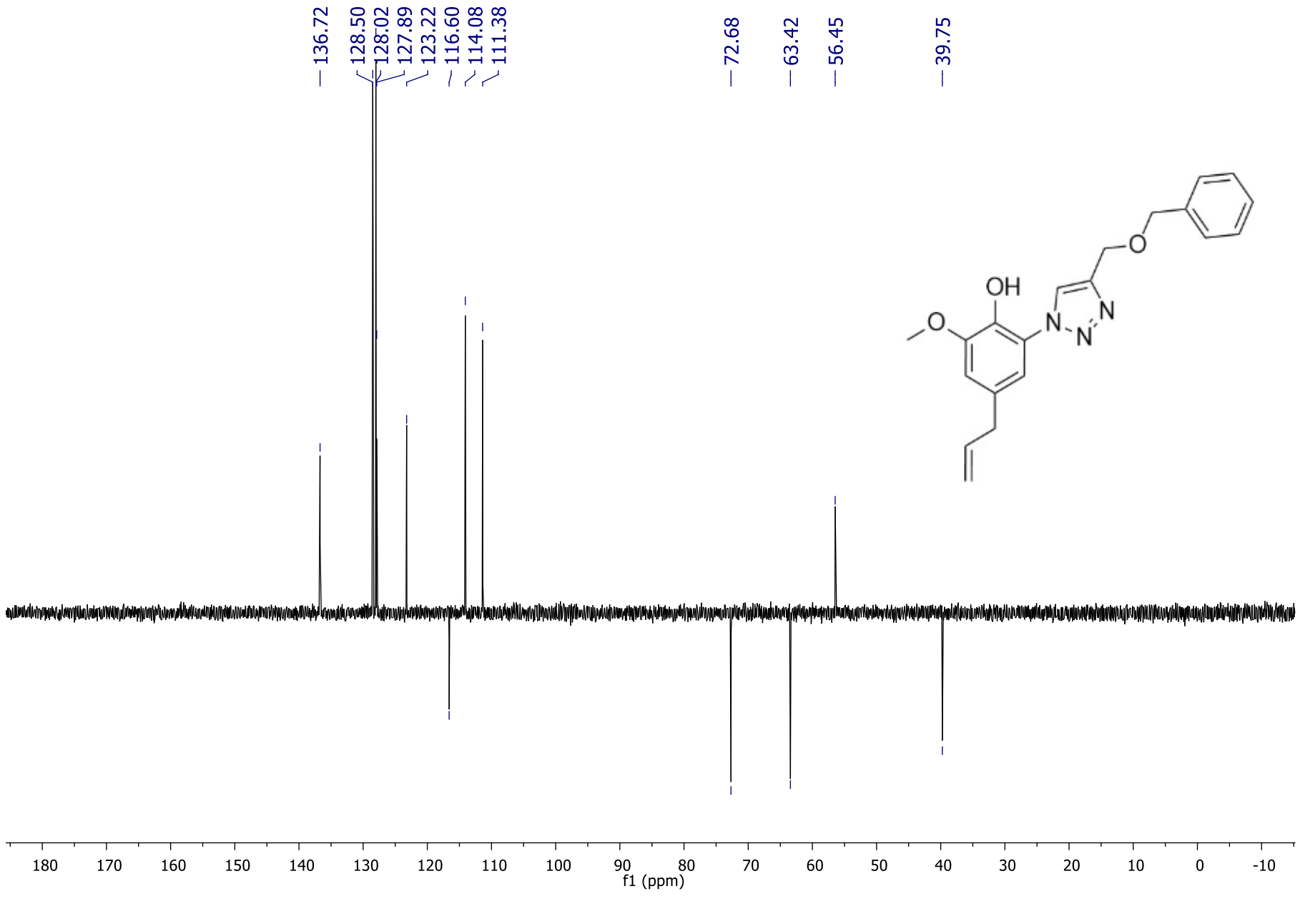

Figure S29. ^{13}C NMR DEPT--135 of **4e** (101 MHz, CDCl$_3$).

$^{1}H/^{13}C$ NMR HSQC of 4e (400 and 101 MHz, $CDCl_3$)

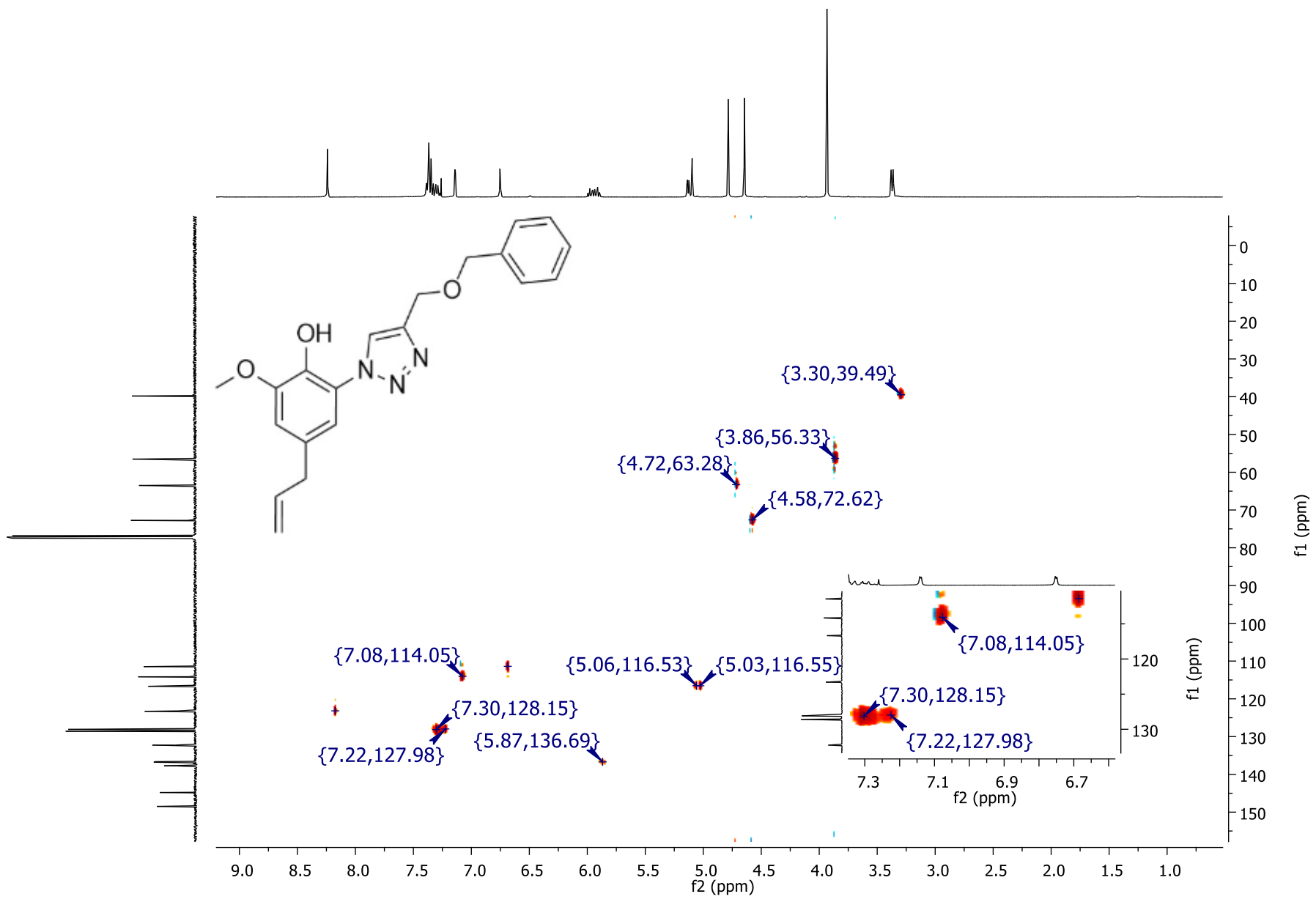

Figure S30. $^{1}H/^{13}C$ NMR HSQC of **4e** (400 and 101 MHz, $CDCl_3$).

^{1}H NMR of 4f (400 MHz, $CDCl_3$)

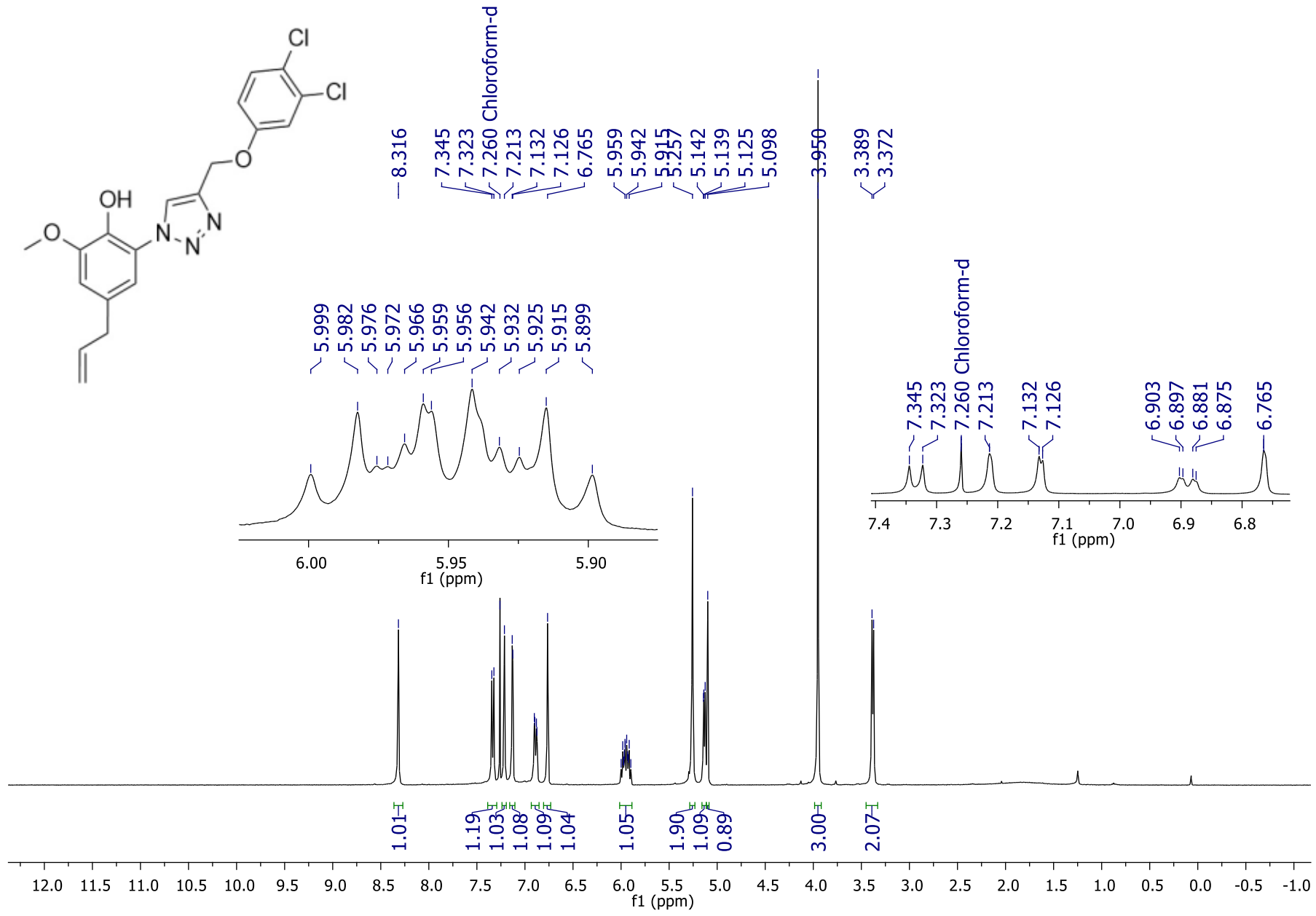

Figure S31. ^{1}H NMR of **4f** (400 MHz, $CDCl_3$).

^{13}C NMR of 4f (101 MHz, CDCl$_3$)

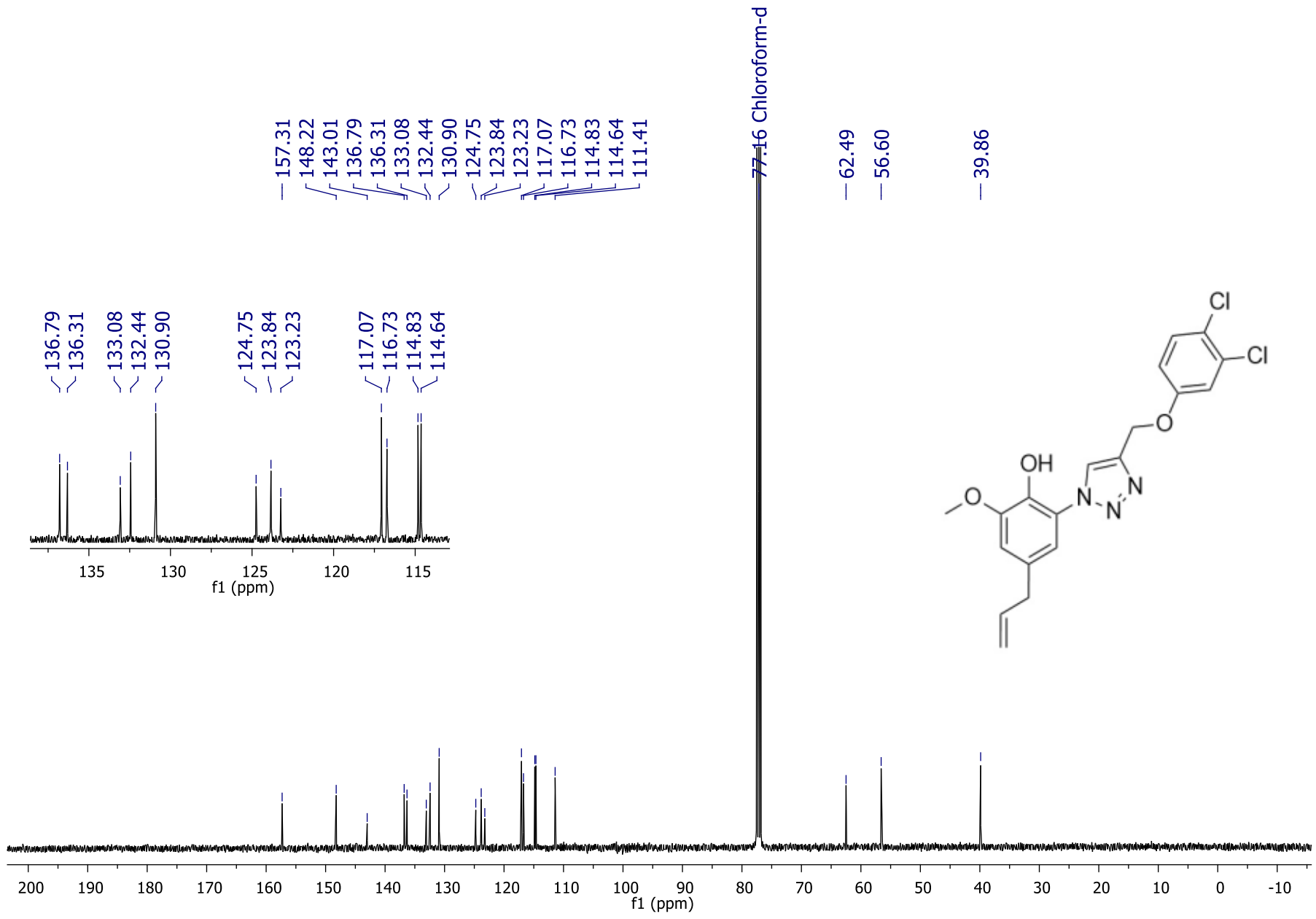

Figure S32. ^{13}C NMR of **4f** (101 MHz, CDCl$_3$).

^{13}C NMR DEPT--135 of 4f (101 MHz, CDCl$_3$)

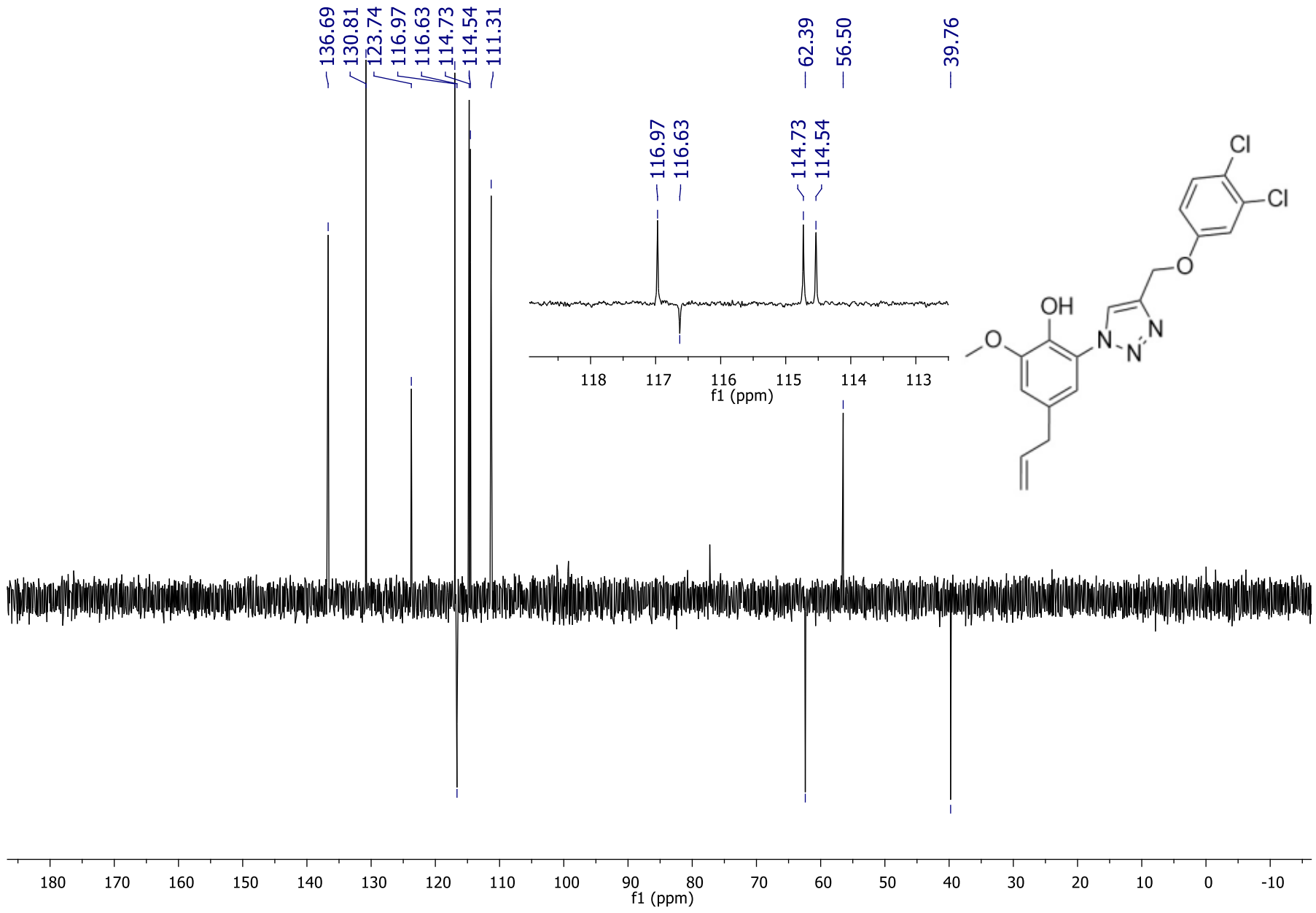

Figure S33. ^{13}C NMR DEPT--135 of **4f** (101 MHz, CDCl$_3$).

$^{1}H/^{13}C$ NMR HSQC of 4f (400 and 101 MHz, $CDCl_3$)

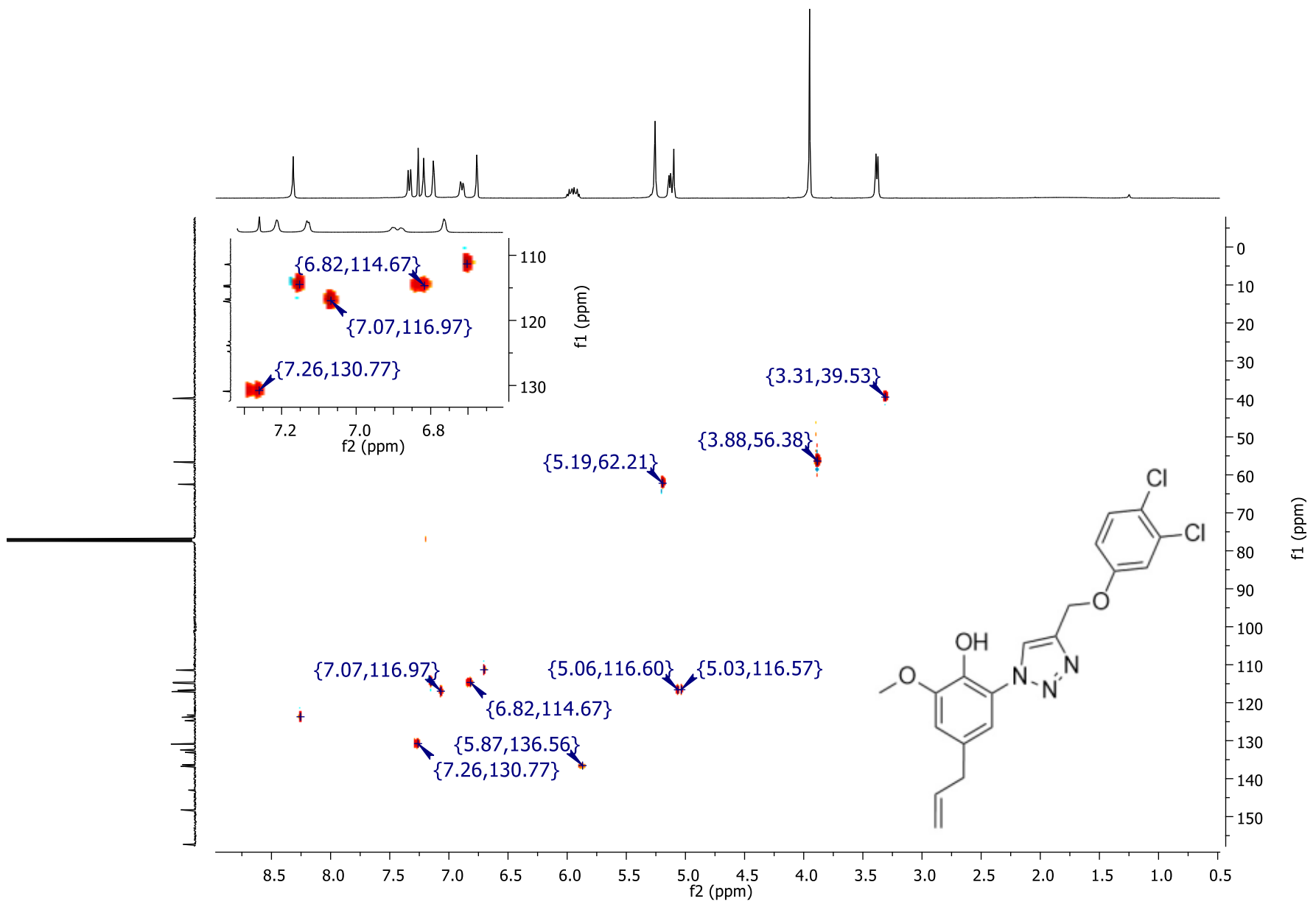

Figure S34. $^{1}H/^{13}C$ NMR HSQC of **4f** (400 and 101 MHz, $CDCl_3$).

^{1}H NMR of 4g (400 MHz, $CDCl_3$)

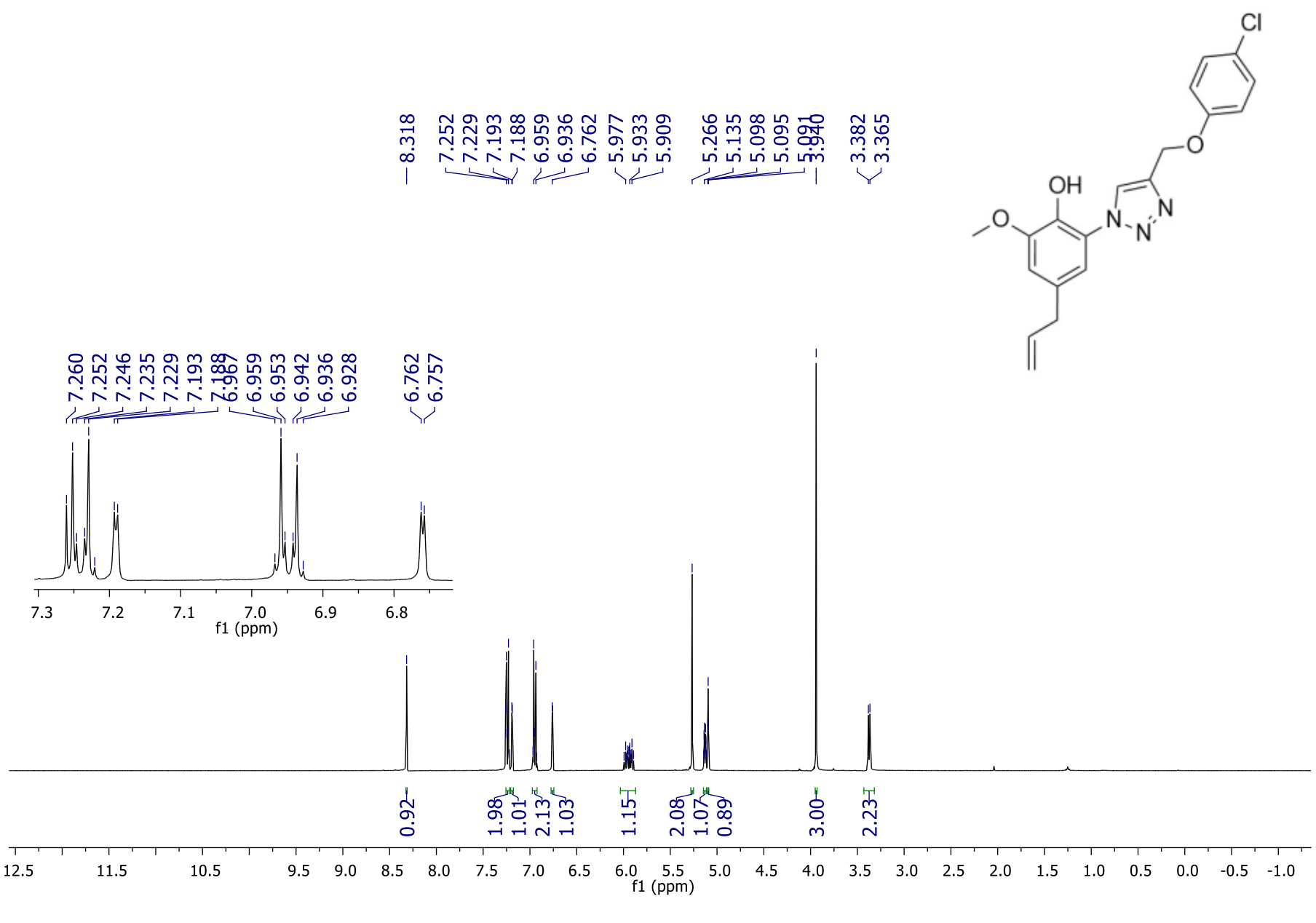

Figure S35. ^{1}H NMR of **4g** (400 MHz, $CDCl_3$).

^{13}C NMR of 4g (101 MHz, $CDCl_3$)

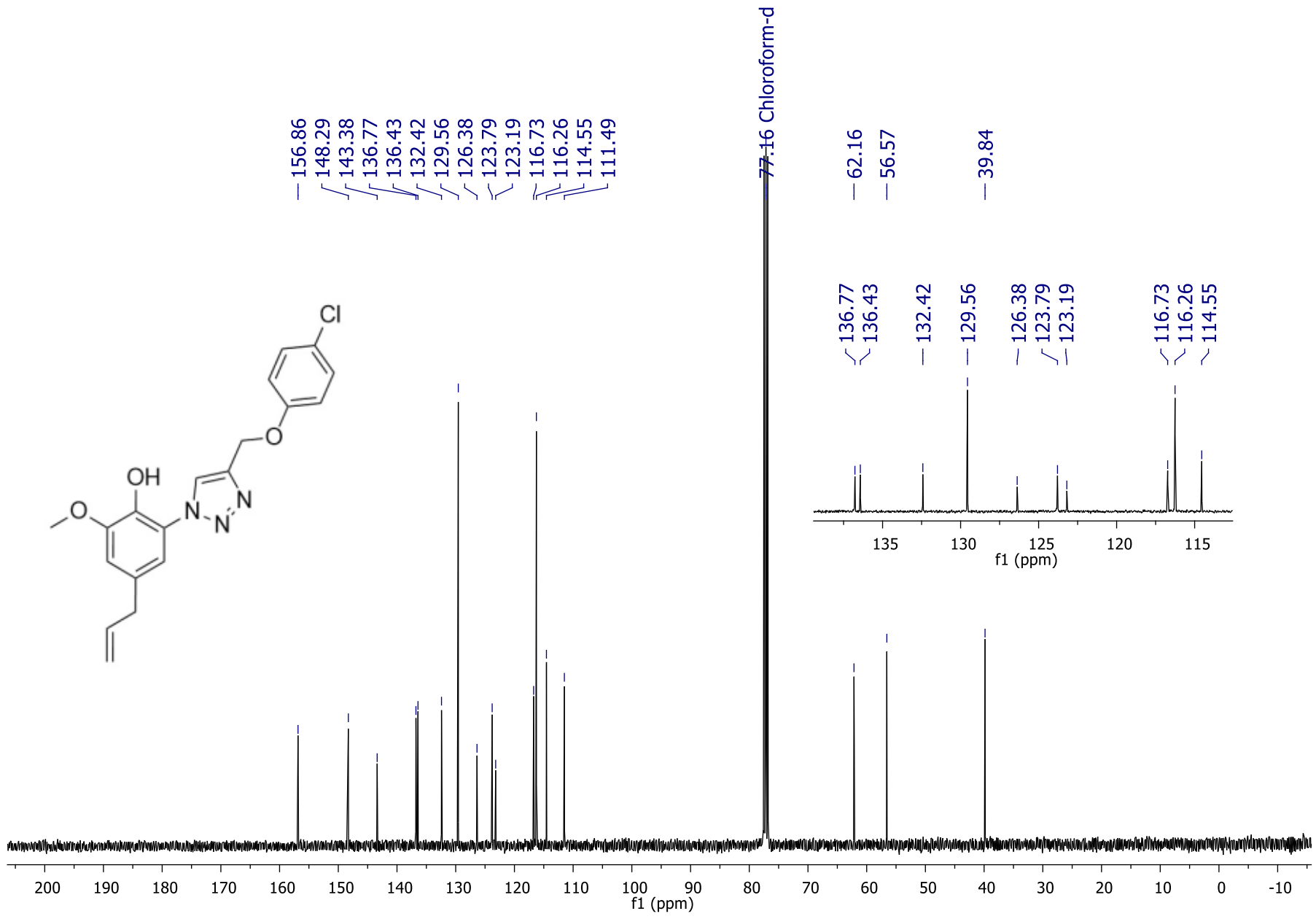

Figure S36. ^{13}C NMR of **4g** (101 MHz, $CDCl_3$).

^{13}C NMR DEPT--135 of 4g (101 MHz, $CDCl_3$)

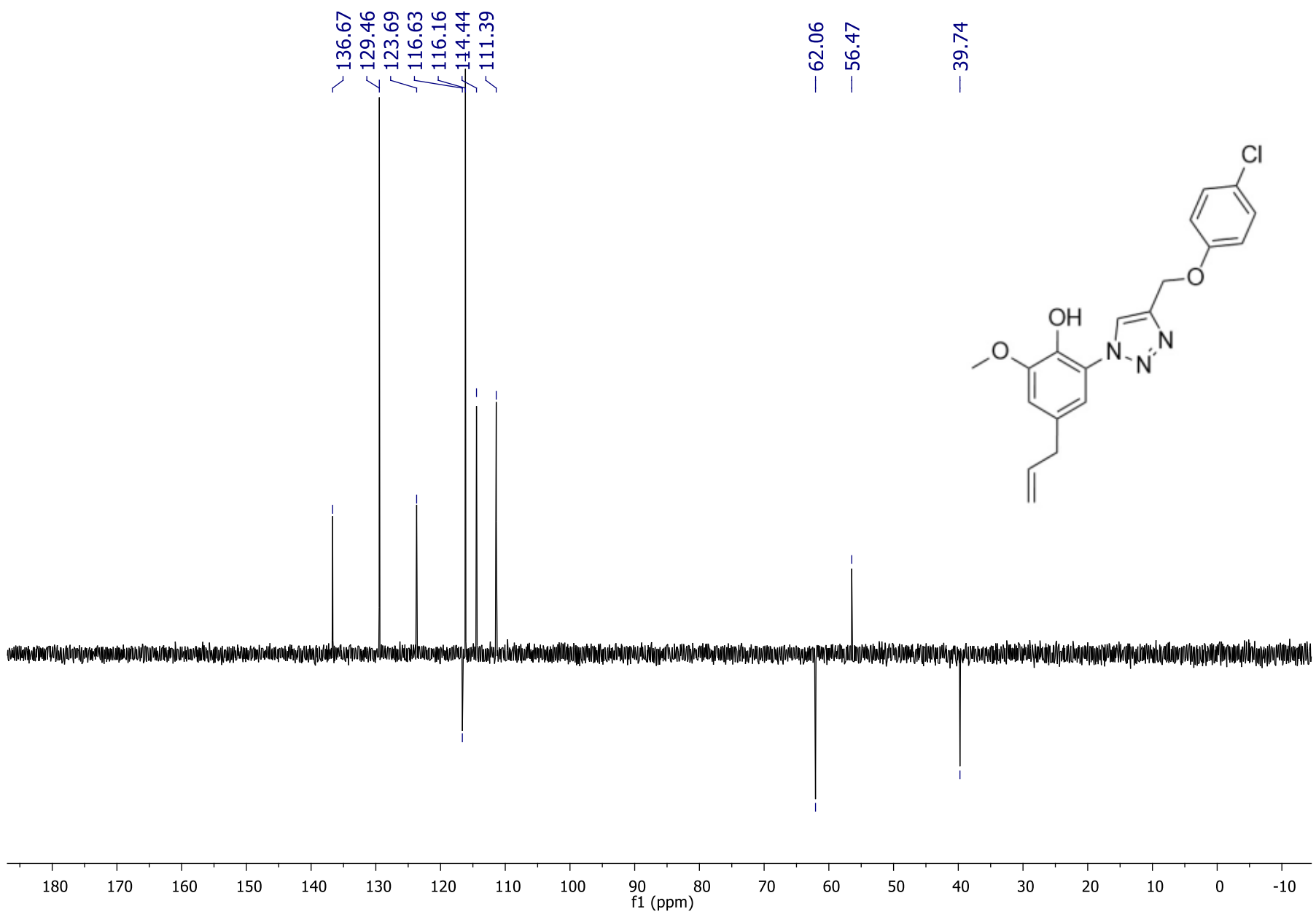

Figure S37. ^{13}C NMR DEPT--135 of **4g** (101 MHz, $CDCl_3$).

$^1H/^{13}C$ NMR HSQC of 4g (400 and 101 MHz, $CDCl_3$)

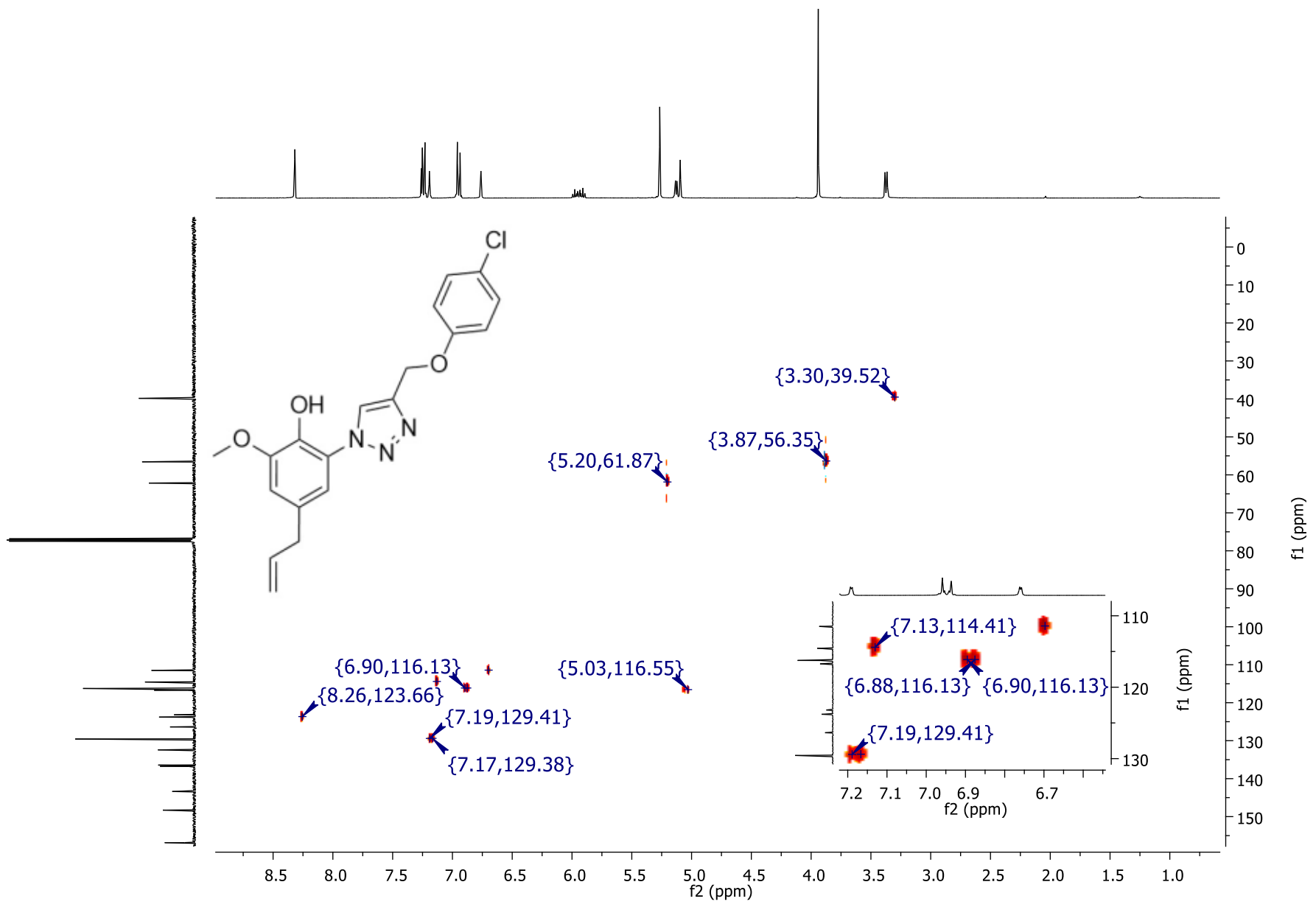

Figure S38. $^1H/^{13}C$ NMR HSQC of **4g** (400 and 101 MHz, $CDCl_3$).

^1H NMR of 4h (400 MHz, $CDCl_3$)

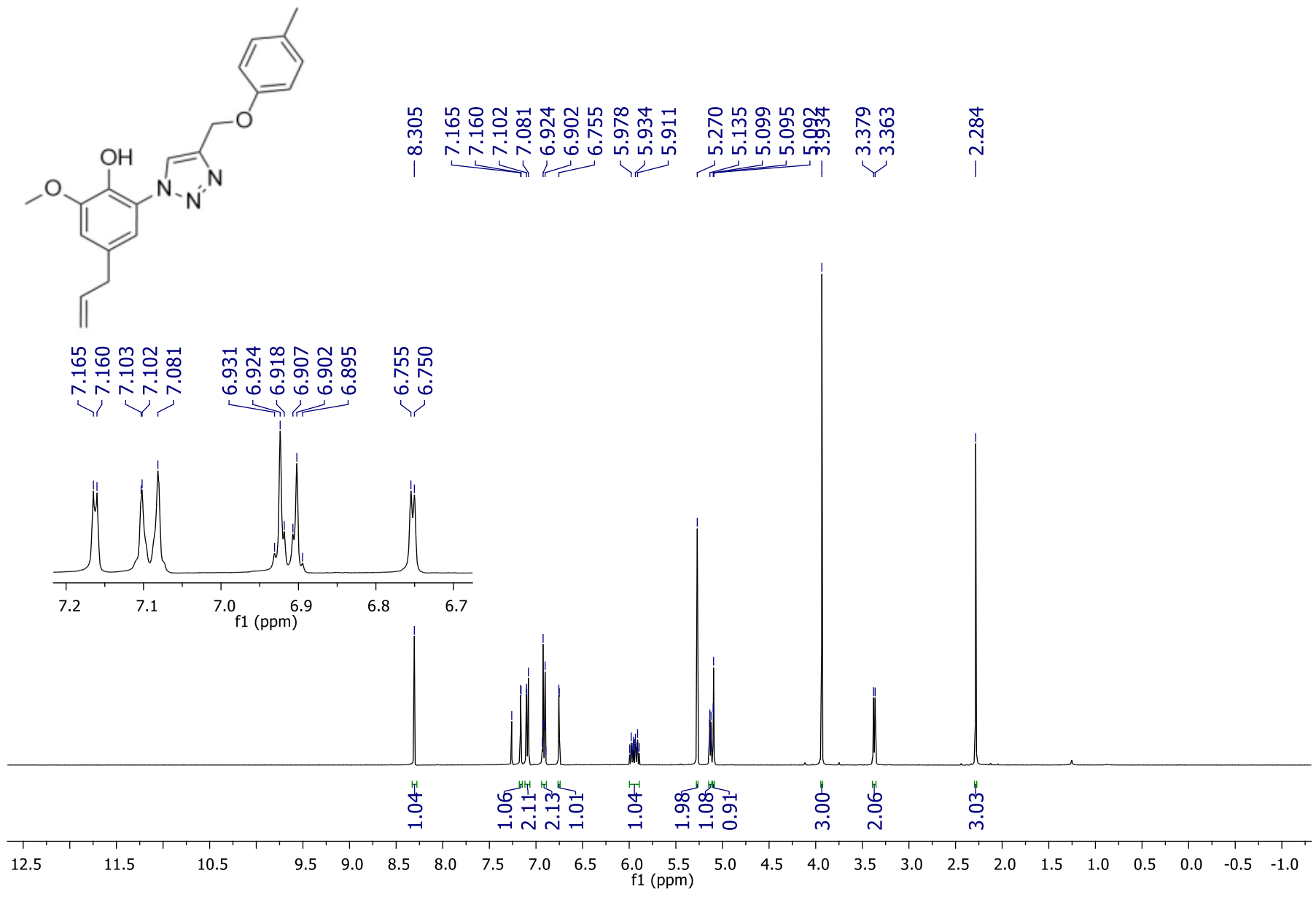

Figure S39. ^1H NMR of **4h** (400 MHz, $CDCl_3$).

^{13}C NMR of 4h (101 MHz, $CDCl_3$)

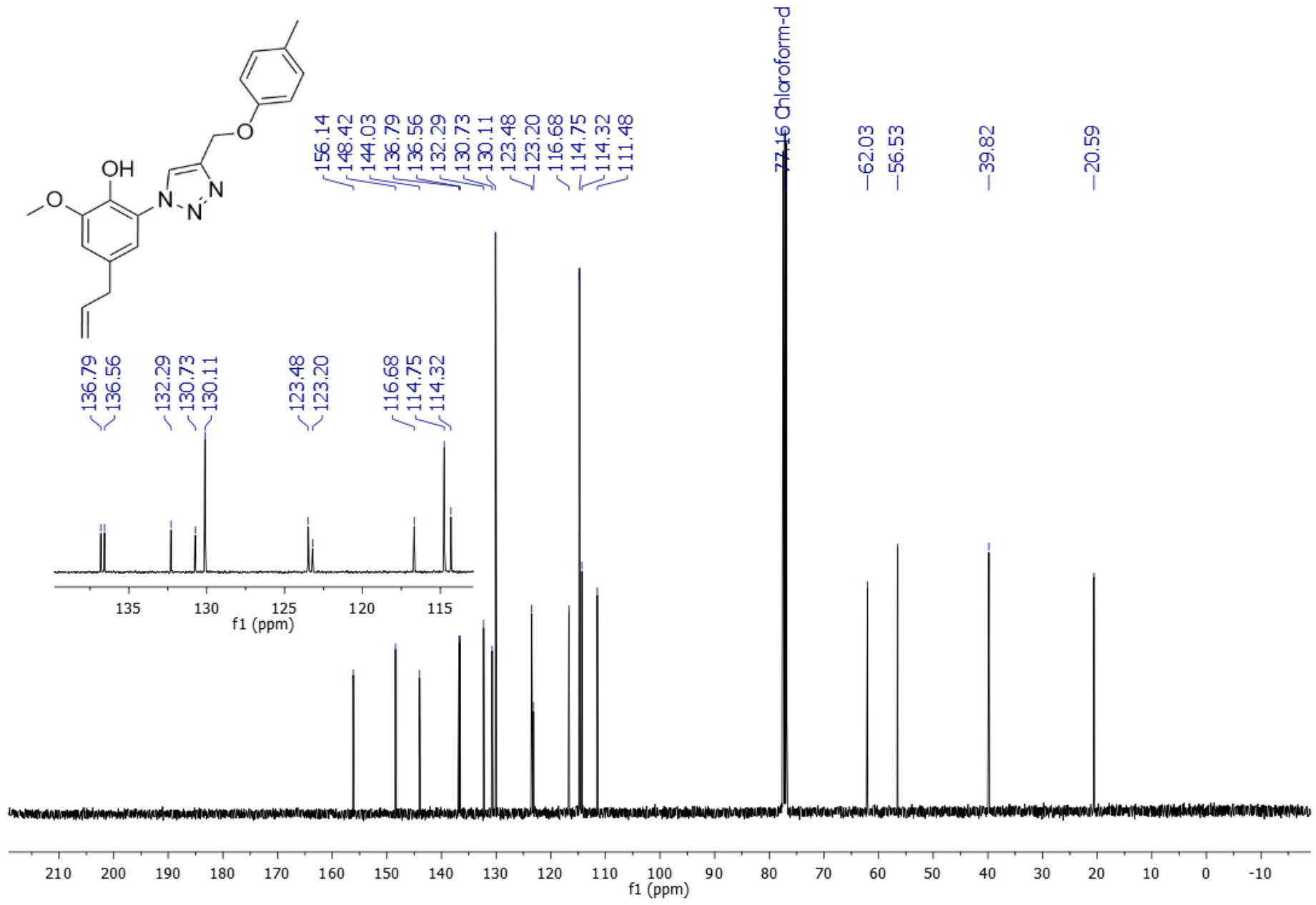

Figure S40. ^{13}C NMR of **4h** (101 MHz, $CDCl_3$).

^{13}C NMR DEPT--135 of 4h (101 MHz, $CDCl_3$)

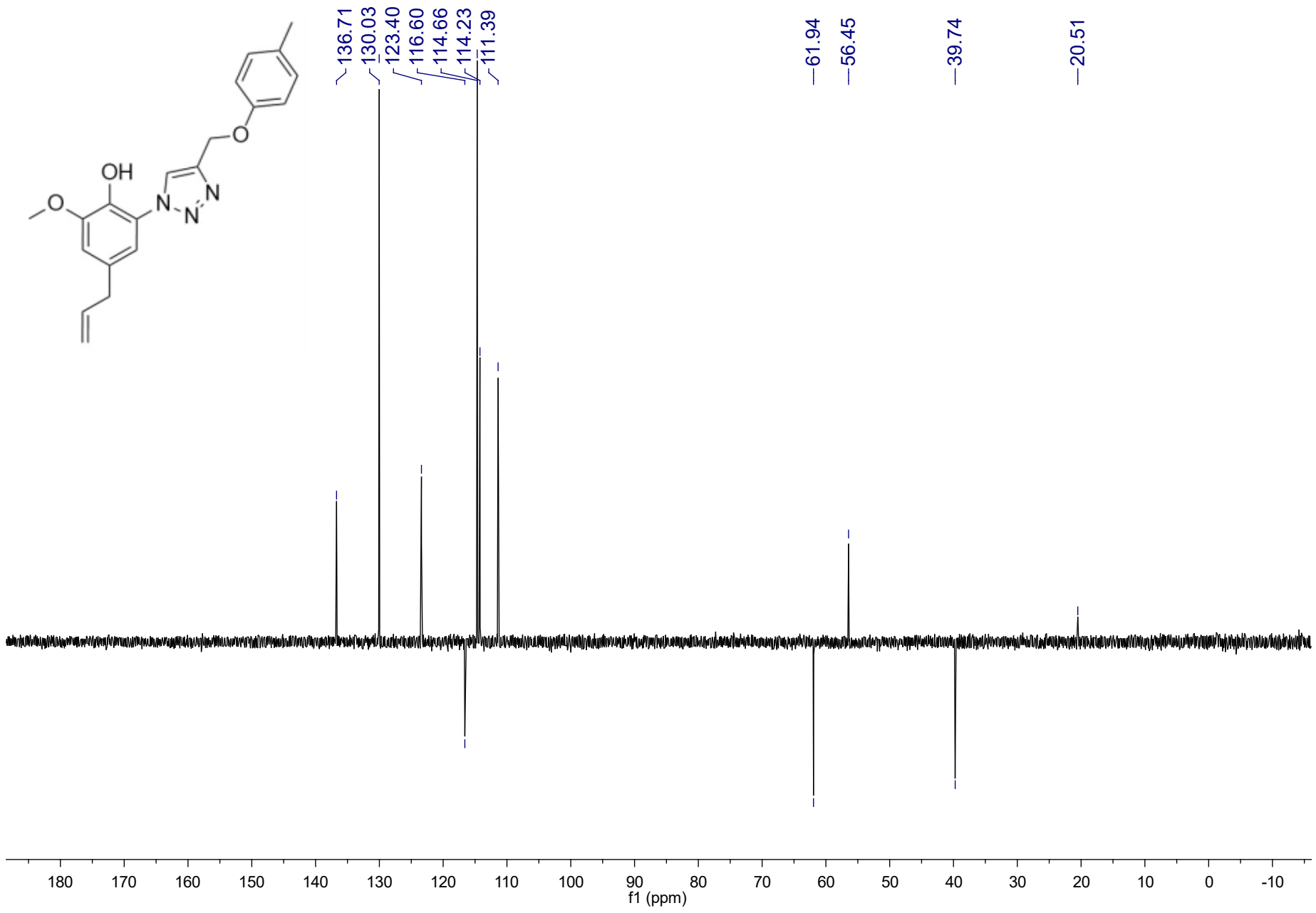

Figure S41. ^{13}C NMR DEPT--135 of **4h** (101 MHz, $CDCl_3$).

$^{1}H/^{13}C$ NMR HSQC of 4h (400 and 101 MHz, $CDCl_3$)

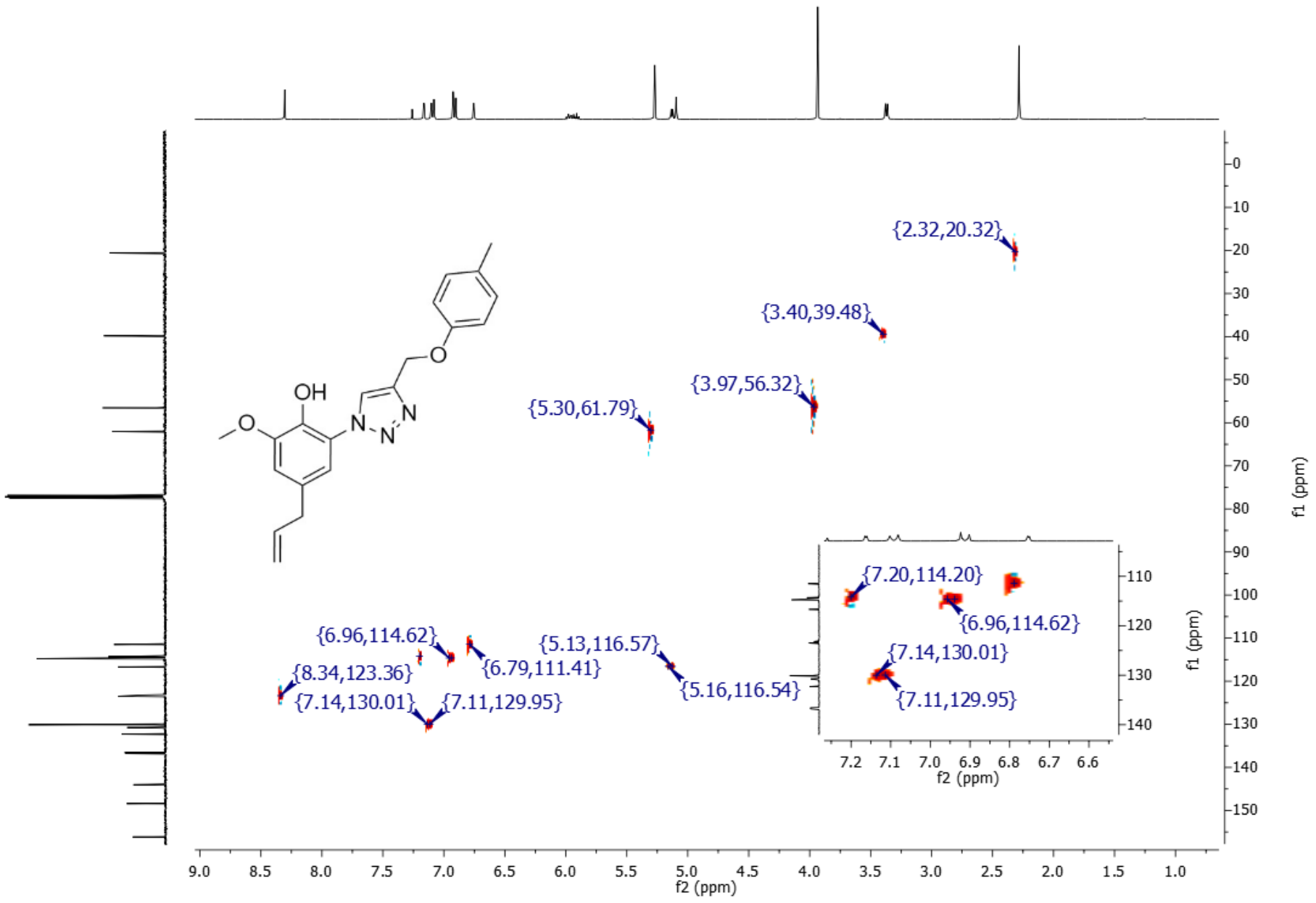

Figure S42. $^{1}H/^{13}C$ NMR HSQC of **4h** (400 and 101 MHz, $CDCl_3$).

^{1}H NMR of 4i (400 MHz, $CDCl_3$)

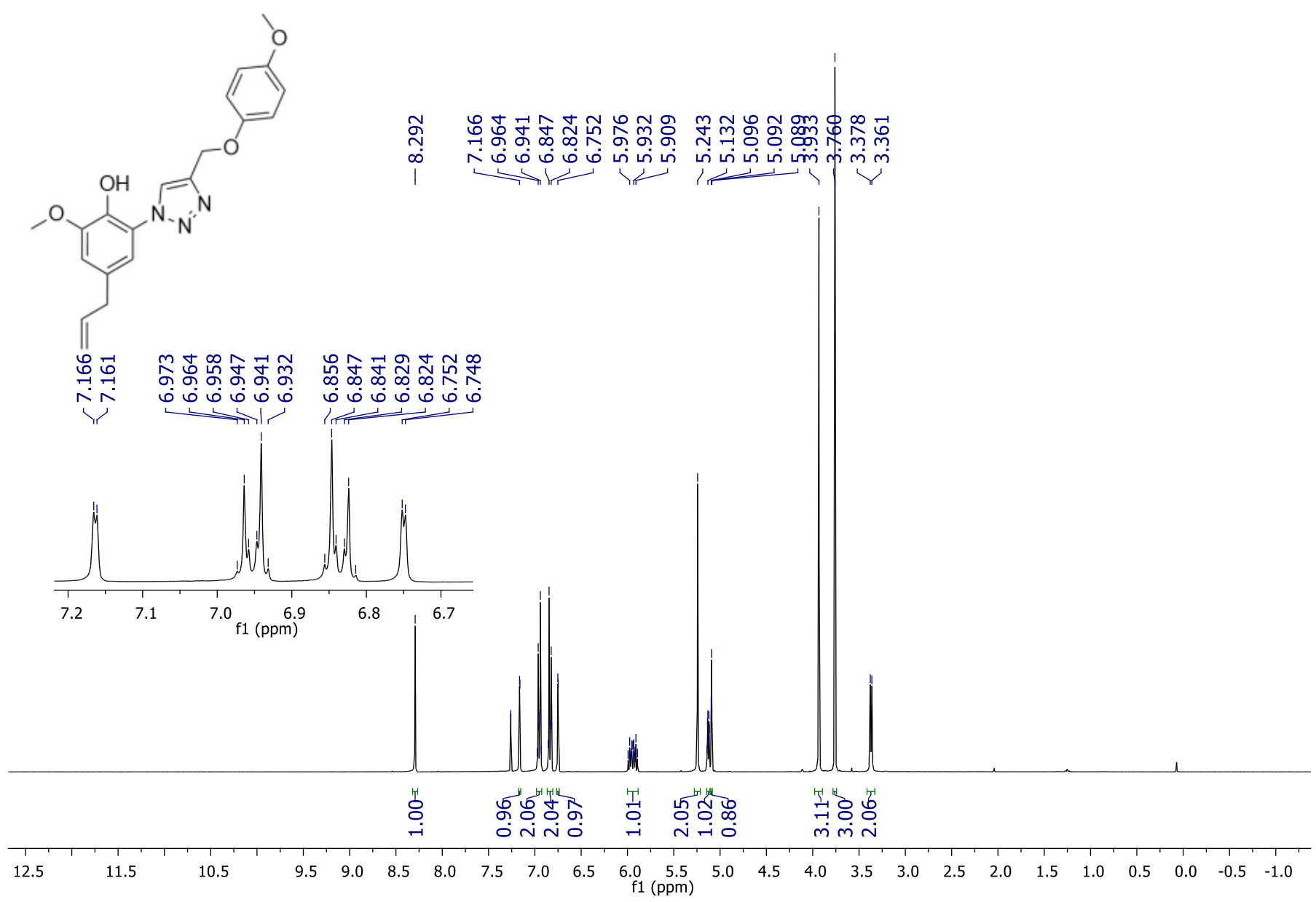

Figure S43. ^{1}H NMR of **4i** (400 MHz, $CDCl_3$).

^{13}C NMR of 4i (101 MHz, CDCl$_3$)

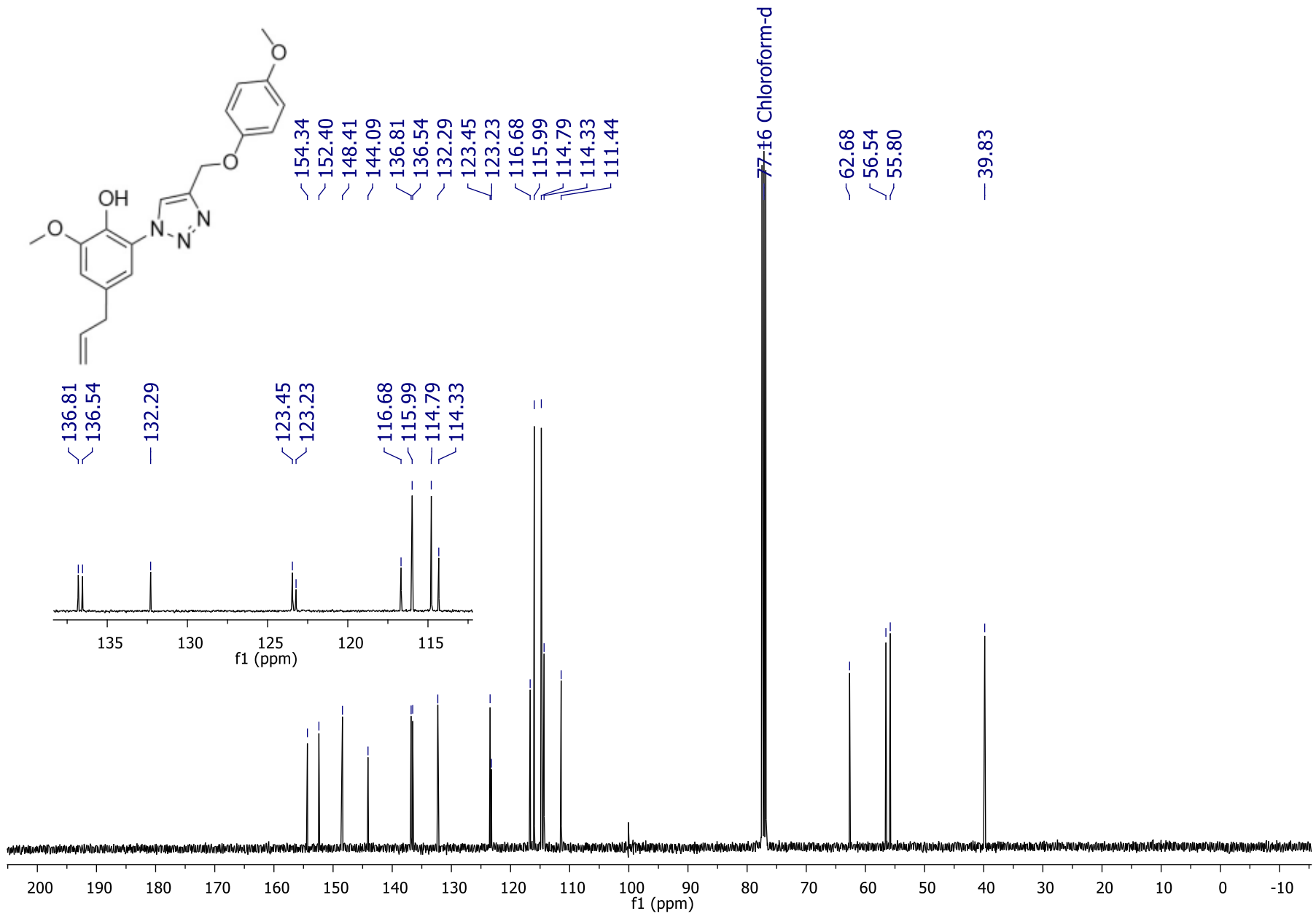

Figure S44. ^{13}C NMR of **4i** (101 MHz, CDCl$_3$).

^{13}C NMR DEPT--135 of 4i (101 MHz, CDCl$_3$)

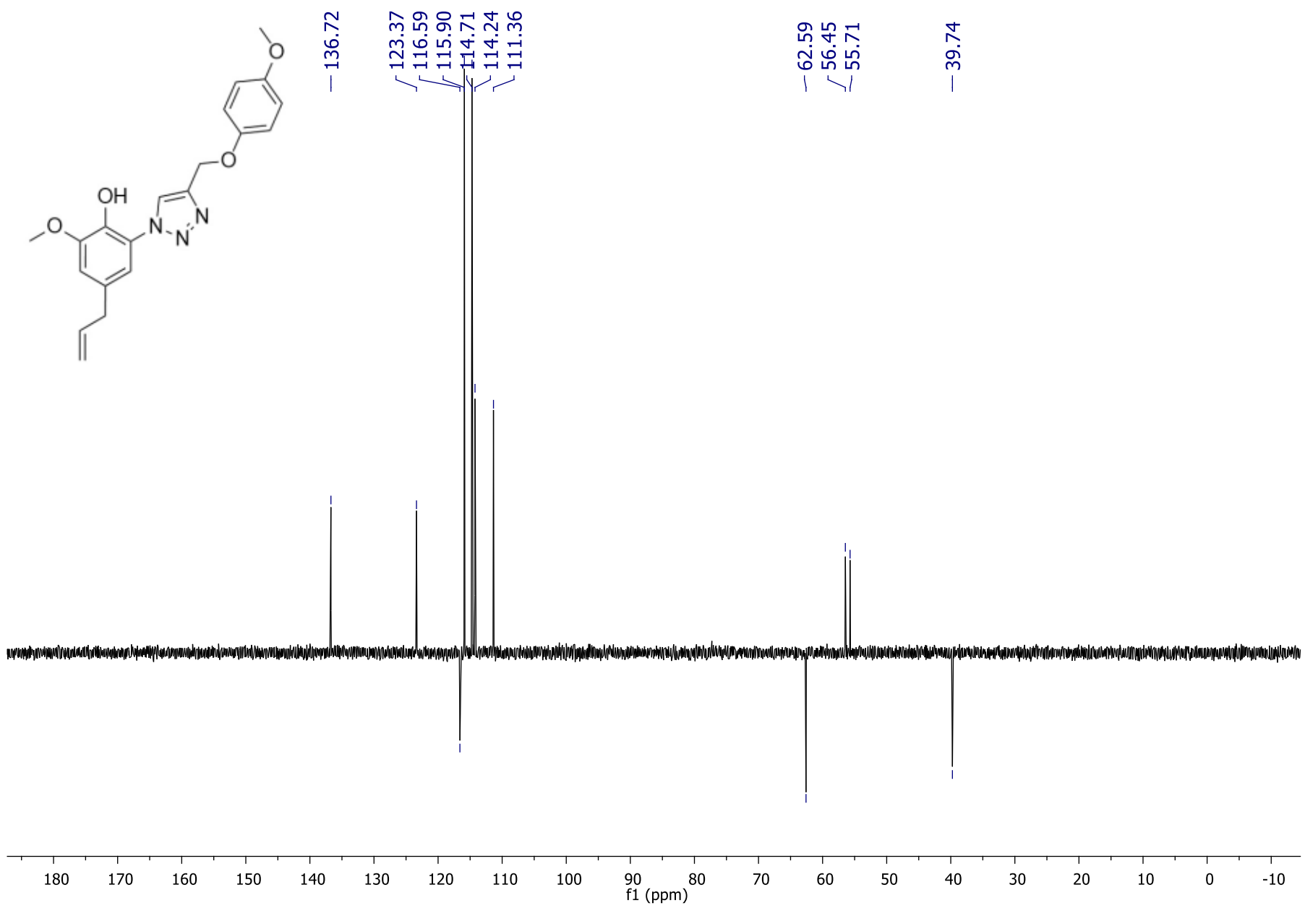

Figure S45. ^{13}C NMR DEPT--135 of **4i** (101 MHz, CDCl$_3$).

$^{1}H/^{13}C$ NMR HSQC of 4i (400 and 101 MHz, $CDCl_3$)

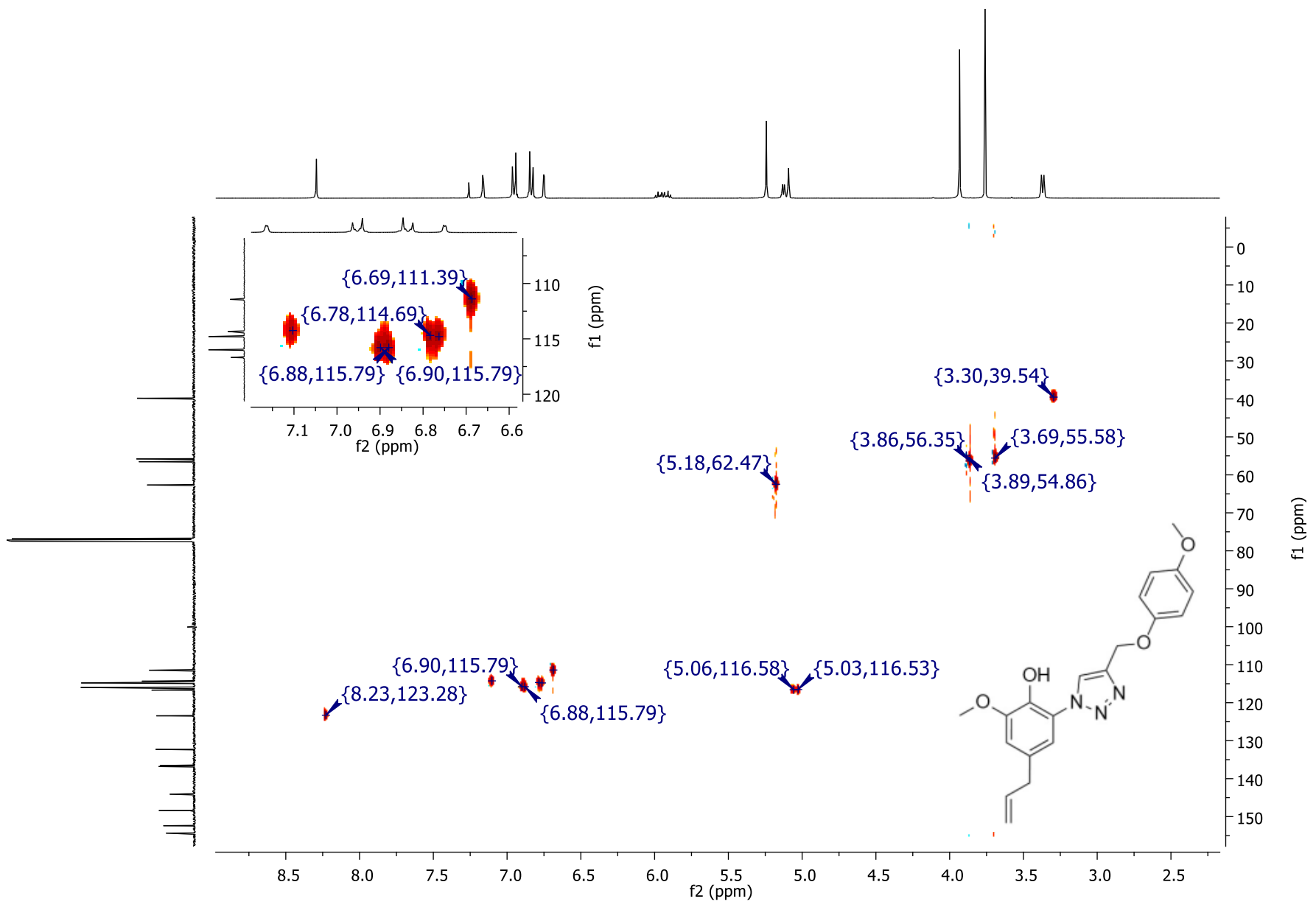

Figure S46. $^{1}H/^{13}C$ NMR HSQC of **4i** (400 and 101 MHz, $CDCl_3$).

^{1}H NMR of 4j (400 MHz, $CDCl_3$)

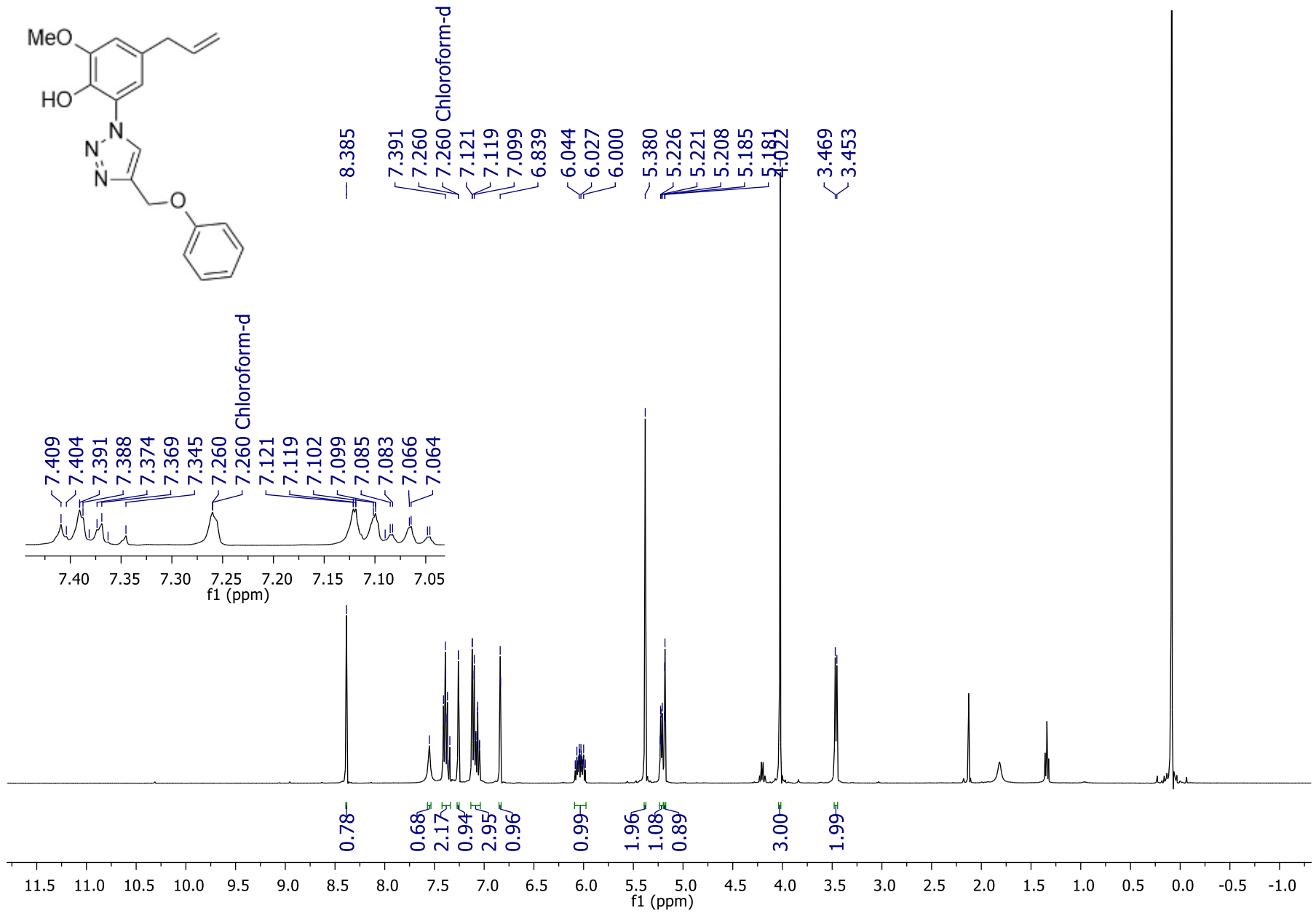

Figure S47. ^{1}H NMR of **4j** (400 MHz, $CDCl_3$).

^{13}C NMR of 4j (101 MHz, $CDCl_3$)

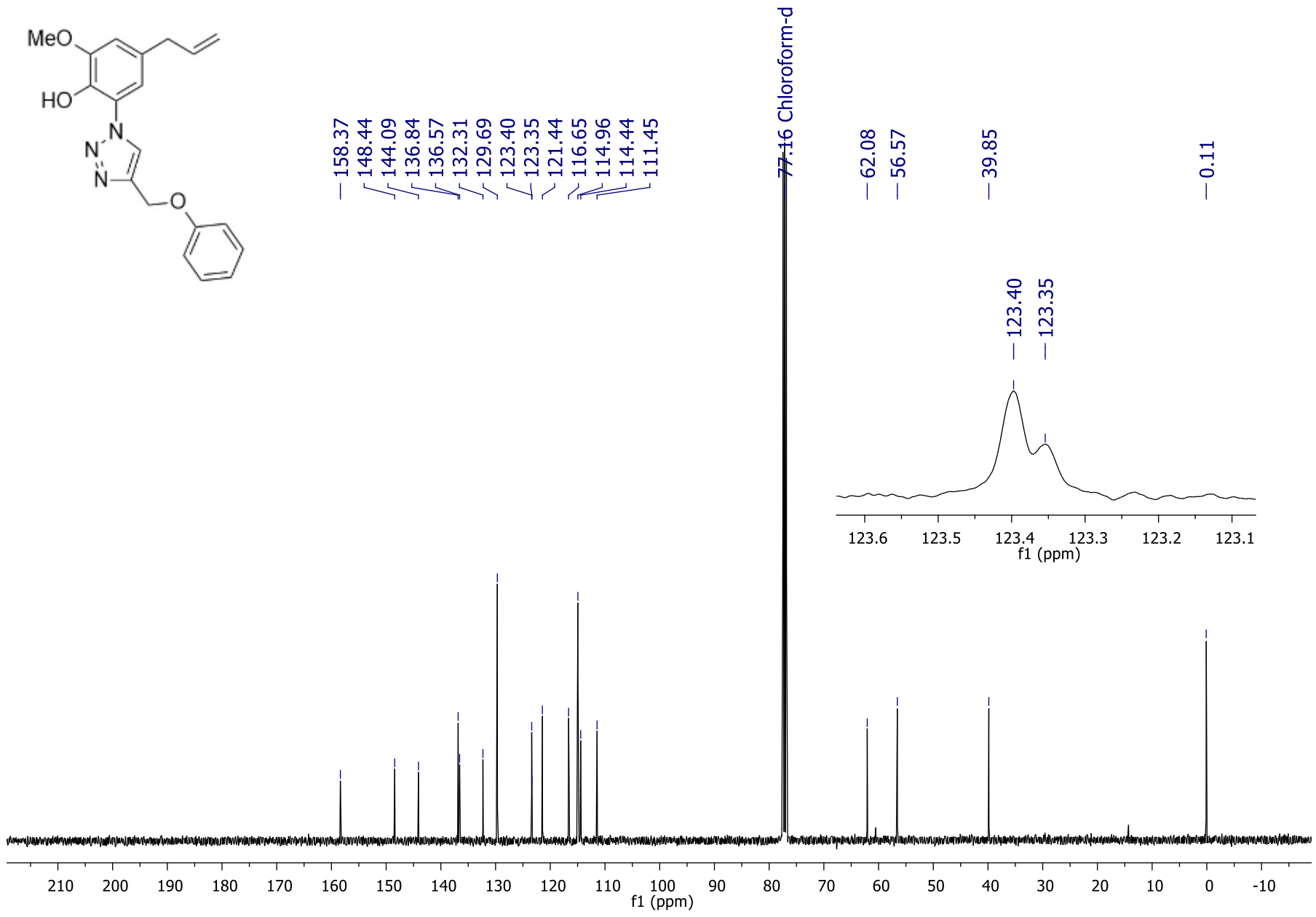

Figure S48. ^{13}C NMR of **4j** (101 MHz, $CDCl_3$).

^{13}C NMR DEPT--135 of 4j (101 MHz, $CDCl_3$)

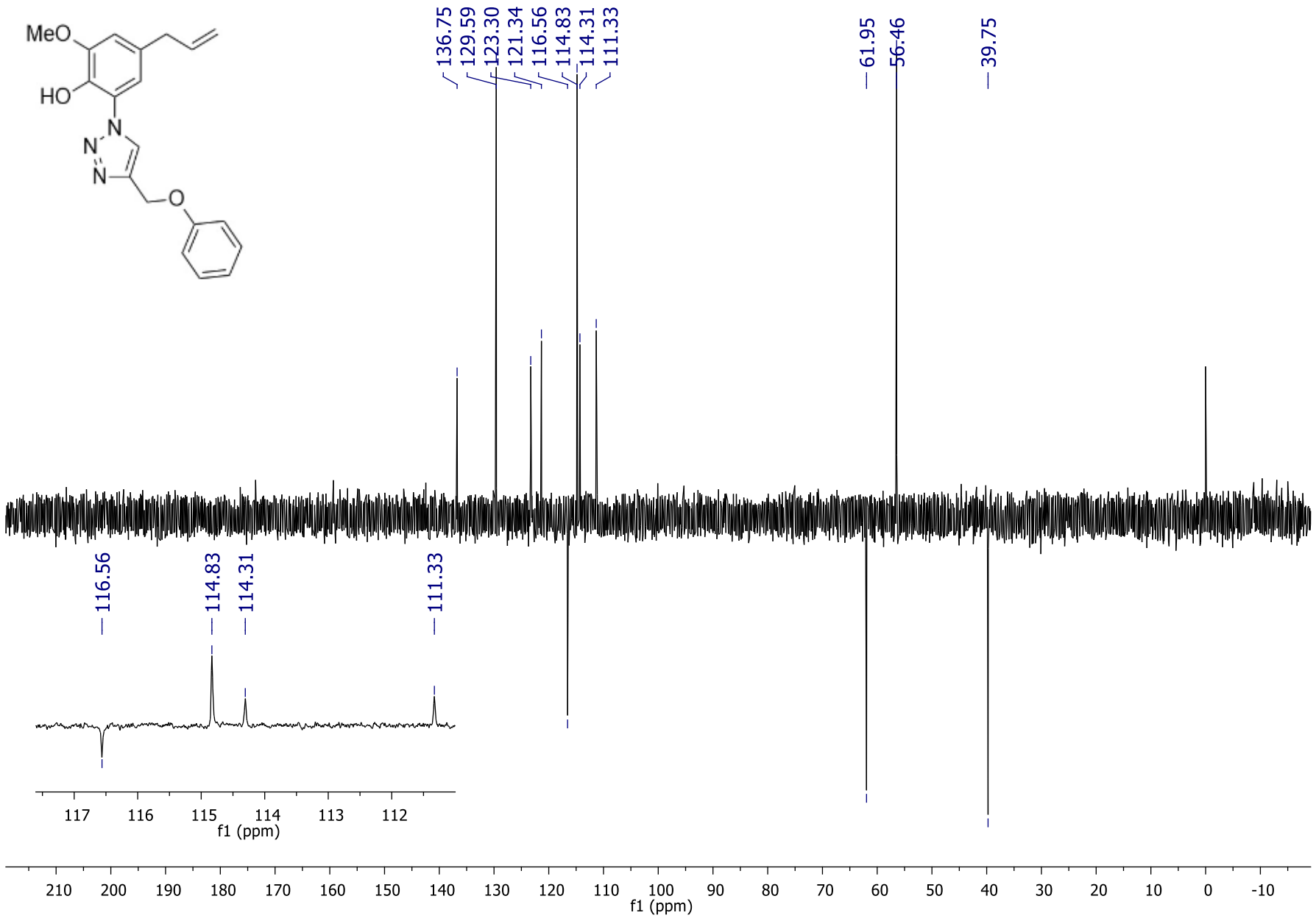

Figure S49. ^{13}C NMR DEPT--135 of **4j** (101 MHz, $CDCl_3$).

$^{1}H/^{13}C$ NMR HSQC of 4j (400 and 101 MHz, $CDCl_3$)

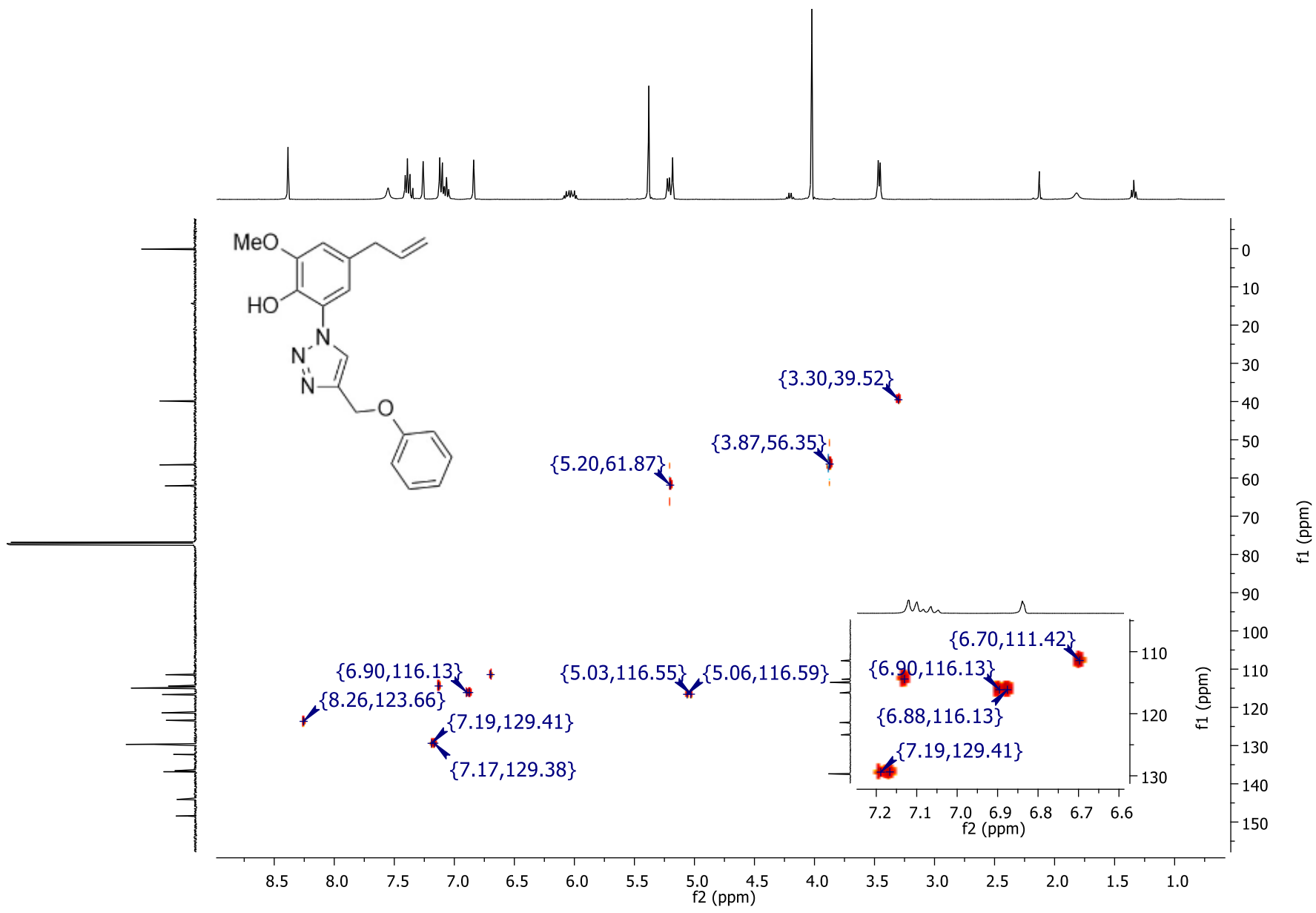

Figure S50. $^{1}H/^{13}C$ NMR HSQC of **4j** (400 and 101 MHz, $CDCl_3$).

^{1}H NMR of 4k (400 MHz, $CDCl_3$)

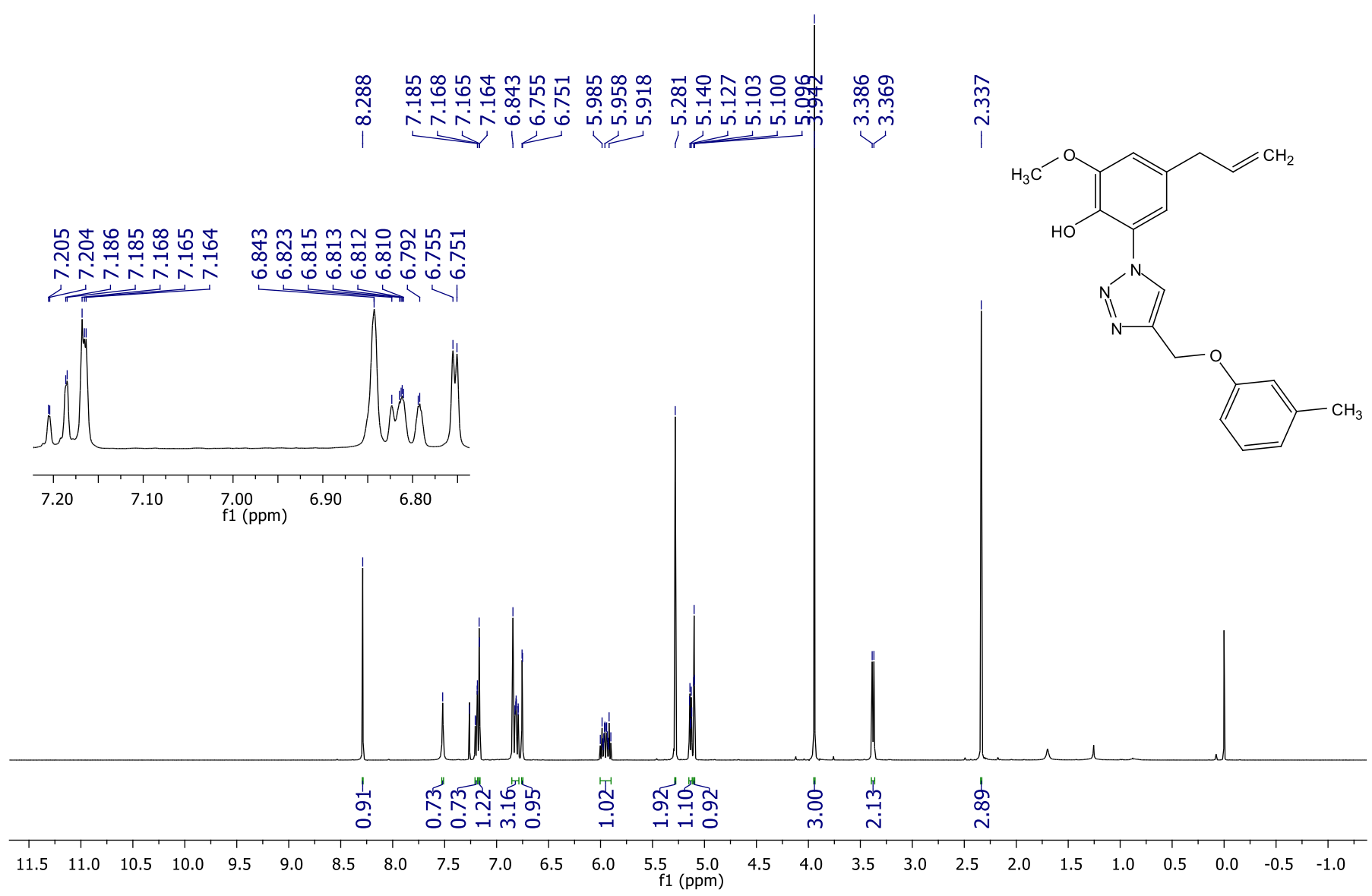

Figure S51. ^{1}H NMR of **4k** (400 MHz, $CDCl_3$).

^{13}C NMR of 4k (101 MHz, CDCl$_3$)

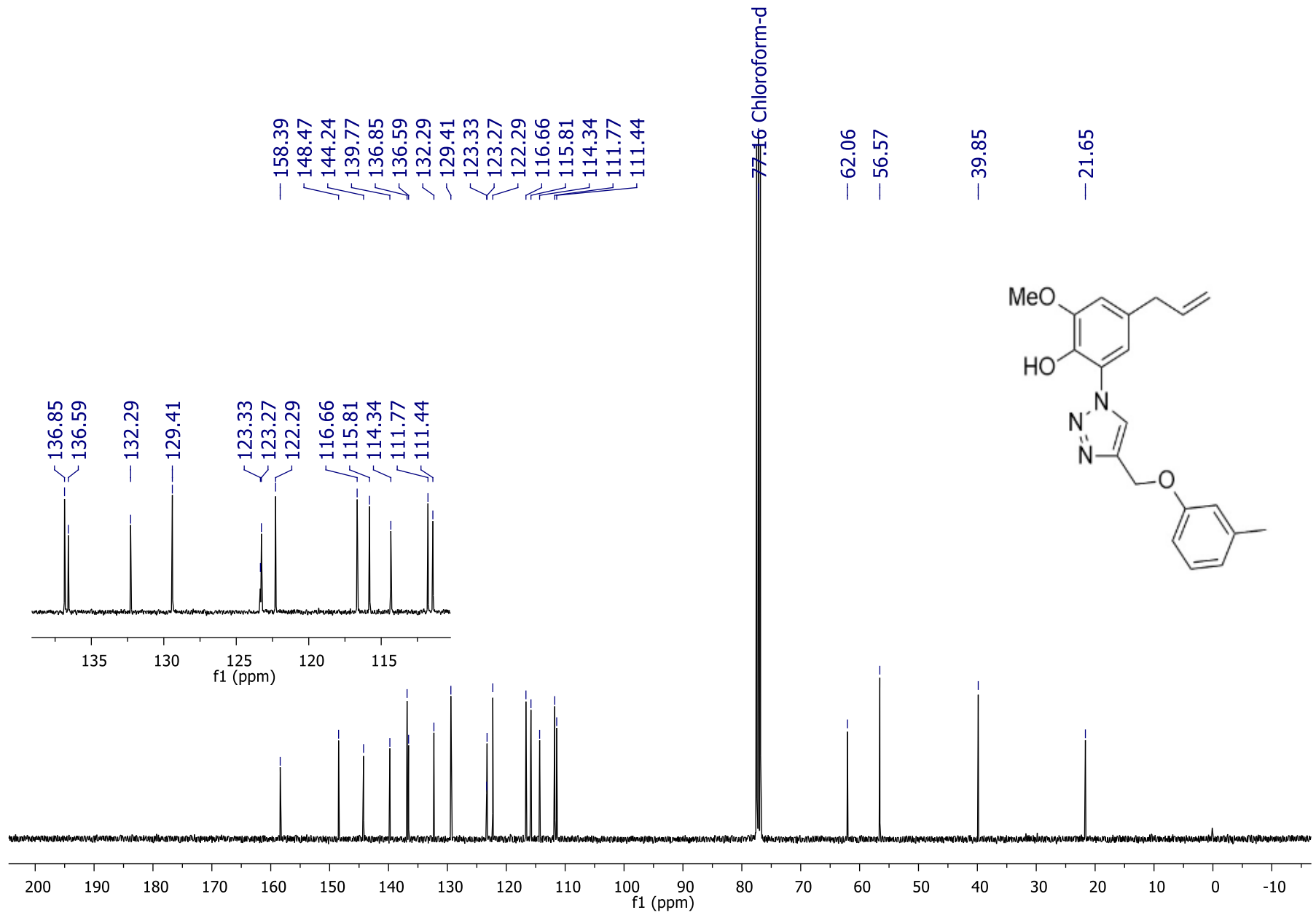

Figure S52. ^{13}C NMR of **4k** (101 MHz, CDCl$_3$).

^{13}C NMR DEPT--135 of 4k (101 MHz, CDCl$_3$)

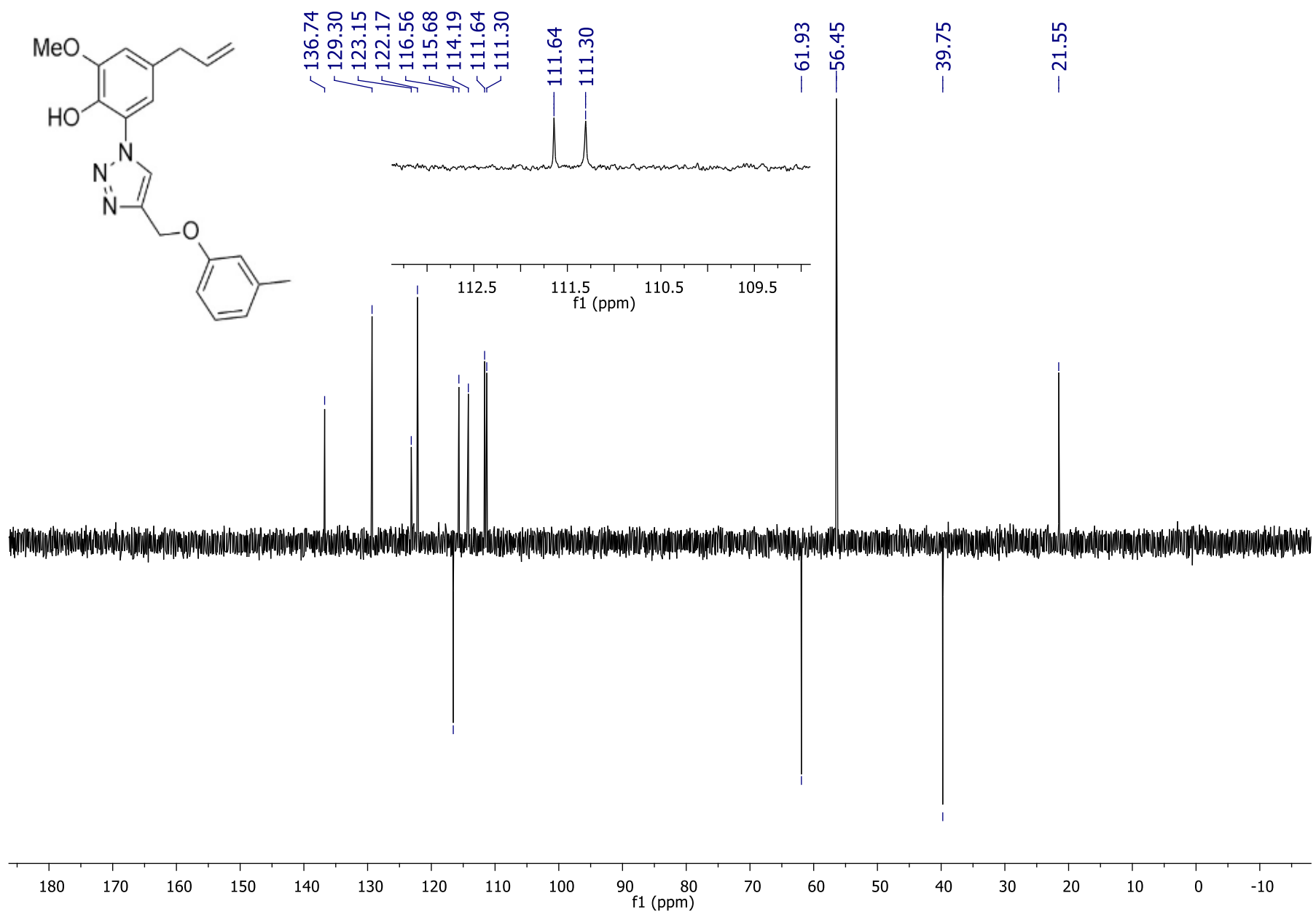

Figure S53. ^{13}C NMR DEPT--135 of **4k** (101 MHz, CDCl$_3$).

$^{1}H/^{13}C$ NMR HSQC of 4k (400 and 101 MHz, $CDCl_3$)

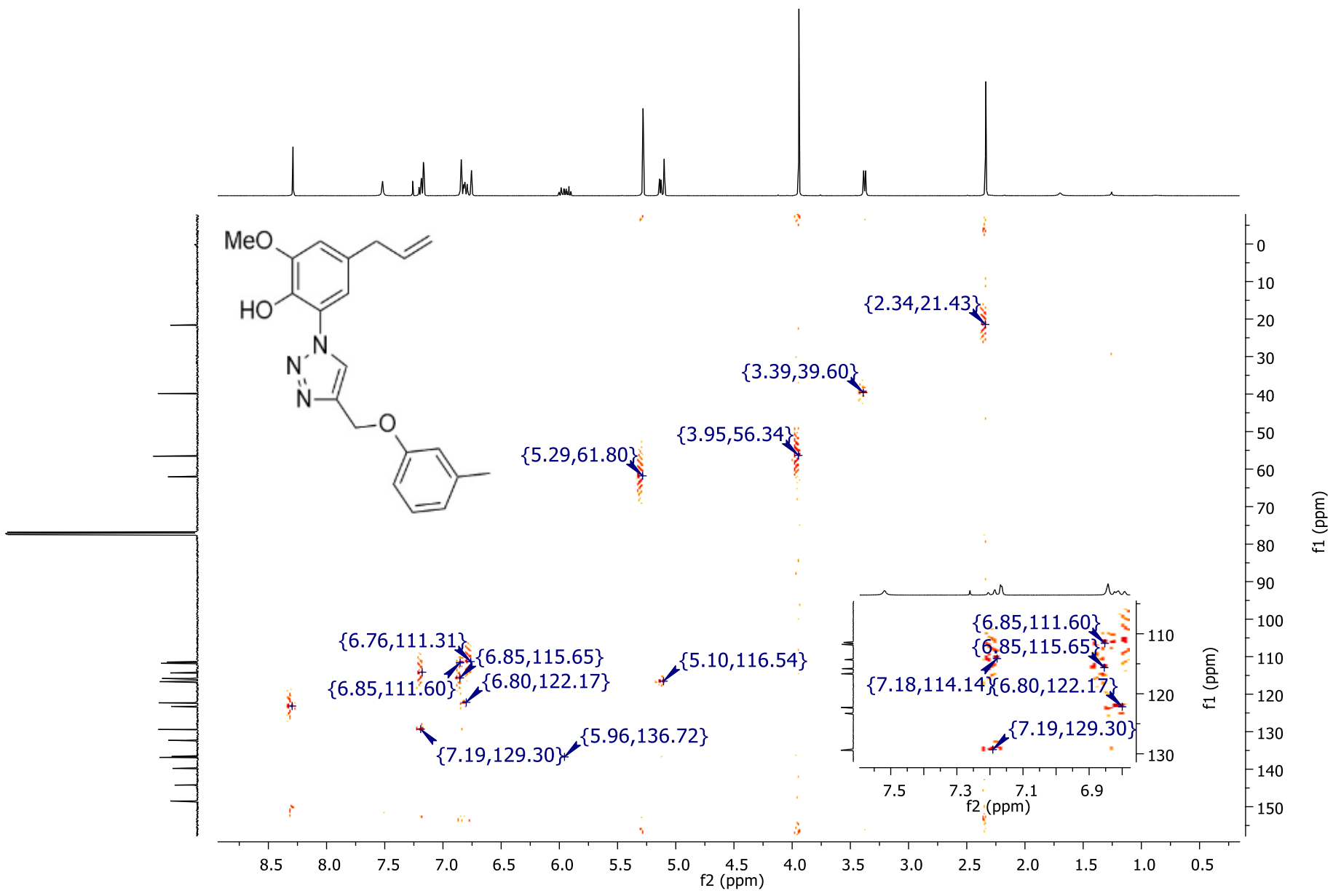

Figure S54. $^{1}H/^{13}C$ NMR HSQC of **4k** (400 and 101 MHz, $CDCl_3$).

^{1}H NMR of 4l (400 MHz, $CDCl_3$)

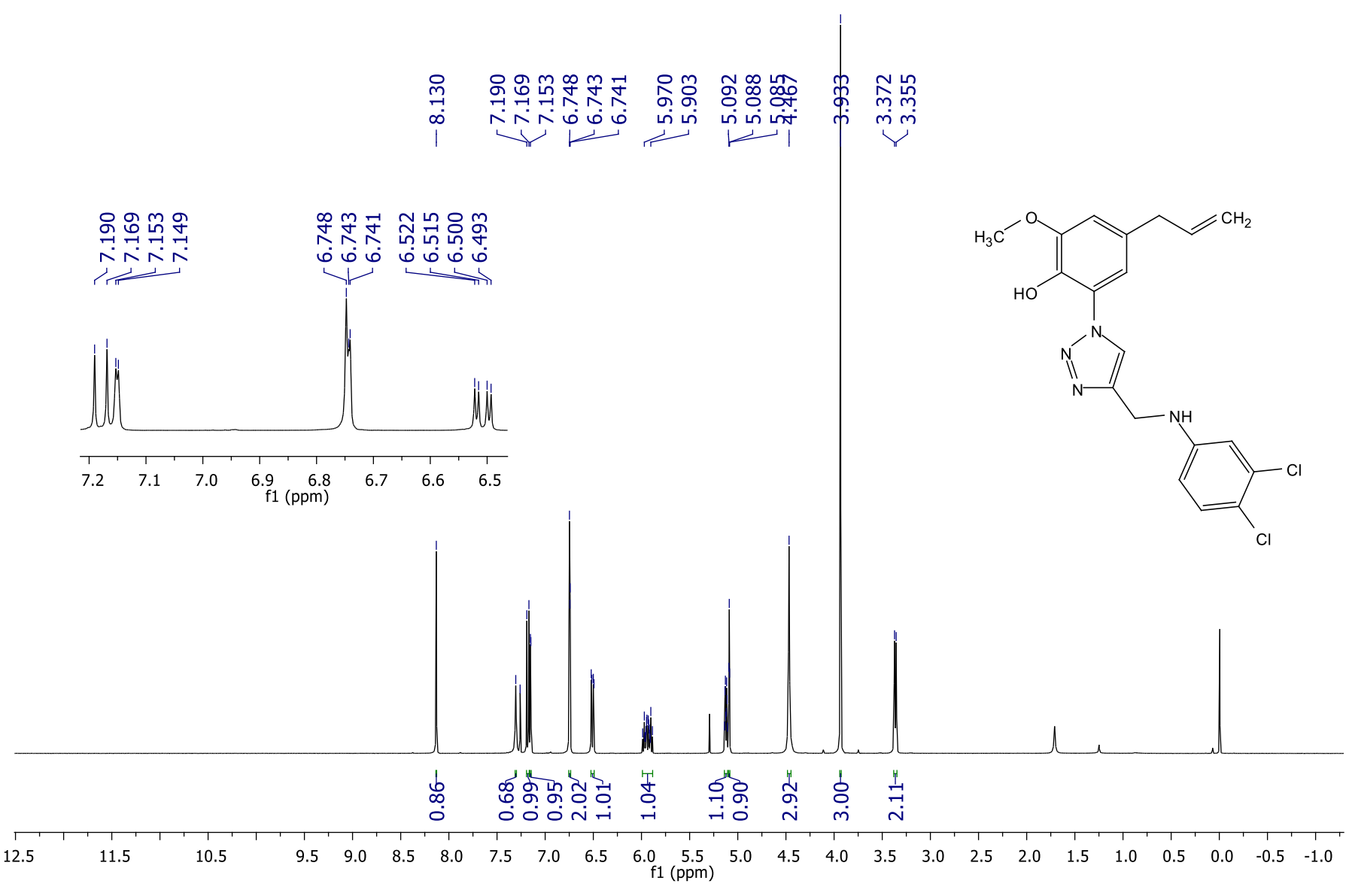

Figure S55. ^{1}H NMR of **4l** (400 MHz, $CDCl_3$).

^{13}C NMR of 4l (101 MHz, CDCl$_3$)

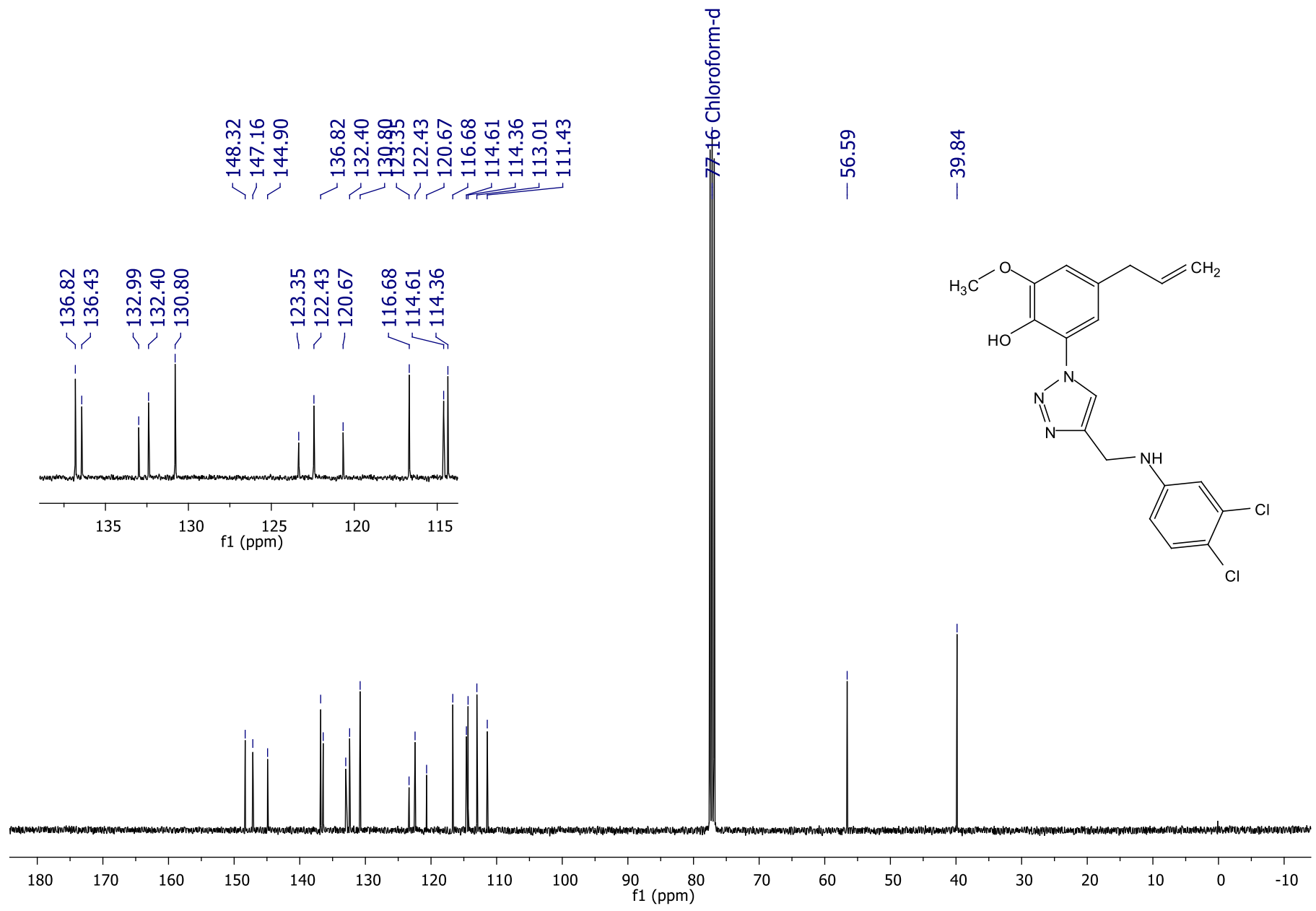

Figure S56. ^{13}C NMR of **4l** (101 MHz, CDCl$_3$).

^{13}C NMR DEPT--135 of 4l (101 MHz, CDCl$_3$)

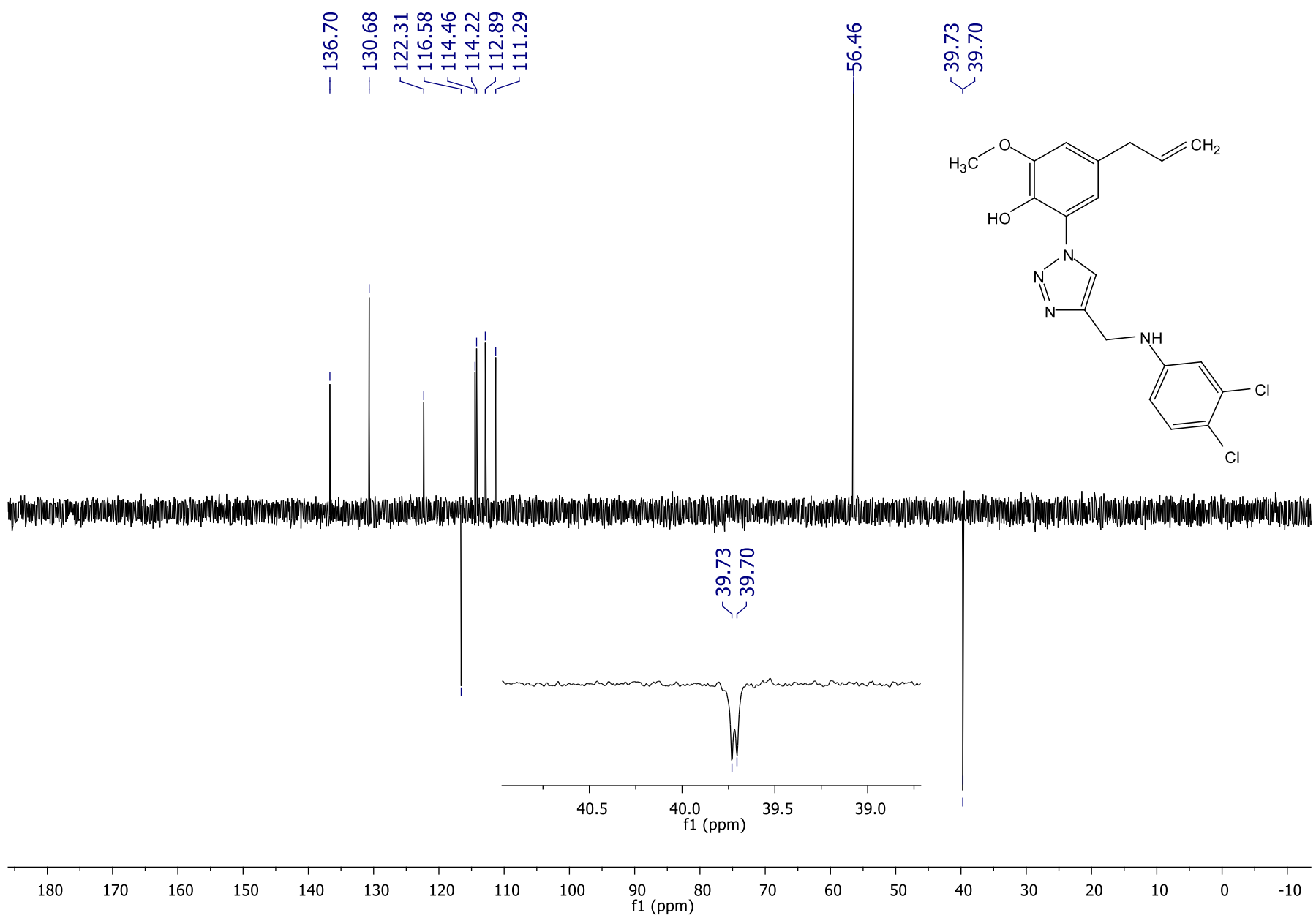

Figure S57. ^{13}C NMR DEPT--135 of **4l** (101 MHz, CDCl$_3$).

$^{1}H/^{13}C$ NMR HSQC of 4l (400 and 101 MHz, CDCl₃)

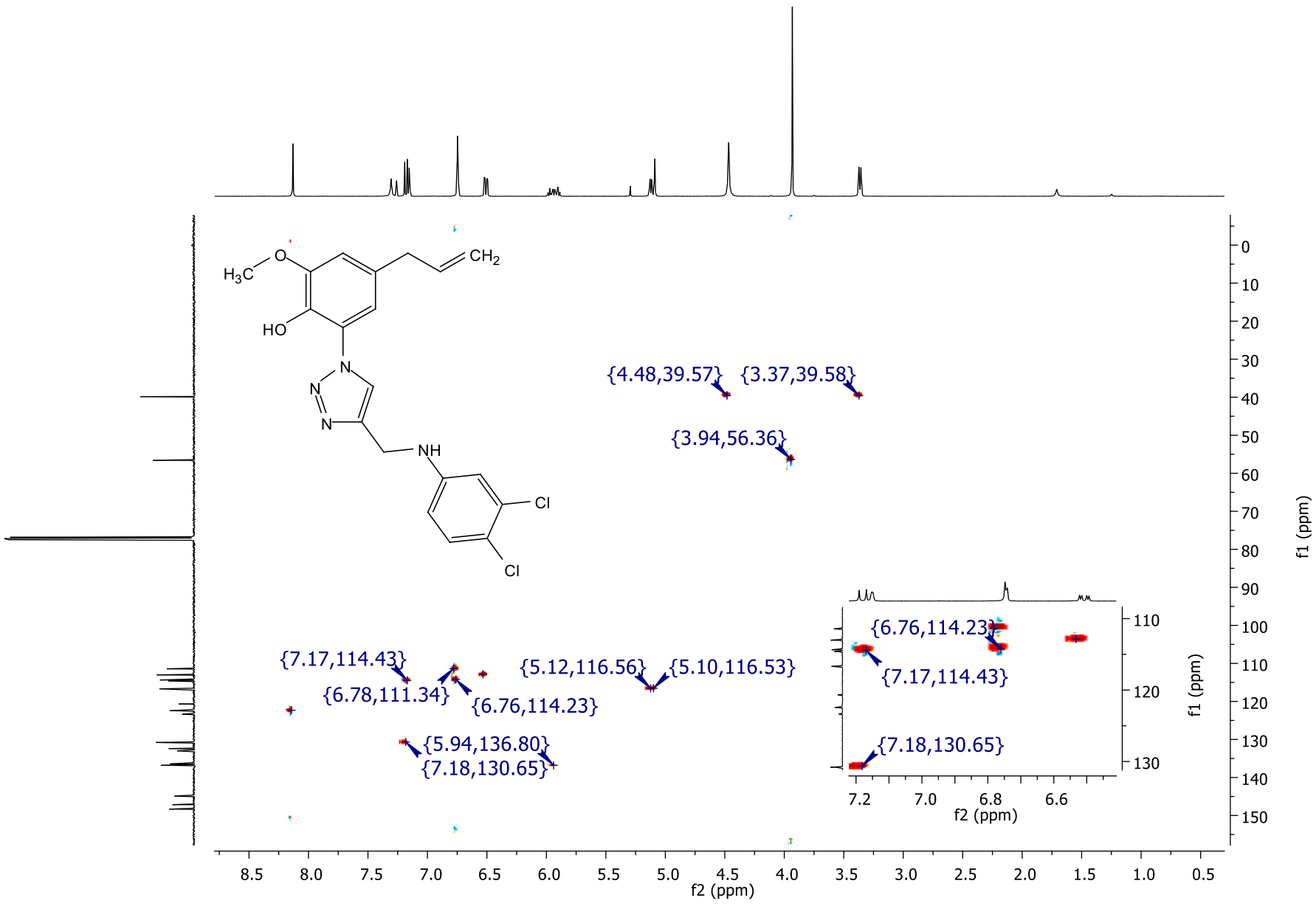

Figure S58. $^{1}H/^{13}C$ NMR HSQC of **4l** (400 and 101 MHz, $CDCl_3$).

^{1}H NMR of 4m (500 MHz, CDCl₃)

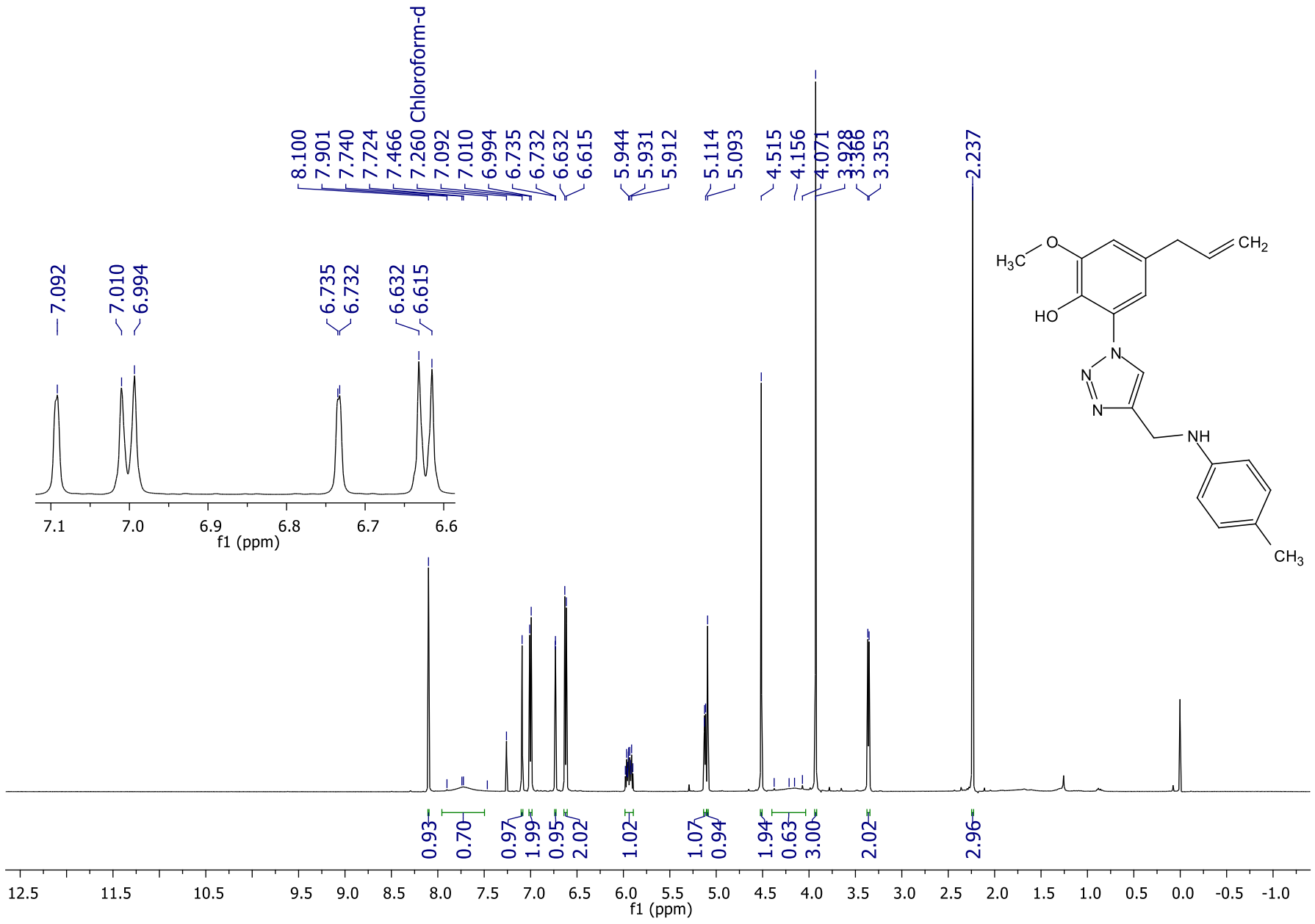

Figure S59. ^{1}H NMR of **4m** (500 MHz, $CDCl_3$).

^{13}C NMR of 4m (126 MHz, CDCl$_3$)

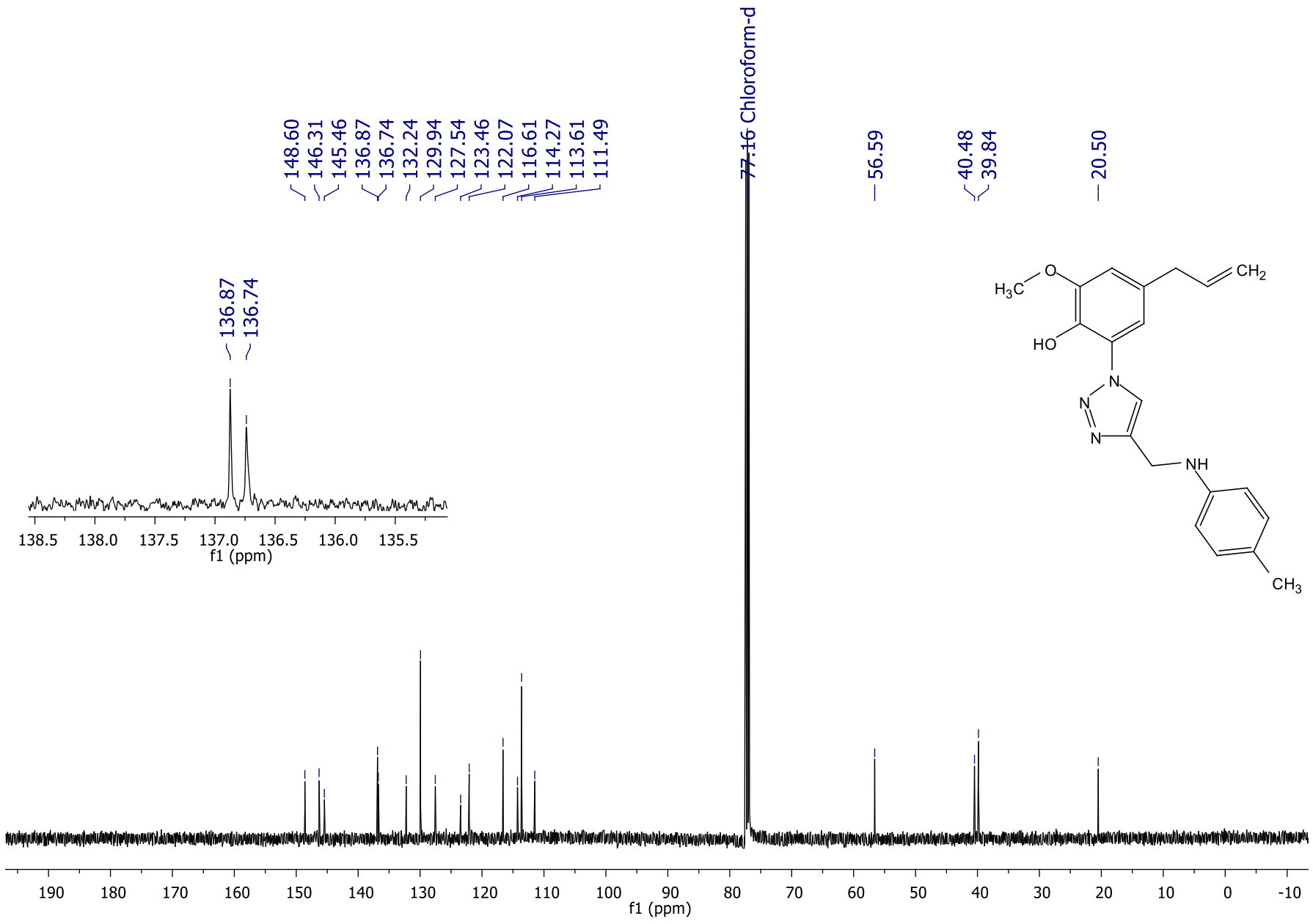

Figure S60. ^{13}C NMR of **4m** (126 MHz, CDCl$_3$).

^{13}C NMR DEPT--135 of 4m (126 MHz, CDCl$_3$)

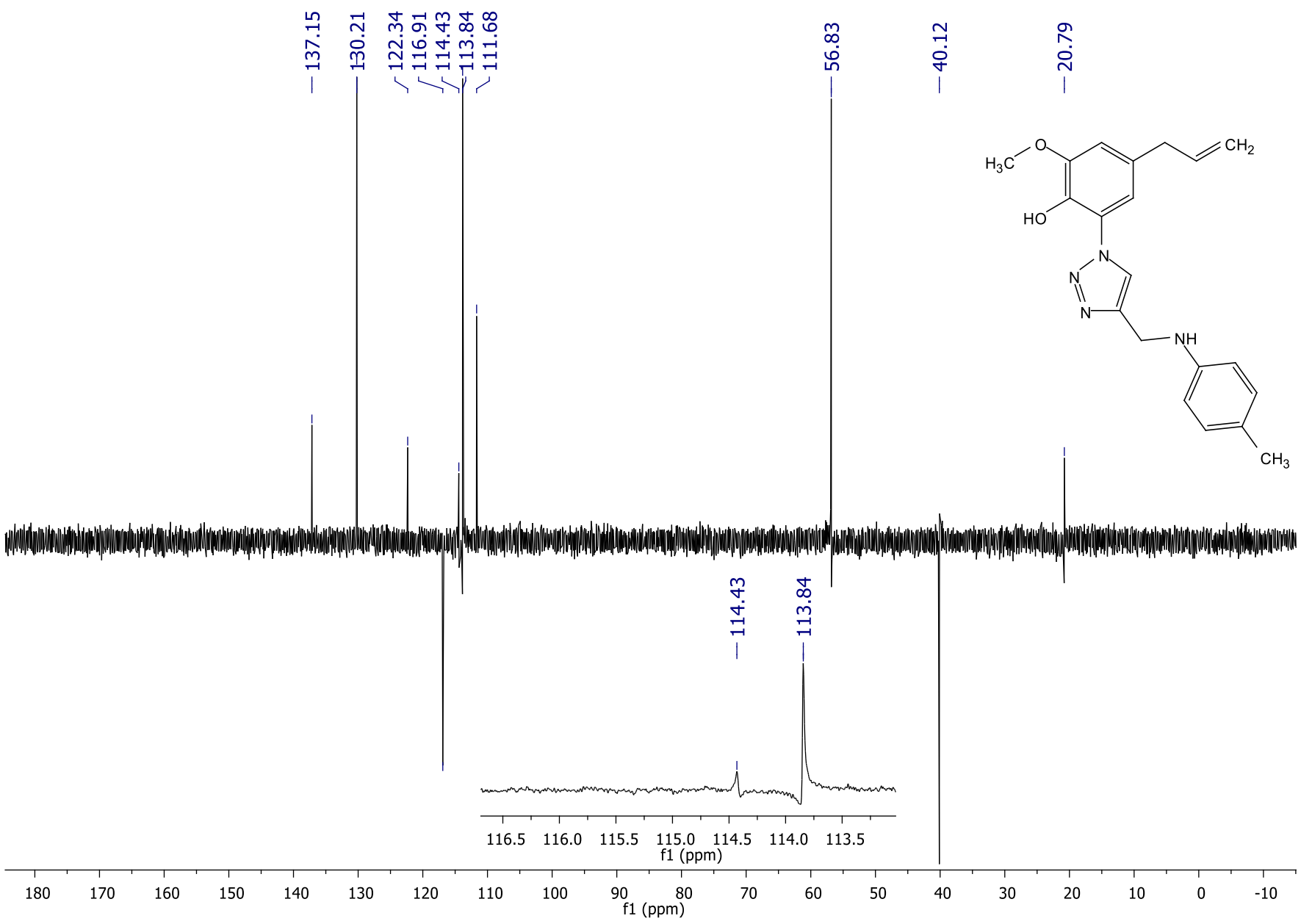

Figure S61. ^{13}C NMR DEPT--135 of **4m** (126 MHz, CDCl$_3$).

$^1H/^{13}C$ NMR HSQC of 4m (500 and 126 MHz, $CDCl_3$)

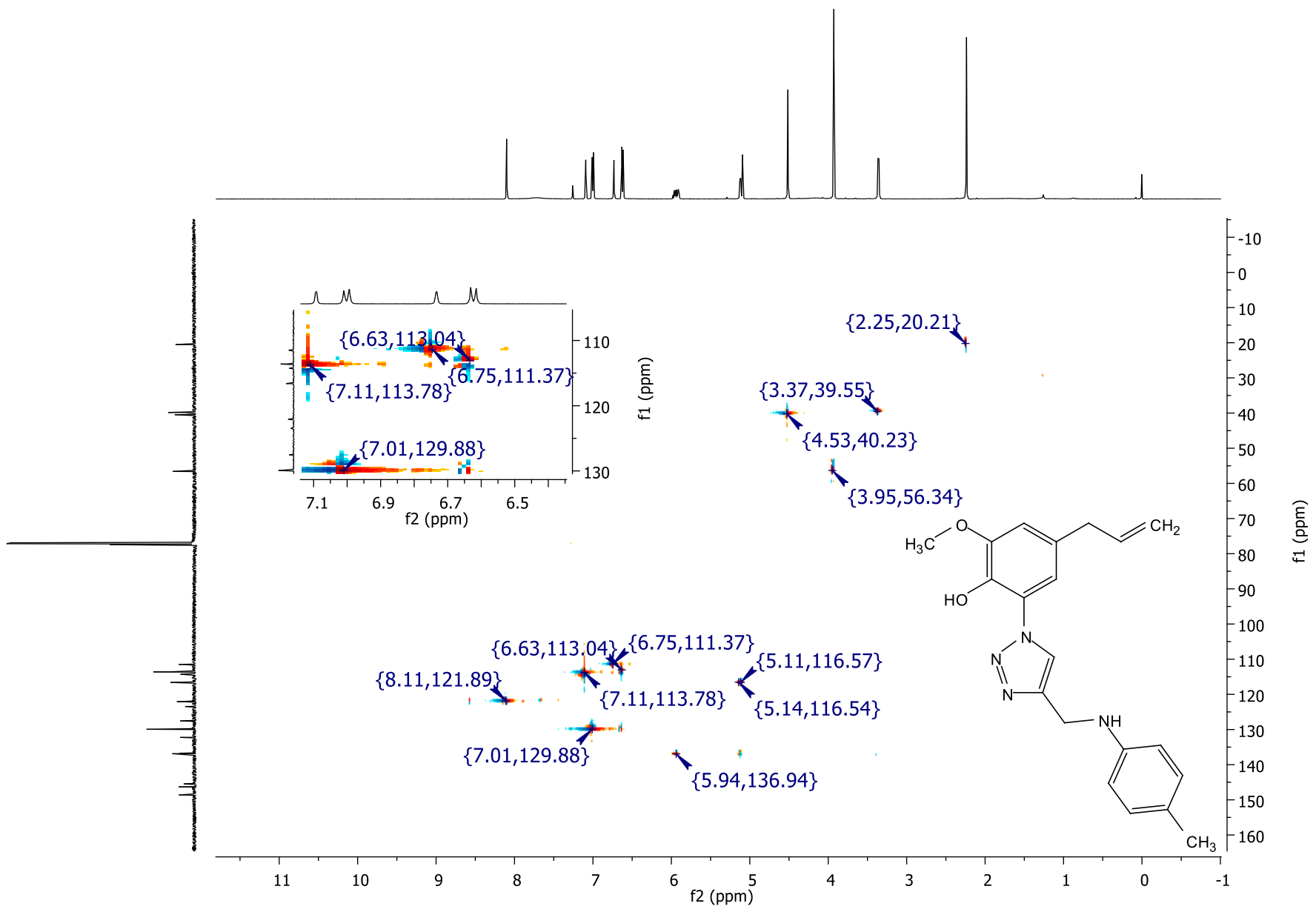

Figure S62. $^1H/^{13}C$ NMR HSQC of **4m** (500 and 126 MHz, $CDCl_3$).

^1H NMR of 4n (400 MHz, $CDCl_3$)

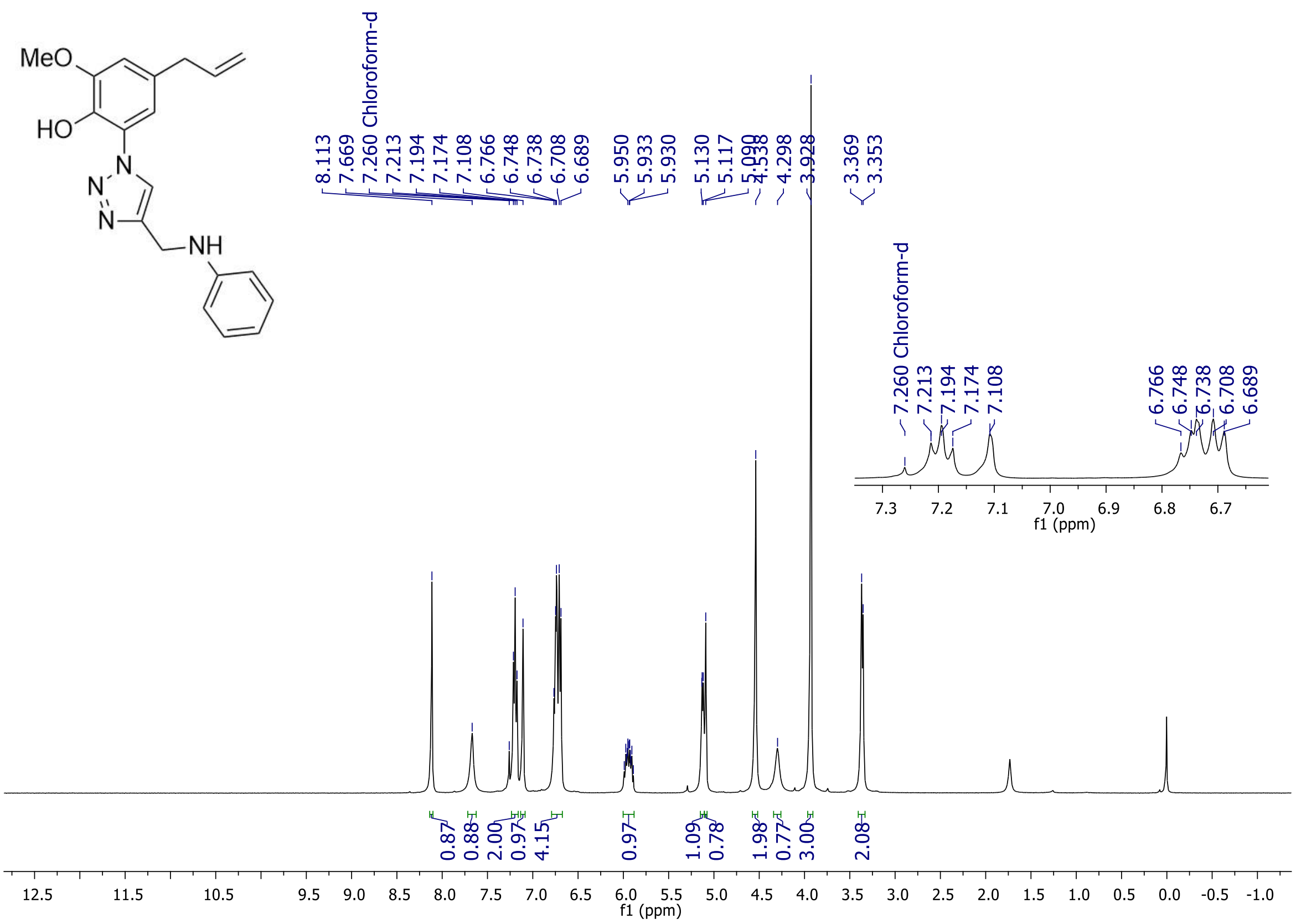

Figure S63. ^1H NMR of **4n** (400 MHz, $CDCl_3$).

^{13}C NMR of 4n (101 MHz, CDCl$_3$)

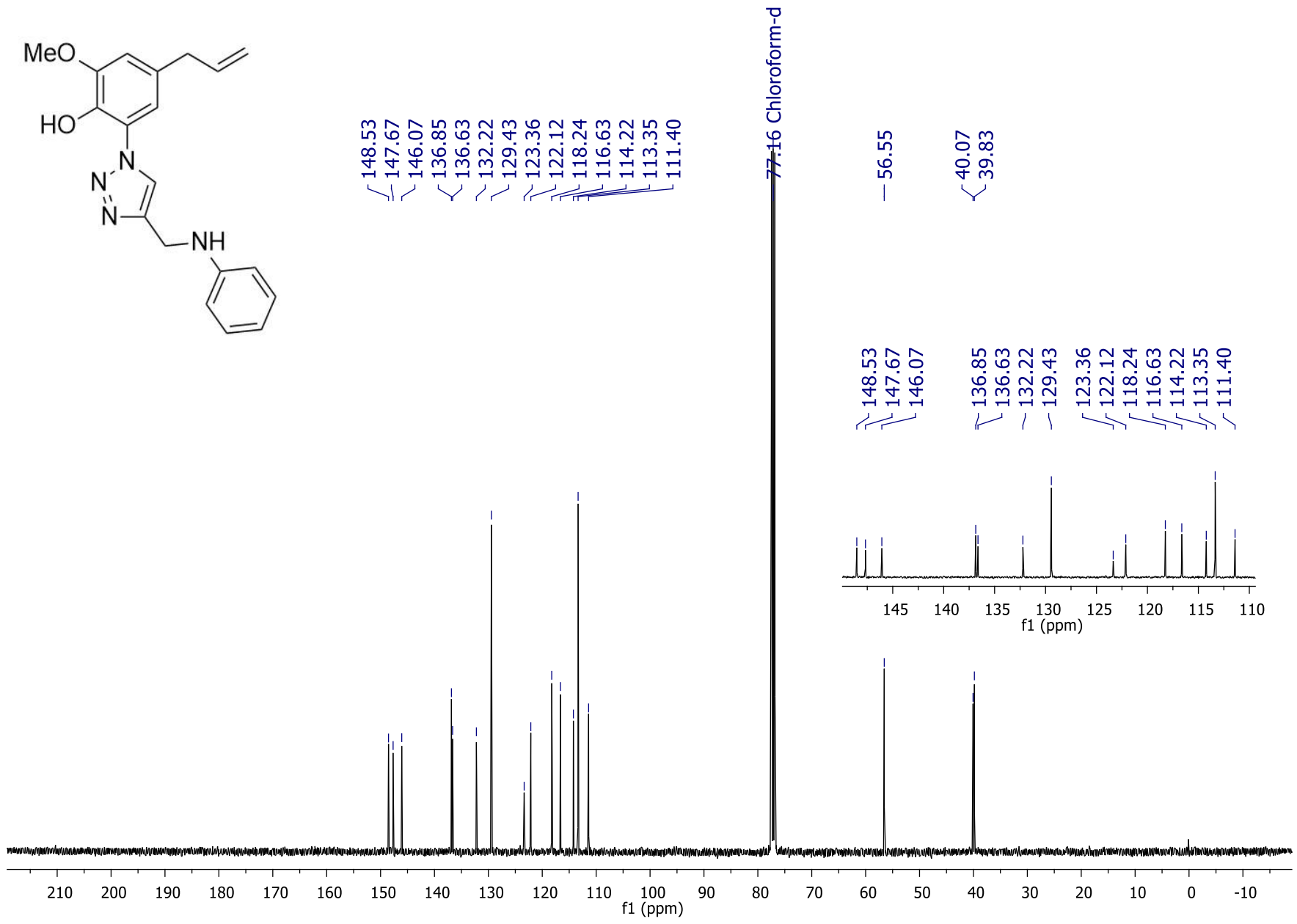

Figure S64. ^{13}C NMR of **4n** (101 MHz, CDCl$_3$).

^{13}C NMR DEPT--135 of 4n (101 MHz, CDCl$_3$)

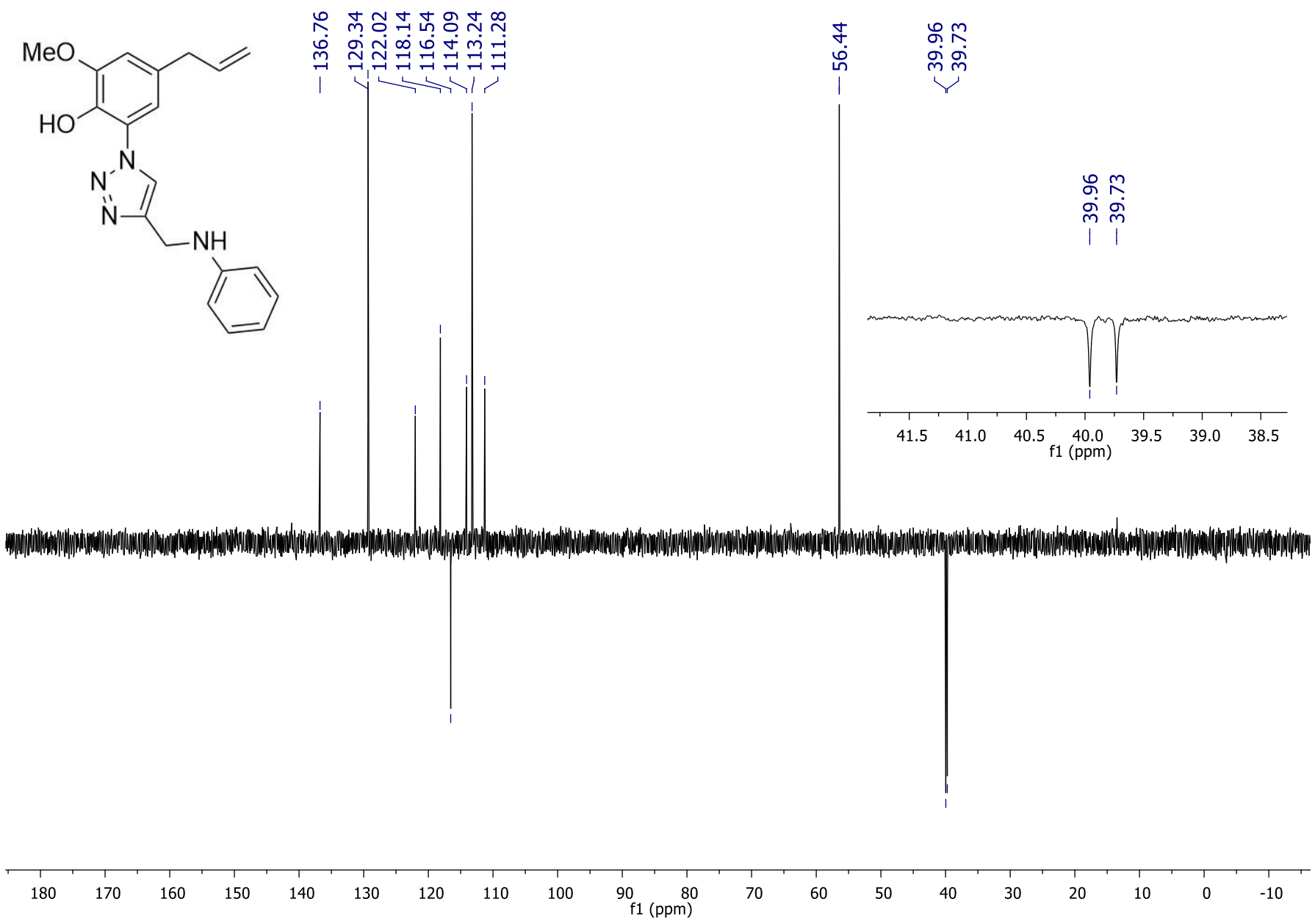

Figure S65. ^{13}C NMR DEPT--135 of **4n** (101 MHz, CDCl$_3$).

$^{1}H/^{13}C$ NMR HSQC of 4n (400 and 101 MHz, CDCl₃)

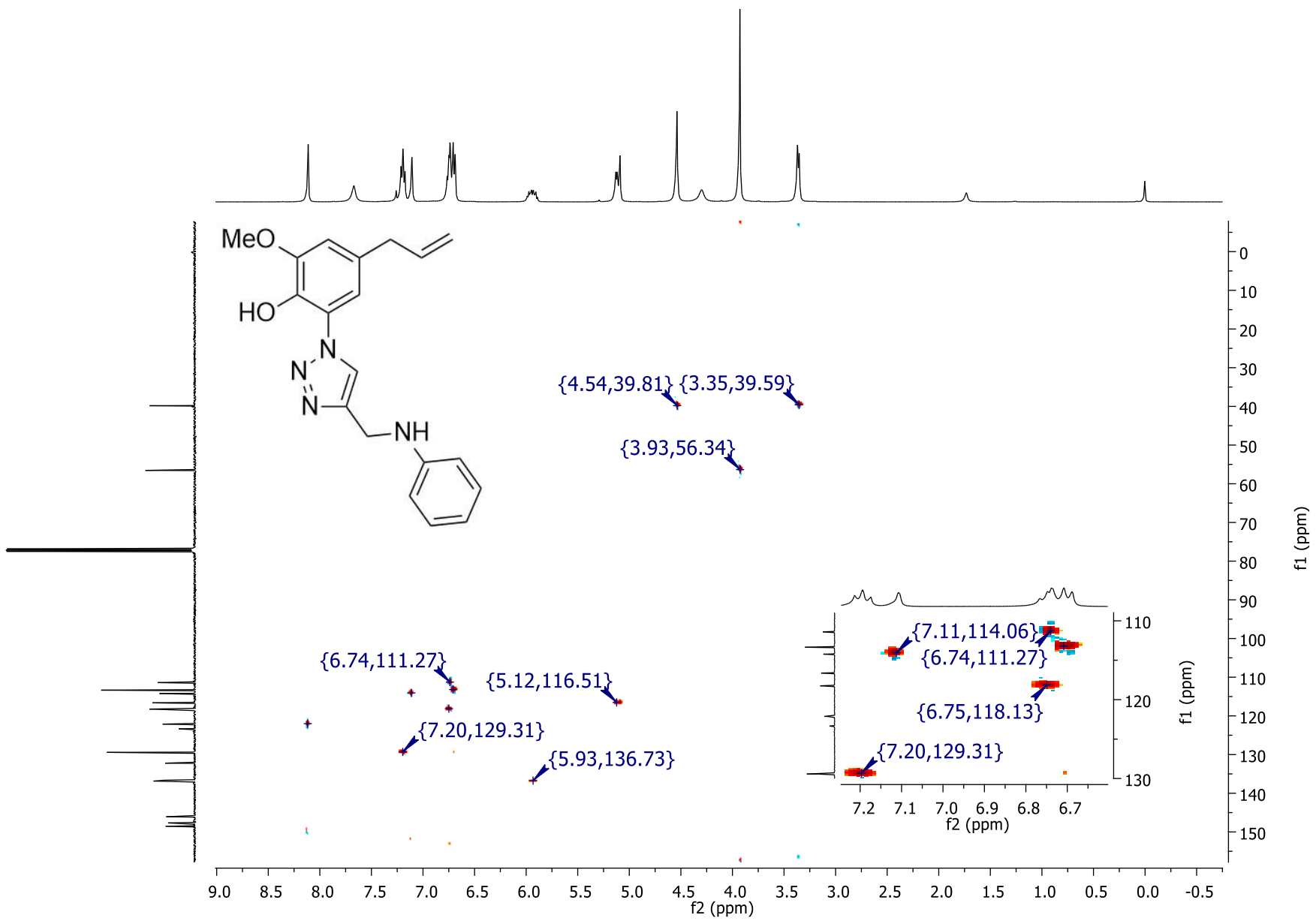

Figure S66. $^{1}H/^{13}C$ NMR HSQC of **4n** (400 and 101 MHz, $CDCl_3$).

^{1}H NMR of 4o (500 MHz, DMSO)

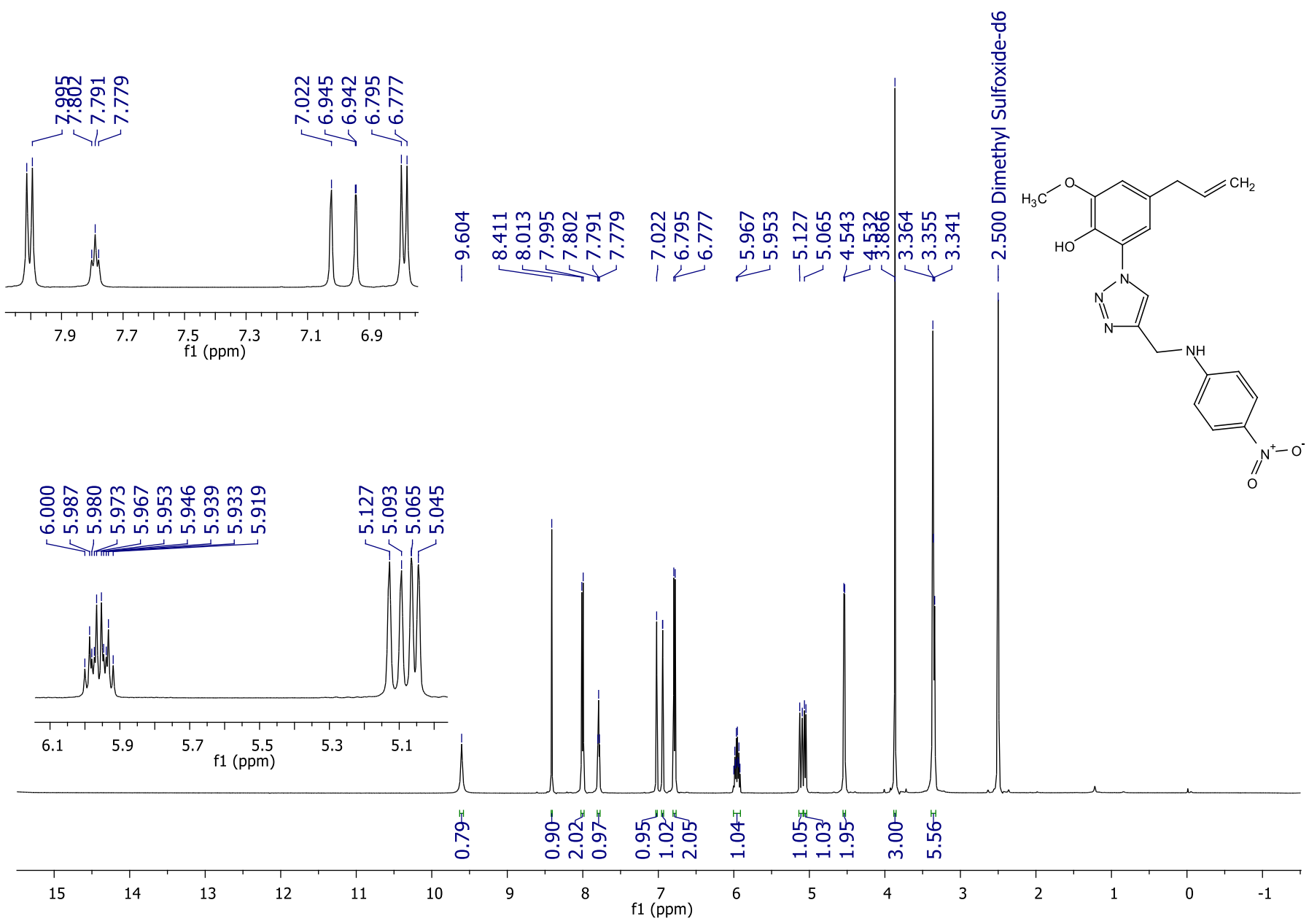

Figure S67. ^{1}H NMR of **4o** (500 MHz, DMSO).

^{13}C NMR of 4o (126 MHz, DMSO)

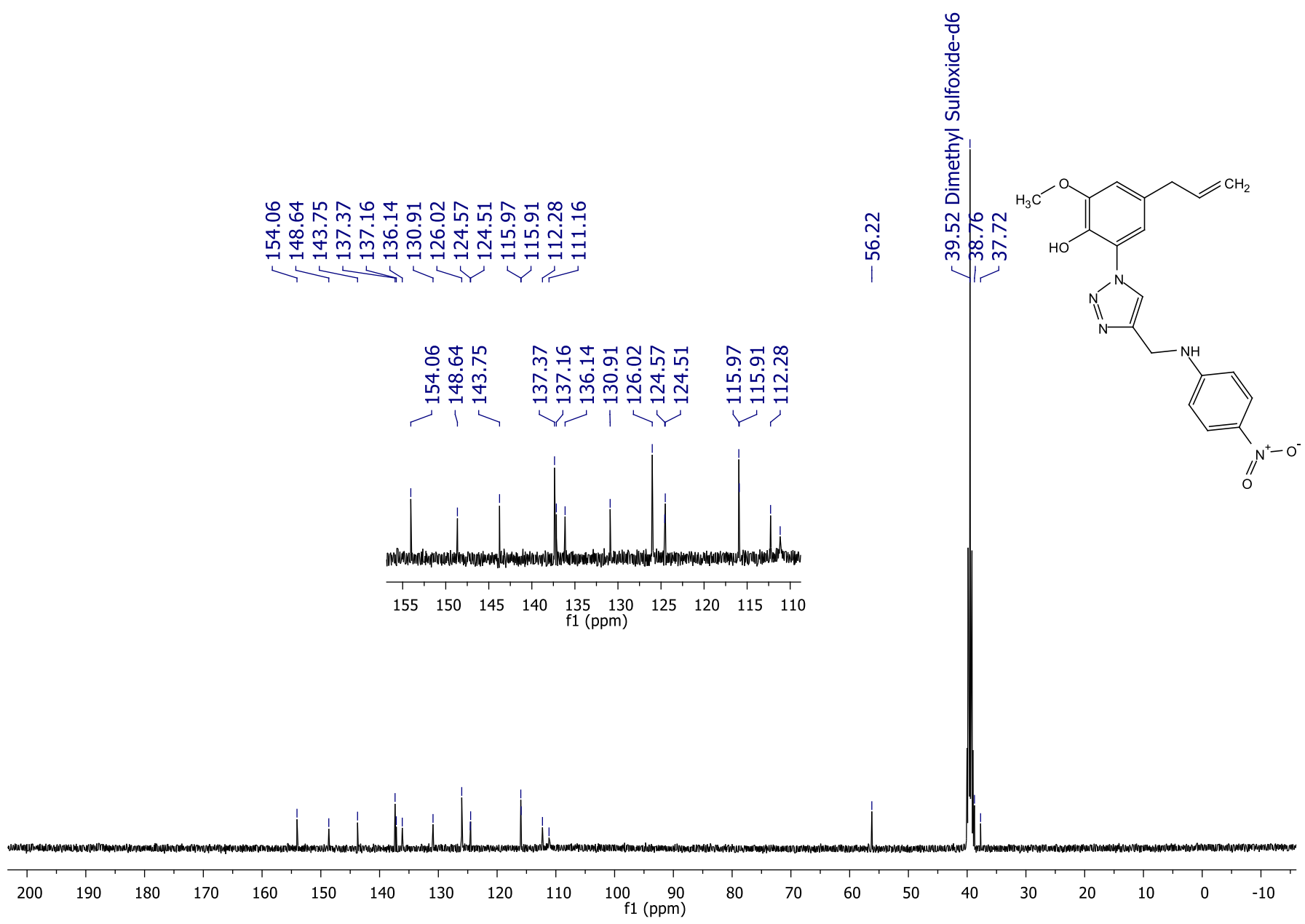

Figure S68. ^{13}C NMR of **4o** (126 MHz, DMSO).

^{13}C NMR DEPT--135 of 4o (126 MHz, DMSO)

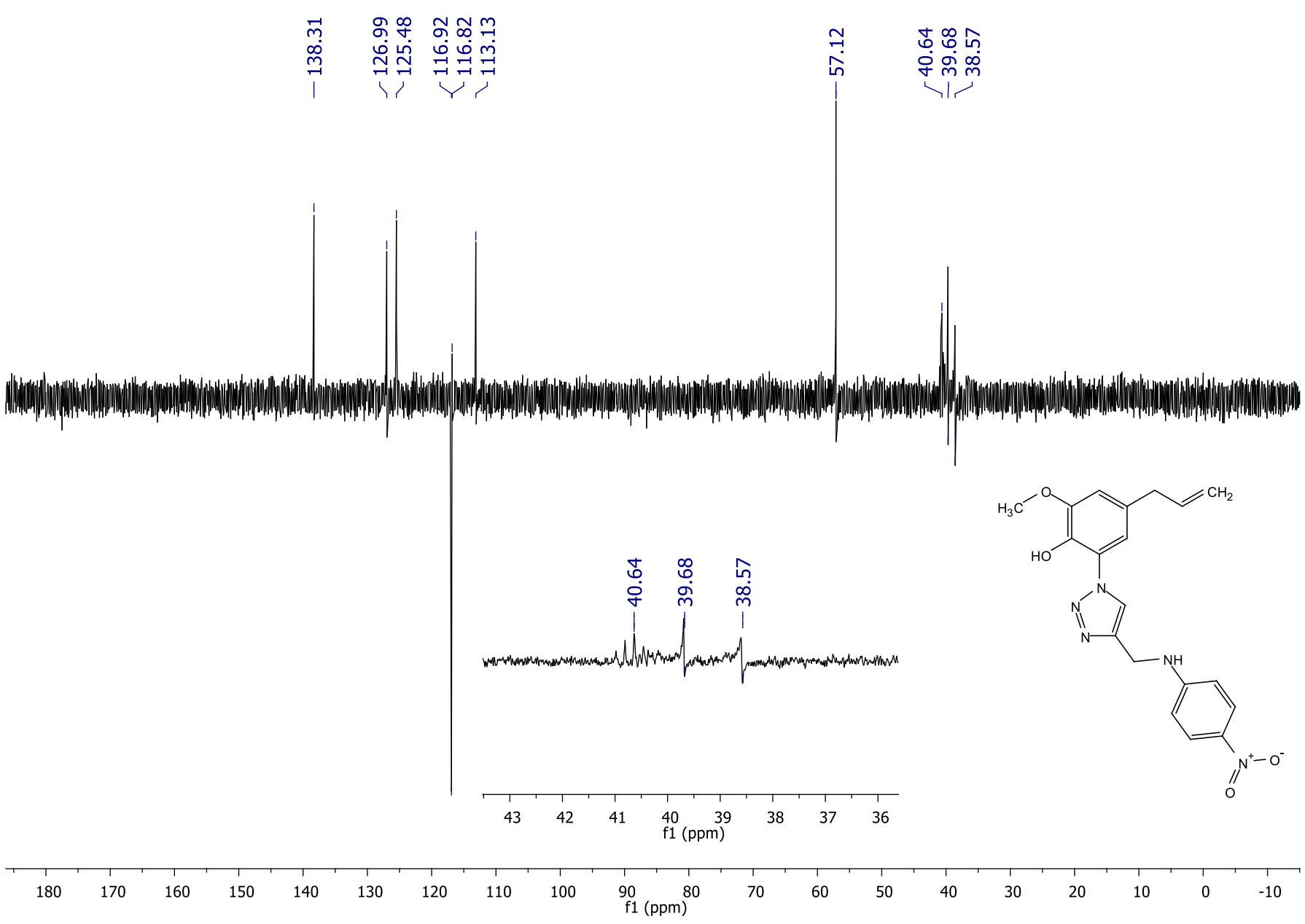

Figure S69. ^{13}C NMR DEPT--135 of **4o** (126 MHz, DMSO).

$^{1}H/^{13}C$ NMR HSQC of 4o (500 and 126 MHz, DMSO)

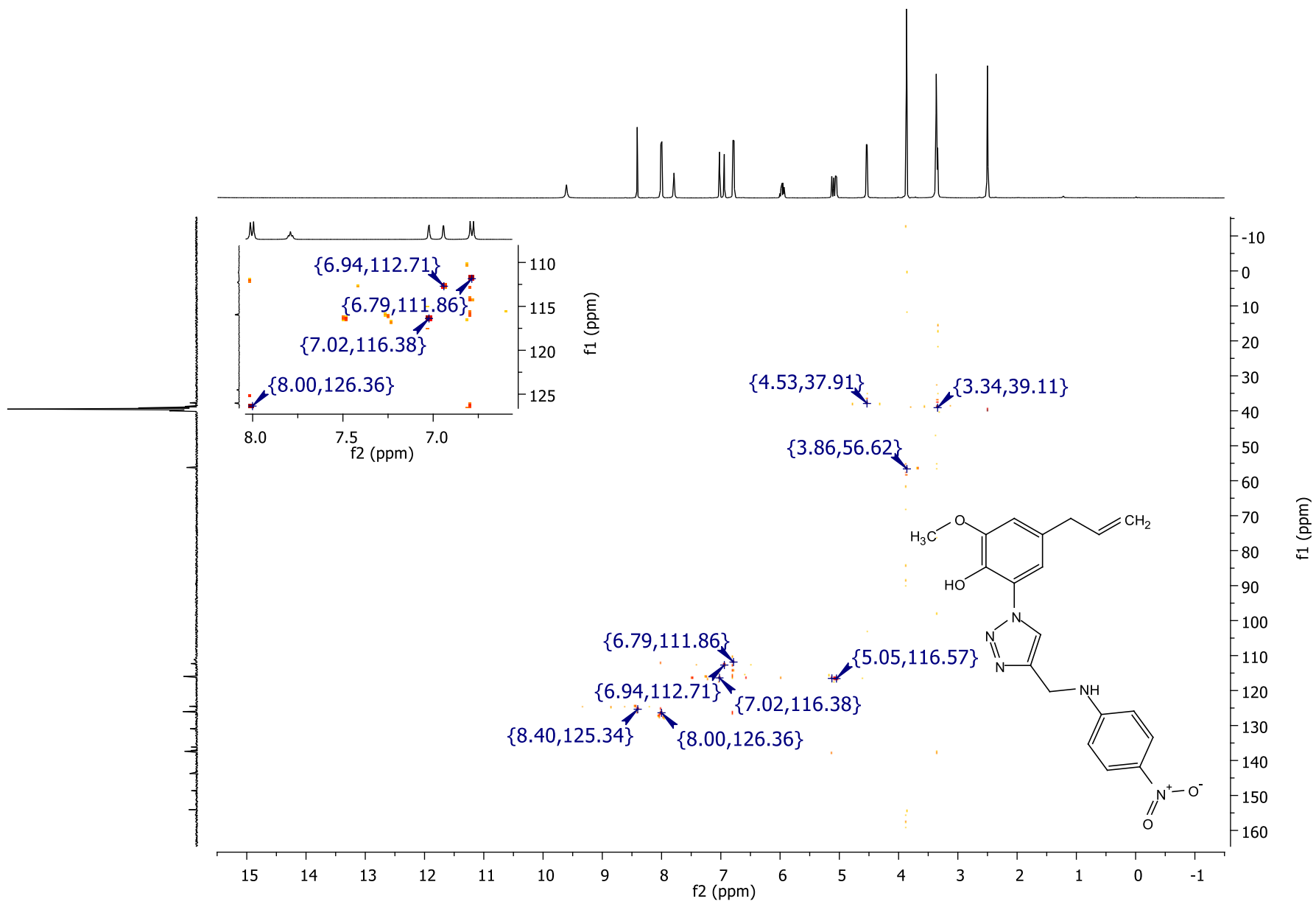

Figure S70. $^{1}H/^{13}C$ NMR HSQC of **4o** (500 and 126 MHz, DMSO).

^{1}H NMR of 4p (400 MHz, DMSO)

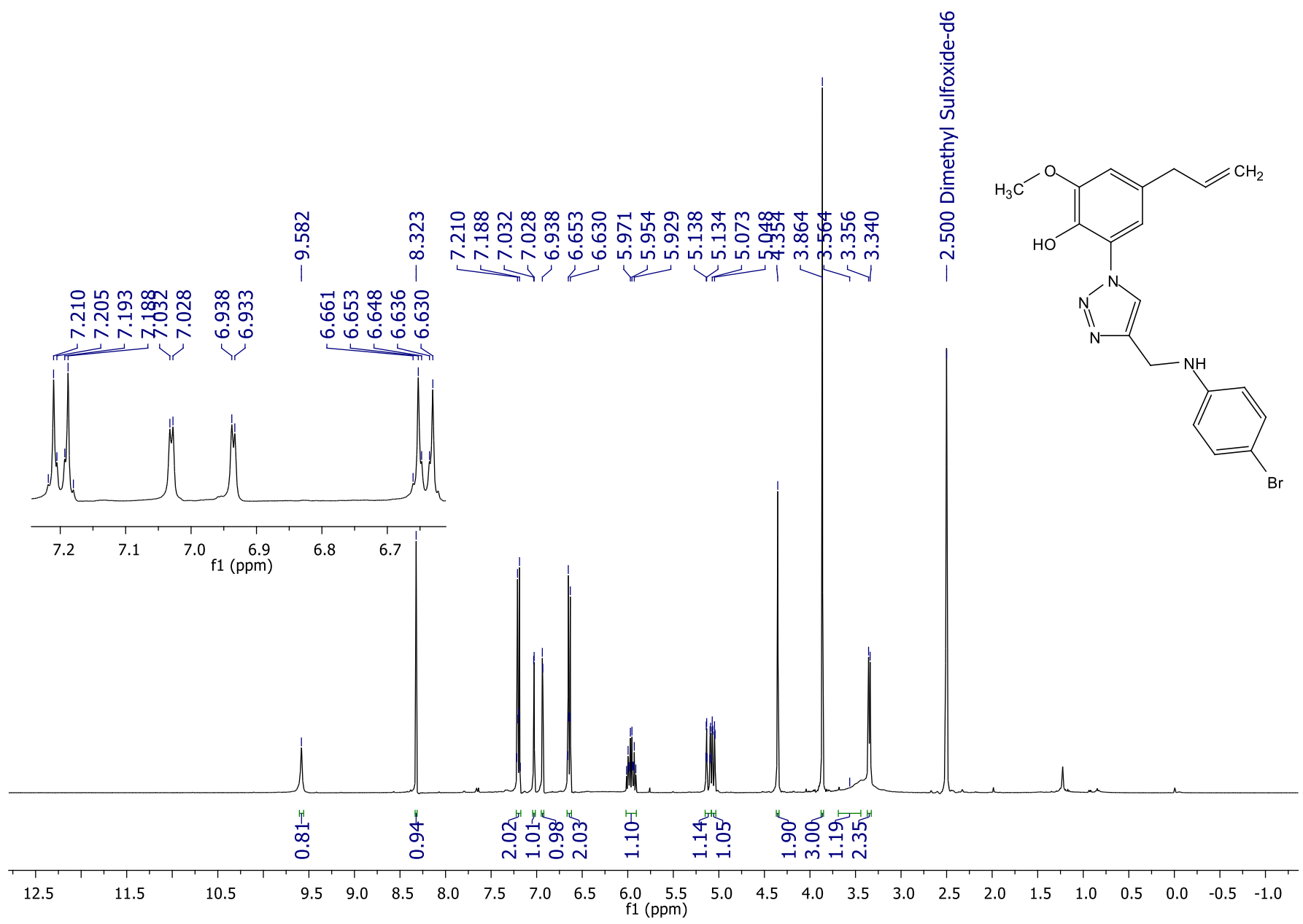

Figure S71. ^{1}H NMR of **4p** (400 MHz, DMSO).

^{13}C NMR of 4p (101 MHz, DMSO)

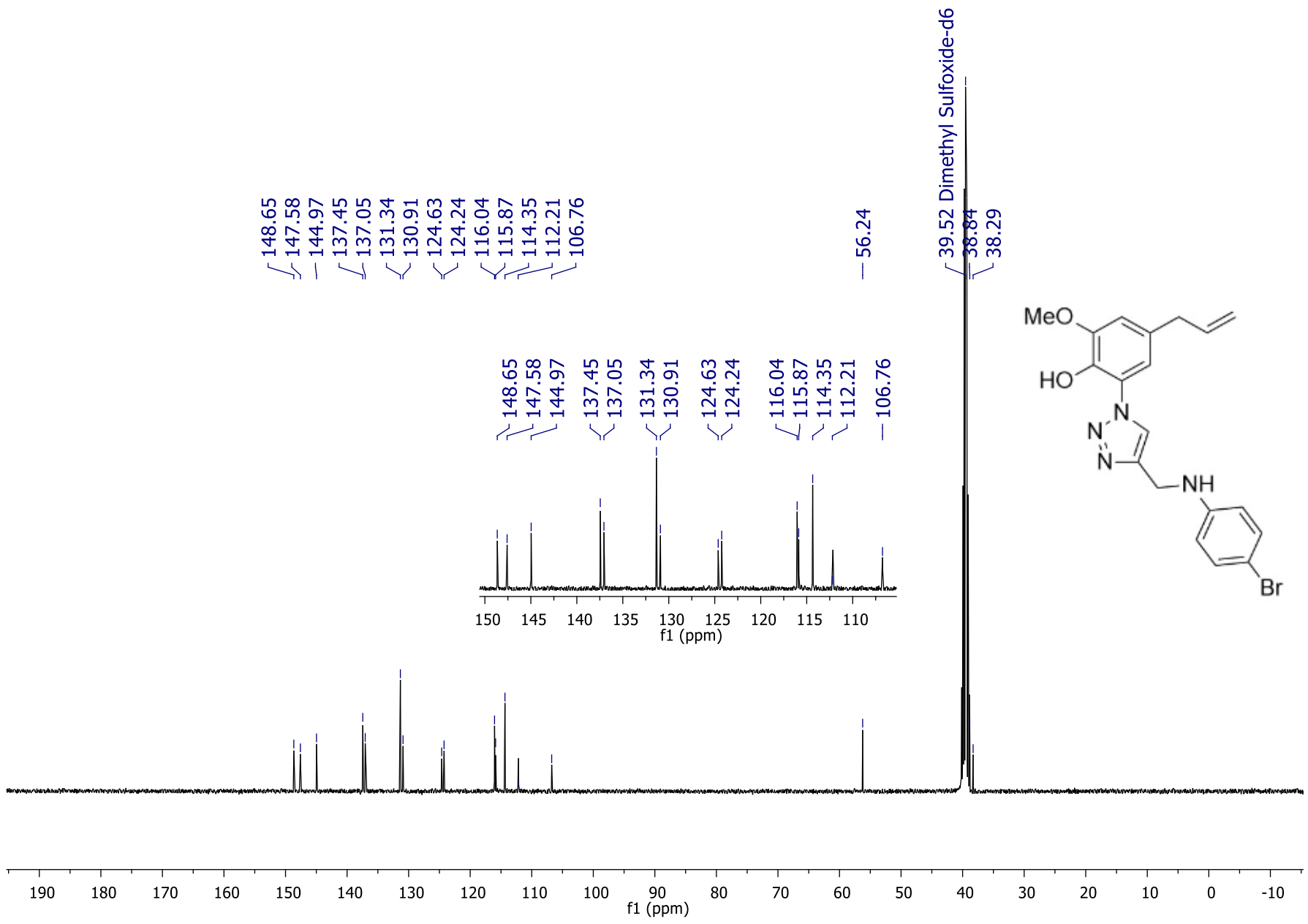

Figure S72. ^{13}C NMR of **4p** (101 MHz, DMSO).

^{13}C NMR DEPT--135 of 4p (101 MHz, DMSO)

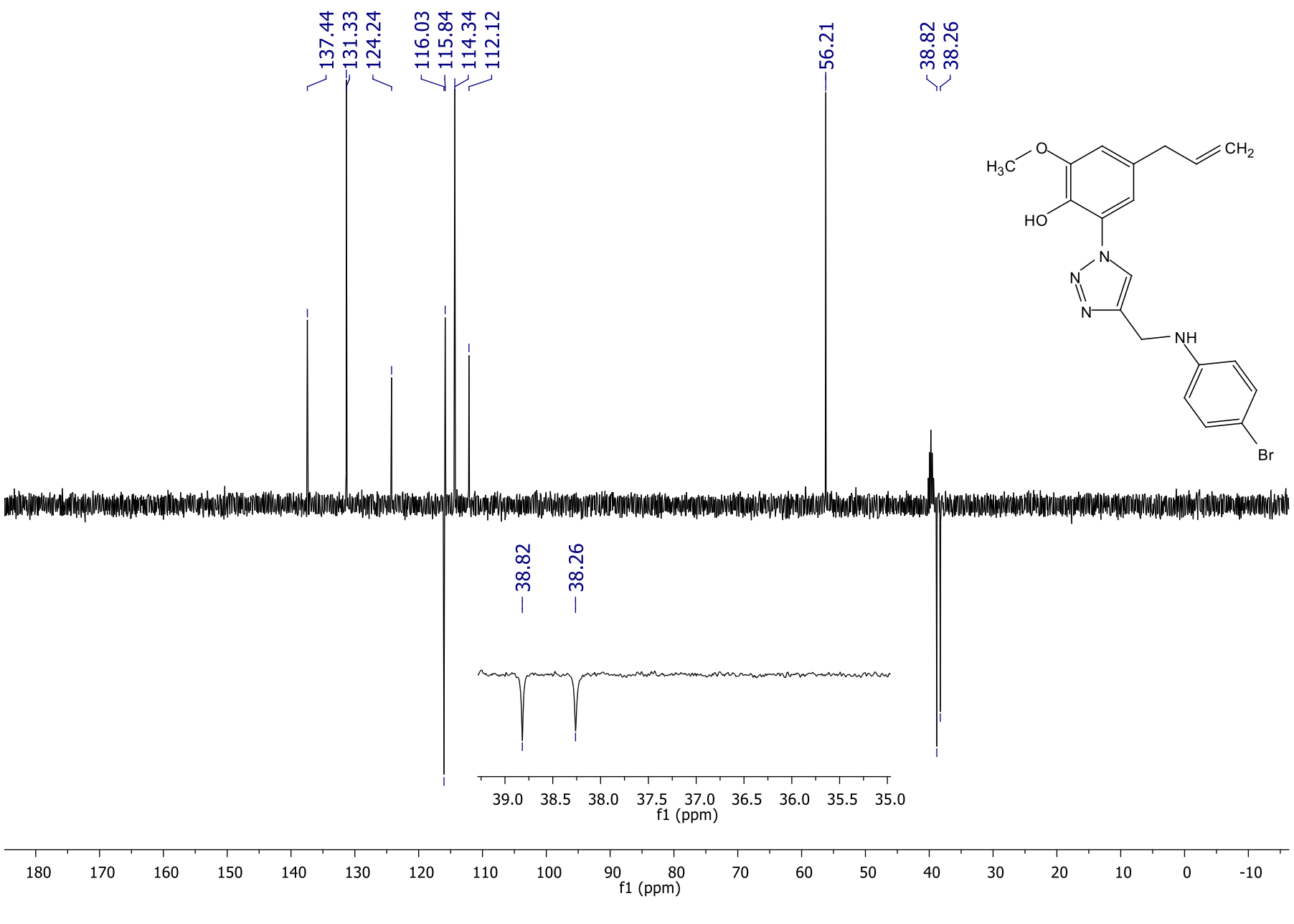

Figure S73. ^{13}C NMR DEPT--135 of **4p** (101 MHz, DMSO).

$^1H/^{13}C$ NMR HSQC of 4p (400 and 101 MHz, DMSO)

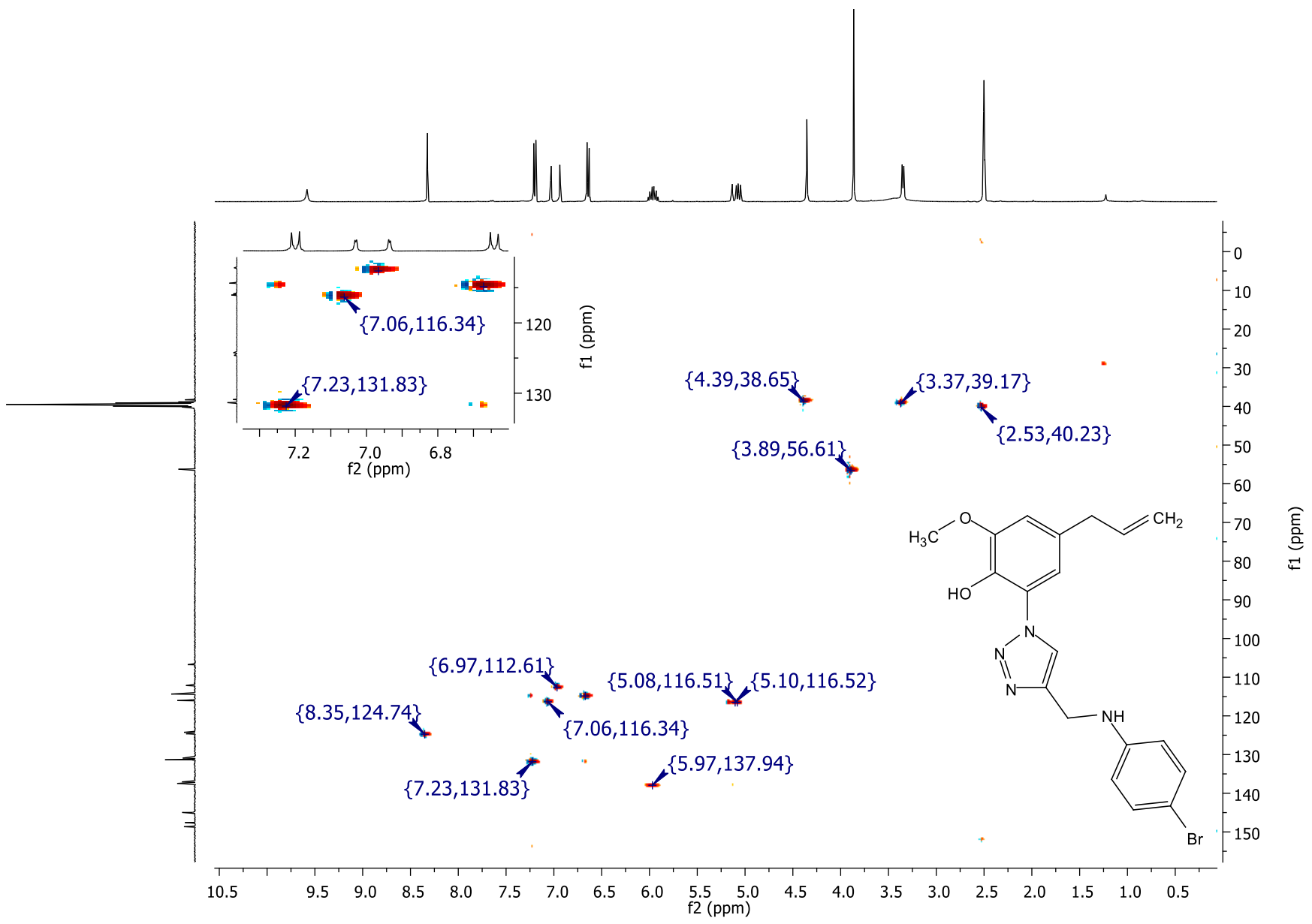

Figure S74. $^1H/^{13}C$ NMR HSQC of **4p** (400 and 101 MHz, DMSO).

^1H NMR of 4q (500 MHz, $CDCl_3$)

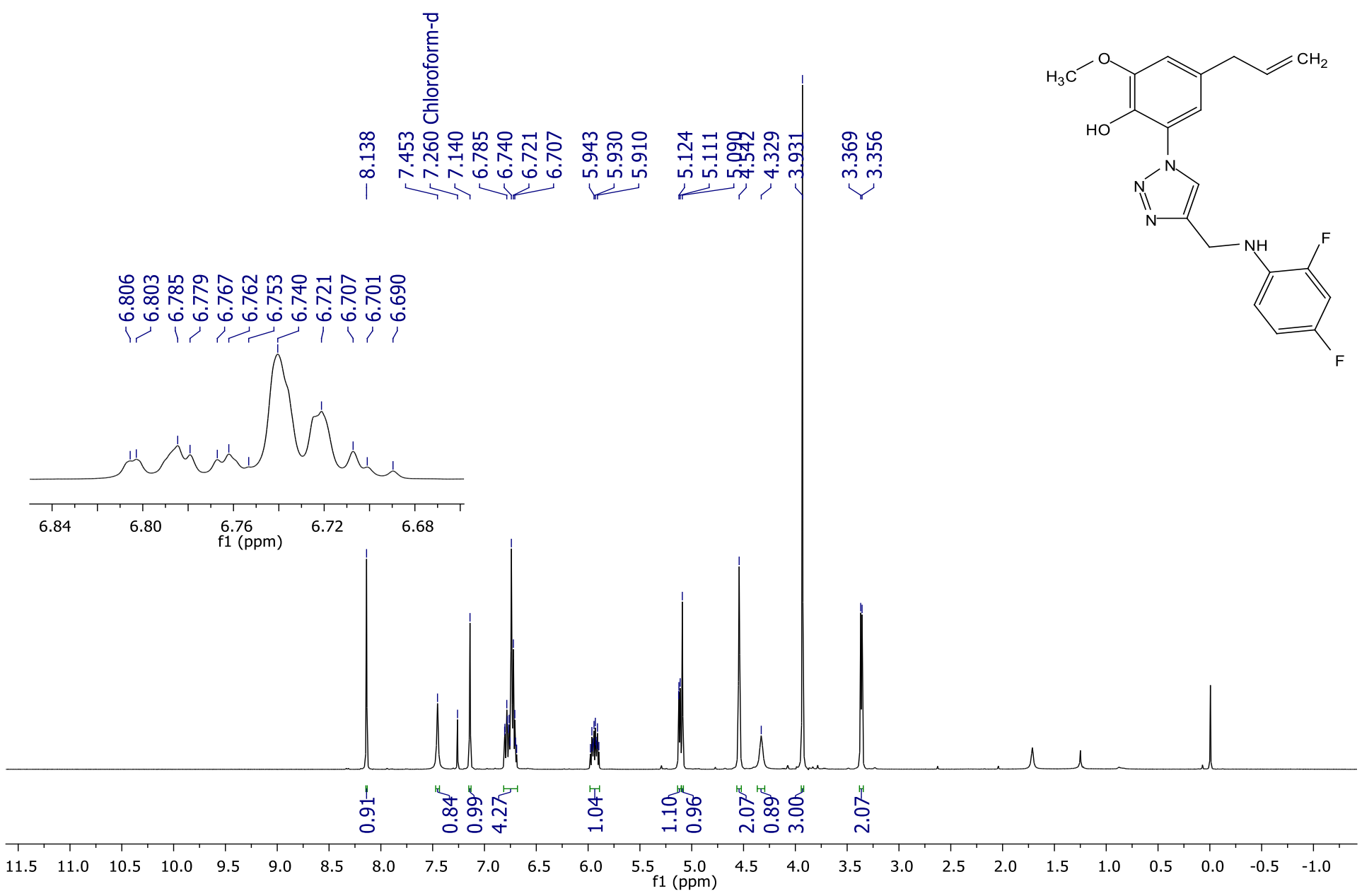

Figure S75. ^1H NMR of **4q** (500 MHz, $CDCl_3$).

^{13}C NMR of 4q (126 MHz, CDCl$_3$)

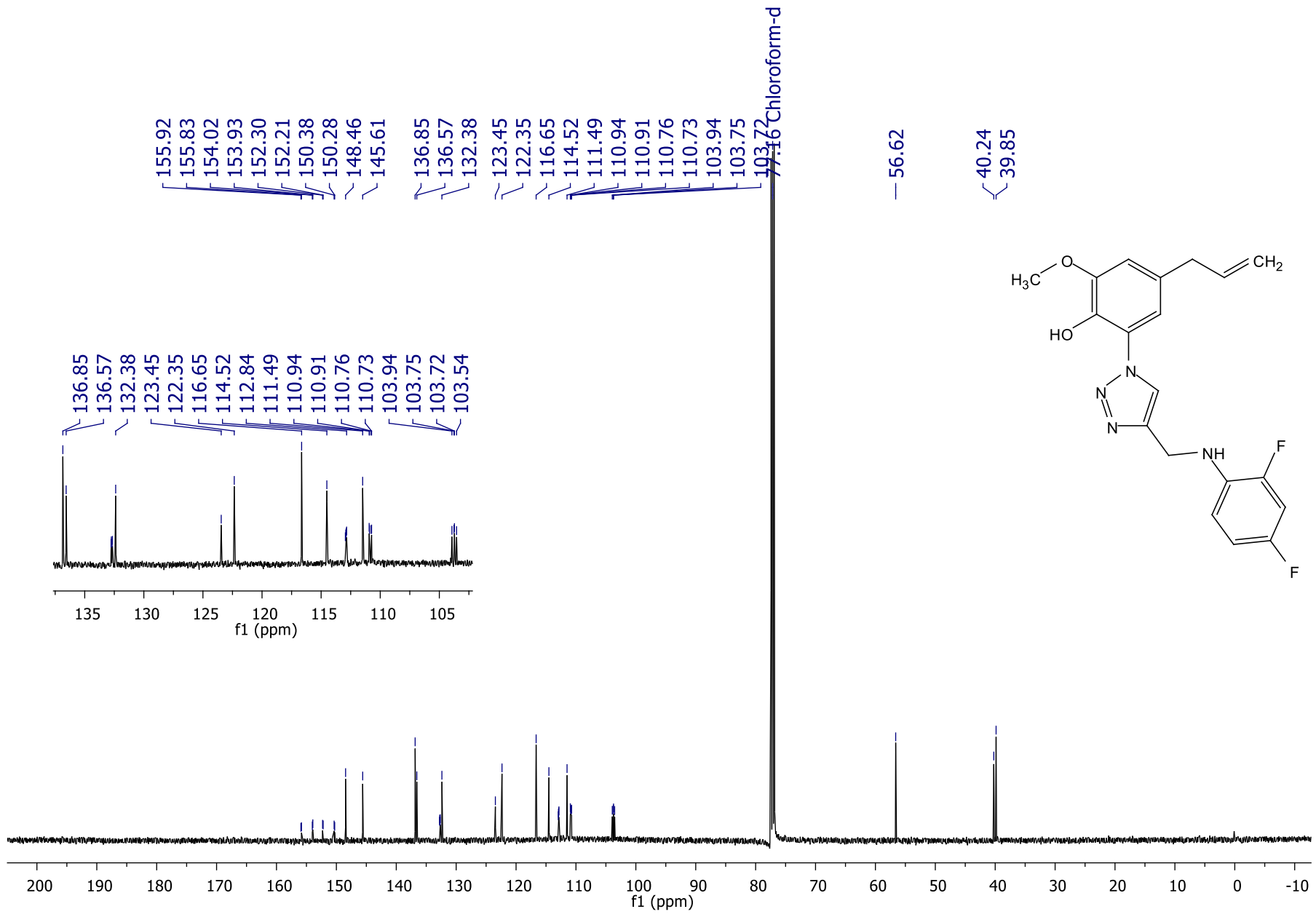

Figure S76. ^{13}C NMR of **4q** (126 MHz, CDCl$_3$).

^{13}C NMR DEPT--135 of 4q (126 MHz, CDCl$_3$)

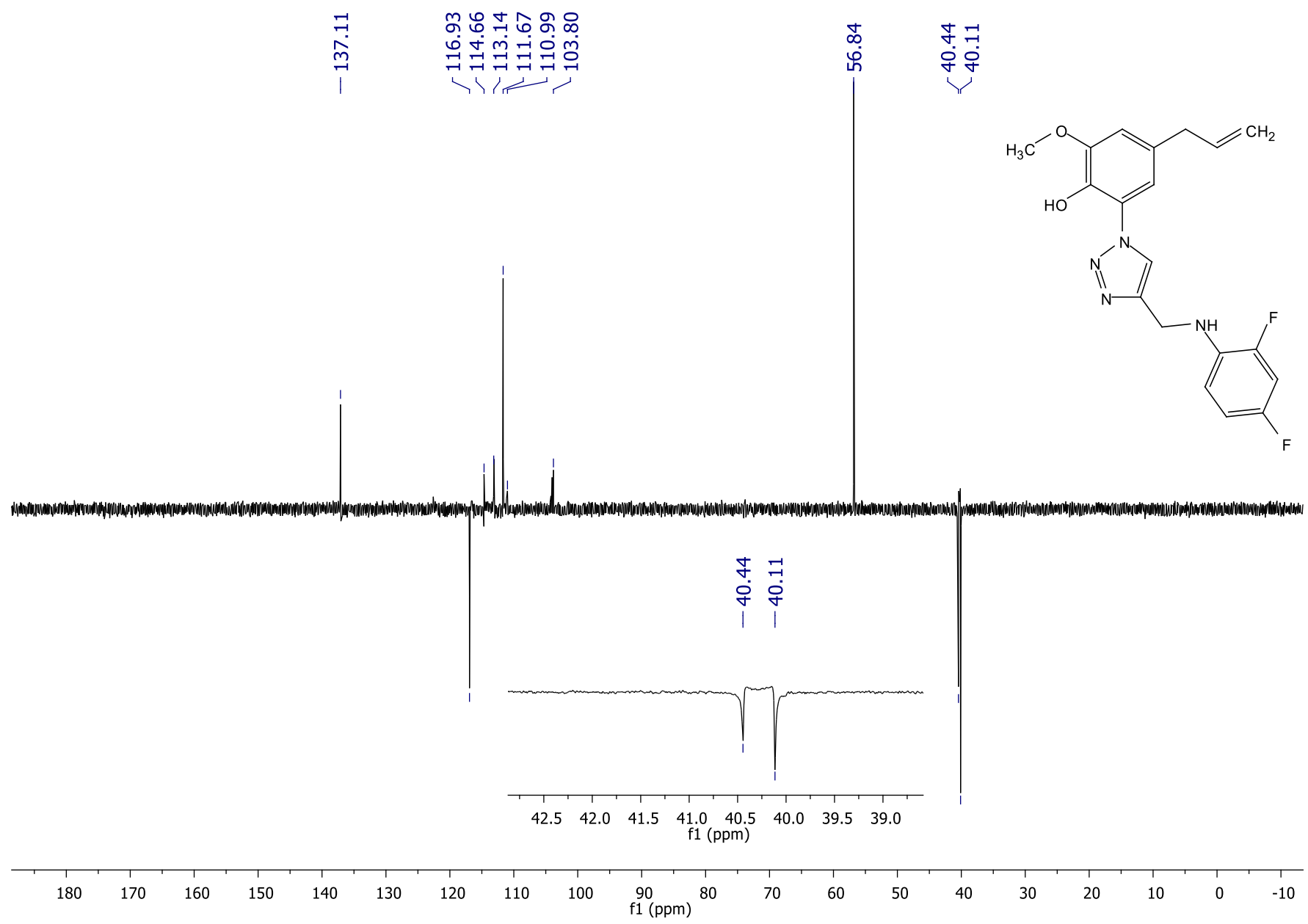

Figure S77. ^{13}C NMR DEPT--135 of **4q** (126 MHz, CDCl$_3$).

$^{1}H/^{13}C$ NMR HSQC of 4q (500 and 126 MHz, $CDCl_3$)

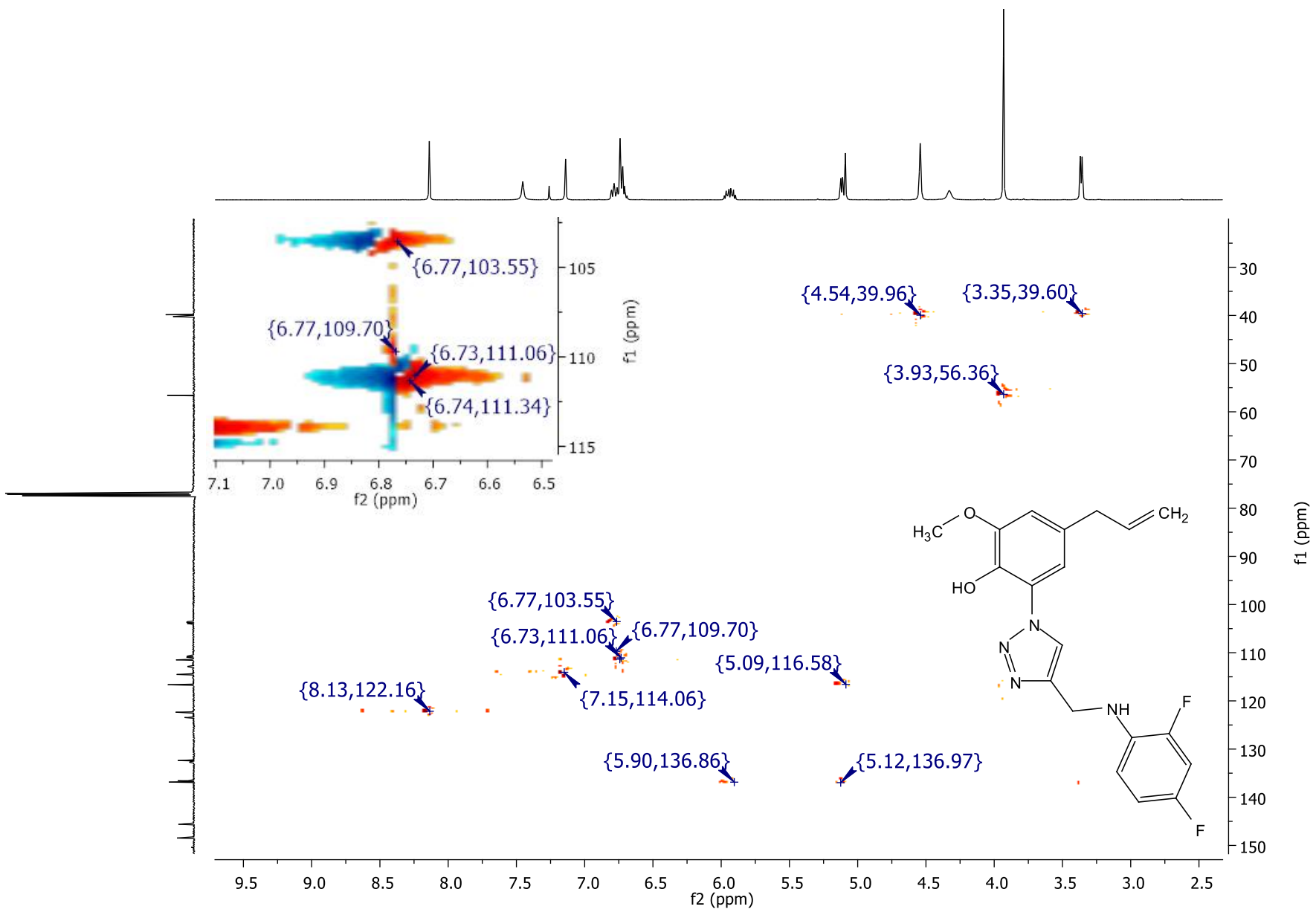

Figure S78. $^{1}H/^{13}C$ NMR HSQC of **4q** (500 and 126 MHz, $CDCl_3$).

^{1}H NMR of 4r (400 MHz, $CDCl_3$)

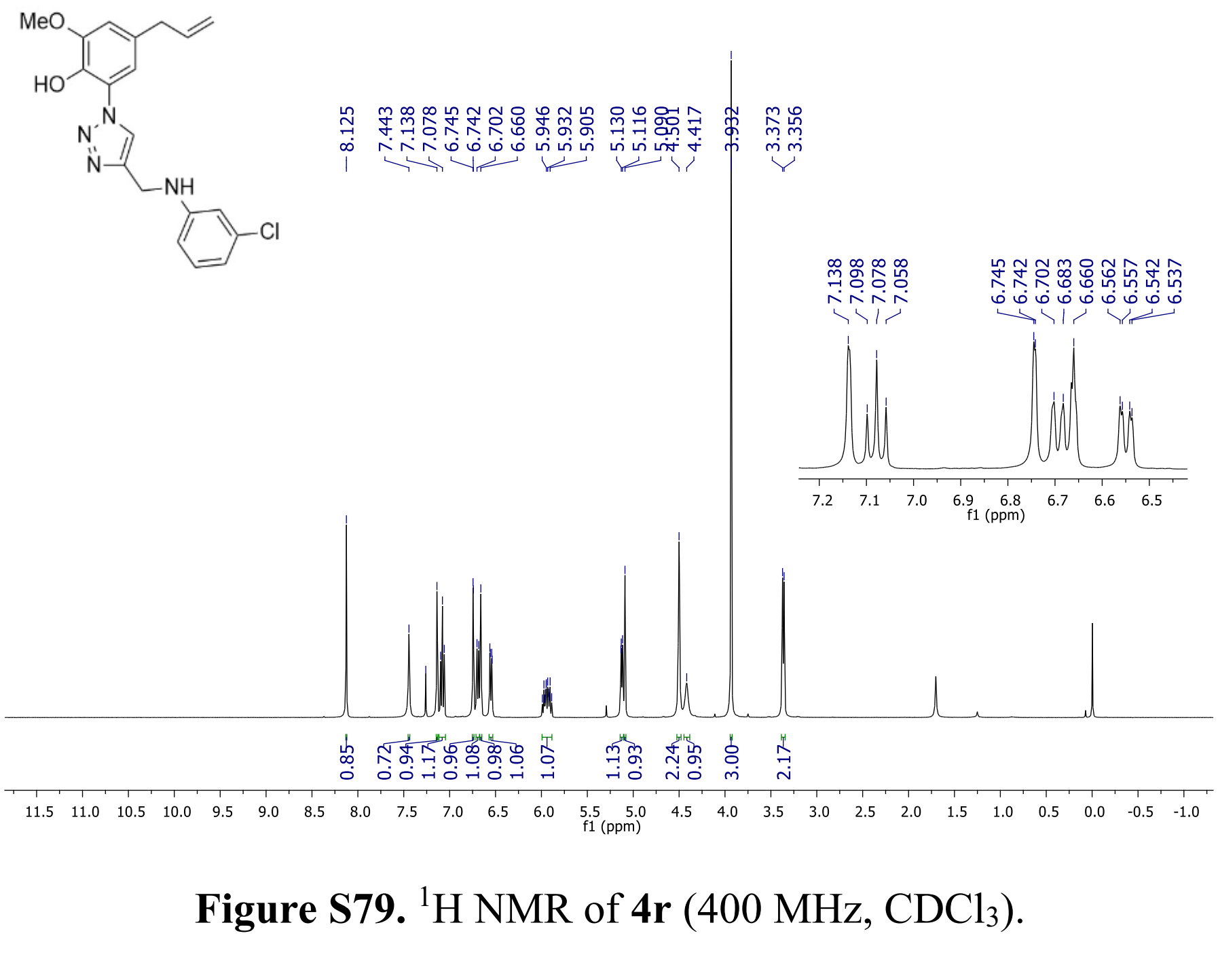

Figure S79. ^{1}H NMR of **4r** (400 MHz, $CDCl_3$).

^{13}C NMR of 4r (101 MHz, CDCl$_3$)

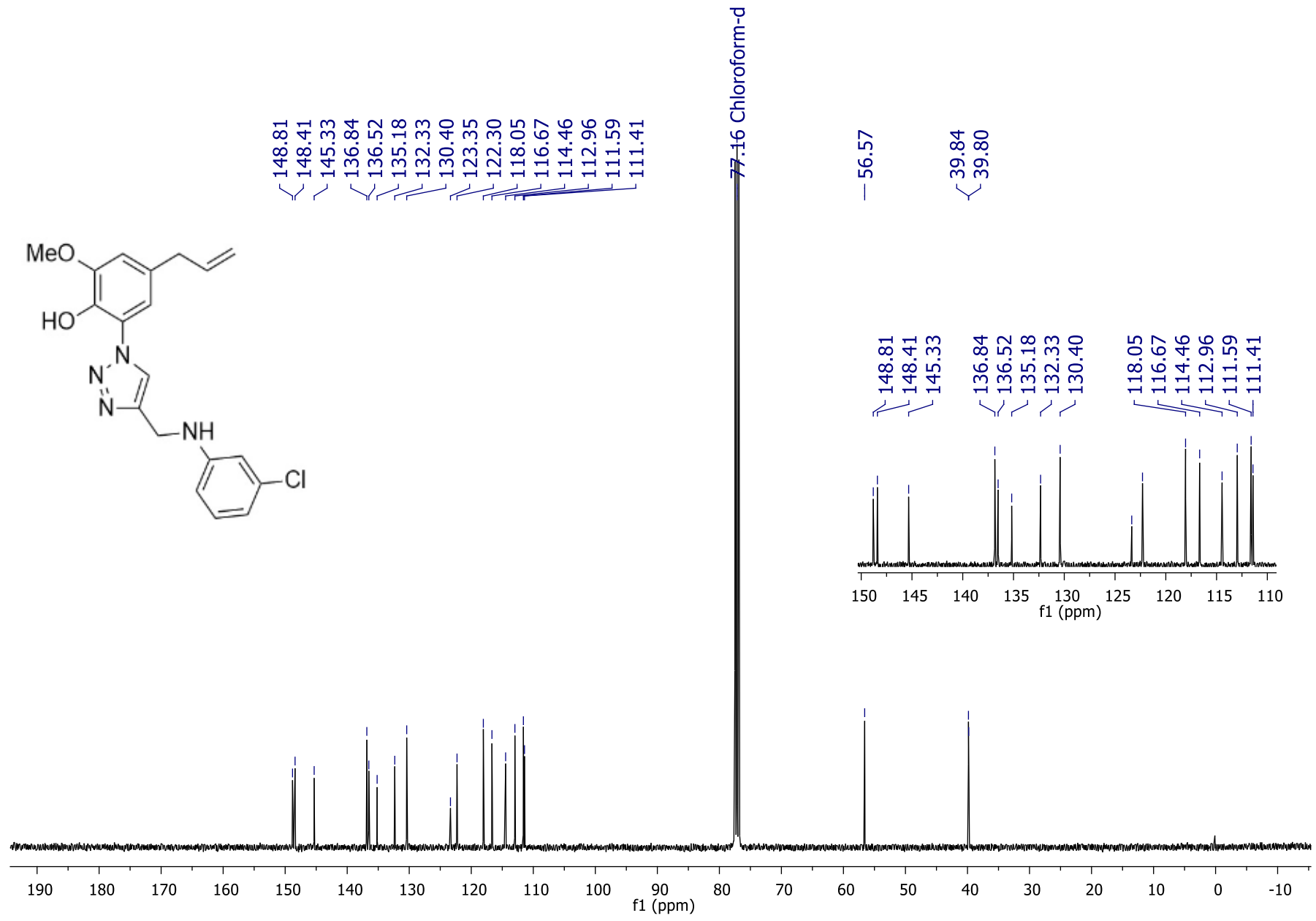

Figure S80. ^{13}C NMR of **4r** (101 MHz, CDCl$_3$).

^{13}C NMR DEPT--135 of 4r (101 MHz, CDCl$_3$)

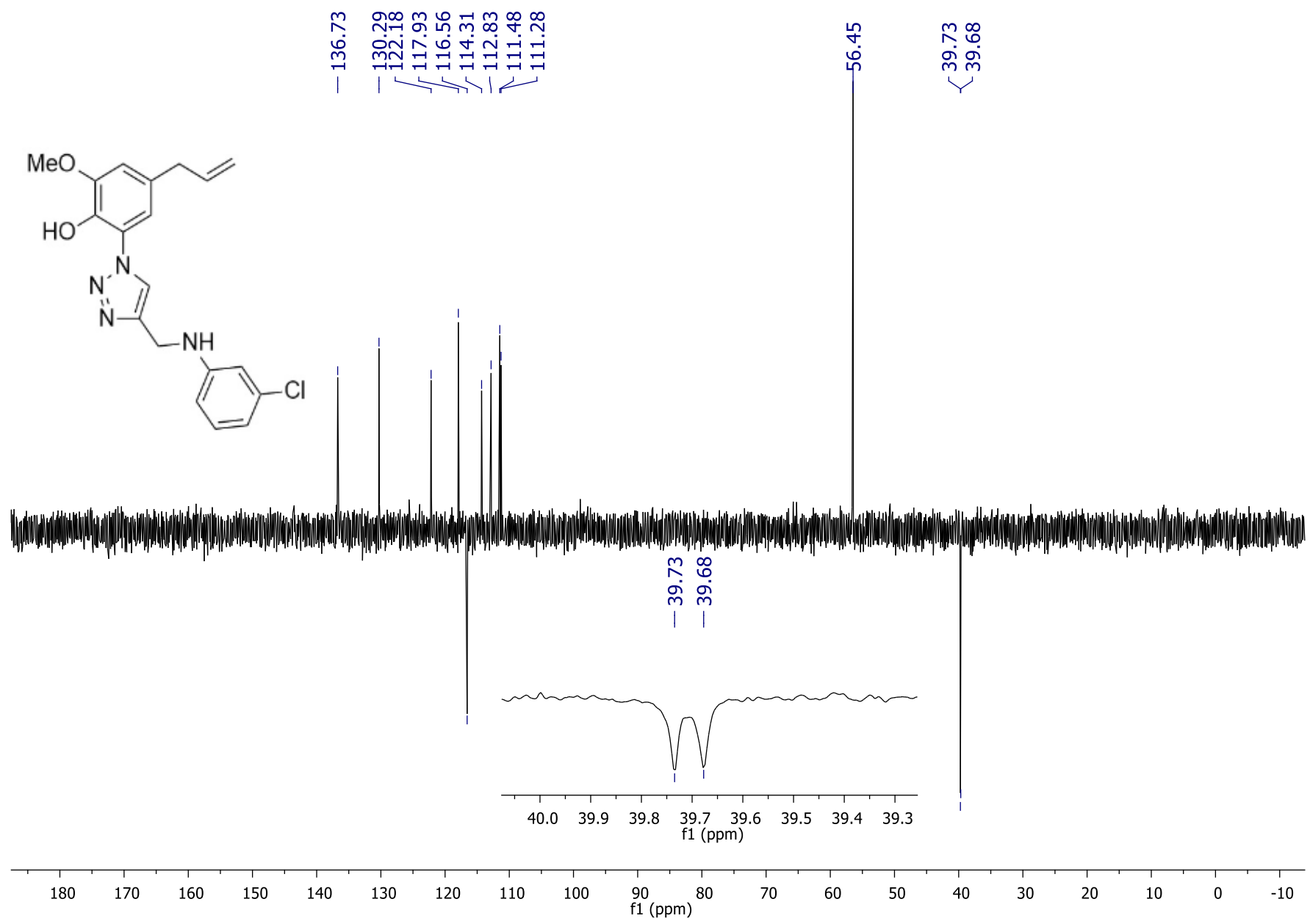

Figure S81. ^{13}C NMR DEPT--135 of **4r** (101 MHz, CDCl$_3$).

$^{1}H/^{13}C$ NMR HSQC of 4r (400 and 101 MHz, $CDCl_3$)

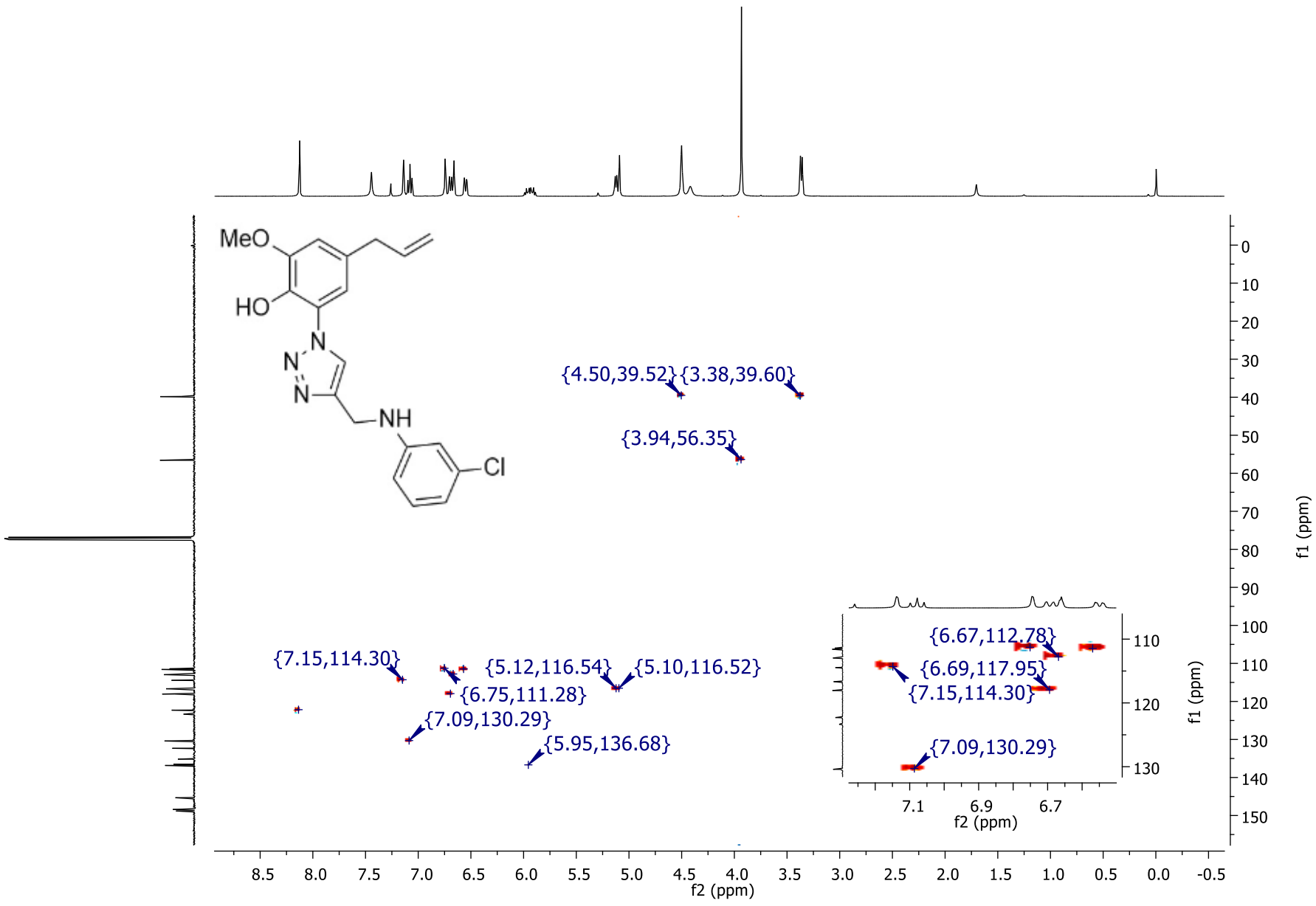

Figure S82. $^{1}H/^{13}C$ NMR HSQC of **4r** (400 and 101 MHz, $CDCl_3$).

^{1}H NMR of 4s (400 MHz, $CDCl_3$)

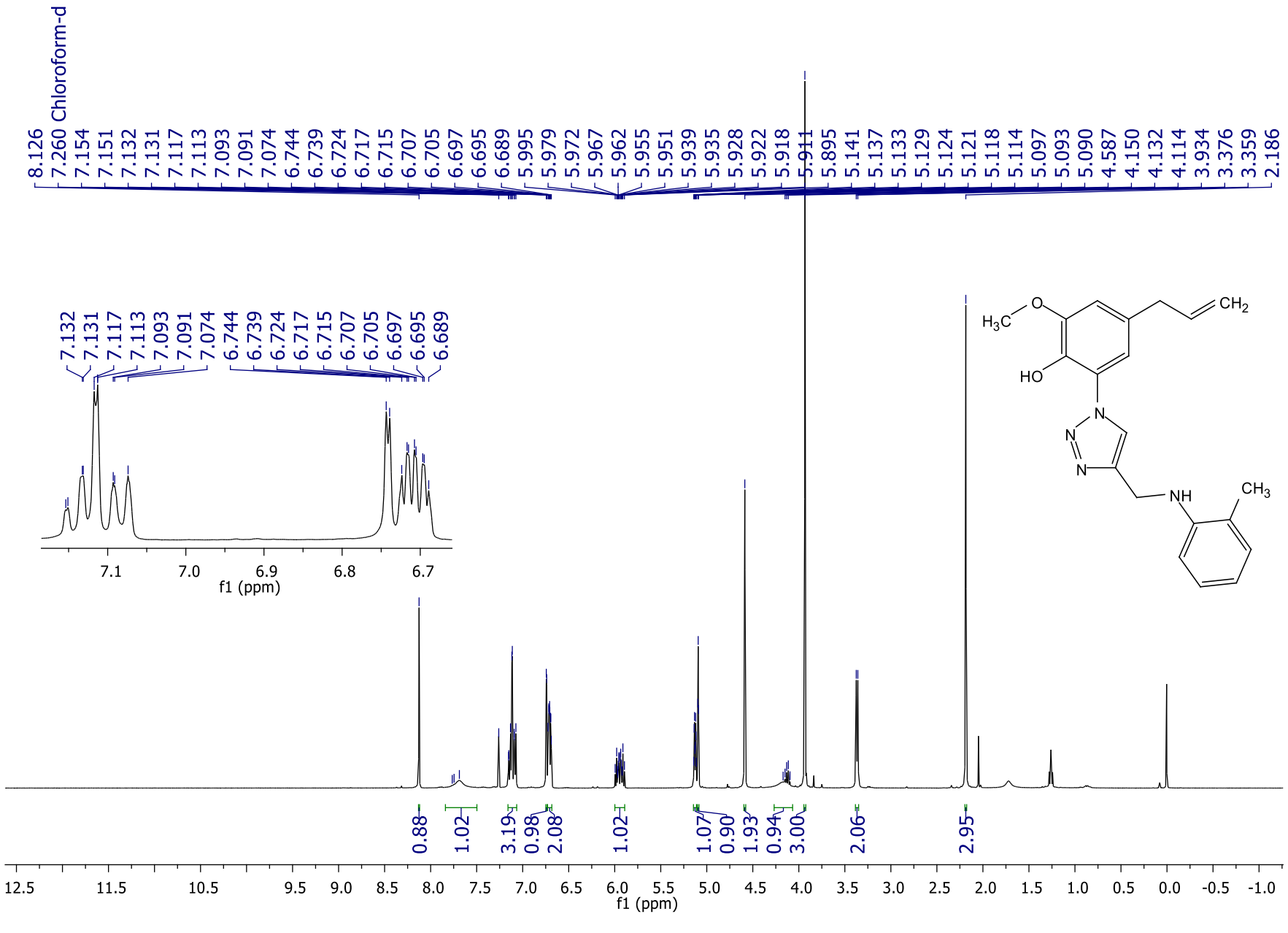

Figure S83. ^{1}H NMR of **4s** (400 MHz, $CDCl_3$).

^{13}C NMR of 4s (101 MHz, $CDCl_3$)

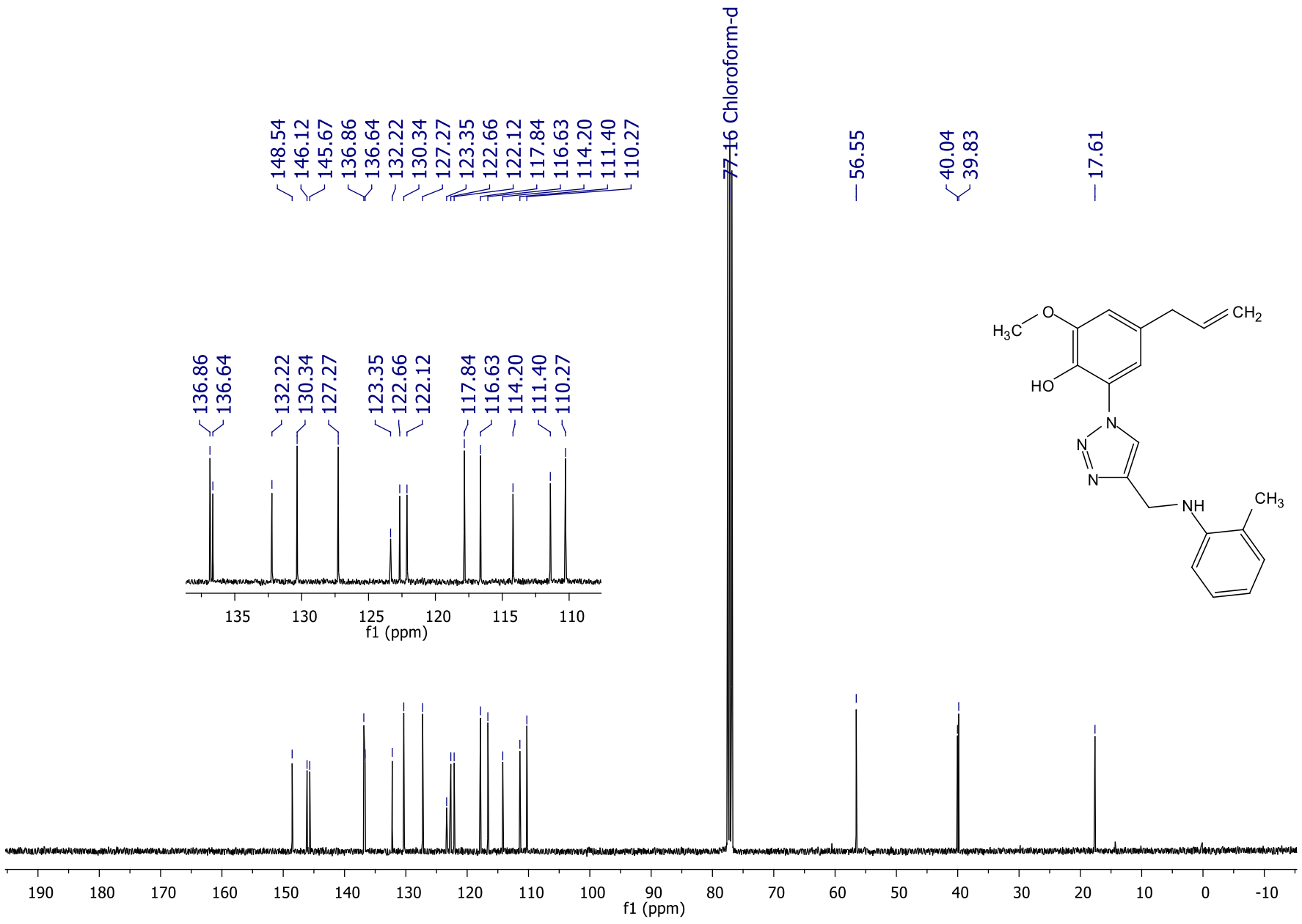

Figure S84. ^{13}C NMR of **4s** (101 MHz, $CDCl_3$).

^{13}C NMR DEPT--135 of 4s (101 MHz, $CDCl_3$)

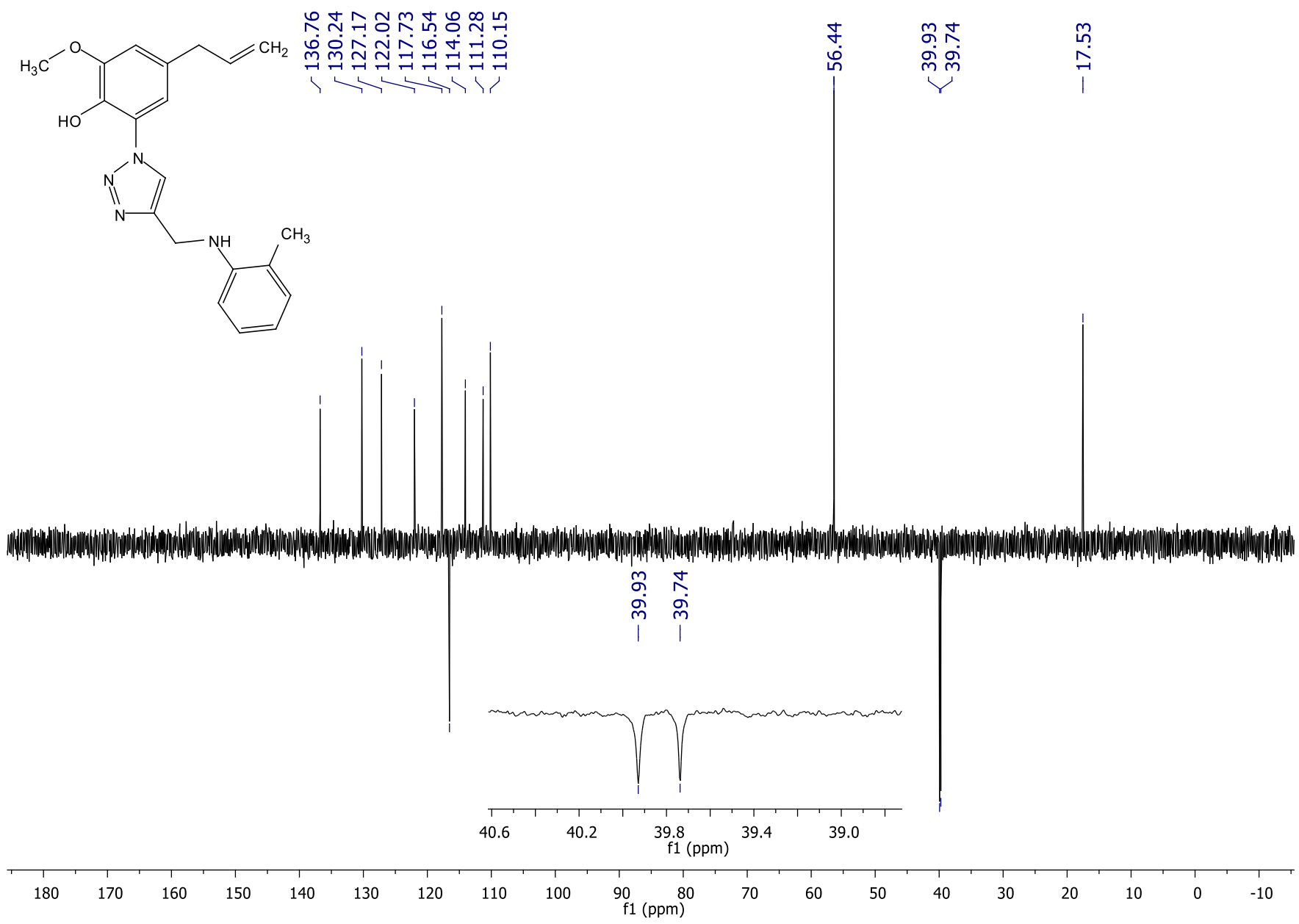

Figure S85. ^{13}C NMR DEPT--135 of **4s** (101 MHz, $CDCl_3$).

$^{1}H/^{13}C$ NMR HSQC of 4s (400 and 101 MHz, $CDCl_3$)

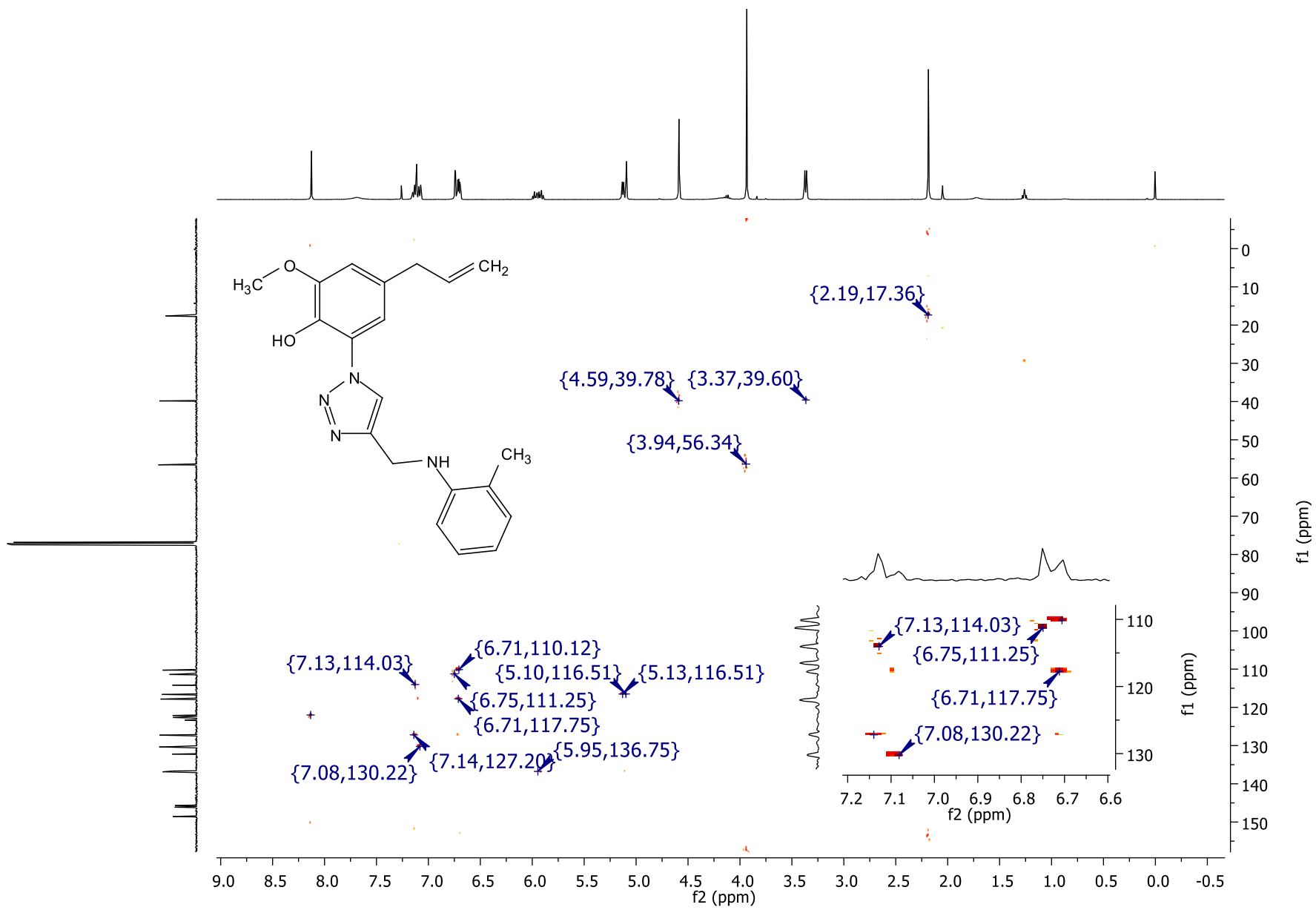

Figure S86. $^{1}H/^{13}C$ NMR HSQC of **4s** (400 and 101 MHz, $CDCl_3$).

^{1}H NMR of 4t (400 MHz, $CDCl_3$)

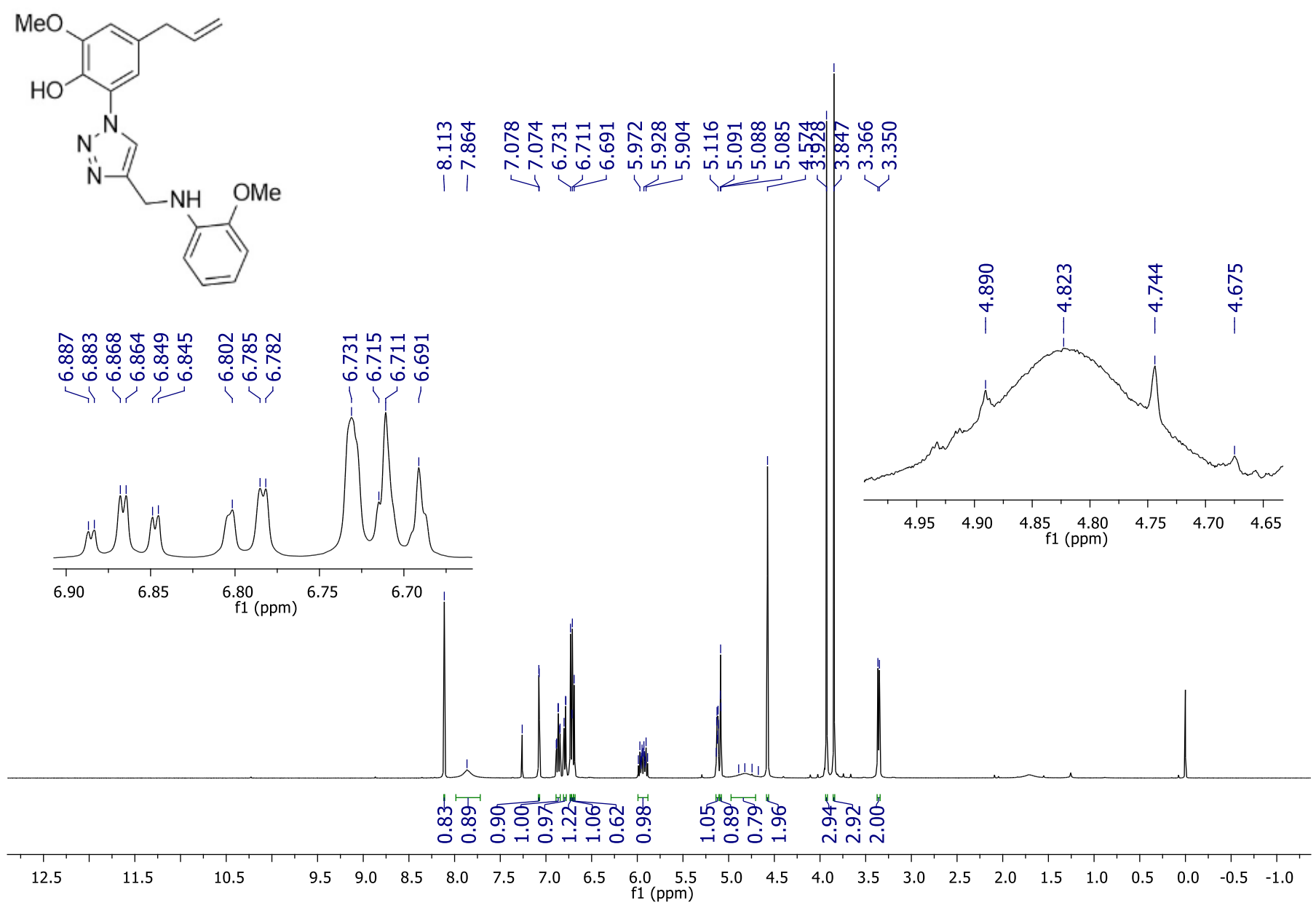

Figure S87. ^{1}H NMR of **4t** (400 MHz, $CDCl_3$).

^{13}C NMR of 4t (101 MHz, $CDCl_3$)

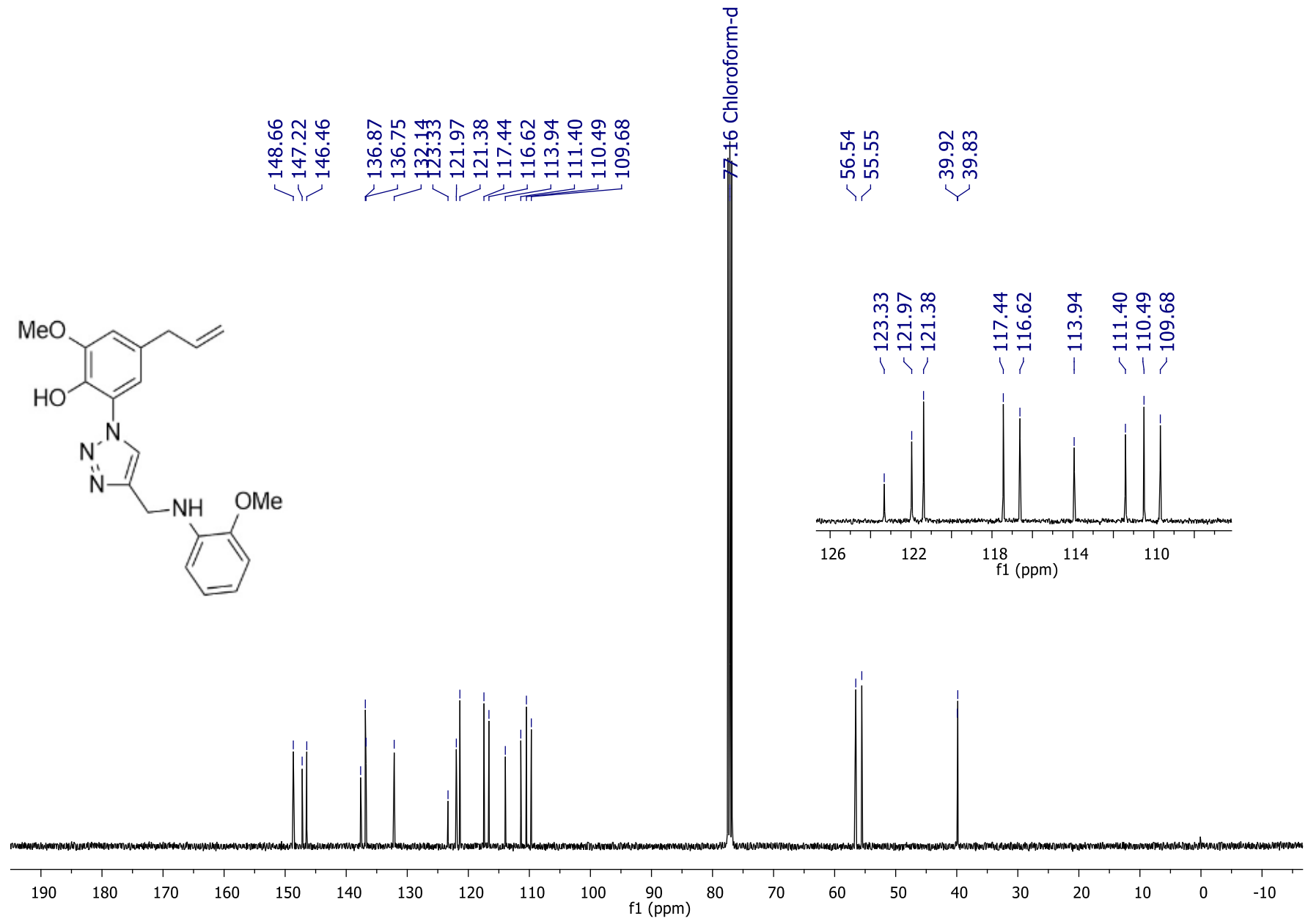

Figure S88. ^{13}C NMR of **4t** (101 MHz, $CDCl_3$).

^{13}C NMR DEPT--135 of 4t (101 MHz, $CDCl_3$)

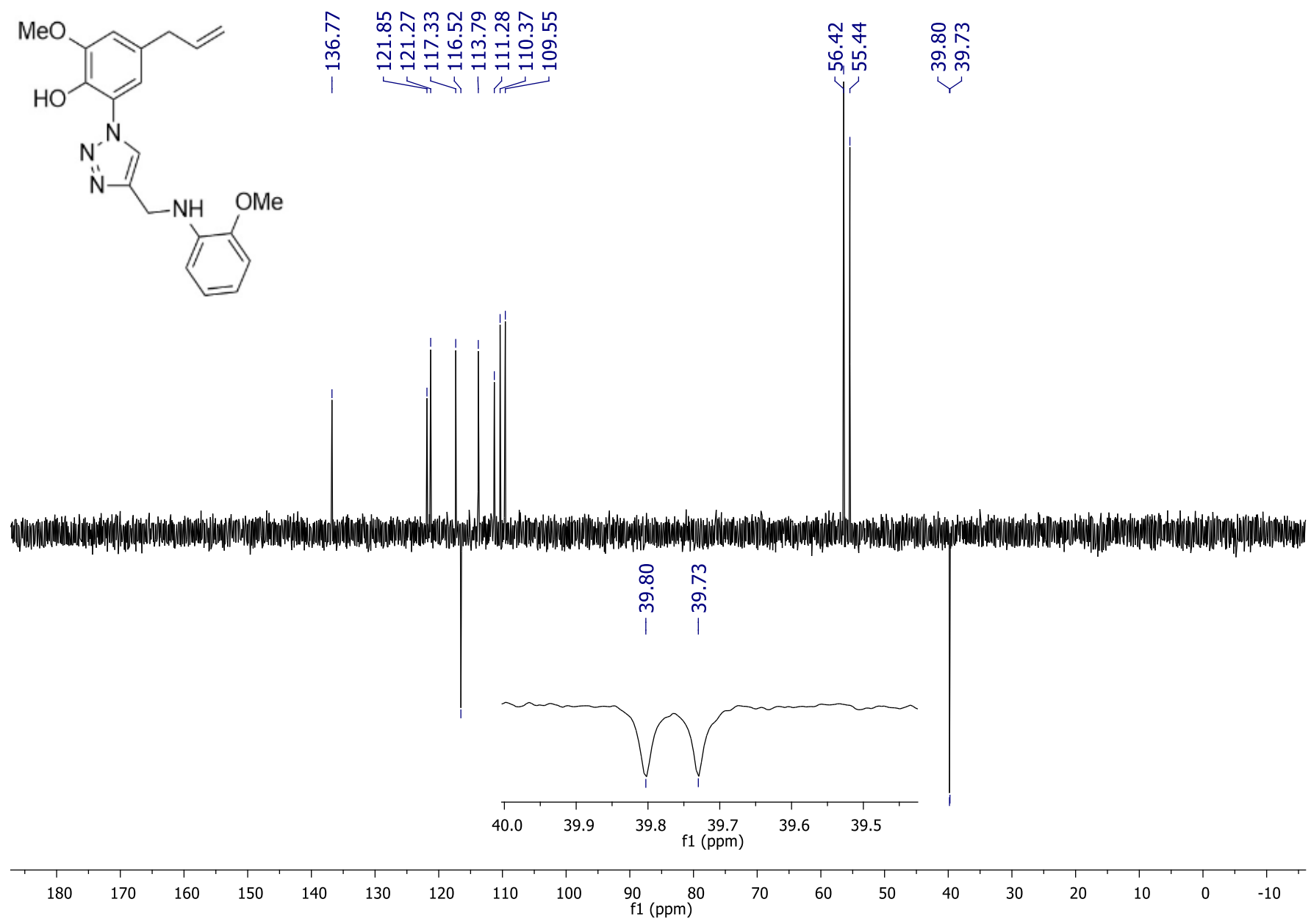

Figure S89. ^{13}C NMR DEPT--135 of **4t** (101 MHz, $CDCl_3$).

$^1H/^{13}C$ NMR HSQC of 4t (400 and 101 MHz, CDCl$_3$)

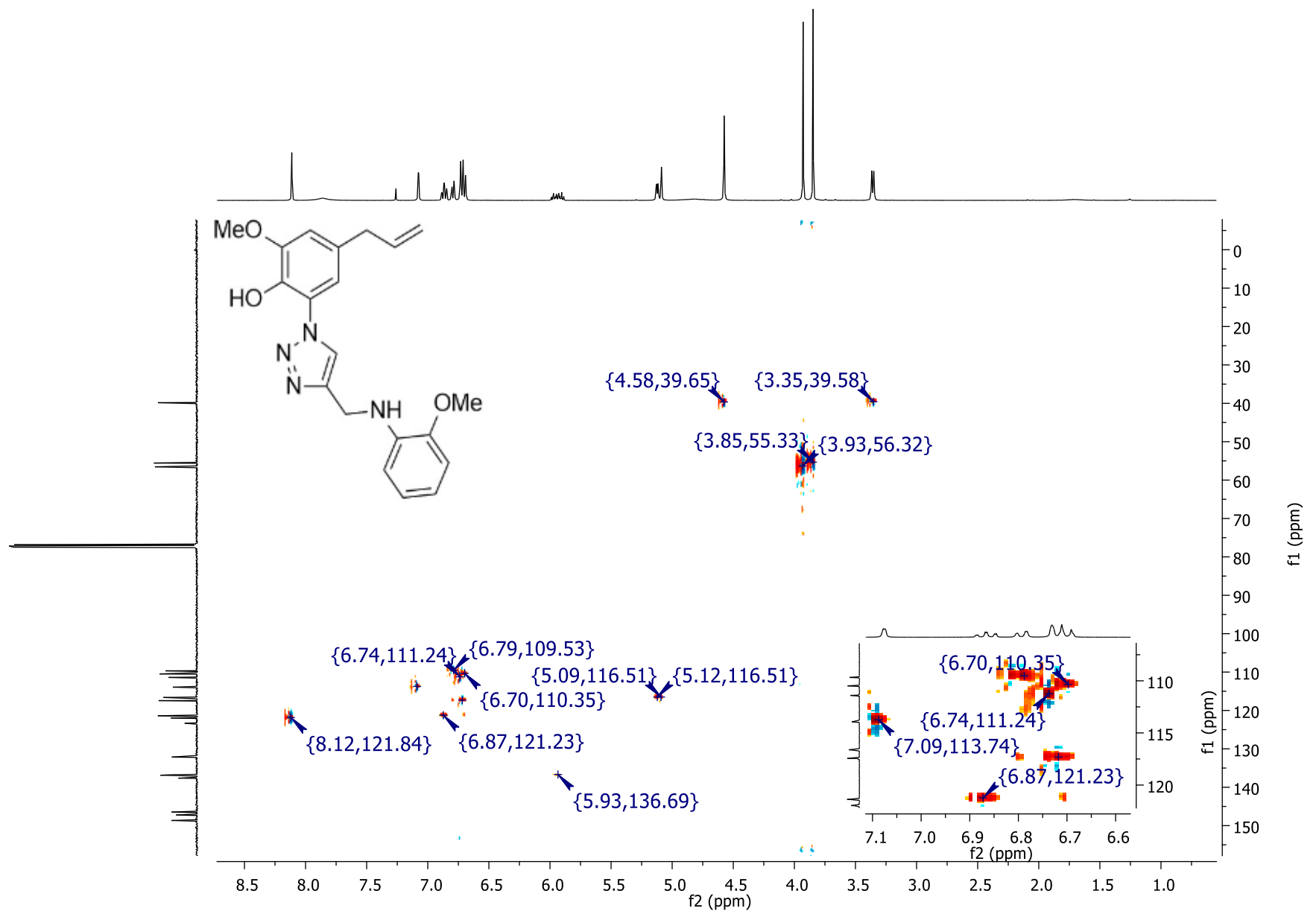

Figure S90. $^1H/^{13}C$ NMR HSQC of **4t** (400 and 101 MHz, CDCl$_3$).

^1H NMR of 5 (400 MHz, DMSO)

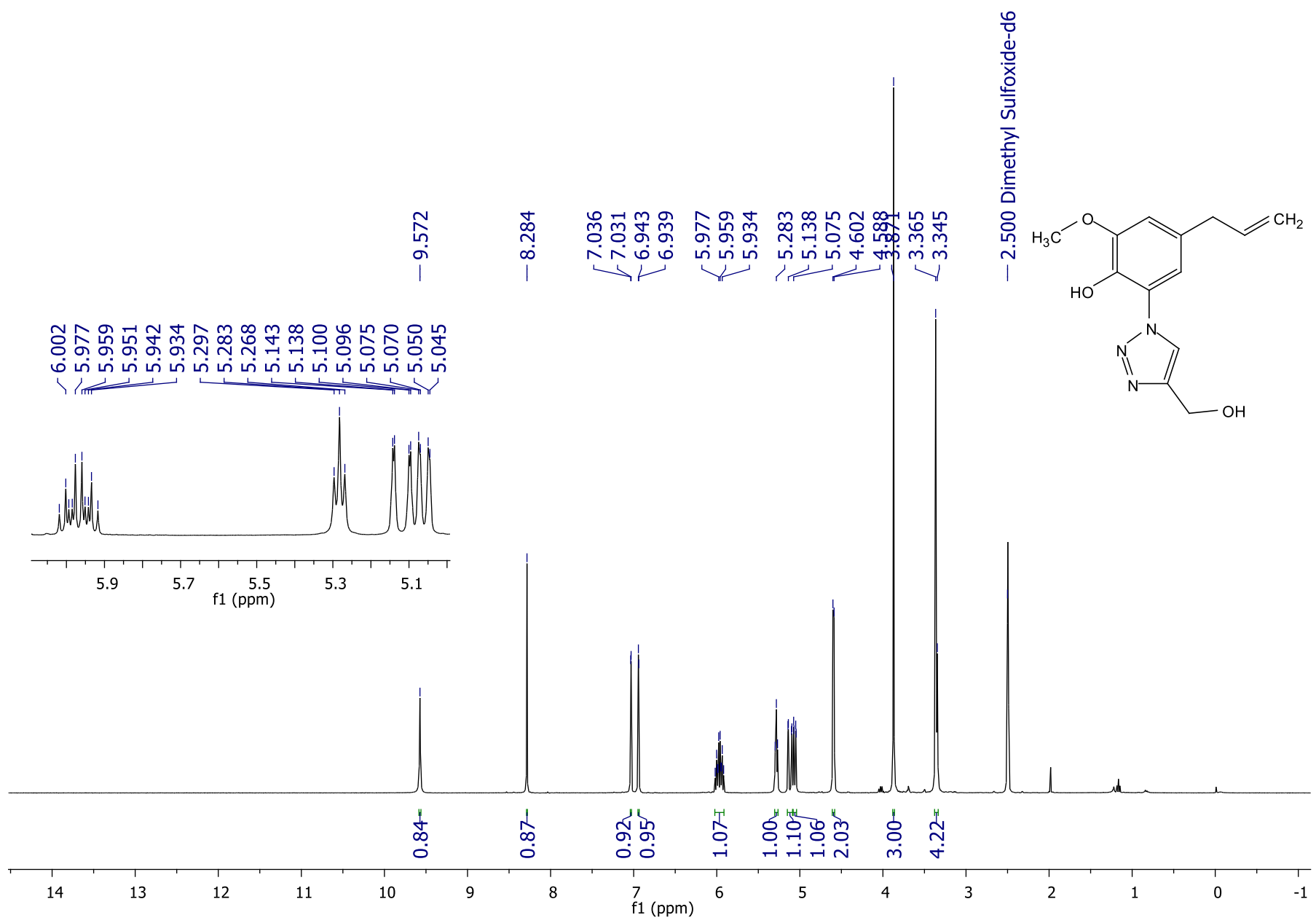

Figure S91. ^1H NMR of **5** (400 MHz, DMSO).

^{1}H NMR of 5 (400 MHz, CD$_3$OD)

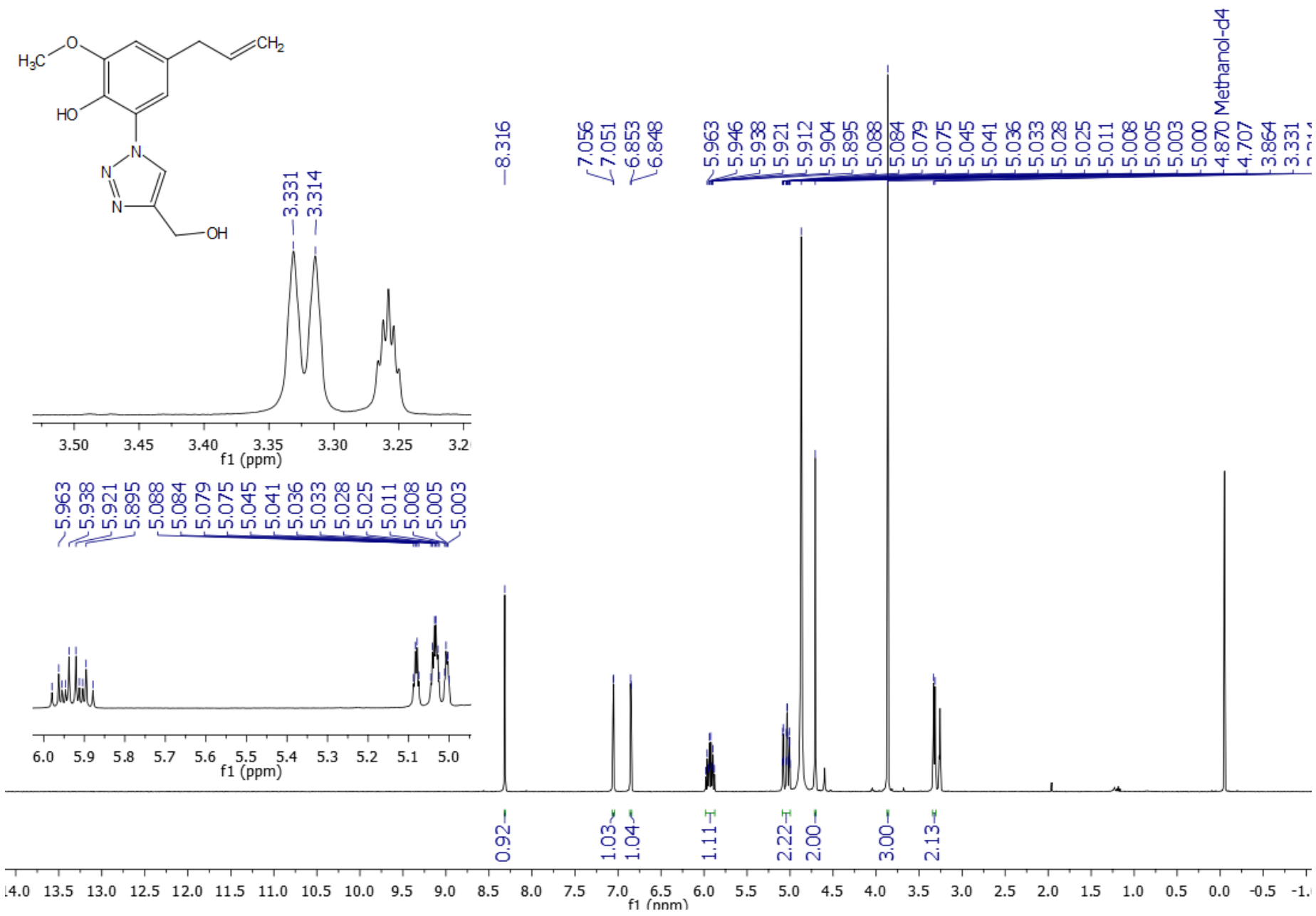

Figure S92. ^{1}H NMR of **5** (400 MHz, CD$_3$OD).

^{13}C NMR of 5 (101 MHz, DMSO)

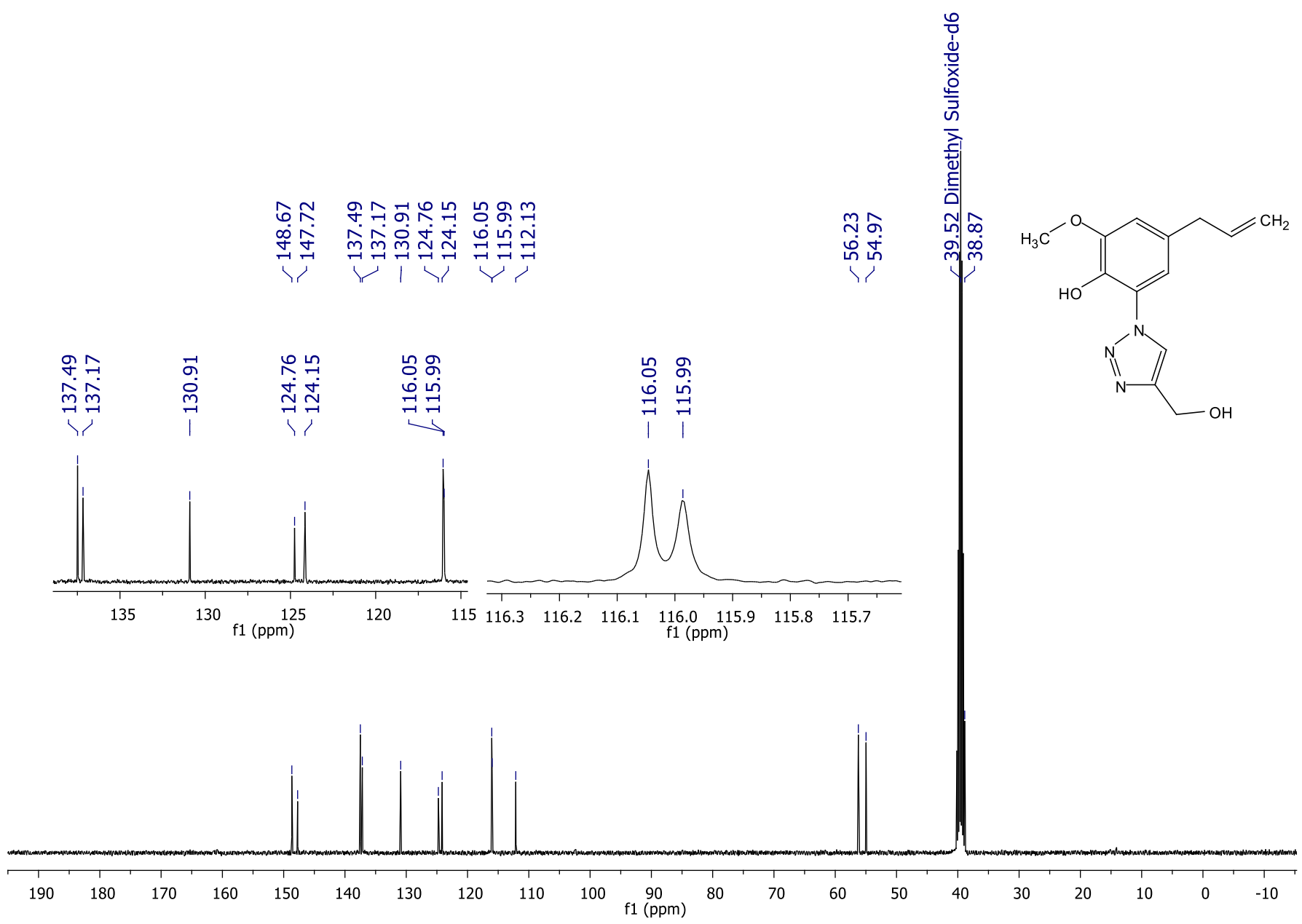

Figure S93. ^{13}C NMR of **5** (101 MHz, DMSO).

^{13}C NMR DEPT--135 of 5 (101 MHz, DMSO)

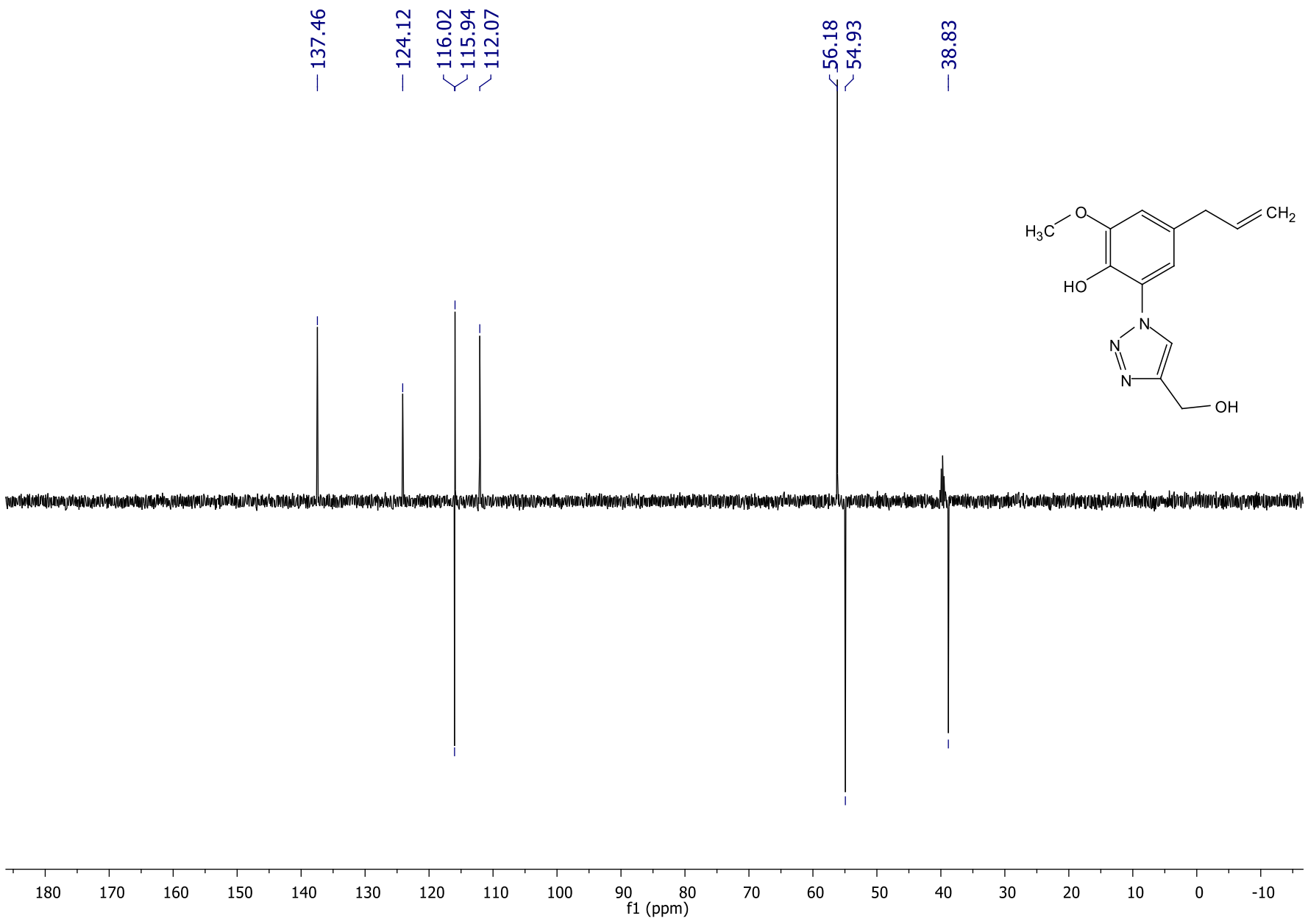

Figure S94. ^{13}C NMR DEPT--135 of **5** (101 MHz, DMSO).

APPENDIX E: High Resolution Mass Spectrometry (ISE) Spectras:

Equipment used: ESI-TOF; Analysis: MS; Analysis mode: positive. Calculations performed with the online mass calculator (Available at <https://warwick.ac.uk/fac/sci/chemistry/research/barrow/barrowgroup/calculators/mass_errors/>, accessed on 20/02/2022).

Ionization Spectrum of 4a (HRMS ESI-TOF)

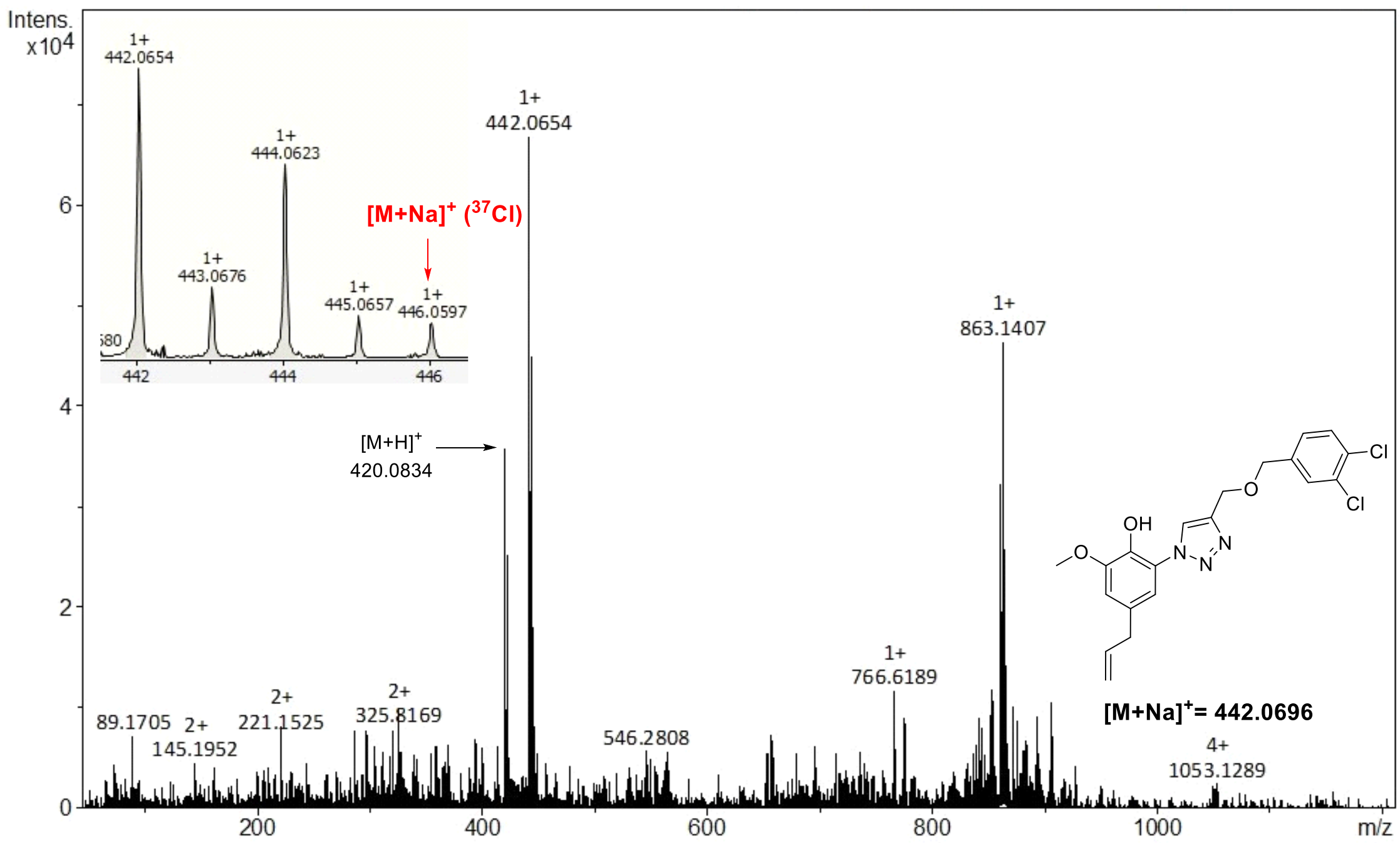

Figure S95. Ionization Spectrum of **4a** (HRMS ESI-TOF).

Ionization Spectrum of 4b (HRMS ESI-TOF)

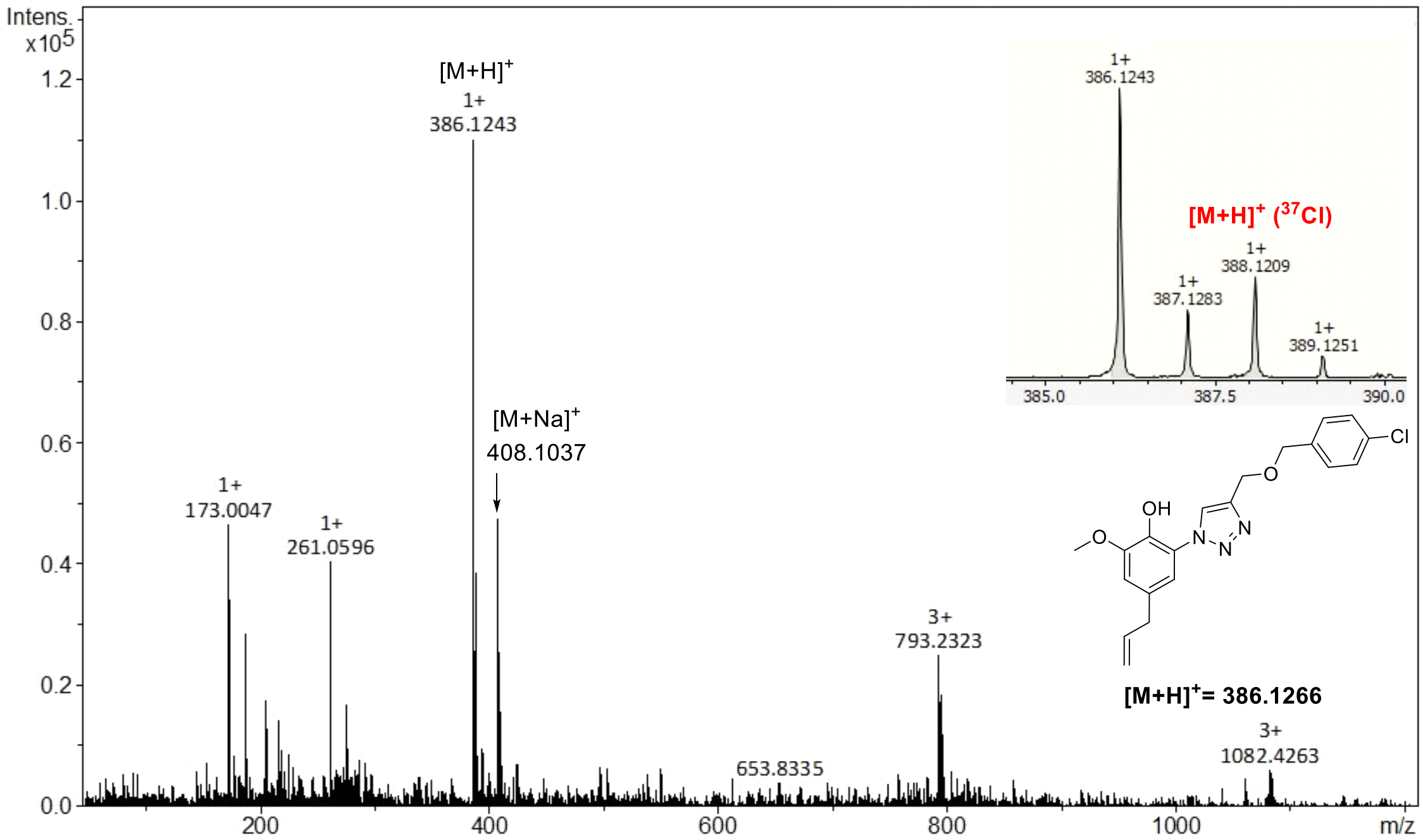

Figure S96. Ionization Spectrum of **4b** (HRMS ESI-TOF).

Ionization Spectrum of 4c (HRMS ESI-TOF)

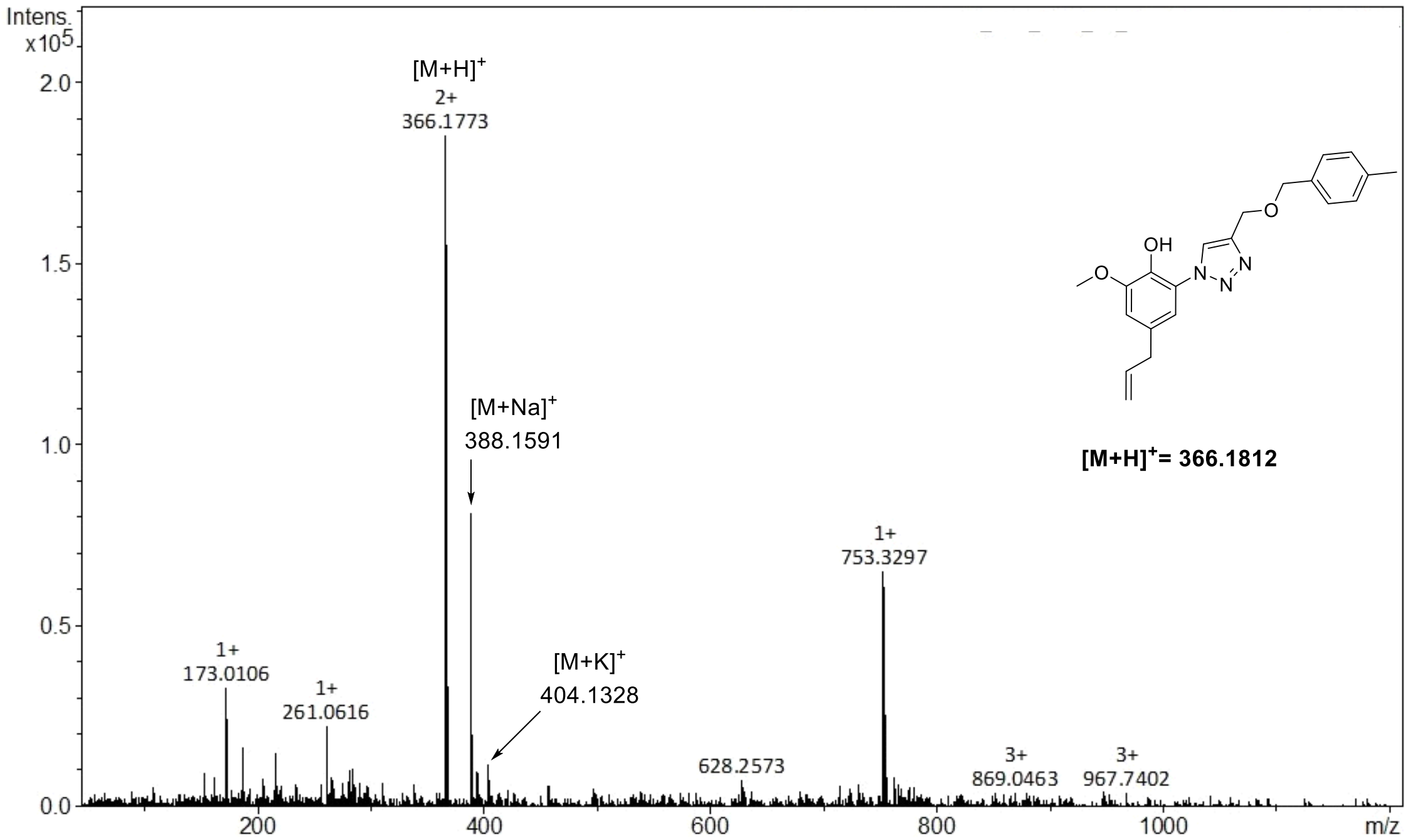

Figure S97. Ionization Spectrum of **4c** (HRMS ESI-TOF).

Ionization Spectrum of 4d (HRMS ESI-TOF)

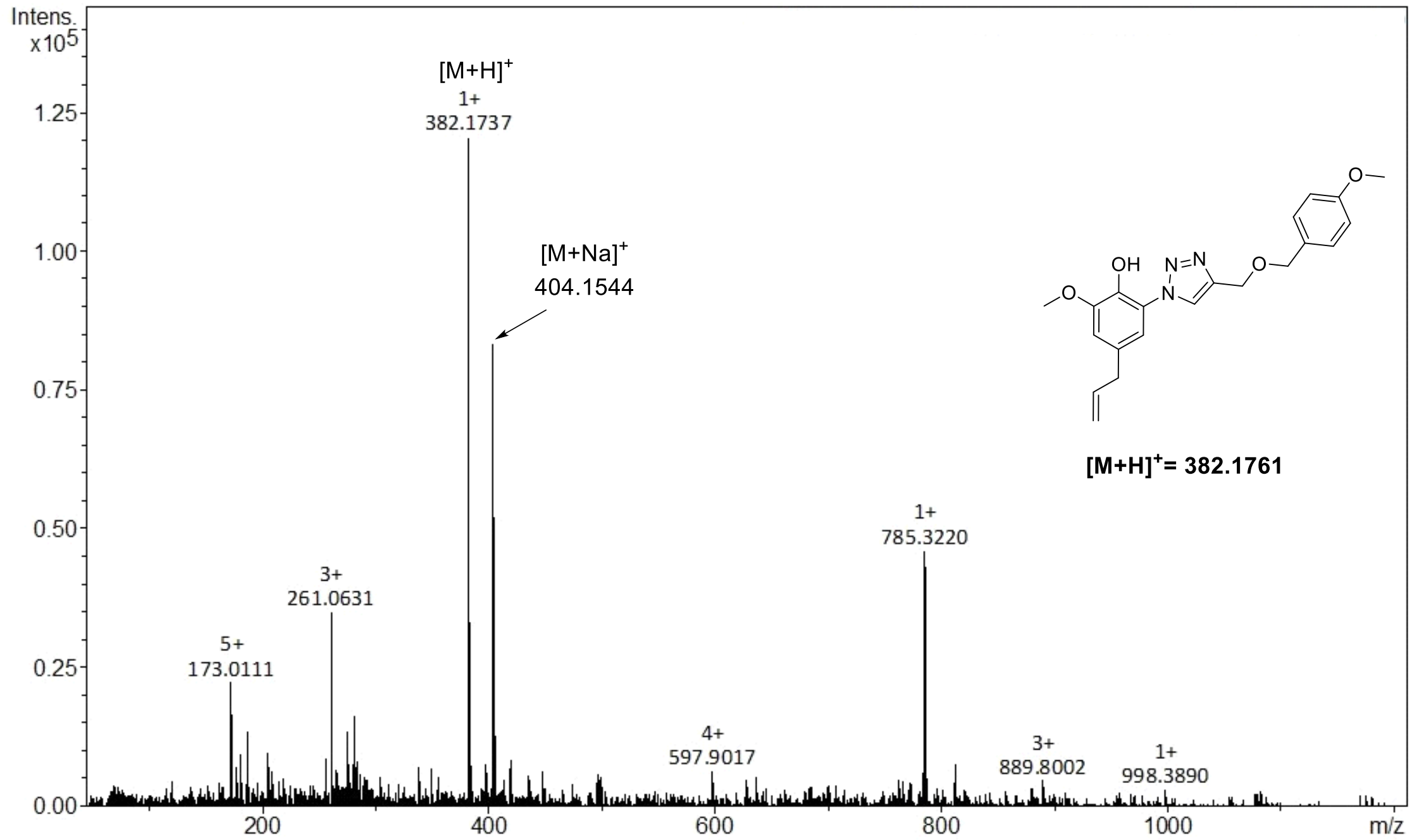

Figure S98. Ionization Spectrum of **4d** (HRMS ESI-TOF).

Ionization Spectrum of 4e (HRMS ESI-TOF)

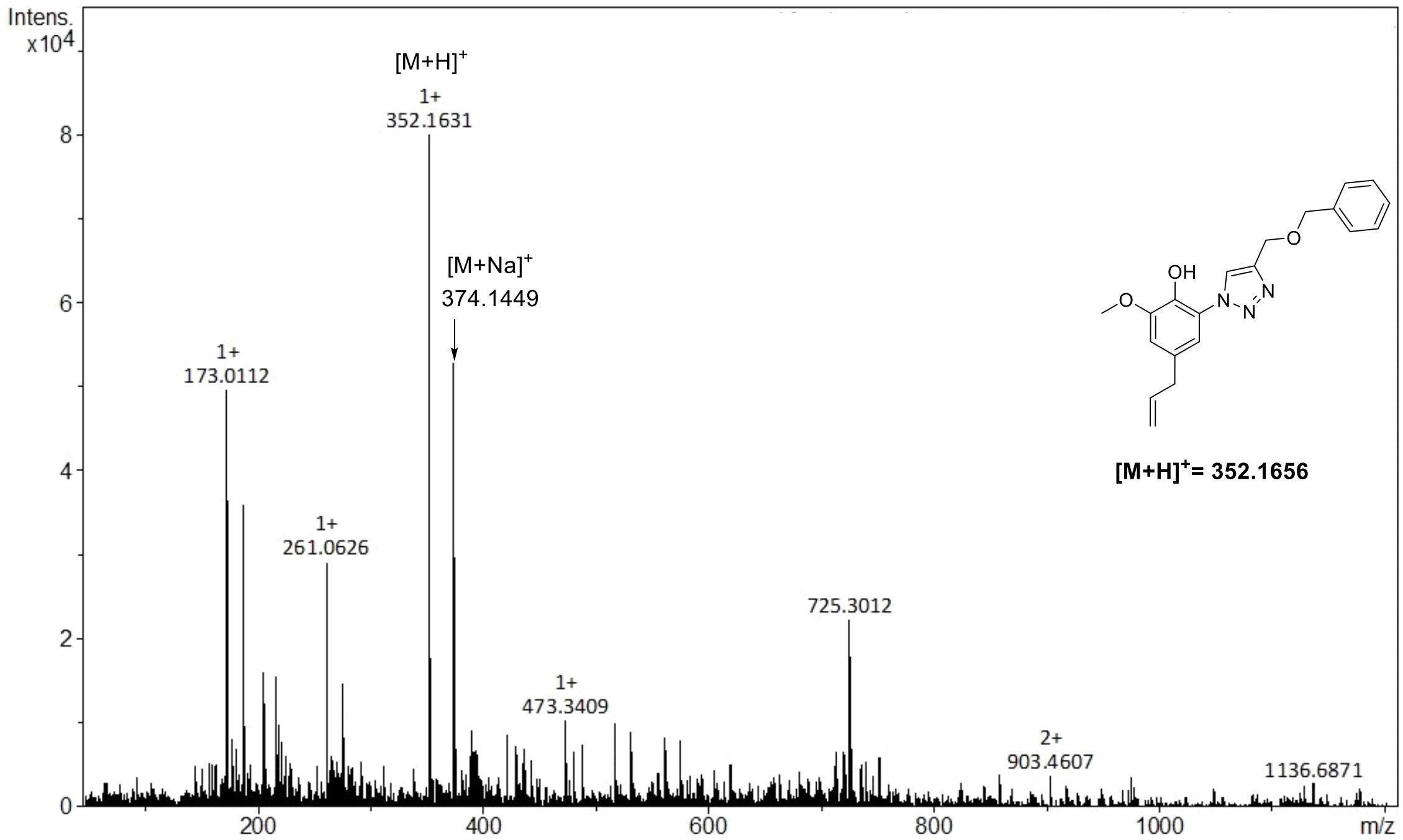

Figure S99. Ionization Spectrum of **4e** (HRMS ESI-TOF).

Ionization Spectrum of 4f (HRMS ESI-TOF)

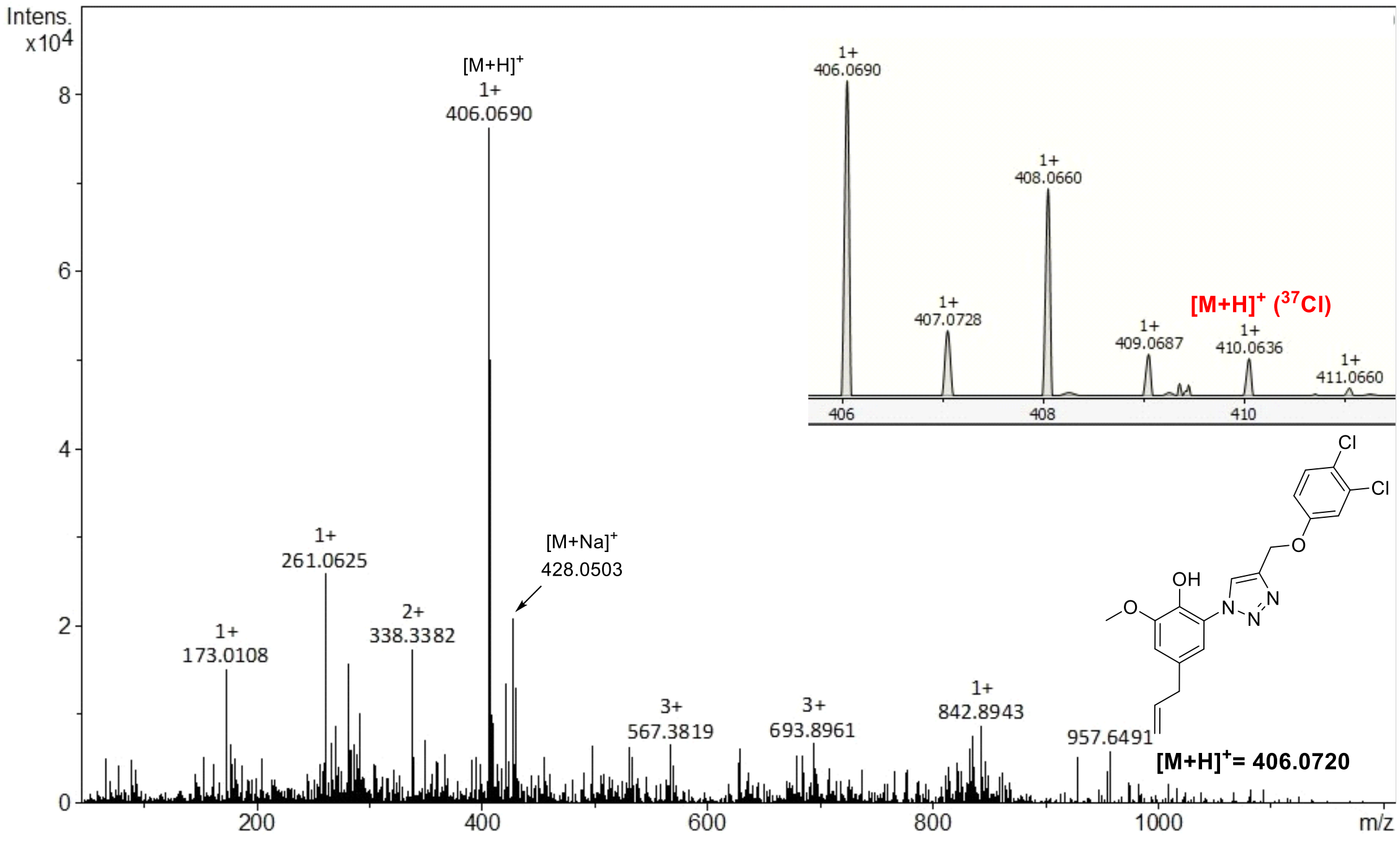

Figure S100. Ionization Spectrum of **4f** (HRMS ESI-TOF).

Ionization Spectrum of 4g (HRMS ESI-TOF)

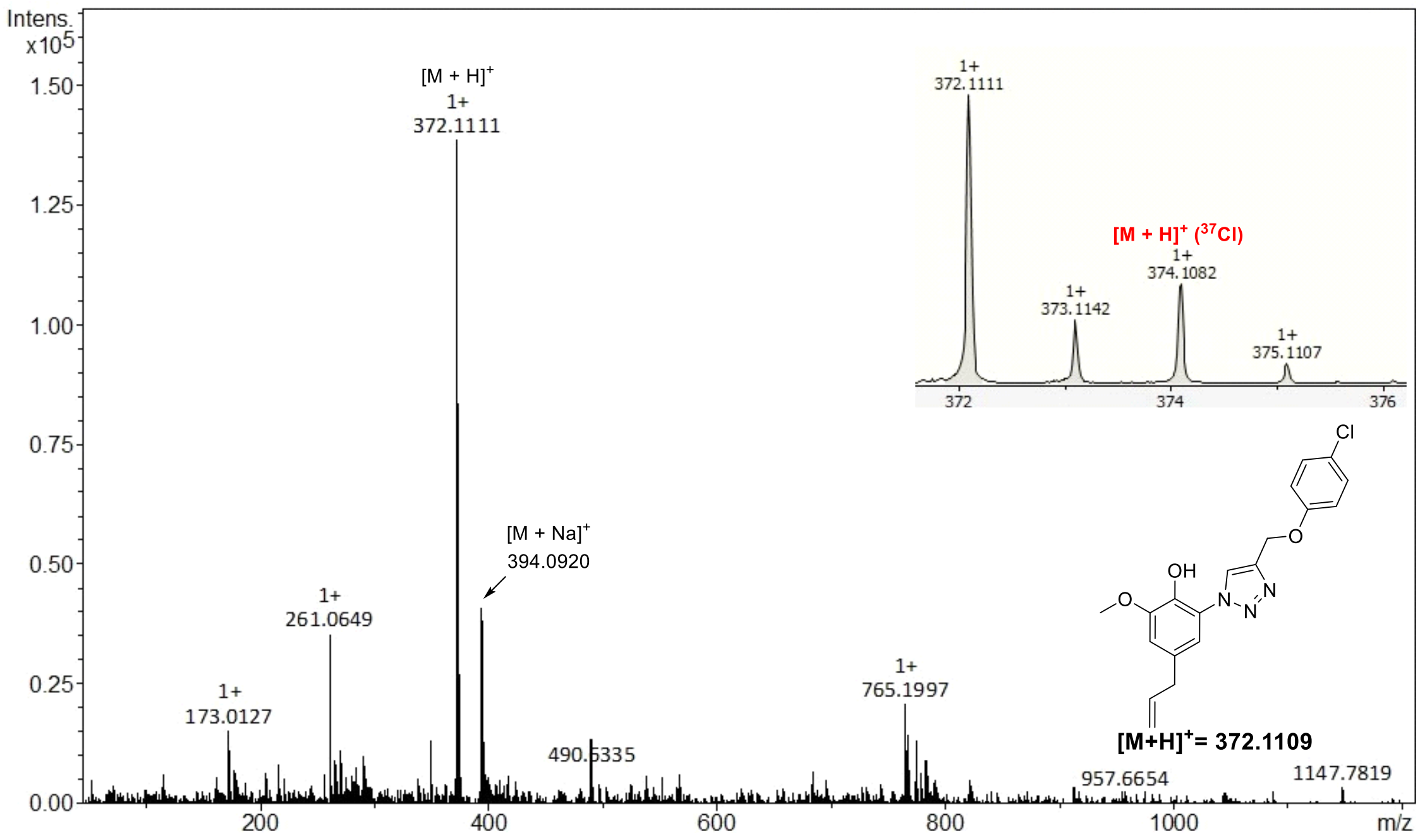

Figure S101. Ionization Spectrum of **4g** (HRMS ESI-TOF).

Ionization Spectrum of 4h (HRMS ESI-TOF)

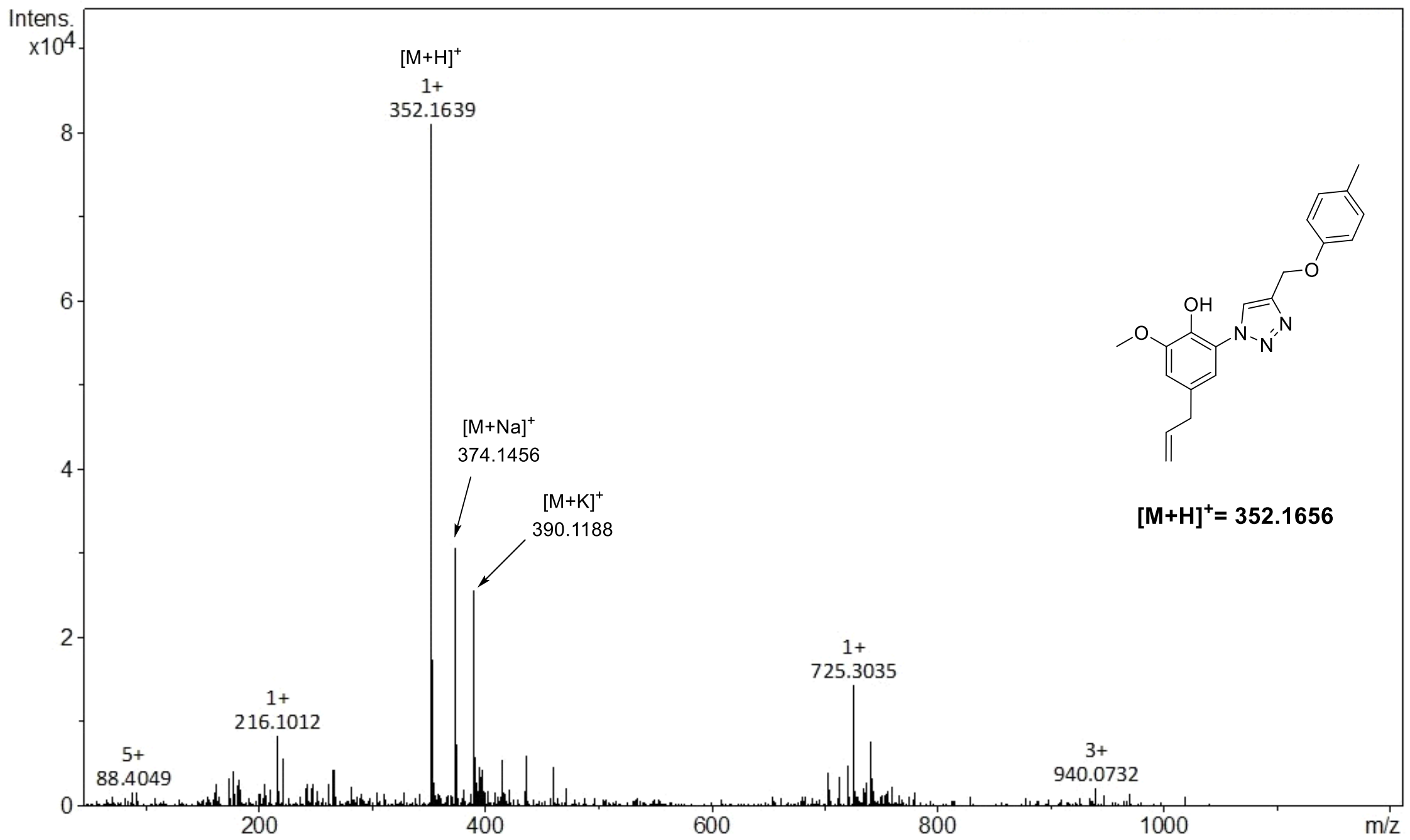

Figure S102. Ionization Spectrum of **4h** (HRMS ESI-TOF).

Ionization Spectrum of 4i (HRMS ESI-TOF)

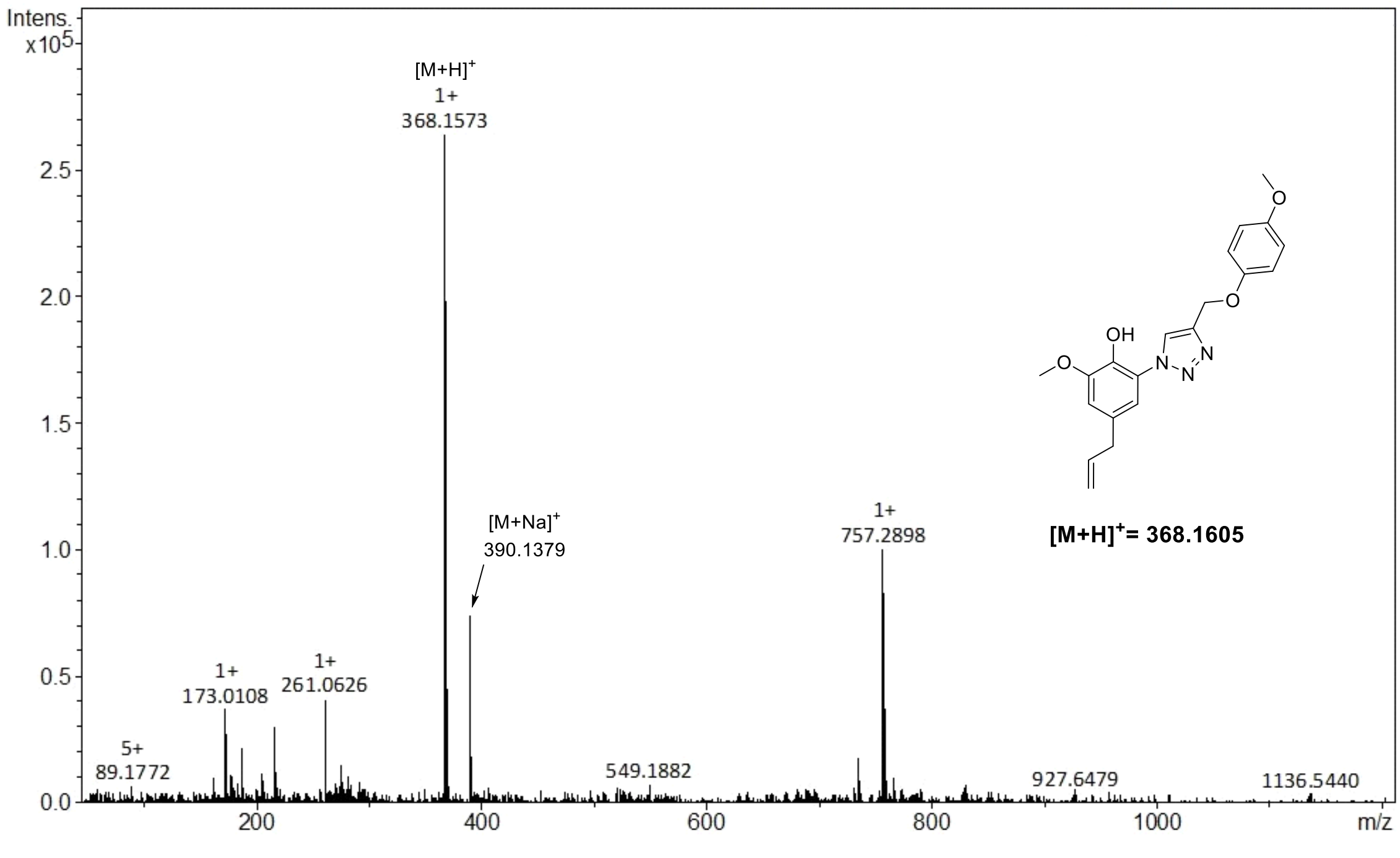

Figure S103. Ionization Spectrum of **4i** (HRMS ESI-TOF).

Ionization Spectrum of 4j (HRMS ESI-TOF)

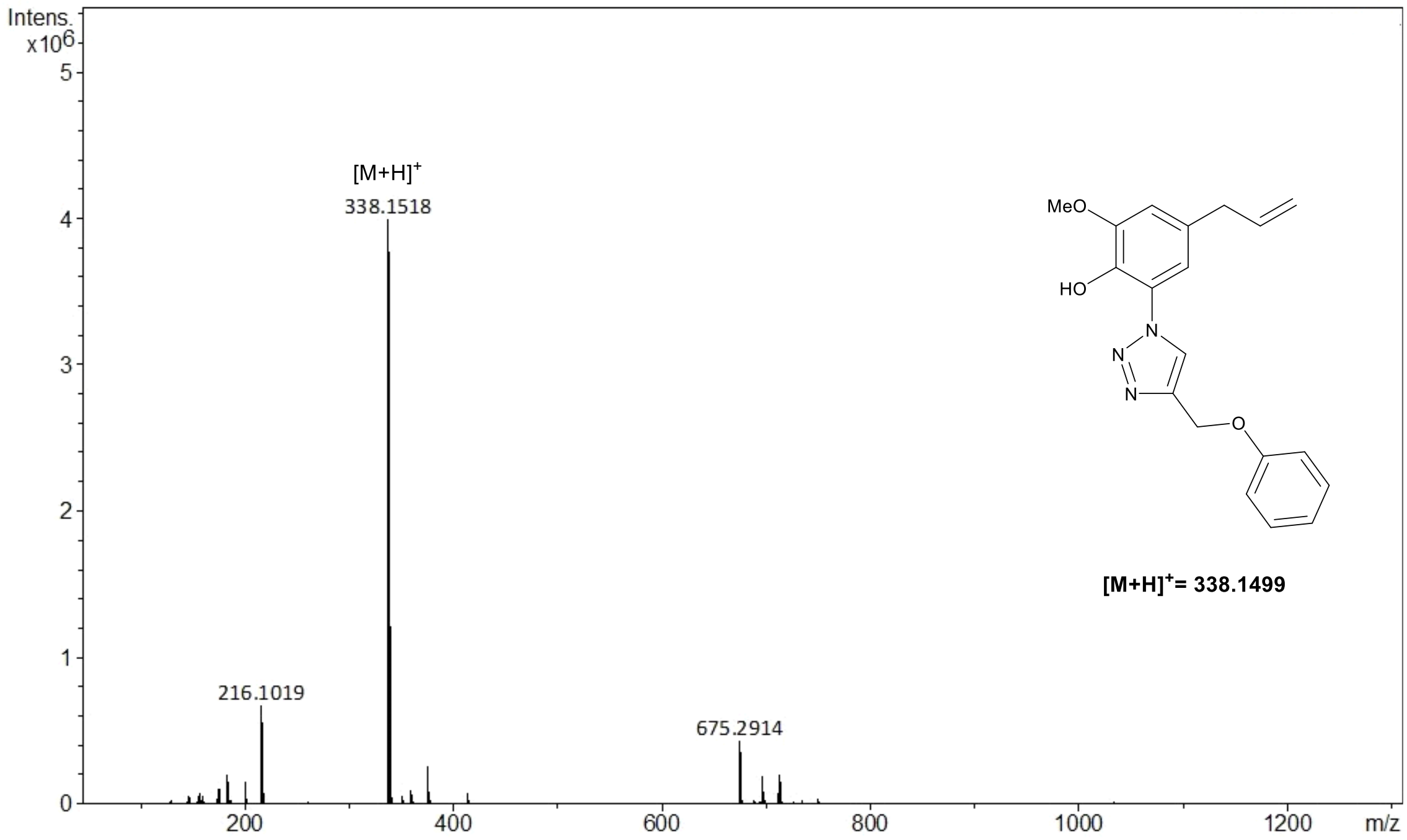

Figure S104. Ionization Spectrum of **4j** (HRMS ESI-TOF).

Ionization Spectrum of 4k (HRMS ESI-TOF)

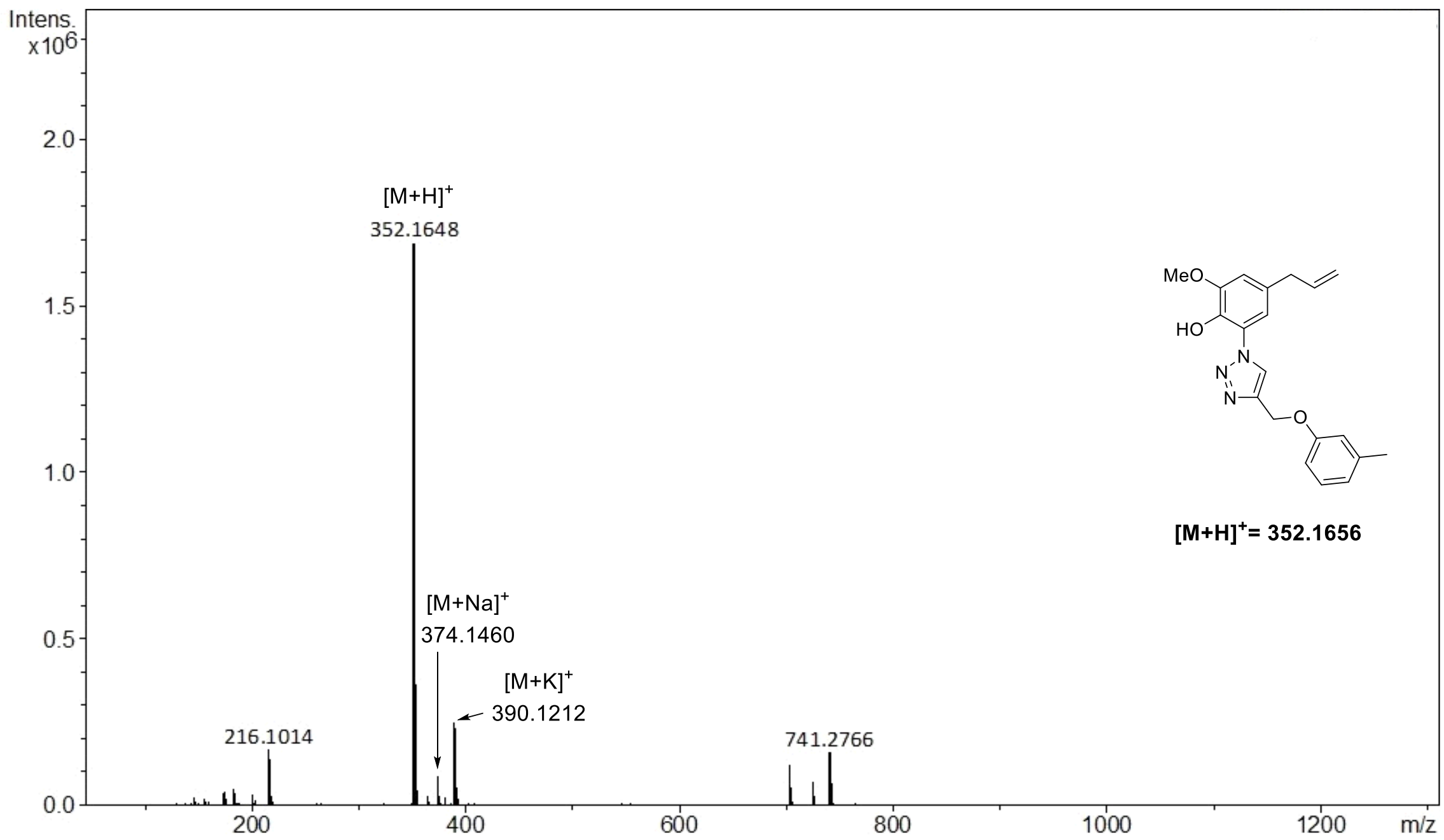

Figure S105. Ionization Spectrum of **4k** (HRMS ESI-TOF).

Ionization Spectrum of 4l (HRMS ESI-TOF)

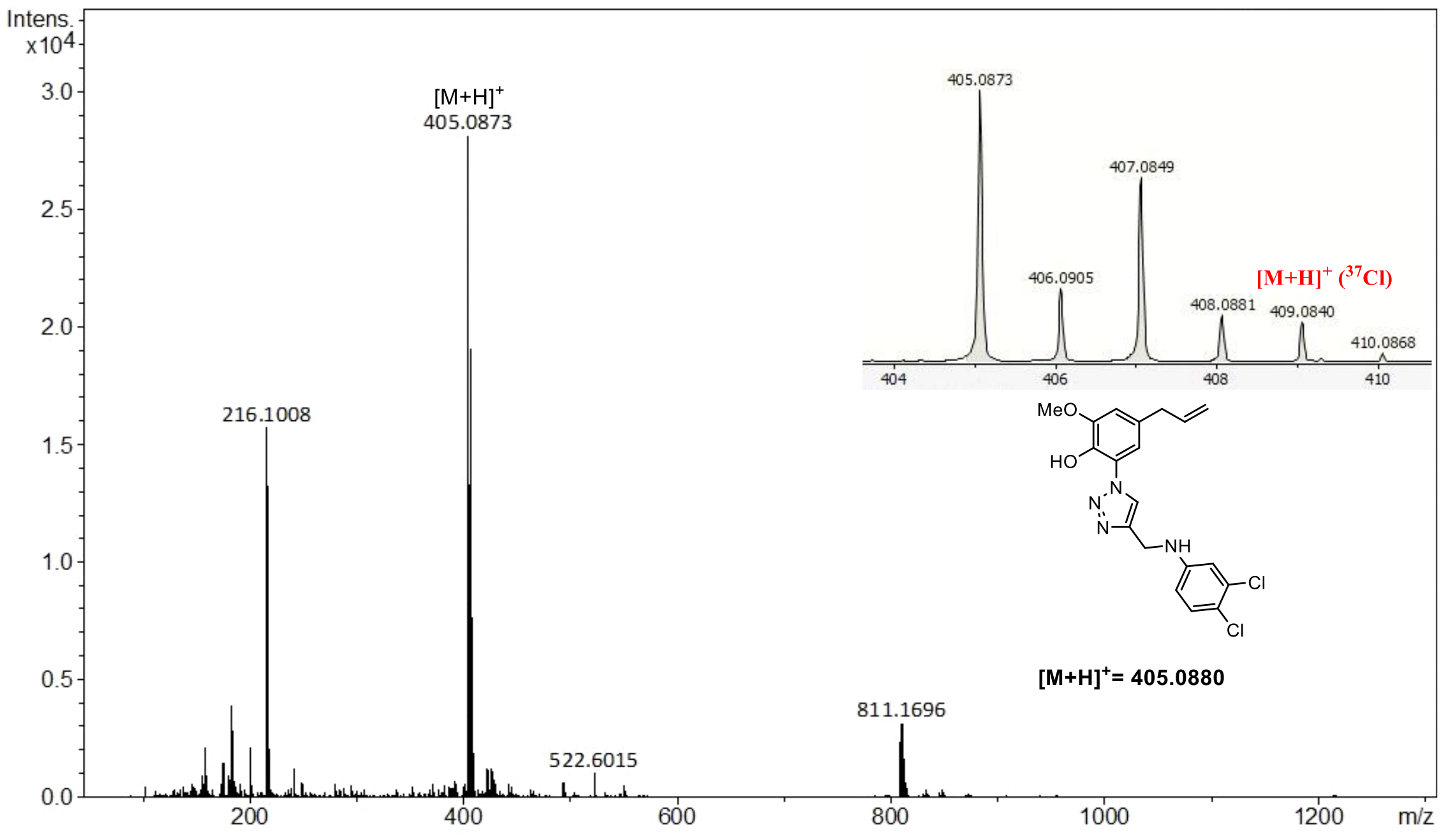

Figure S106. Ionization Spectrum of **4l** (HRMS ESI-TOF).

Ionization Spectrum of 4m (HRMS ESI-TOF)

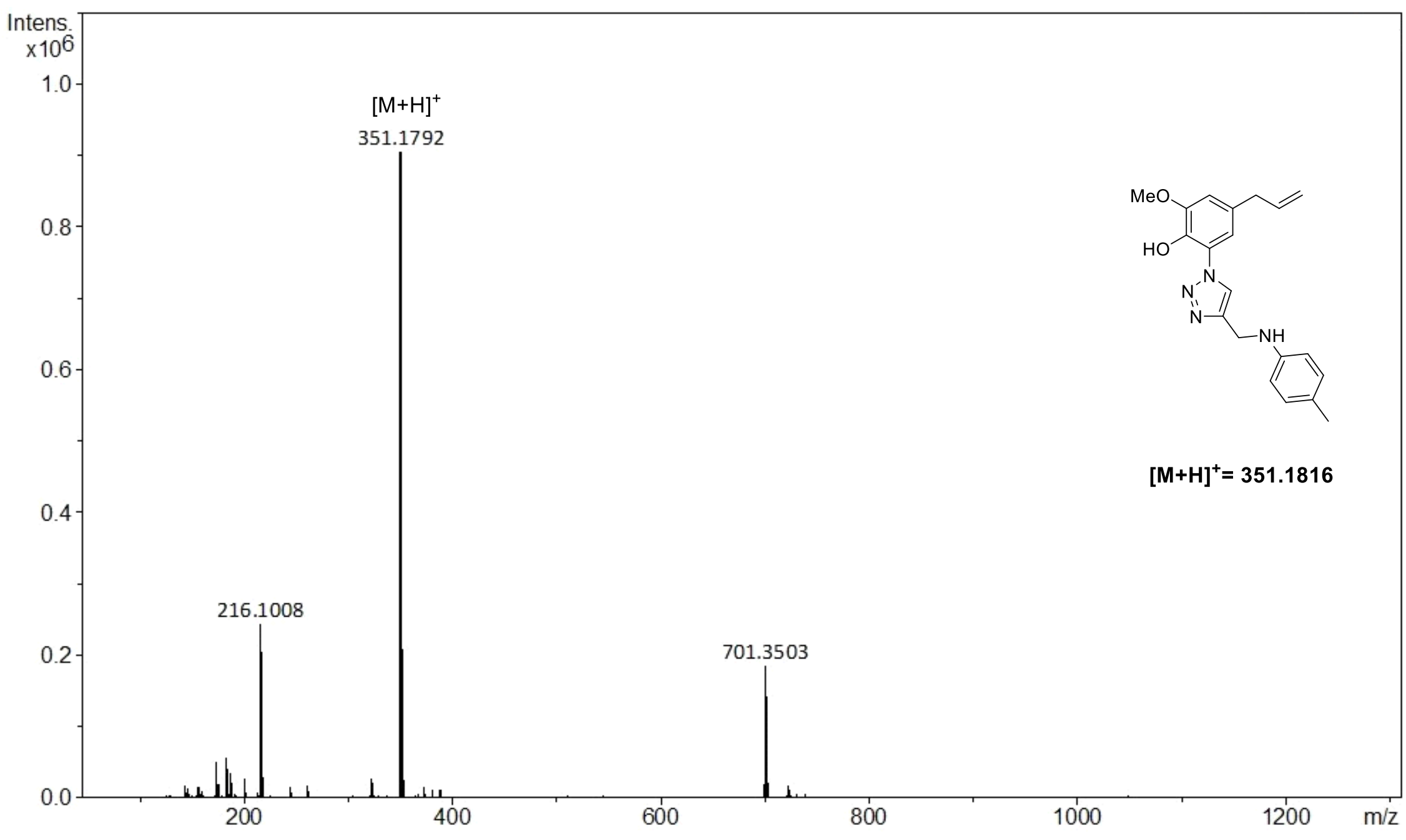

Figure S107. Ionization Spectrum of **4m** (HRMS ESI-TOF).

Ionization Spectrum of 4n (HRMS ESI-TOF)

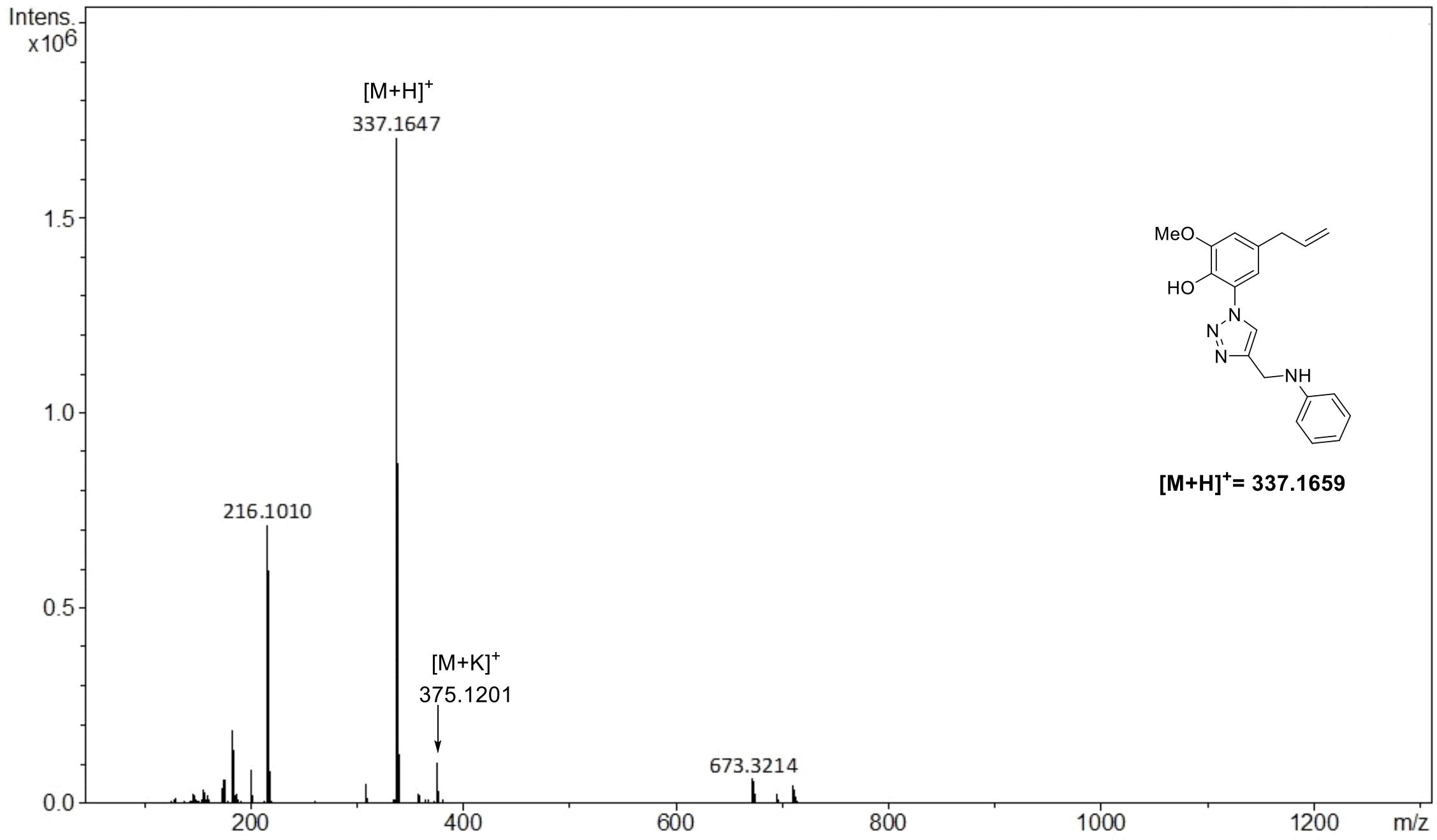

Figure S108. Ionization Spectrum of **4n** (HRMS ESI-TOF).

Ionization Spectrum of 4o (HRMS ESI-TOF)

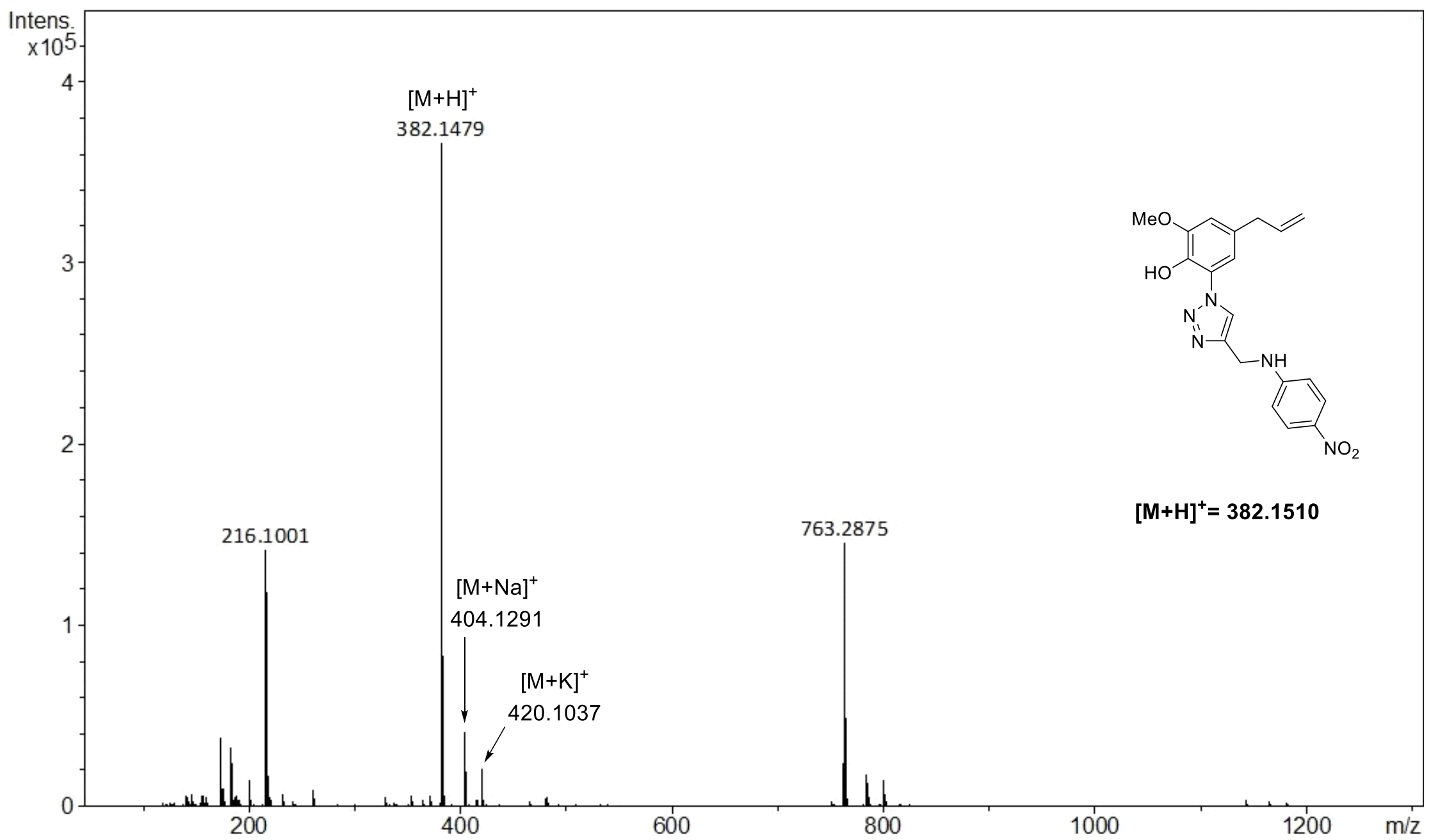

Figure S109. Ionization Spectrum of **4o** (HRMS ESI-TOF).

Ionization Spectrum of 4p (HRMS ESI-TOF)

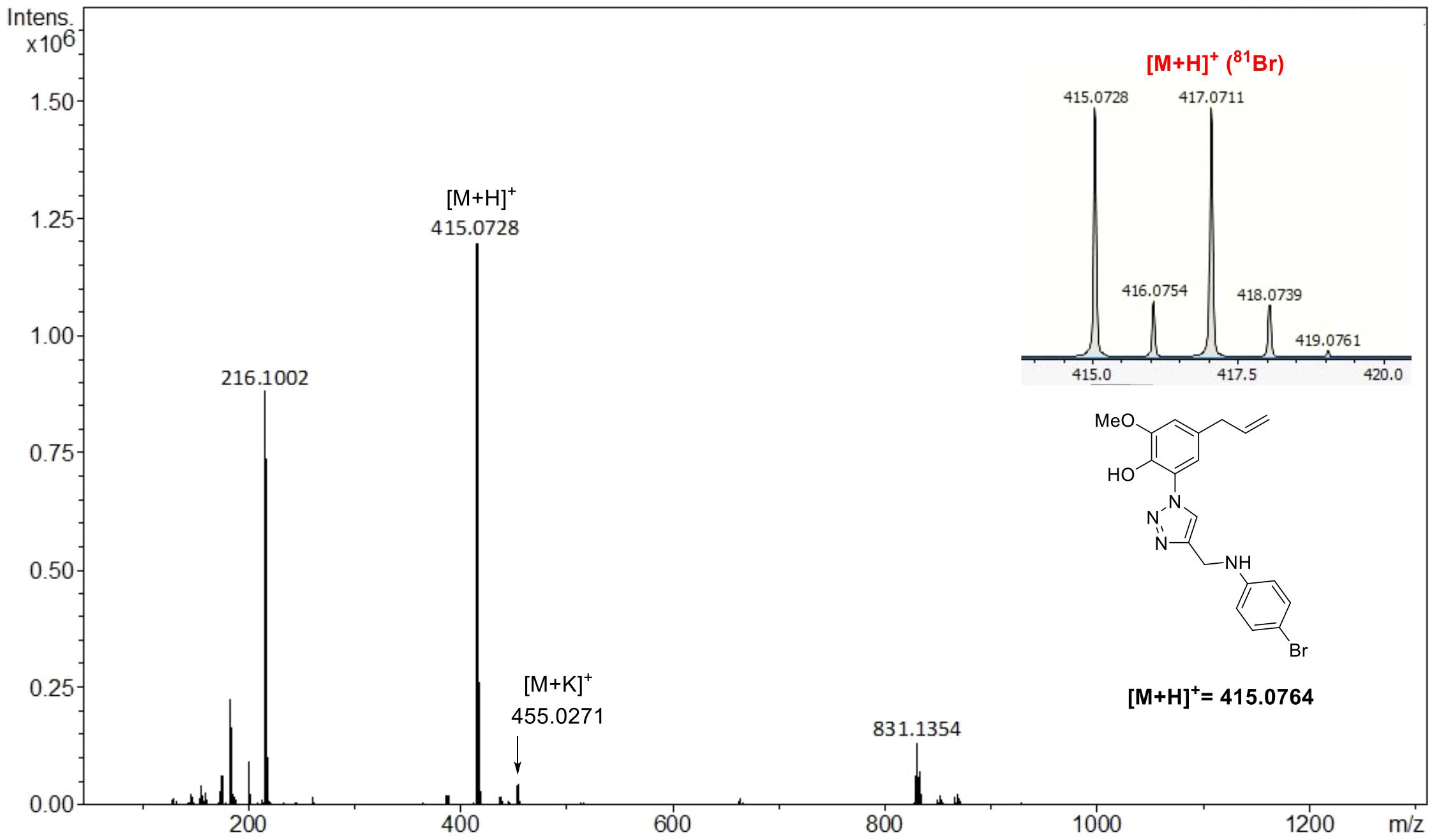

Figure S110. Ionization Spectrum of **4p** (HRMS ESI-TOF).

Ionization Spectrum of 4q (HRMS ESI-TOF)

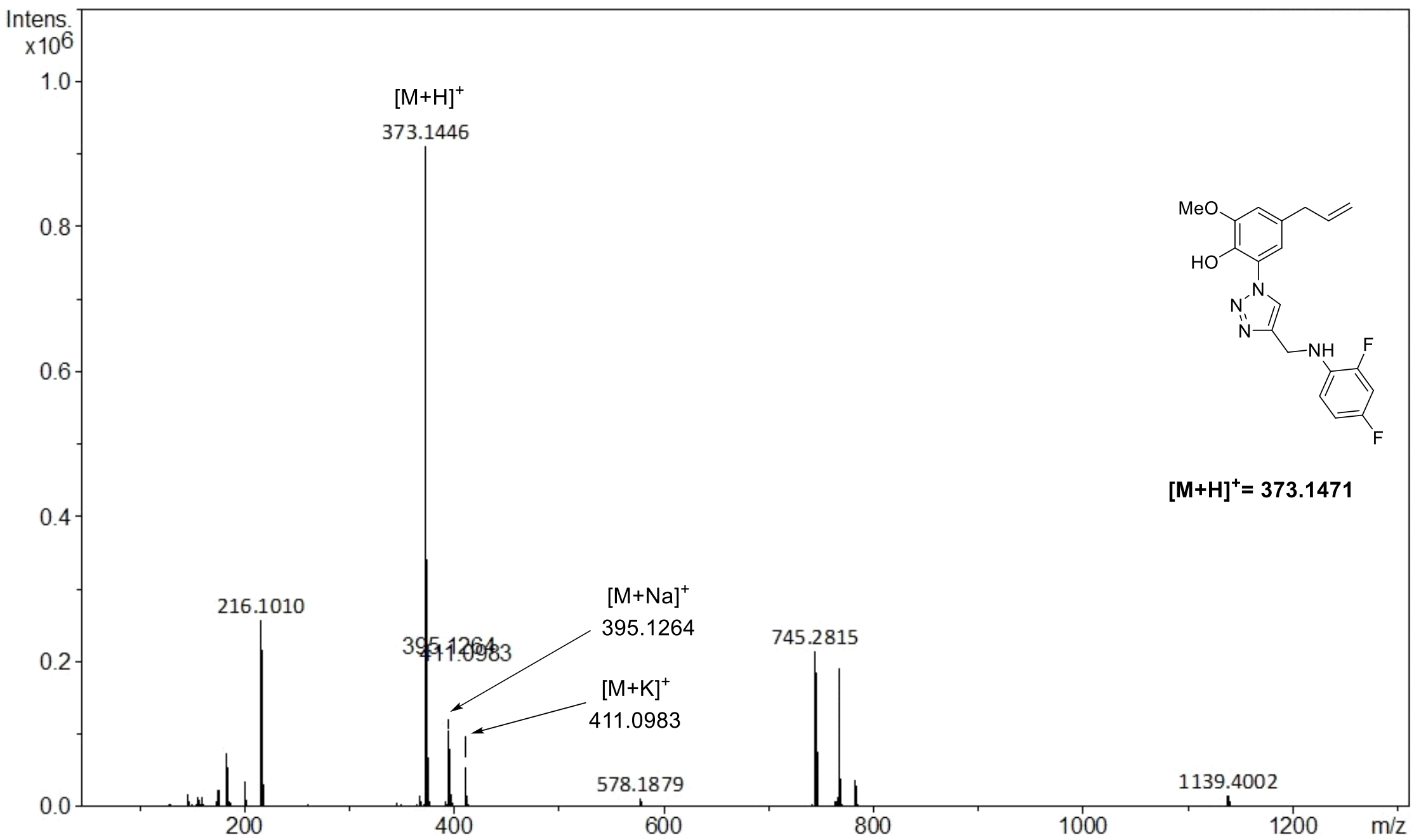

Figure S111. Ionization Spectrum of **4q** (HRMS ESI-TOF).

Ionization Spectrum of 4r (HRMS ESI-TOF)

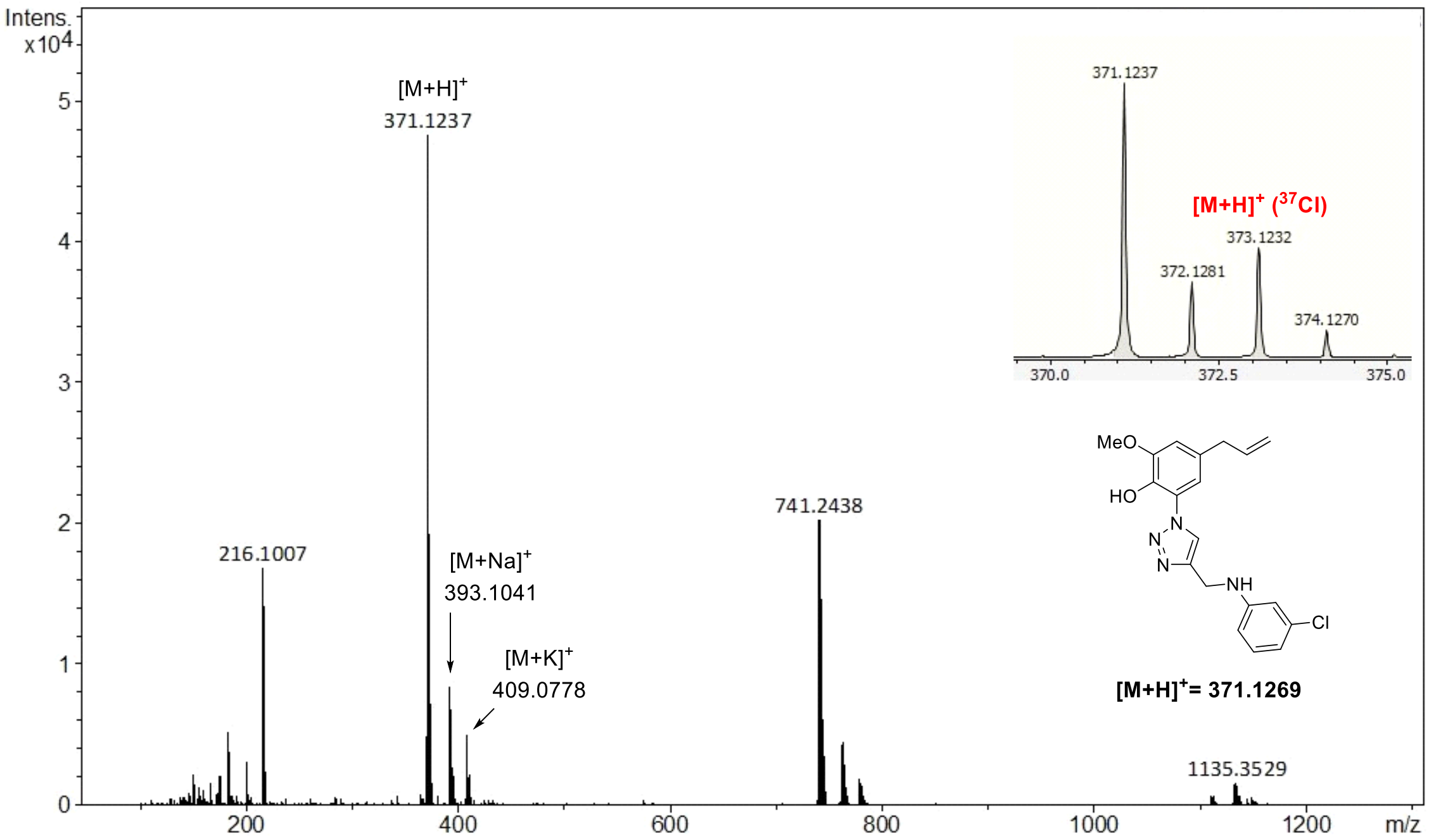

Figure S112. Ionization Spectrum of **4r** (HRMS ESI-TOF).

Ionization Spectrum of 4s (HRMS ESI-TOF)

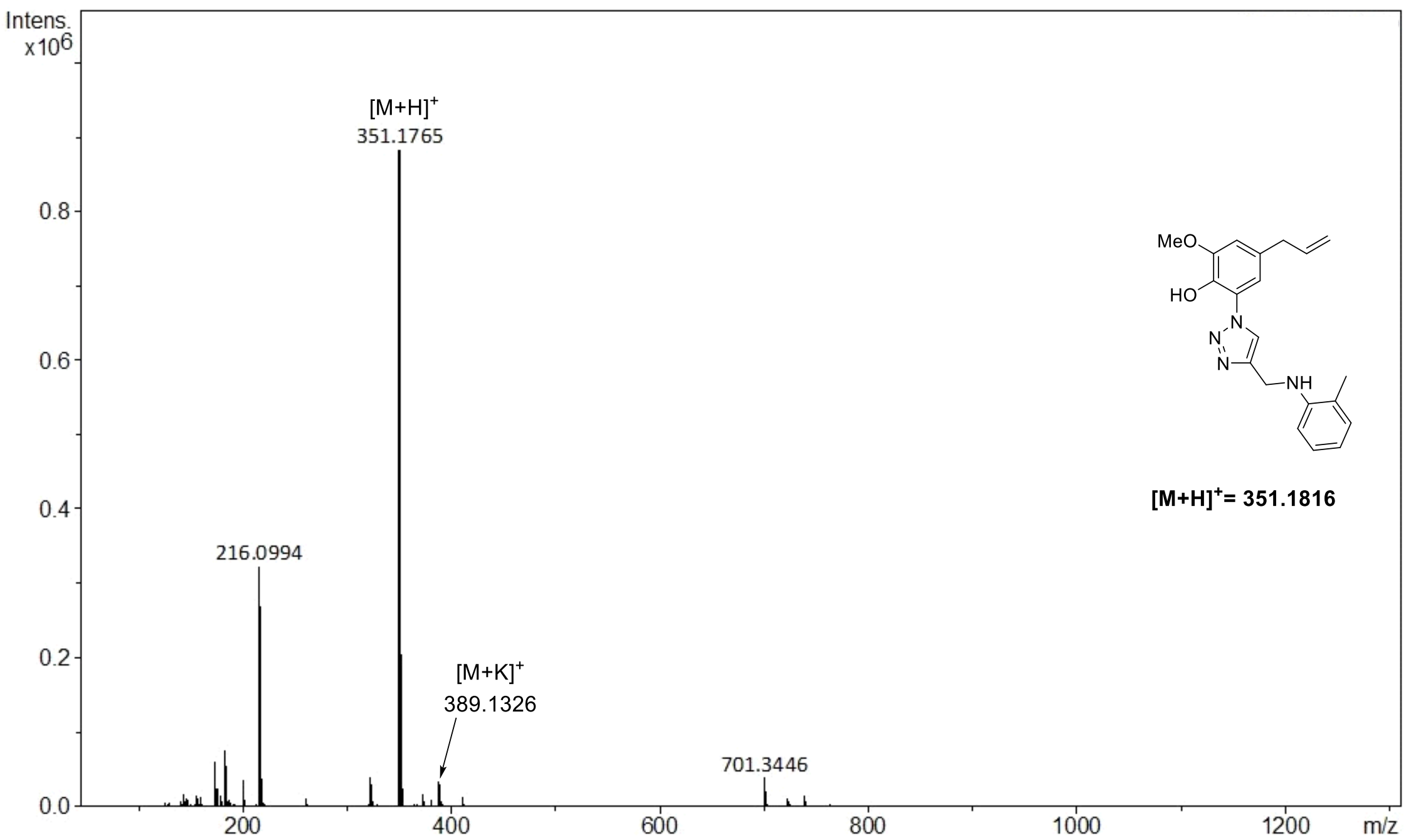

Figure S113. Ionization Spectrum of **4s** (HRMS ESI-TOF).

Ionization Spectrum of 4t (HRMS ESI-TOF)

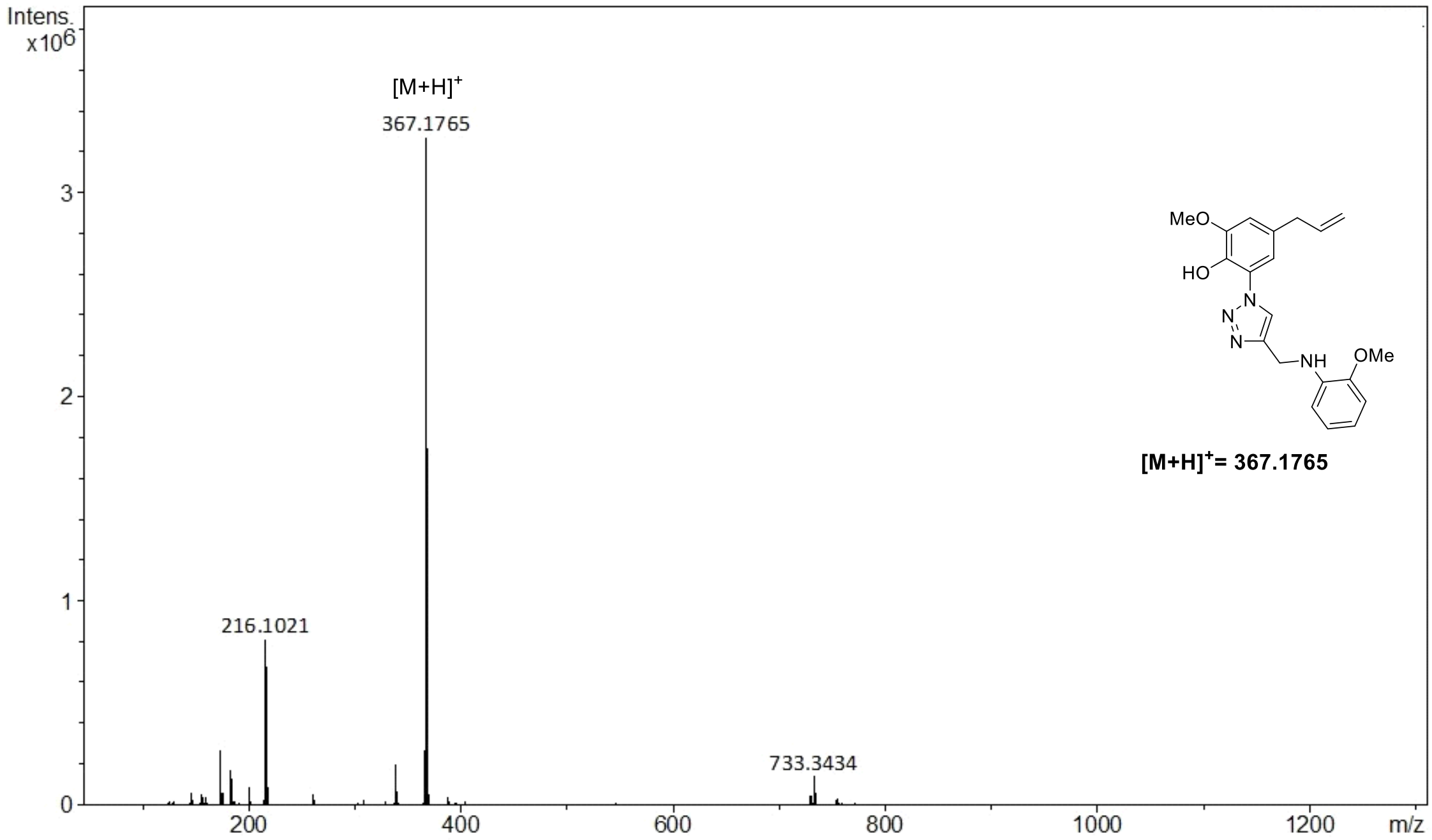

Figure S114. Ionization Spectrum of **4t** (HRMS ESI-TOF).

Ionization Spectrum of 5 (HRMS ESI-TOF)

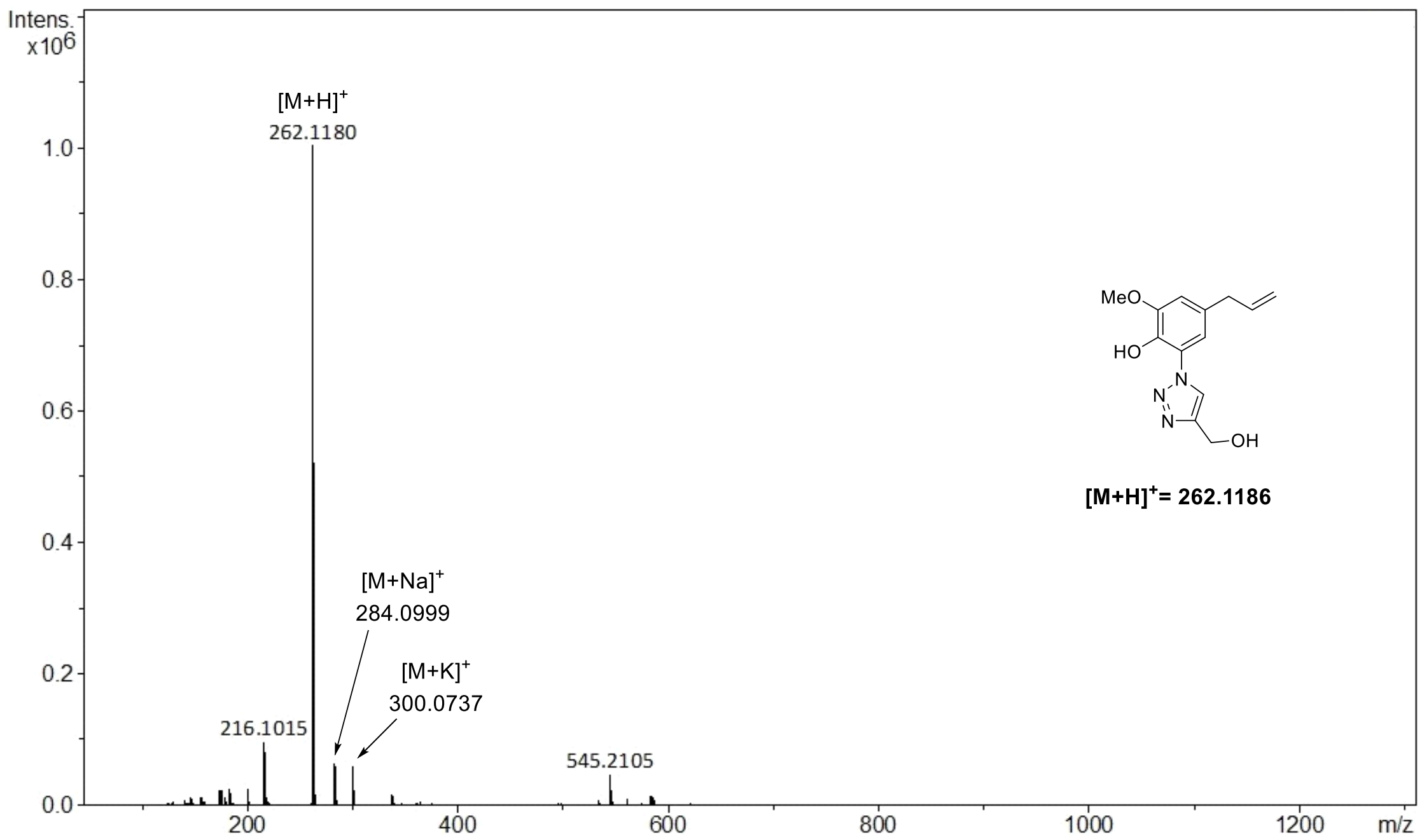

Figure S115. Ionization Spectrum of **5** (HRMS ESI-TOF).

APPENDIX F: High-pressure Liquid Chromatography:

The equipment used for obtaining purity values was an Shimadzu HPLC system composed of a SPD-M10A Multi-wavelength Detector (UV detector), SIL-10AF Autosampler, SCL-10AV Controller, and LC-6AD Binary Pump. Data acquisition was performed using Class VP software. The analyses were carried out with a Phenomenex Luna Phenyl-hexyl column (5 µm, 100 Å, 250 × 4.6 mm) at room temperature, with a flow rate of 1.0 mL/min and an injection volume of 50.0 µL. The Method used mobile phase consisted of solvent A (0.1% formic acid in H_2O) and solvent B (MeOH), following the gradient program: 50% A and 50% B at 0.0 min, 0% A and 100% B at 15.0 min, maintaining 100% B at 15.00–20.0 min and returning to 50% B at 25-29 min Detection was performed at a UV wavelength of 254 nm.

HPLC Chromatogram of: 0.1% formic acid in H_2O, and MeOH

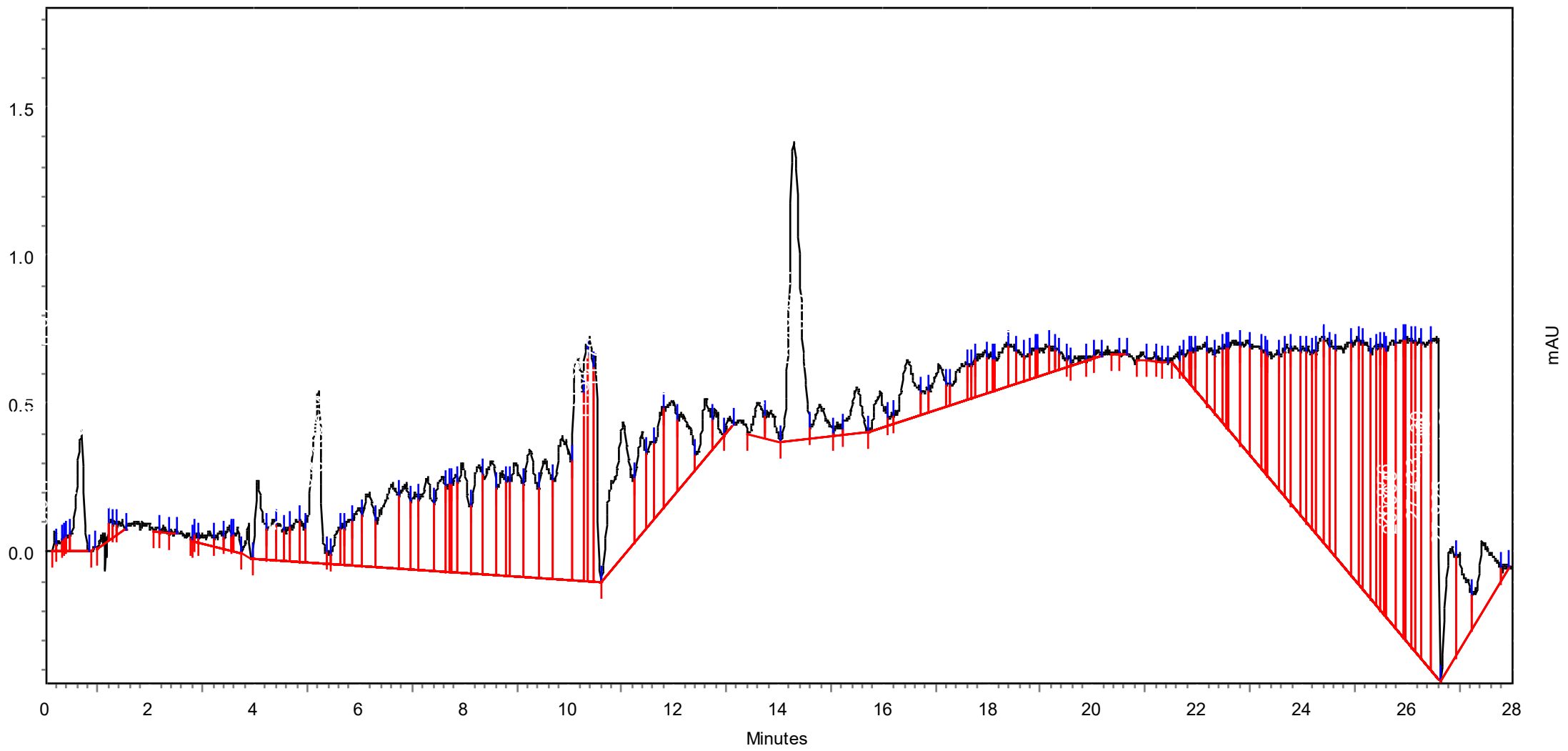

Figure S116. HPLC Chromatogram of: 0.1% formic acid in H_2O, and MeOH.

HPLC Chromatogram of 4a

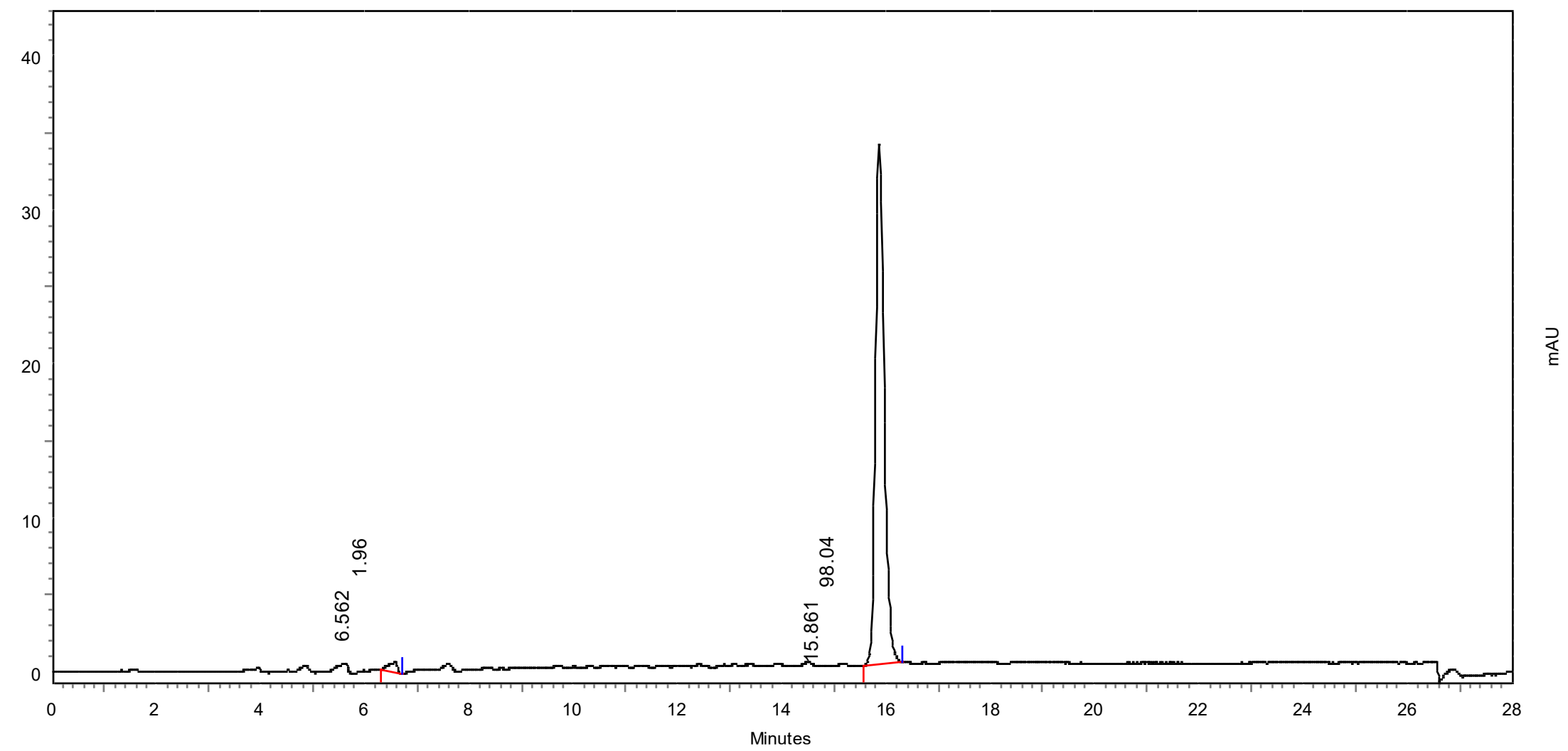

Figure S117. HPLC Chromatogram of **4a**.

HPLC Chromatogram of 4b

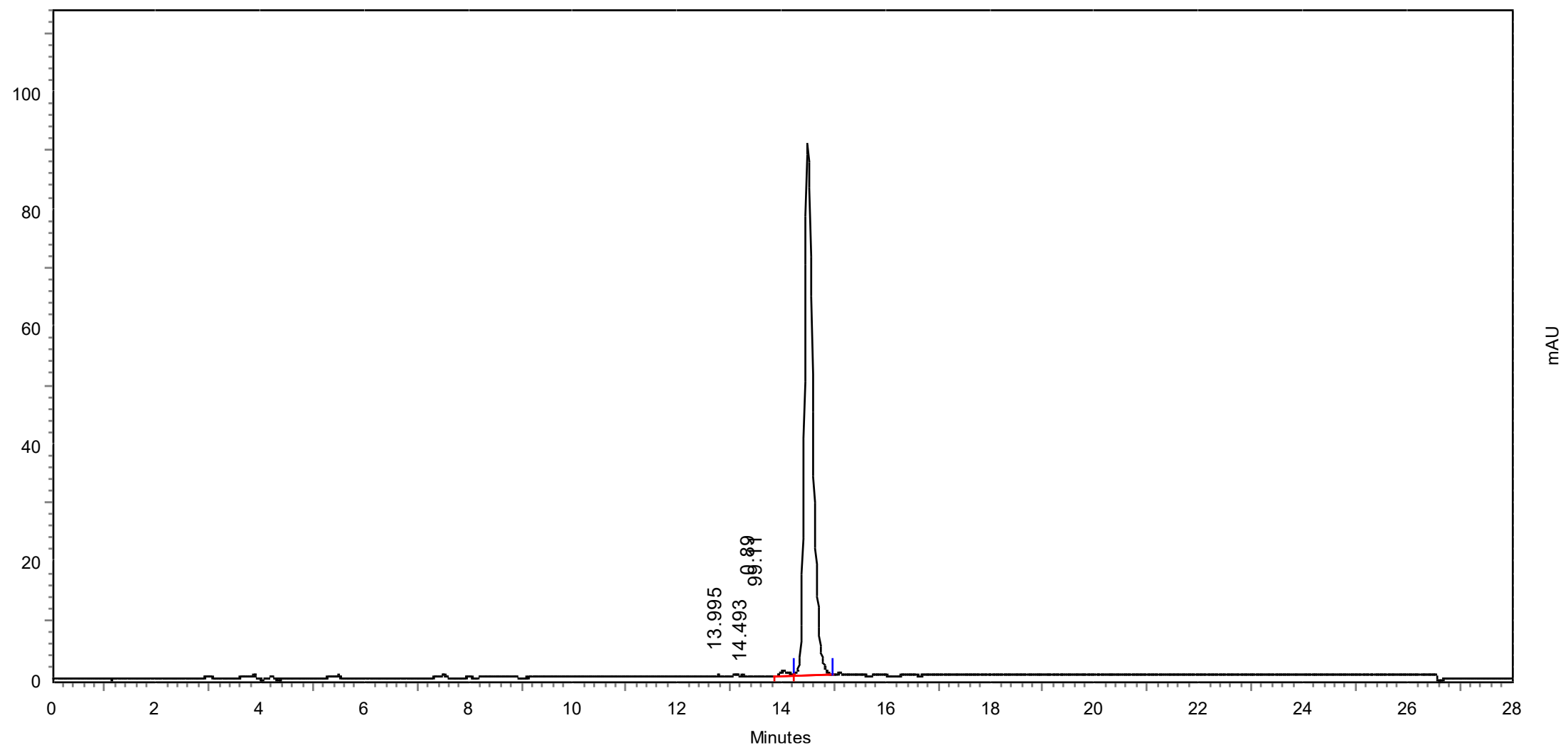

Figure S118. HPLC Chromatogram of **4b**.

HPLC Chromatogram of 4c

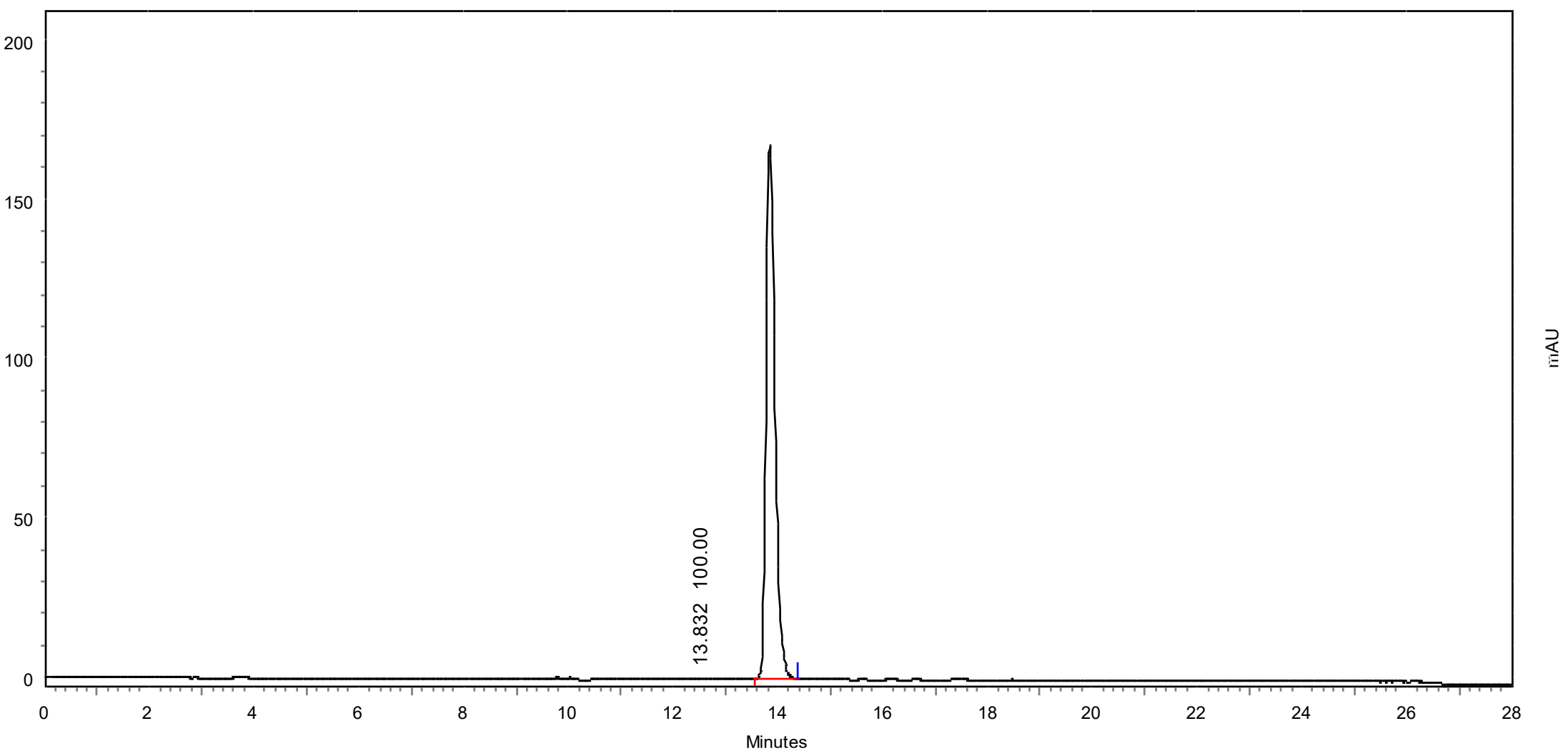

Figure S119. HPLC Chromatogram of **4c**.

HPLC Chromatogram of 4d

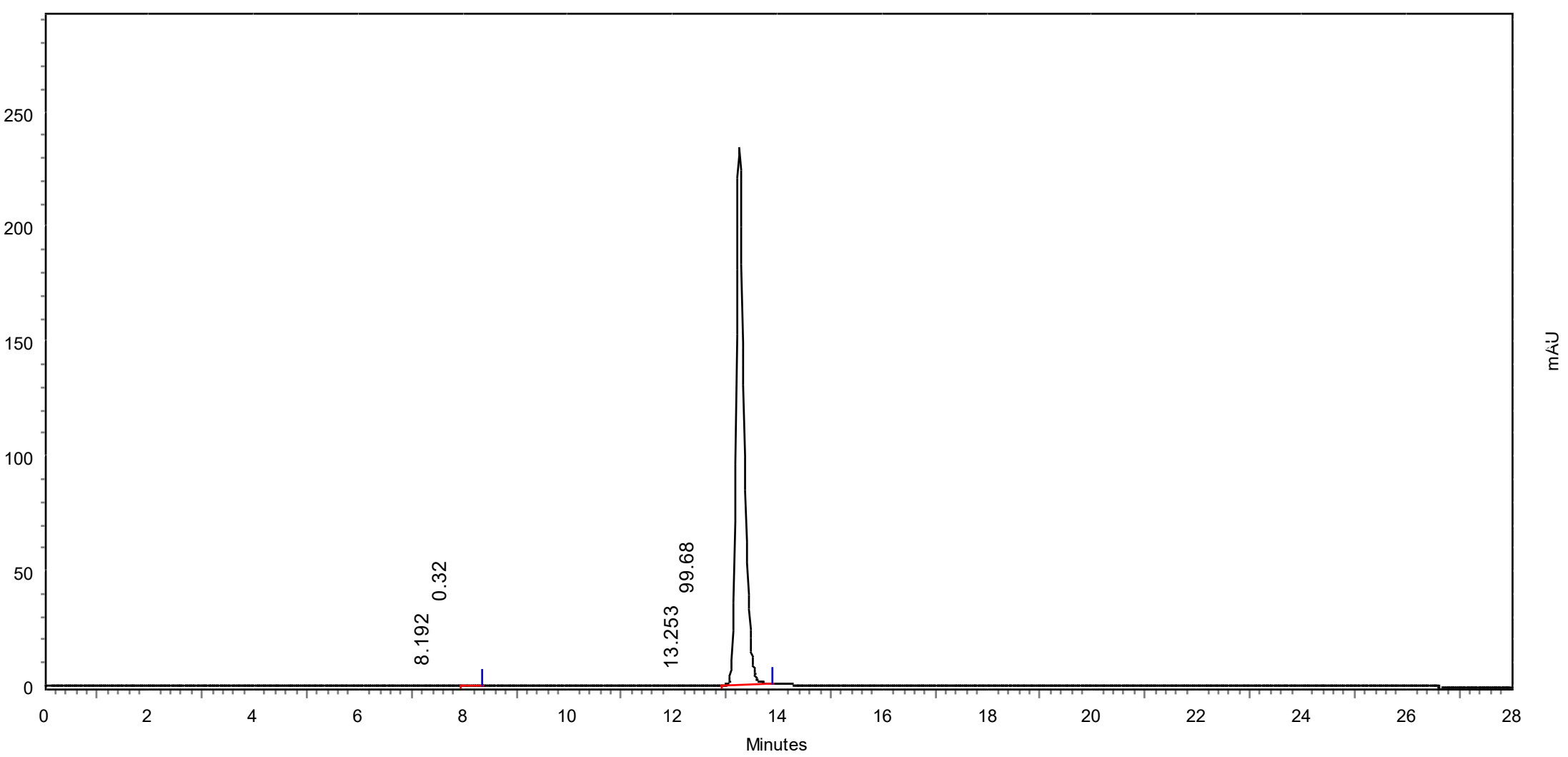

Figure S120. HPLC Chromatogram of **4d**.

HPLC Chromatogram of 4e

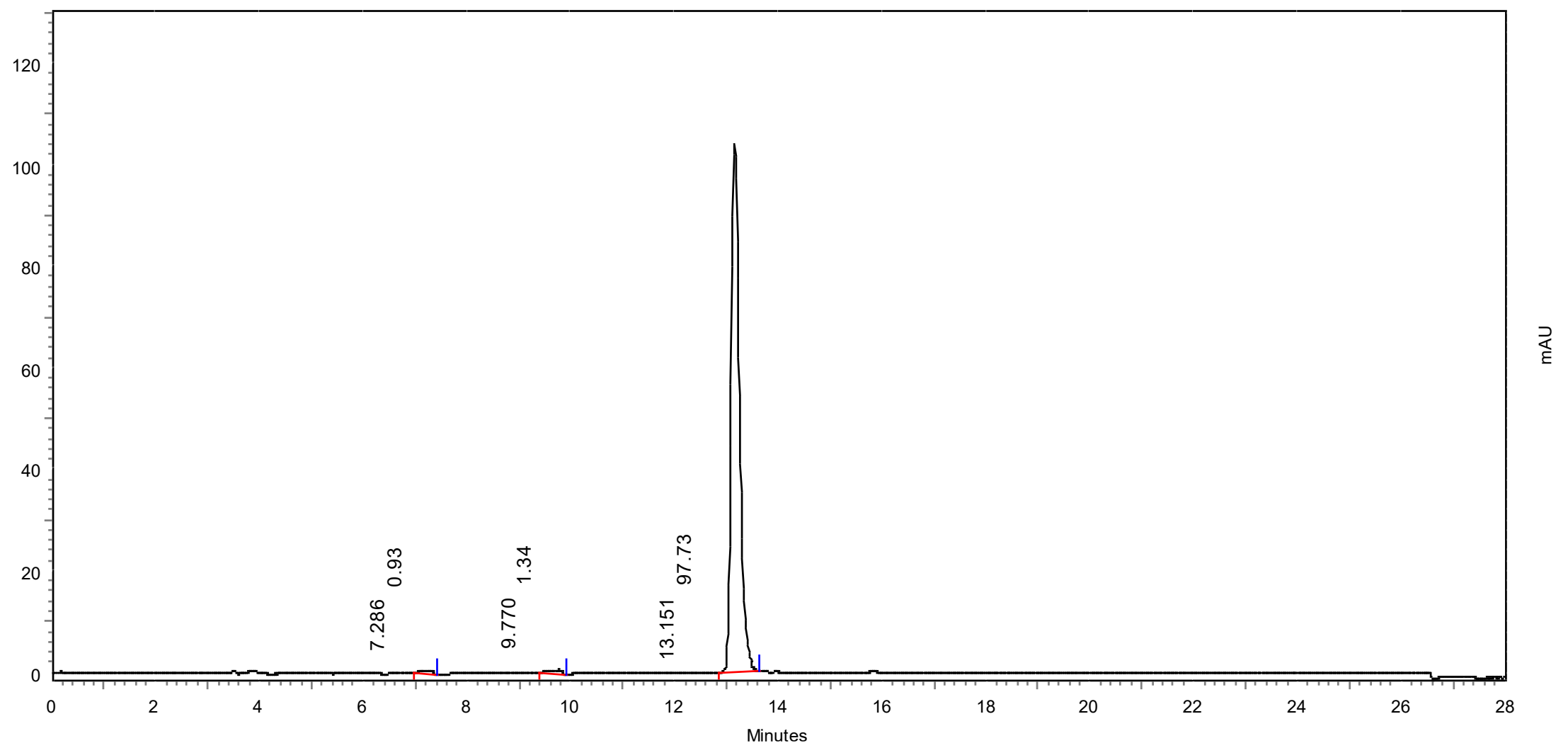

Figure S121. HPLC Chromatogram of **4e**.

HPLC Chromatogram of 4f

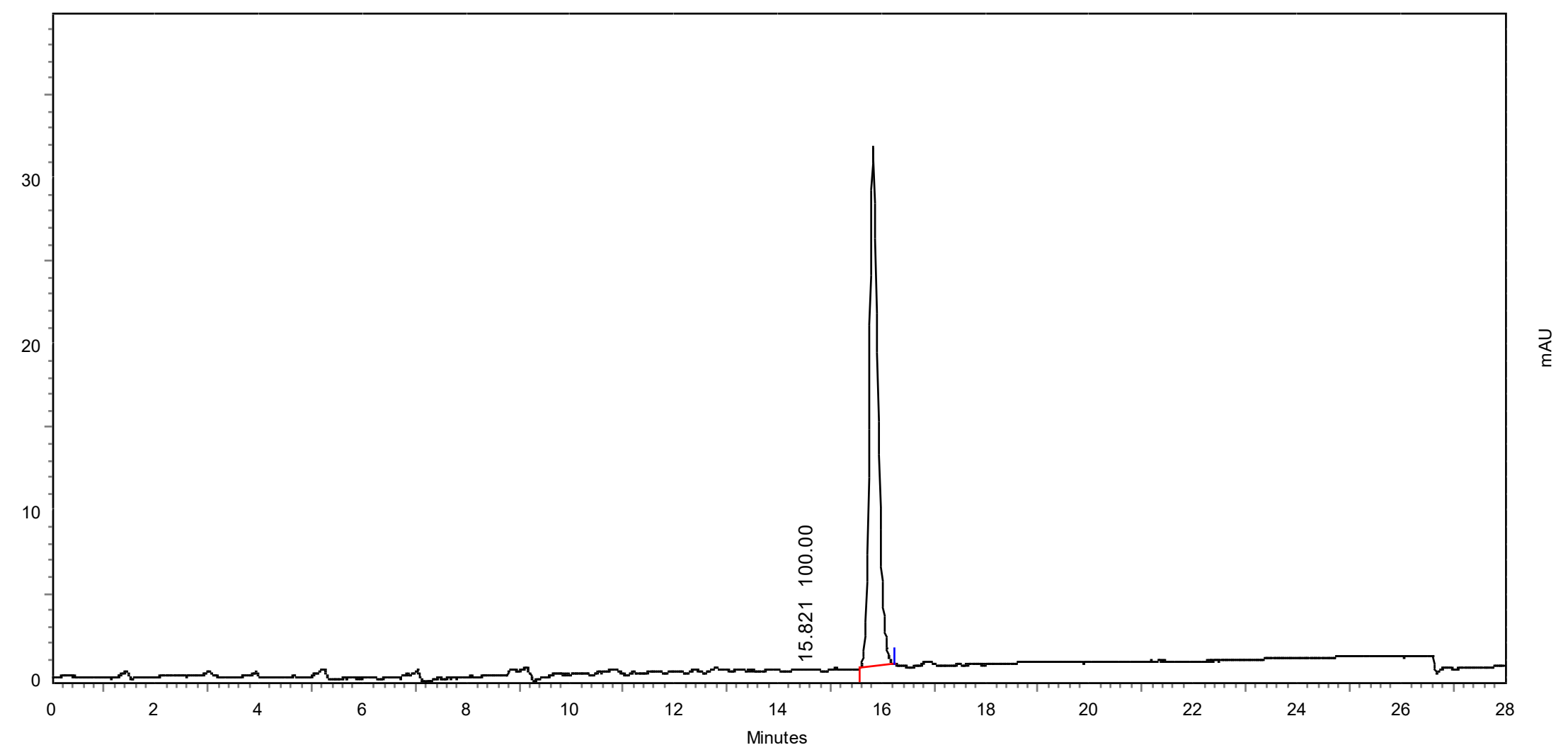

Figure S122. HPLC Chromatogram of **4f**.

HPLC Chromatogram of 4g

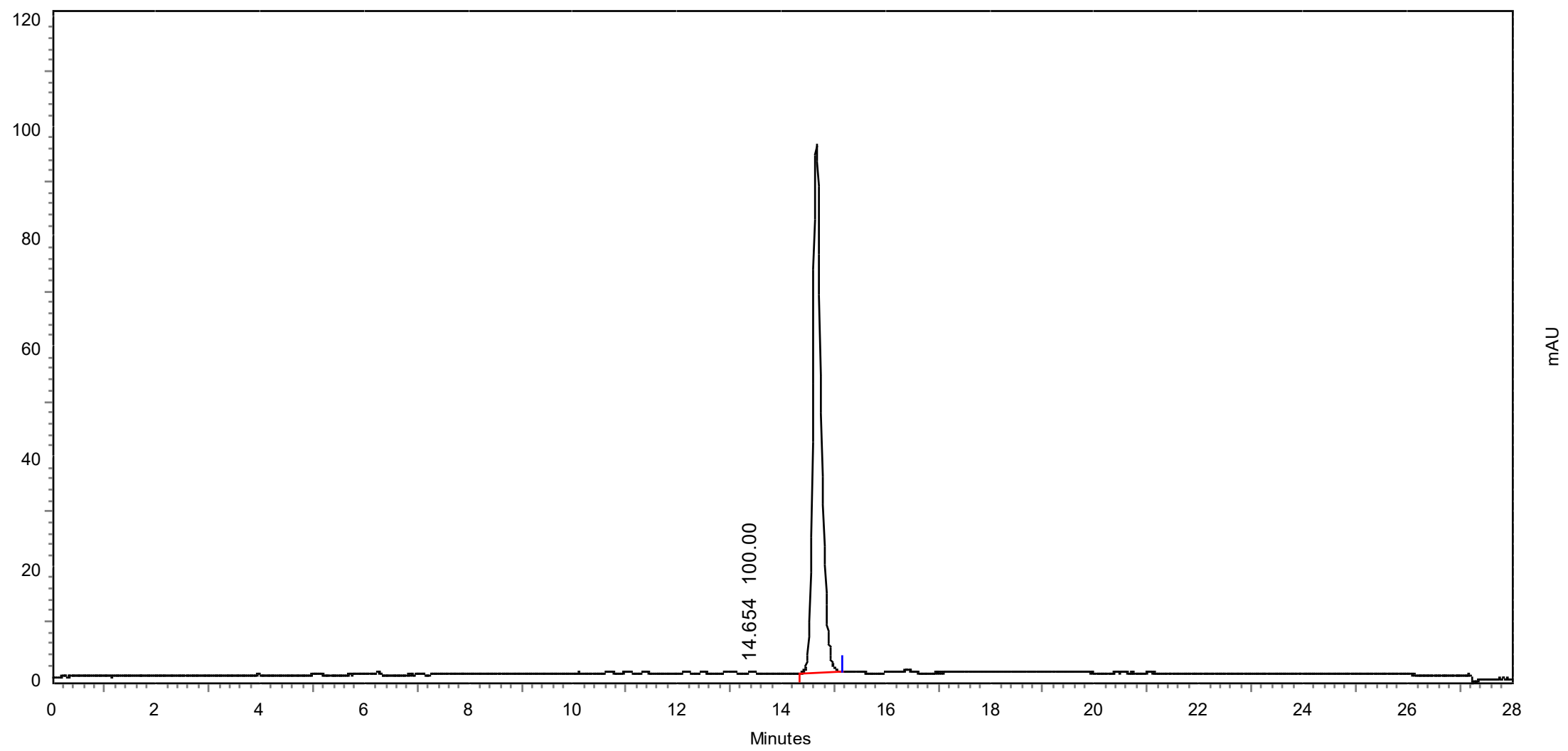

Figure S123. HPLC Chromatogram of **4g**.

HPLC Chromatogram of 4h

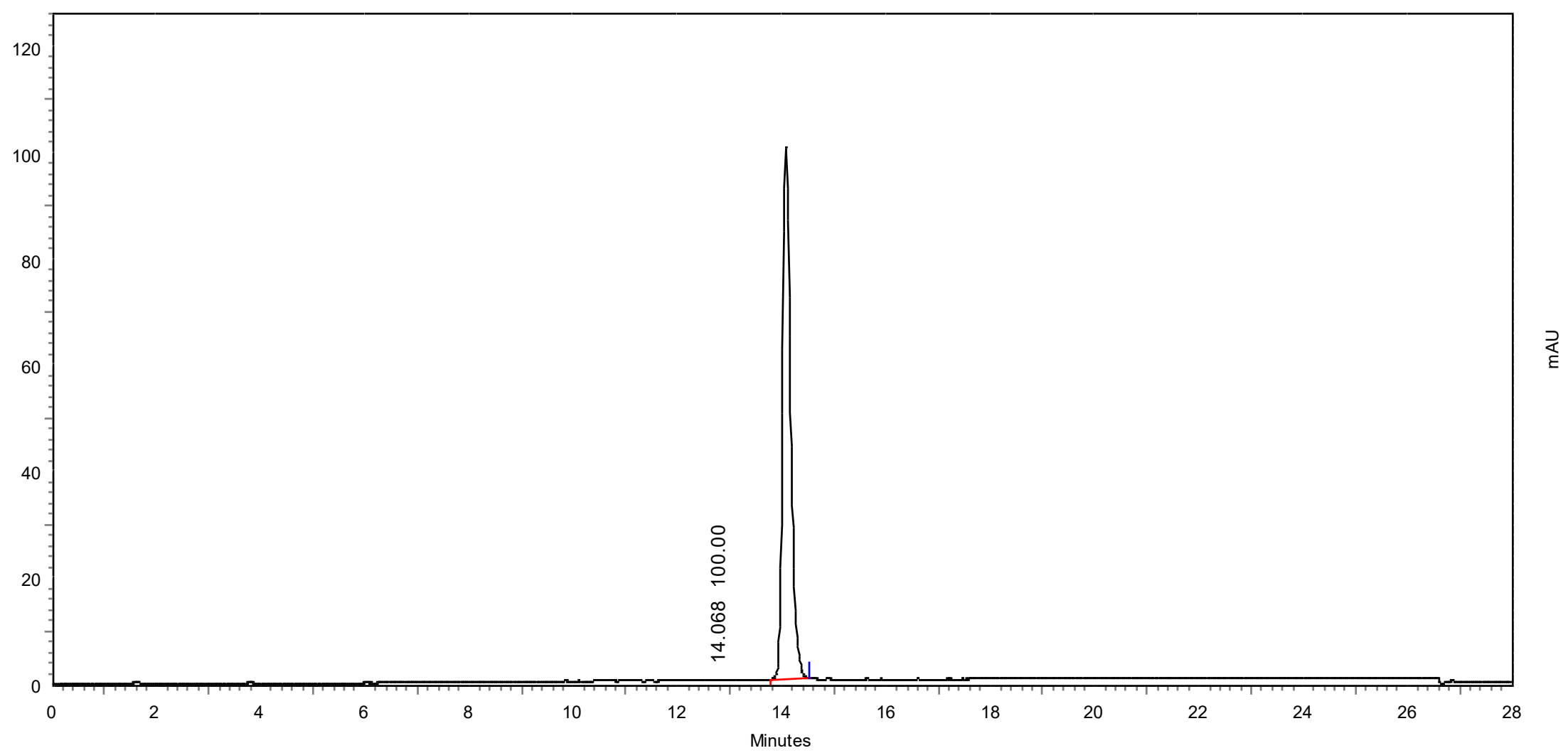

Figure S124. HPLC Chromatogram of **4h**.

HPLC Chromatogram of 4i

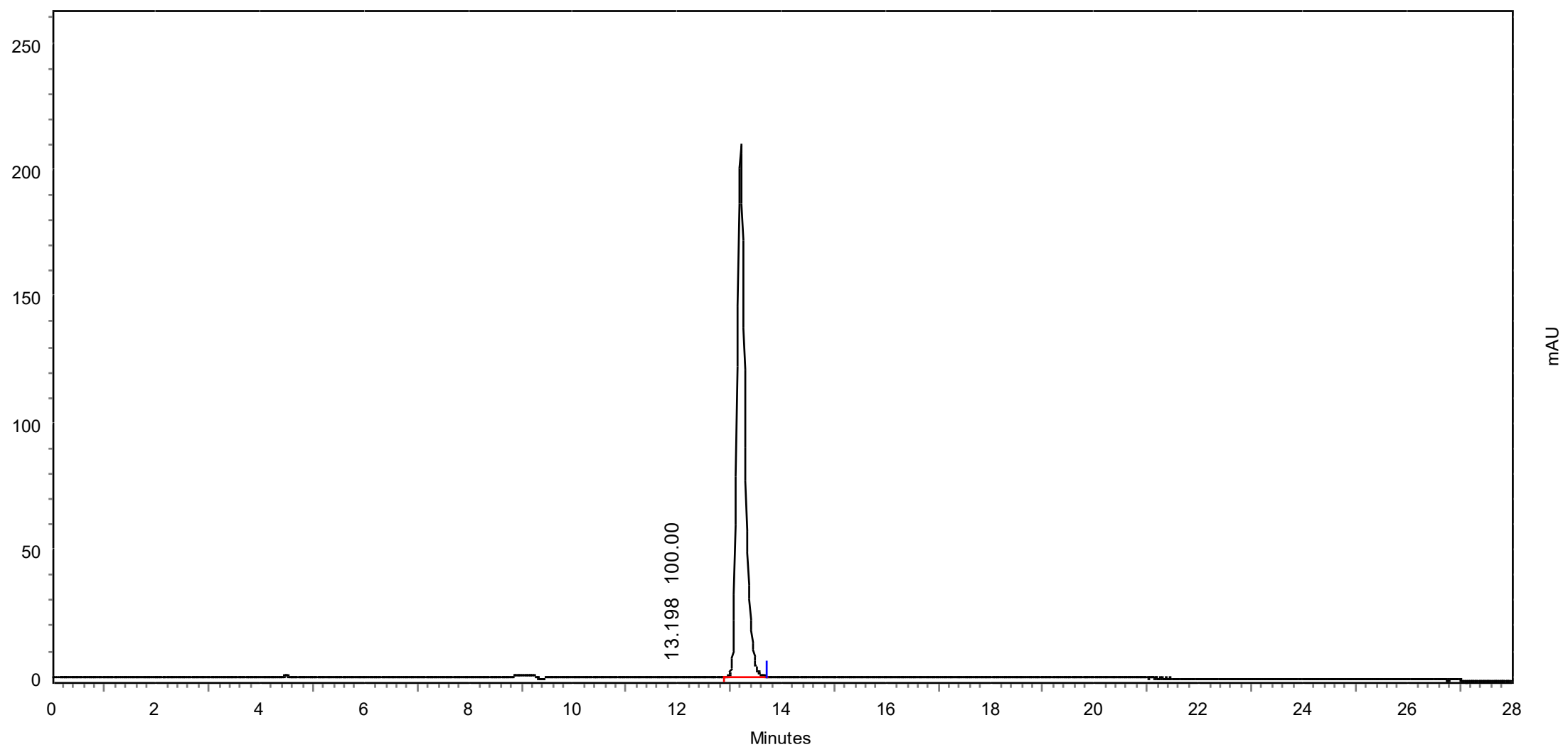

Figure S125. HPLC Chromatogram of **4i**.

HPLC Chromatogram of 4j

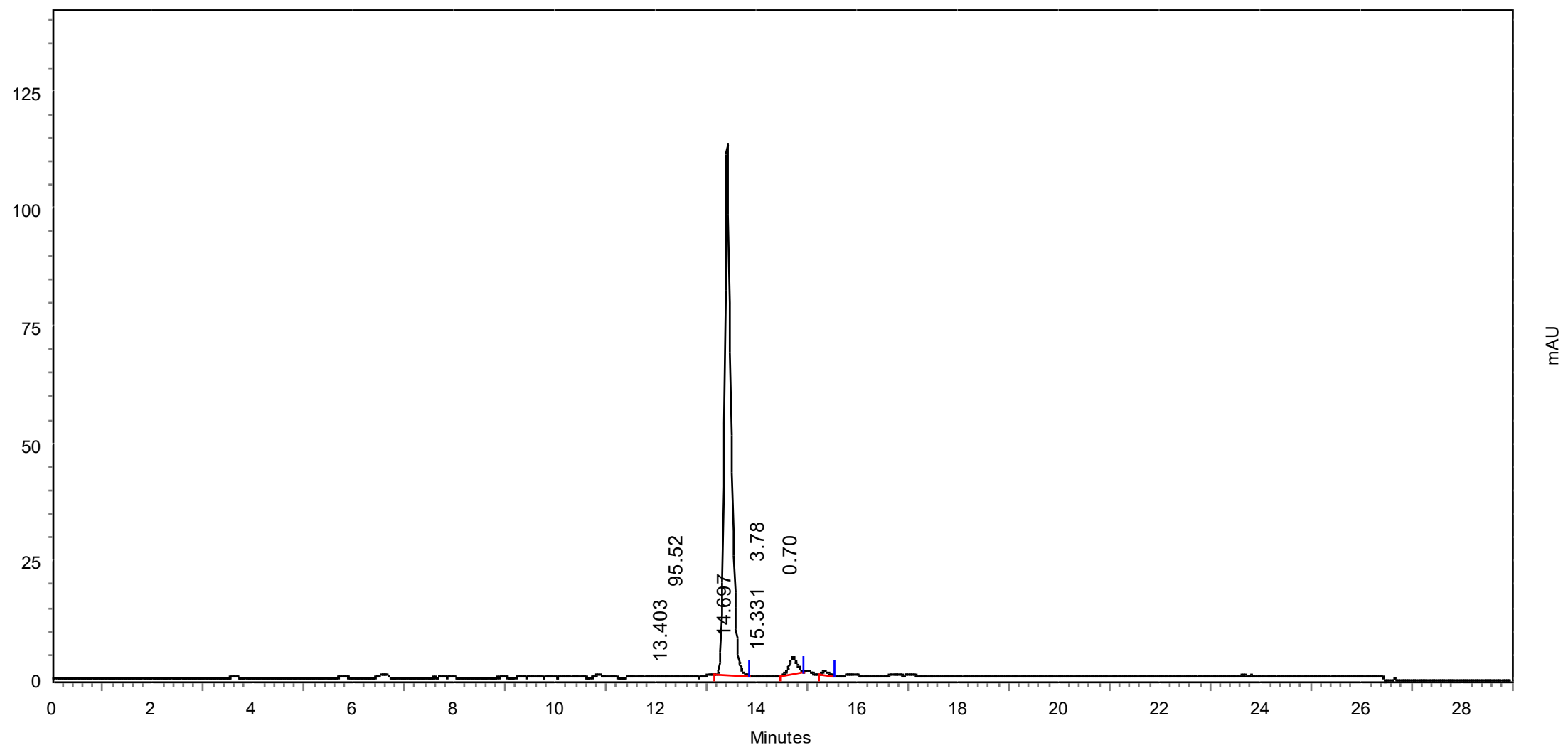

Figure S126. HPLC Chromatogram of **4j**.

HPLC Chromatogram of 4k

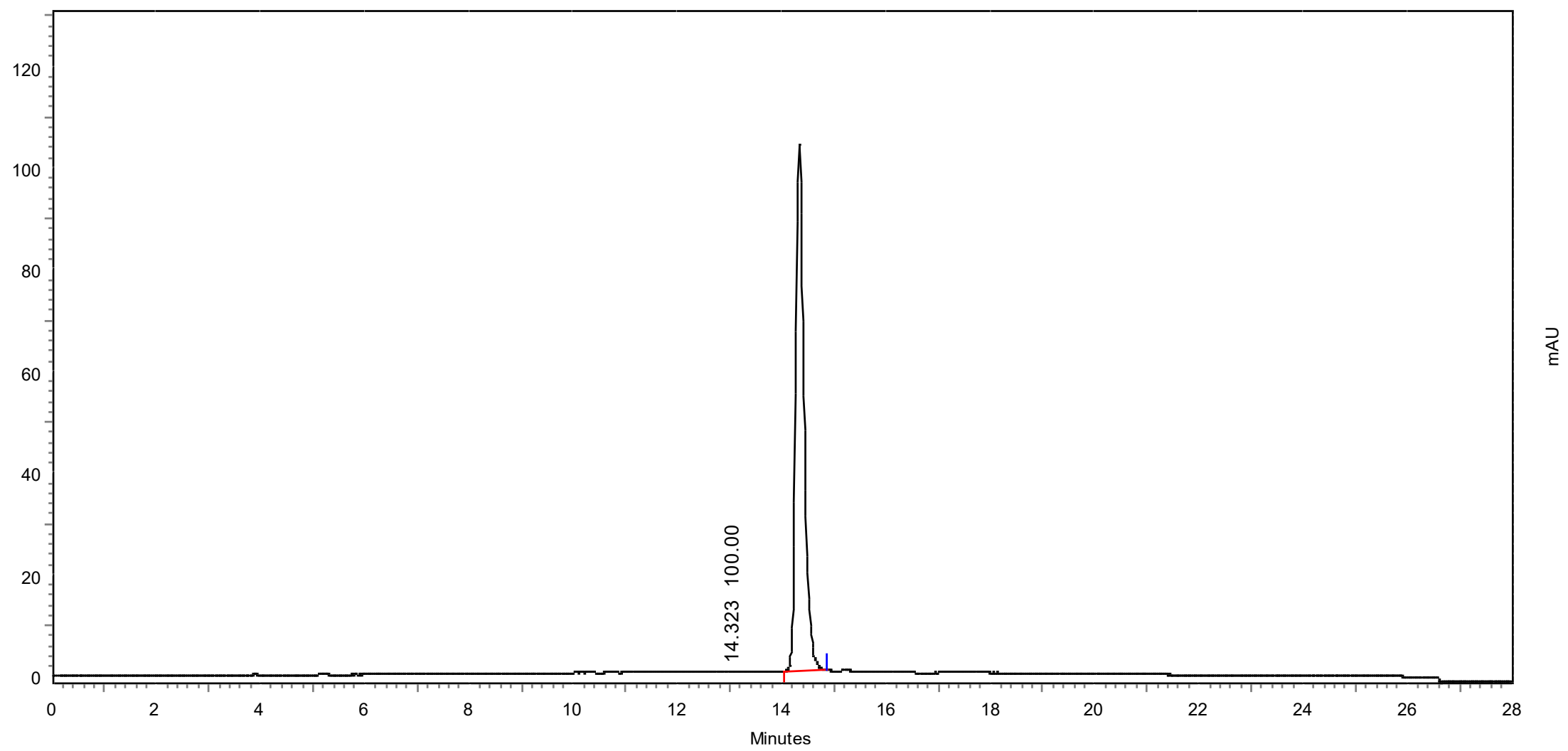

Figure S127. HPLC Chromatogram of **4k**.

HPLC Chromatogram of 4l

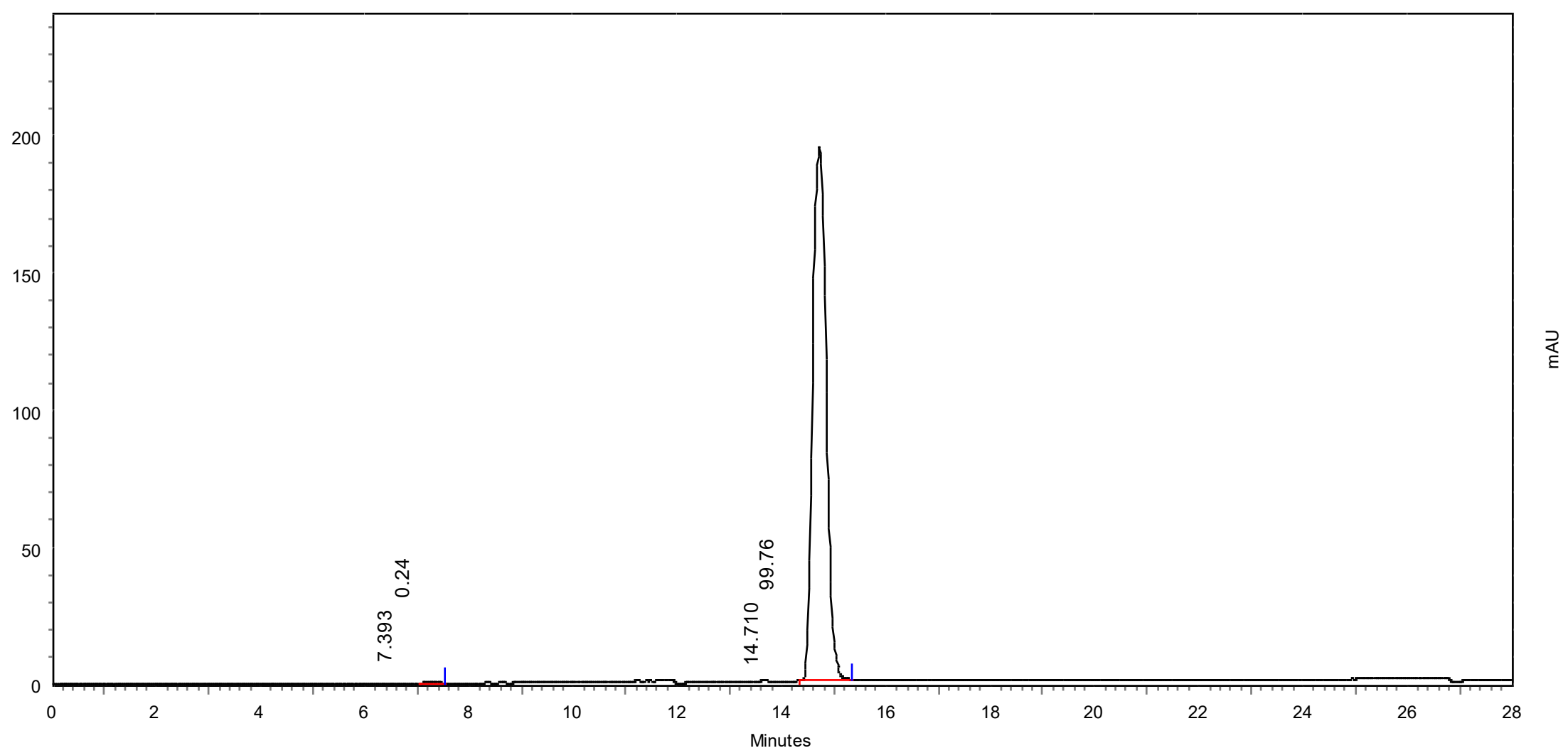

Figure S128. HPLC Chromatogram of **4l**.

HPLC Chromatogram of 4m

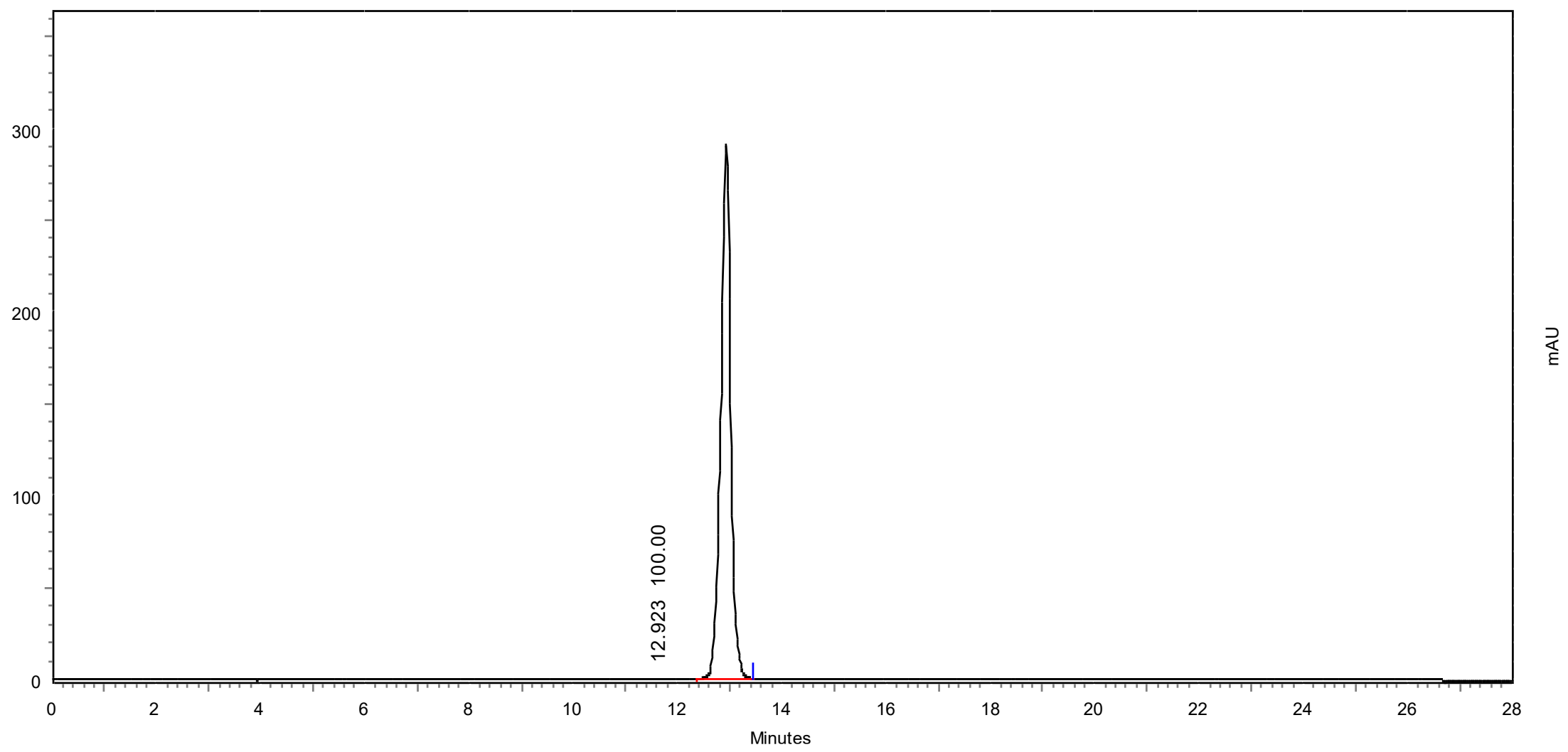

Figure S129. HPLC Chromatogram of **4m**.

HPLC Chromatogram of 4n

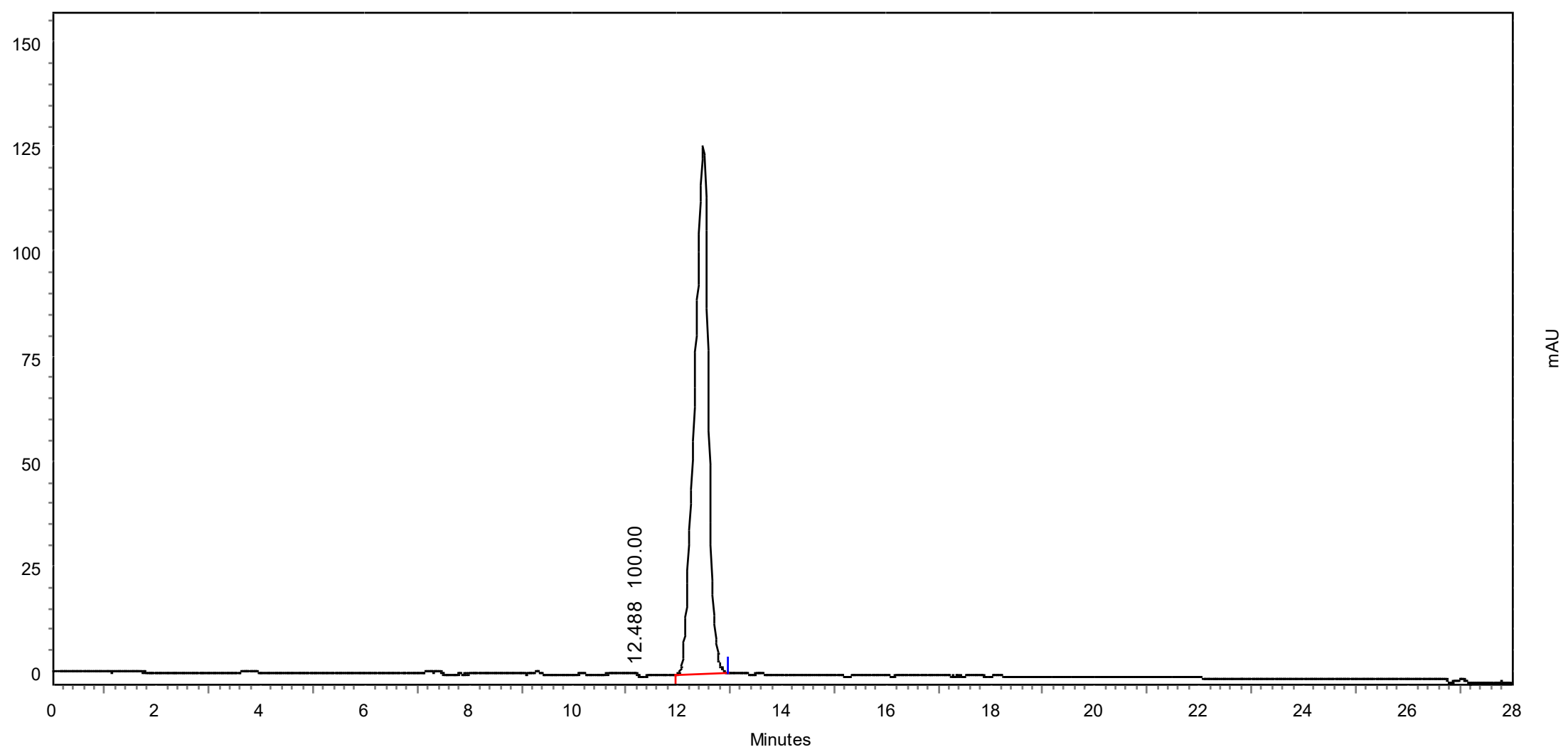

Figure S130. HPLC Chromatogram of **4n**.

HPLC Chromatogram of 4o

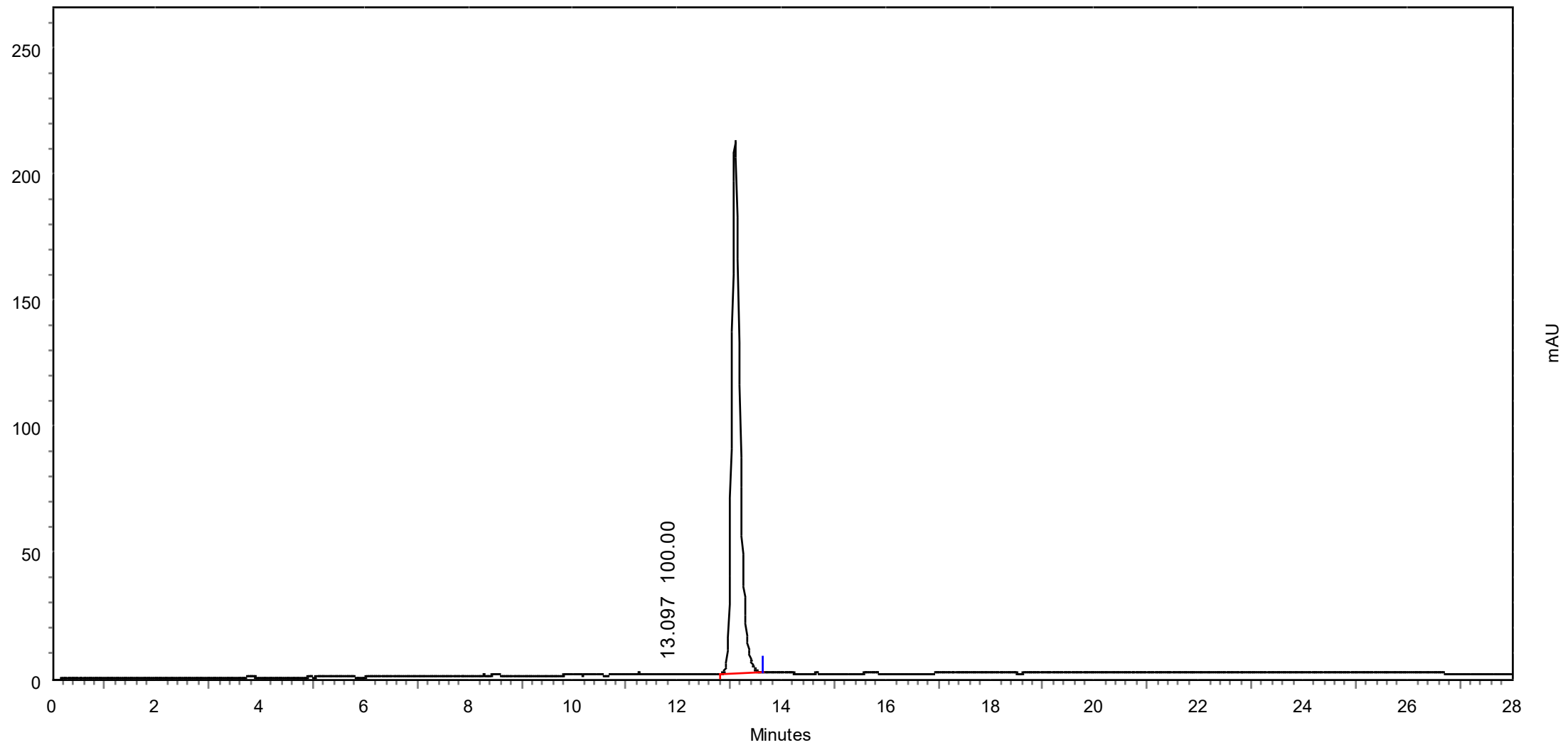

Figure S131. HPLC Chromatogram of **4o**.

HPLC Chromatogram of 4p

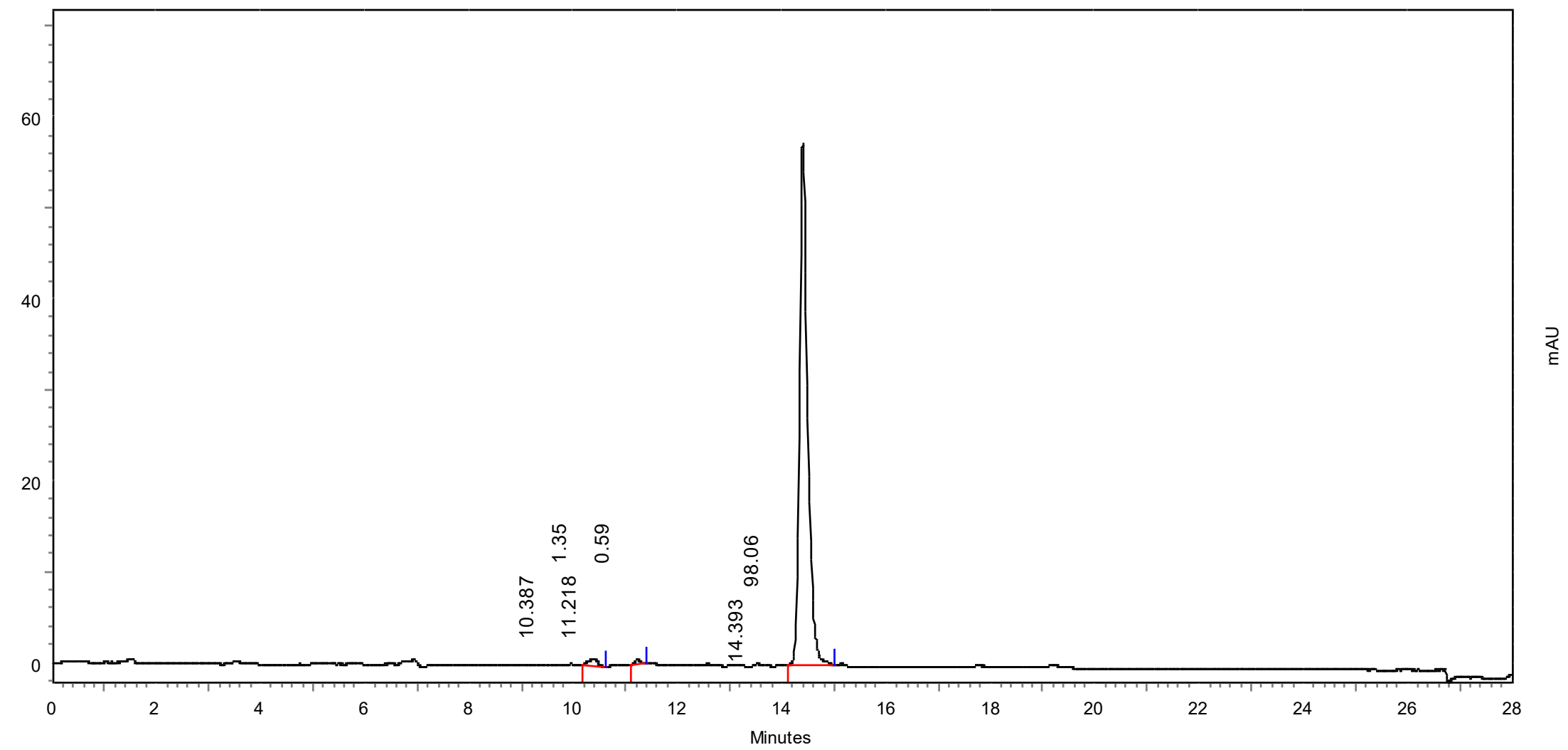

Figure S132. HPLC Chromatogram of **4p**.

HPLC Chromatogram of 4q

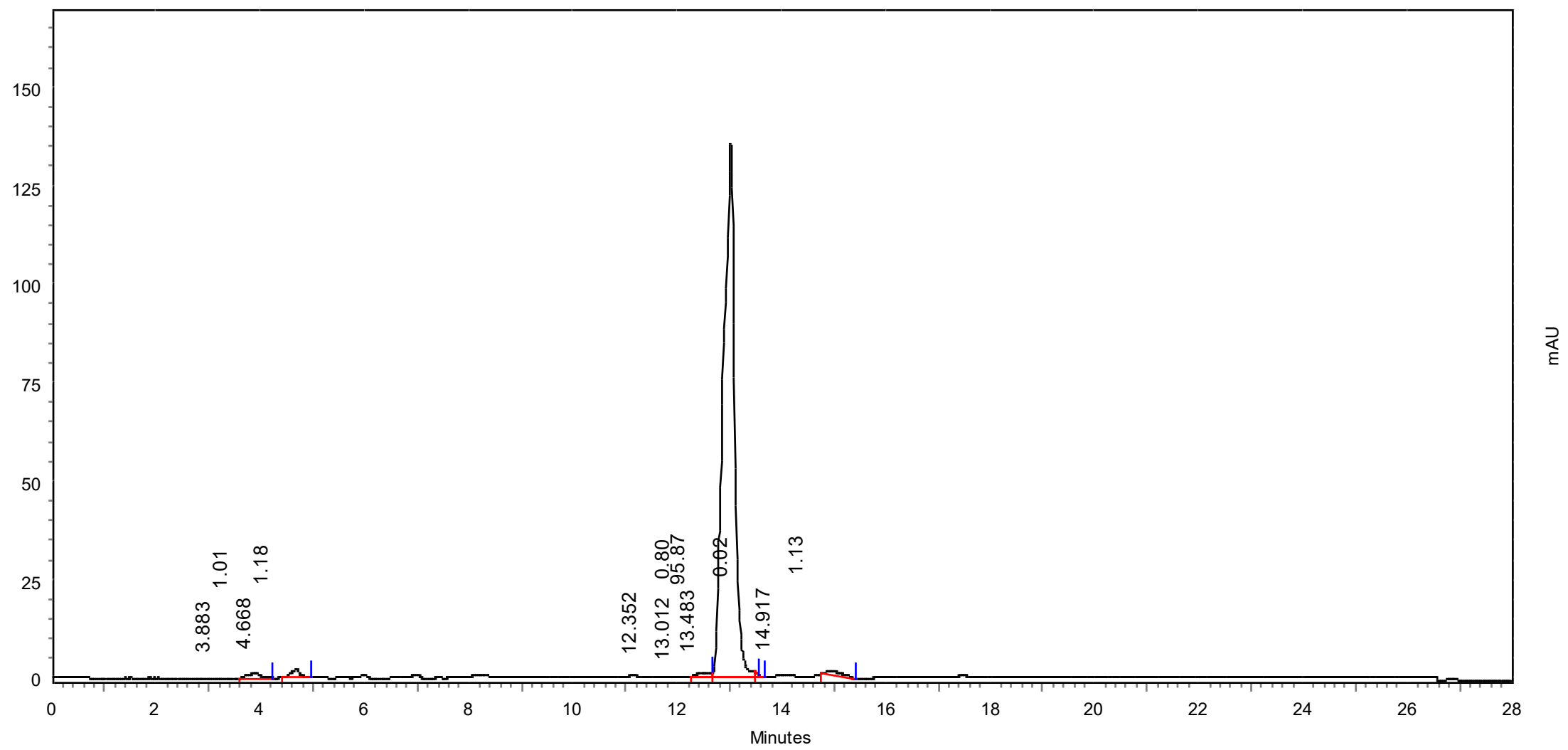

Figure S133. HPLC Chromatogram of **4q**.

HPLC Chromatogram of 4r

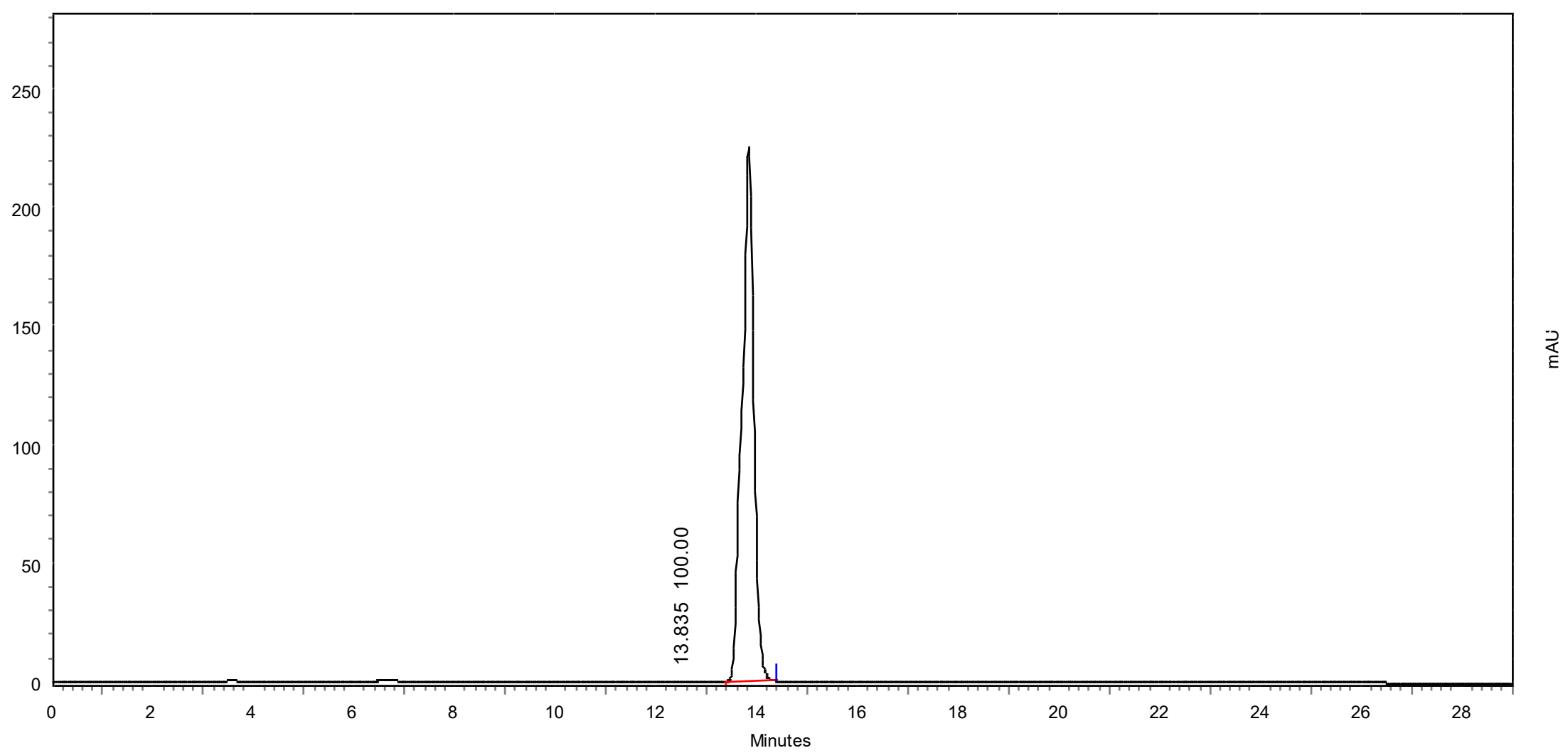

Figure S134. HPLC Chromatogram of **4r**.

HPLC Chromatogram of 4s

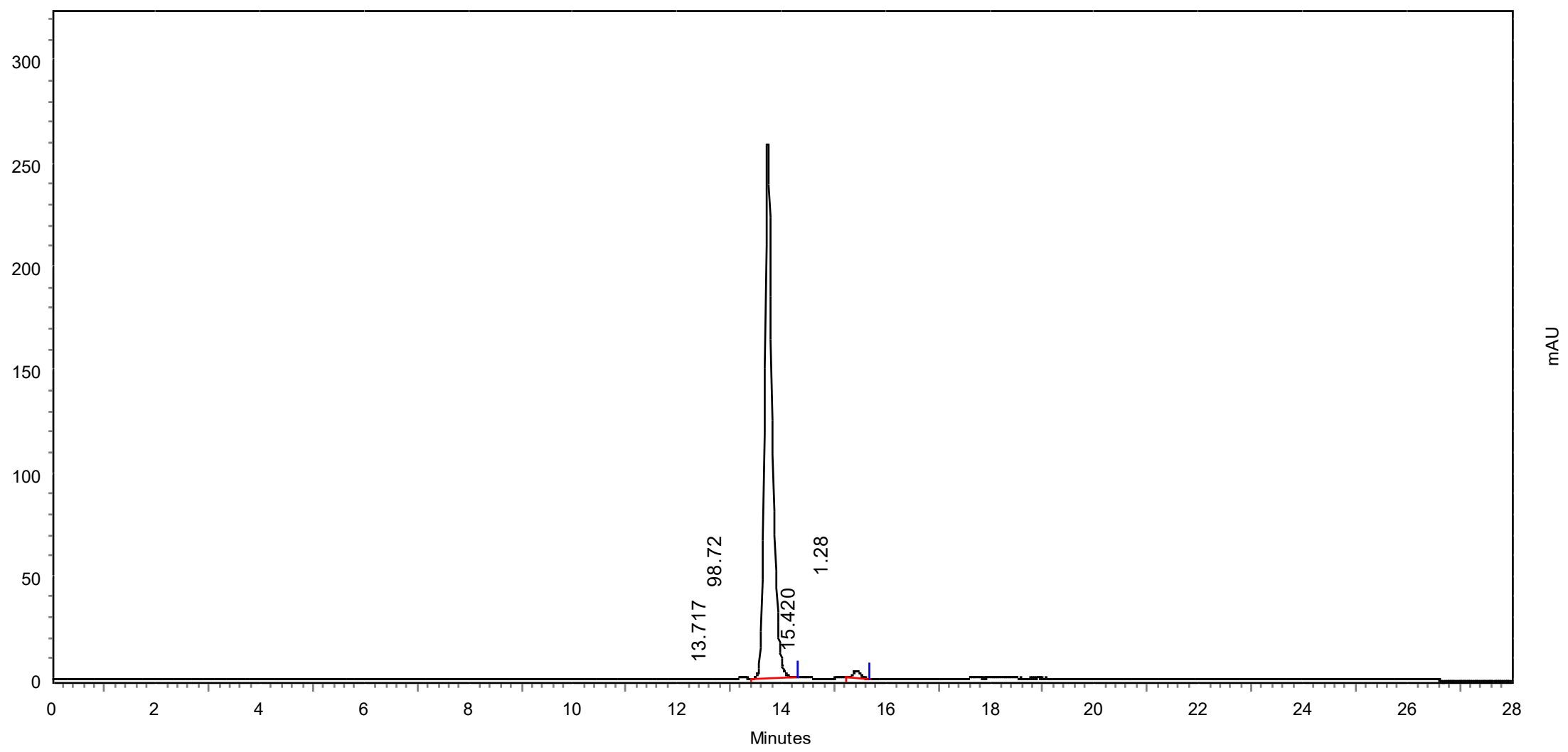

Figure S135. HPLC Chromatogram of **4s**.

HPLC Chromatogram of 4t

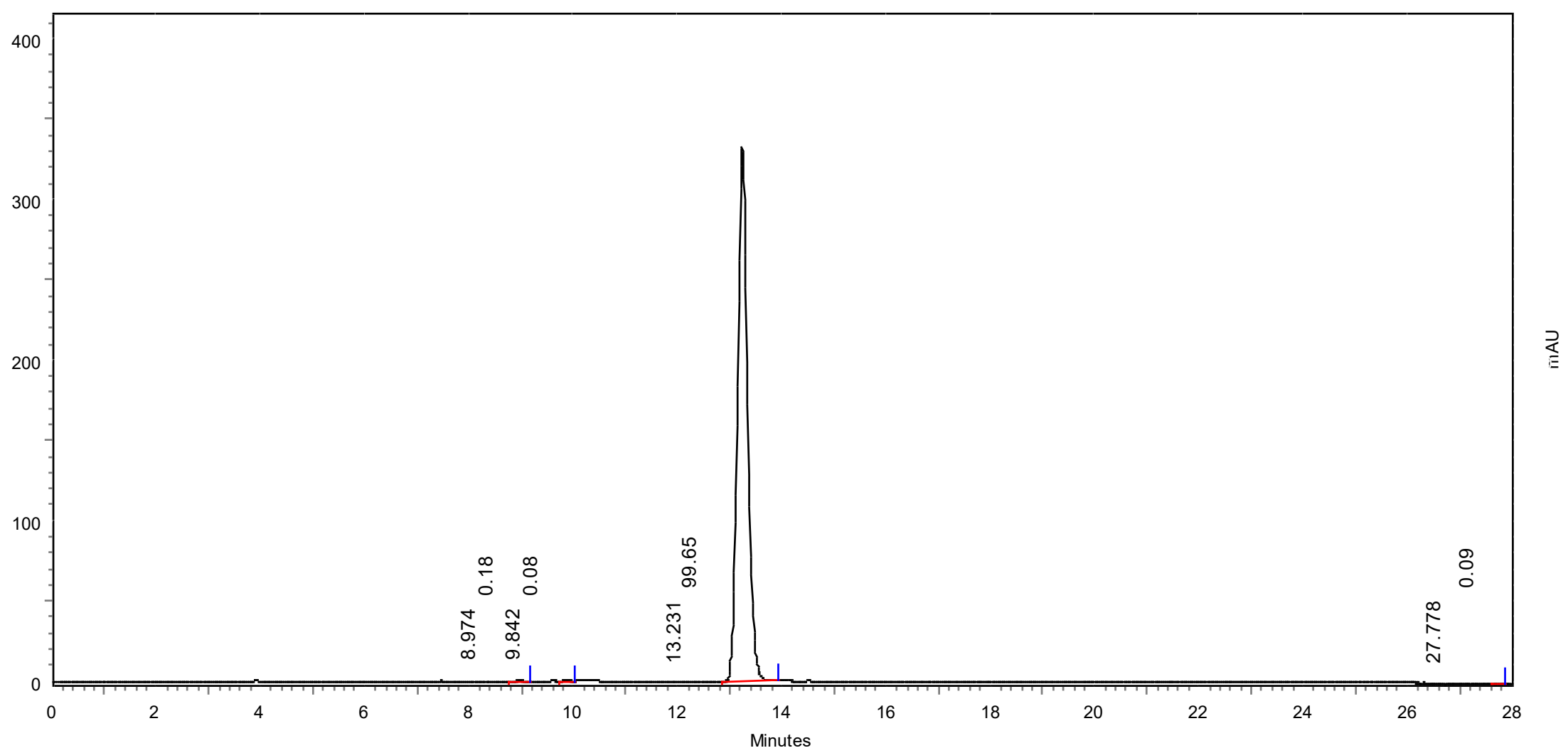

Figure S136. HPLC Chromatogram of **4t**.

HPLC Chromatogram of 5

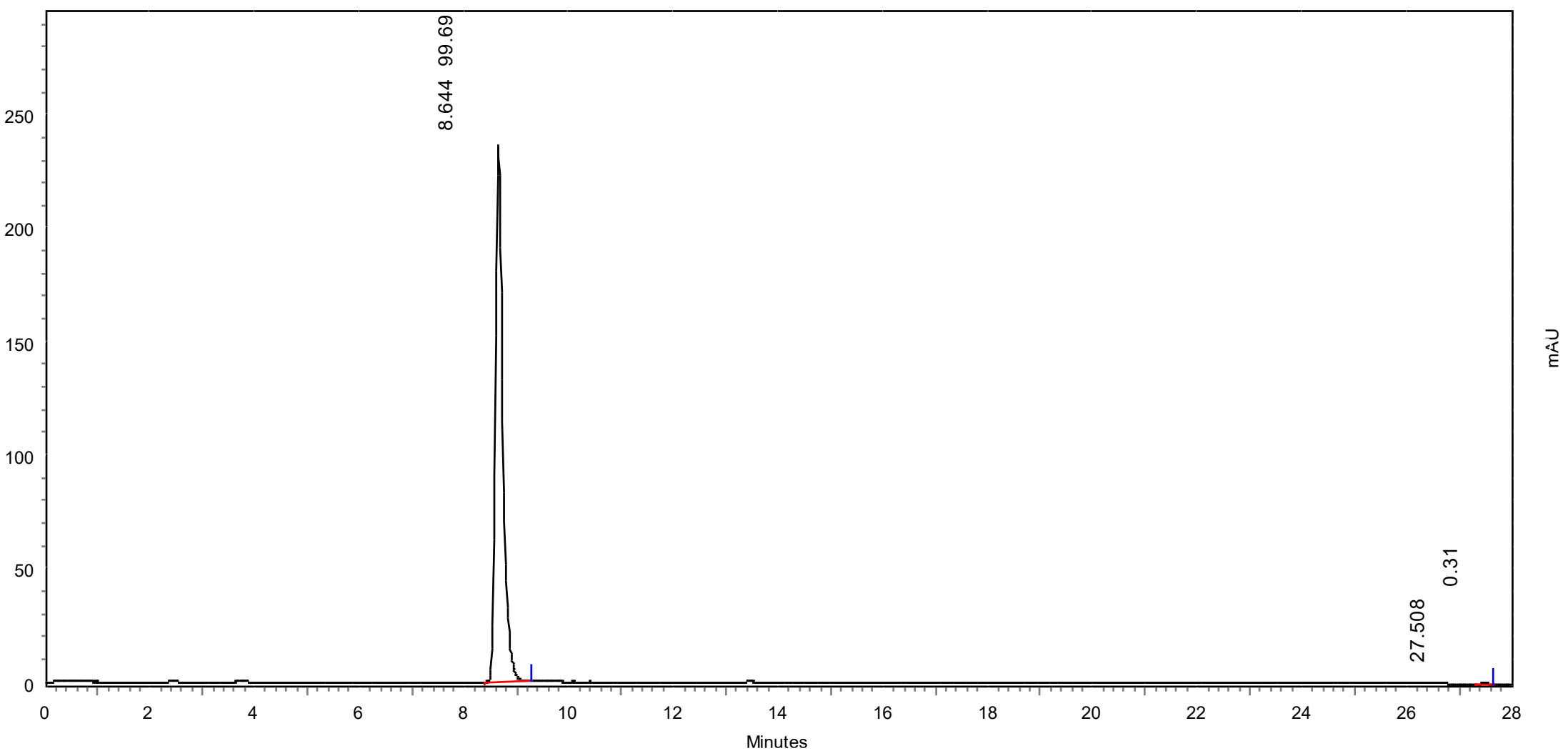

Figure S137. HPLC Chromatogram of **5**.

APPENDIX G: Parasite growth inhibition profile of *L. amazonensis* promastigotes:

Parasite growth inhibition profile of *L. amazonensis* promastigotes incubated with the triazolic derivatives at the indicated concentrations at 26°C. After 72 hours, parasite growth was assessed using rezasurin. Mean ± standard deviation (n = 3).

4a
IC_{50} = 16.90 ± 1.04

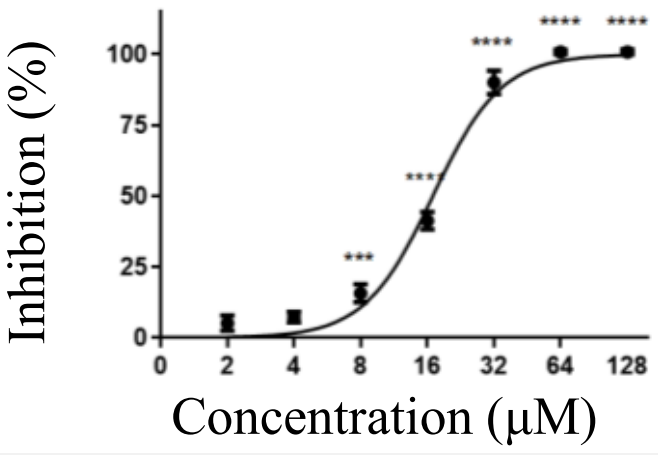

4b
IC_{50} = 9.70 ± 1.03

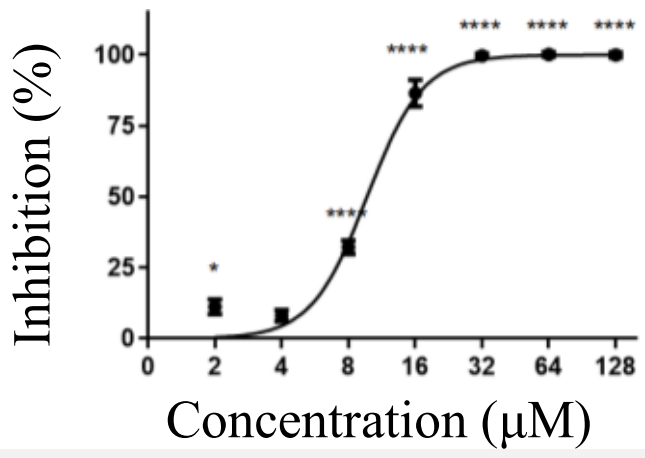

4c
IC_{50} = 10.30 ± 1.04

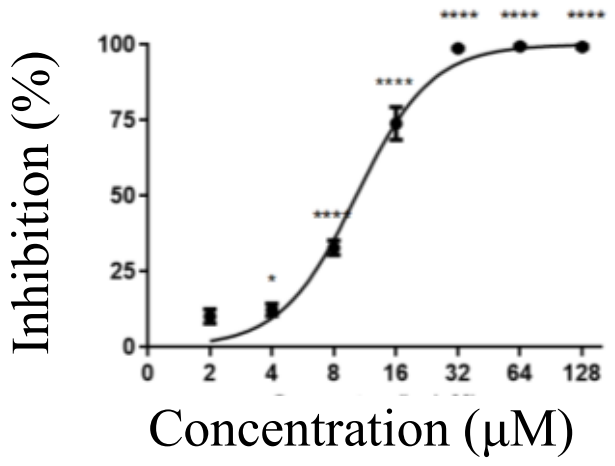

4d
IC_{50} = 11.60 ± 1.08

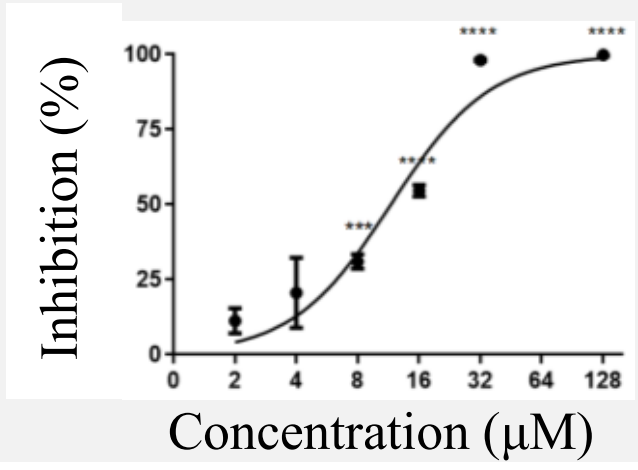

4e
IC_{50} = 14.50 ± 1.05

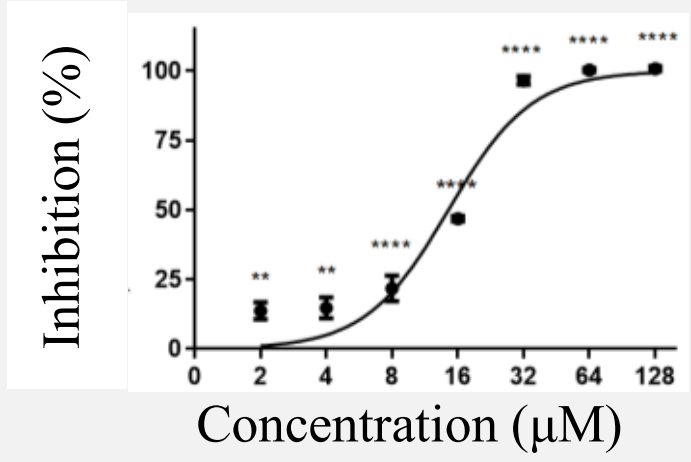

4f
IC_{50} = 31.91 ± 1.12

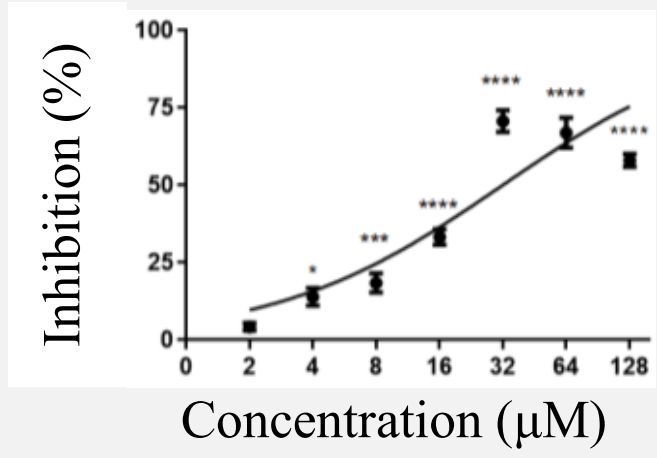

4g
IC_{50} = 18.95 ± 1.04

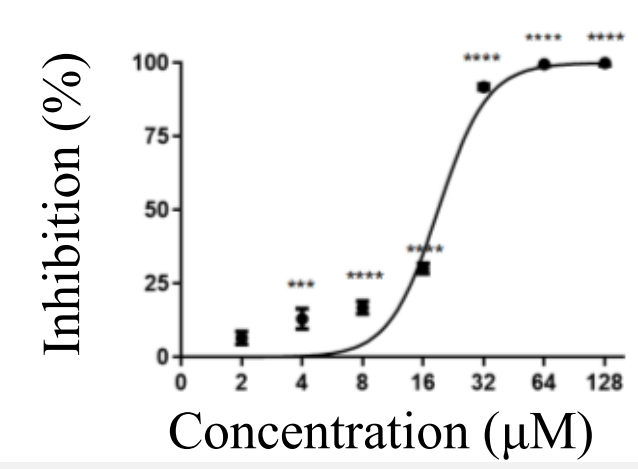

4h
IC_{50} = 27.25 ± 1.03

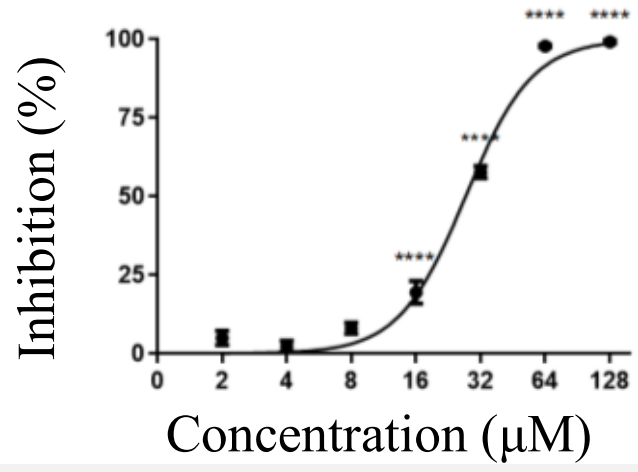

4i
IC_{50} = 22.60 ± 1.04

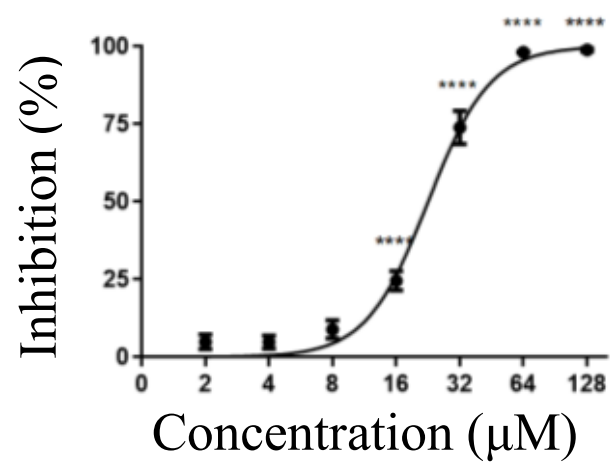

4j
IC_{50} = 16.40 ± 1.02

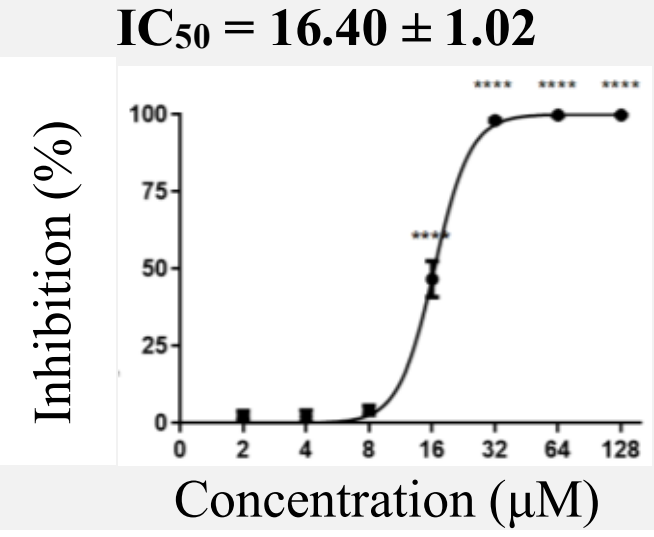

4k
IC_{50} = 17.30 ± 1.03

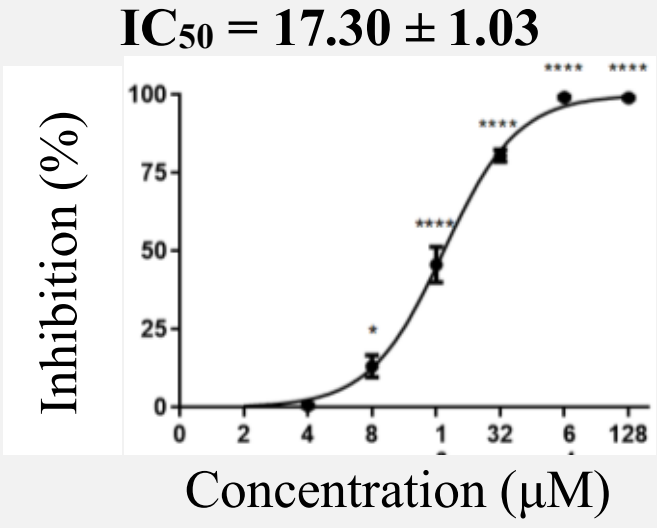

4l
IC_{50} = 18.50 ± 1.03

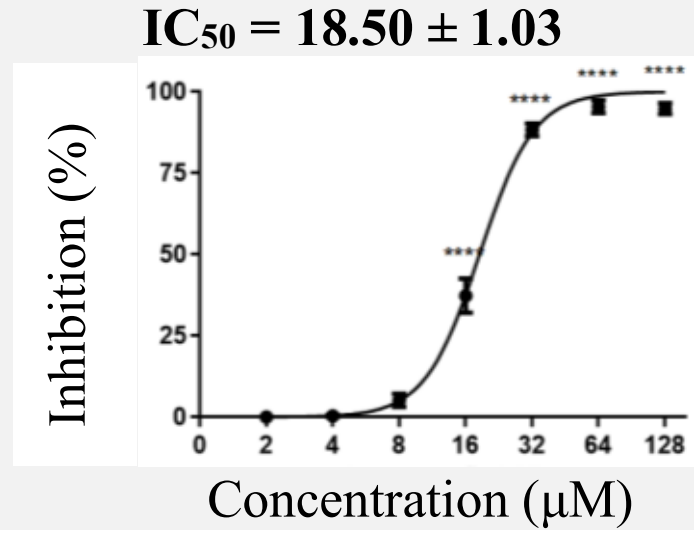

4n
IC_{50} = 63.00 ± 1.04

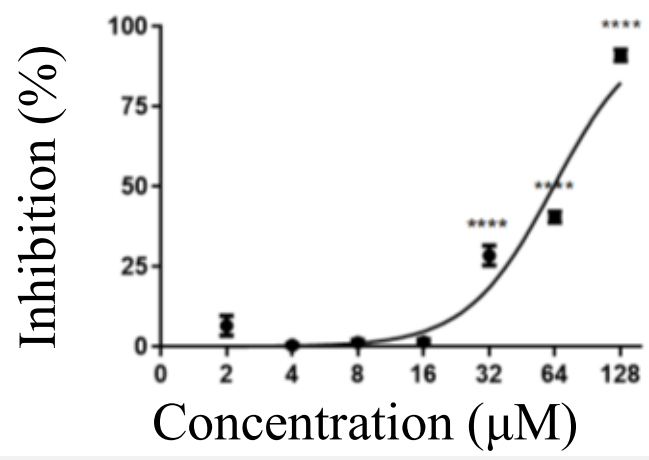

4o
IC_{50} = 97.70 ± 1.16

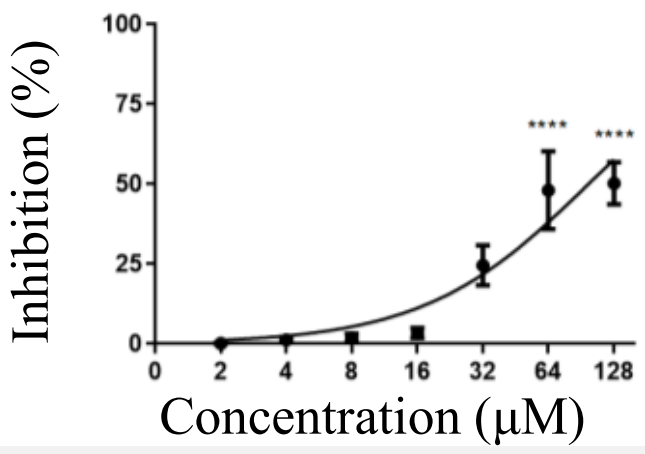

4q
IC_{50} = 27.20 ± 1.04

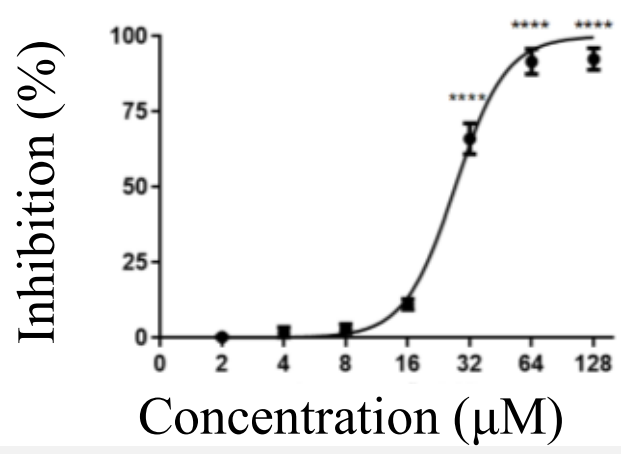

4s **IC_{50} = 35.80 ± 1.02** **4t** **IC_{50} = 37.20 ± 1.03**

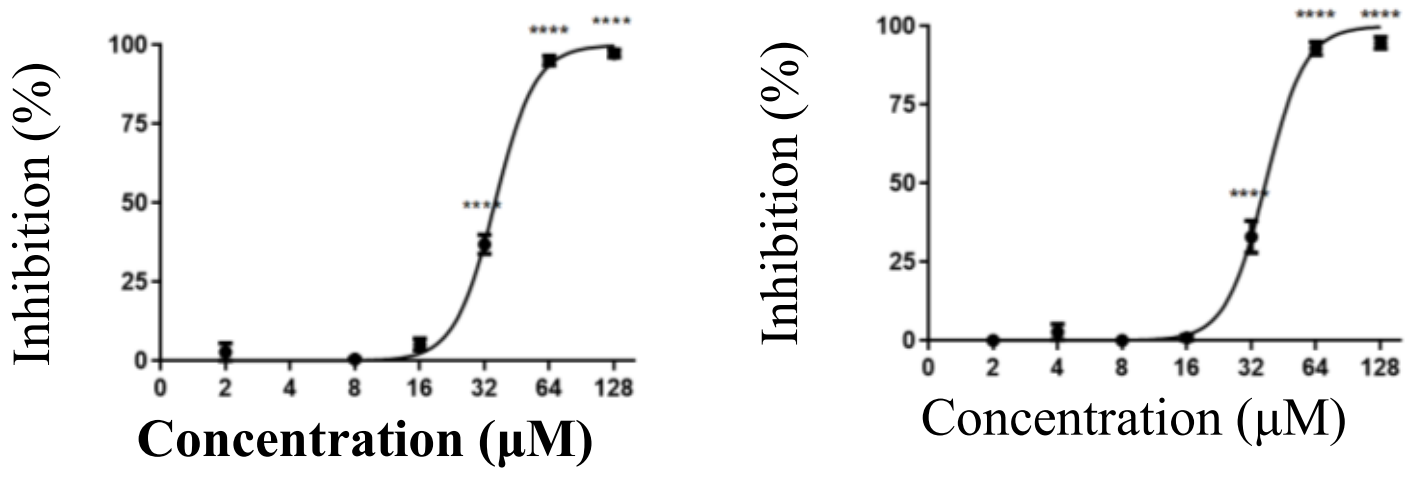

Figure S138. Inhibition curves of test compounds against *L. amazonensis* promastigotes. Data are expressed as mean ± standard error of the mean (SEM) of three independent experiments performed in triplicate. Concentration–response curves were generated by nonlinear regression analysis using GraphPad Prism software to determine IC_{50} values. Statistical significance was assessed by one-way ANOVA followed by Bonferroni's multiple comparison test. Differences were considered statistically significant when $p < 0.05$. Significance levels are indicated as follows: * $p < 0.05$; ** $p < 0.01$; *** $p < 0.001$; **** $p < 0.0001$.

References

[1]ARMAREGO, Wilfred LF. Purification of laboratory chemicals. Butterworth-Heinemann, 2017.

[2]Heredia, D. A.; Larghi, E. L.; Kaufman, T. S. A Straightforward Synthesis of 5-Methylaaptamine from Eugenol, Employing a 6π-Electrocyclization Reaction of a 1-Azatriene. *European Journal of Organic Chemistry* **2016**, 2016 (7),1397-1404, DOI: 10.1002/ejoc.201501566

[3]Zhao, F.; Chen, Z.; Lei, P.; Kong, L.; Jiang, Y. Facile one-pot synthesis of aryl azides from nitrobenzenes. *Tetrahedron Letters* **2015**, 56, 2197–2199, DOI: 10.1016/j.tetlet.2015.03.015

[4]Munagala, G.; Yempalla, K. R.; Singh, S.; Sharma, S.; Kalia, N. P.; Rajput, V. S.; Kumar, S.; Sawant, S. D.; Khan, I. A.; Vishwakarma, R. A.; Singh, P. P. Synthesis of new generation triazolyl-and isoxazolyl-containing 6-nitro-2, 3-dihydroimidazooxazoles as anti-TB agents: in vitro, structure–activity relationship, pharmacokinetics and in vivo evaluation. *Organic & biomolecular chemistry* **2015**, 13 (12), 3610-3624, DOI: 10.1039/C5OB00054H

[5]Pagano, M.; Castagnolo, D.; Bernardini, M.; Fallacara, A. L.; Laurenzana, I.; Deodato, D.; Kessler, U.; Pilger, B.; Stergiou, L.; Strunze, S.; Tintori, C.; Botta, M. The fight against the influenza A virus H1N1: synthesis, molecular modeling, and biological evaluation of benzofurazan derivatives as viral RNA polymerase inhibitors. *ChemMedChem* **2014**, 9 (1), 129-150, DOI: 10.1002/cmdc.201300378

[6]Tabélé, C.; Faiões, V. D. S.; Grimaud, F.; Torres-Santos, E. C.; Khoumeri, O.; Curti, C.; Vanelle, P. Original antileishmanial hits: Variations around amidoximes. *European Journal of Medicinal Chemistry* **2018**, 148, 154-164, DOI: 10.1016/j.ejmech.2018.02.029
